# Proceedings to the 60^th^ Annual Conference of the Particle Therapy Cooperative Group: 27 June – 2 July, 2022, Hosted by Miami Cancer Institute, part of Baptist Health South Florida, Miami, FL, USA

**DOI:** 10.14338/IJPT-23-PTCOG60-9.4

**Published:** 2023-02-14

## Program Description

### Overview

The theme of PTCOG 60, **Optimization of Radiotherapy Modalities**, reflects the transformation of particle therapy from a nascent esoteric technology to a mainstream modality with a much better-defined use case. This transformation has permitted the field to move on from dosimetric evaluations, case studies, and retrospective reports to well-conducted prospective clinical trials and large and robust database analysis incorporating vast numbers of patients and producing real-world evidence, demonstrating not only clinical benefit efficacy, but also cost-effectiveness. Significantly, particle therapy is genuinely integrated with advances in surgical approaches, the development of targeted and immunotherapeutic agents, and entwined with other radiotherapy modalities. PTCOG 60 offered two days of educational and parallel sessions and three days of multi-session scientific reports, as well as integrated subcommittee meetings.

Objectives for the 60^th^ annual conference were to:

- Address individual needs in compliance with their Continuous Professional Development (CPD) plan.
- Discuss the latest technological and clinical innovations in particle therapy.
- Identify educational resources, networks and other for exchange of knowledge and learning about particle therapy,
- Discuss the current research projects within the Particle Therapy Co-Operative Group (PTCOG) and enhance opportunities for future collaboration between groups of young researchers.
- Discuss the latest developments about practical clinical application of particle therapy.
- Describe diagnostics and treatments in the field of particle radiation therapy.

### Target Audience

Healthcare professionals who treat cancer patients using radiation therapy/particle therapy and specifically:

- Radiation Oncologists
- Medical Physicists
- Dosimetrists
- Residents
- Radiation Therapists

### Particle Therapy Cooperative Group (PTCOG) 2022 Committees

***PTCOG Executive and Organizing Committees***

**Jay Flanz, Ph.D., PTCOG Chairman**

**Tadashi Kamada, M.D., PTCOG Vice-Chairman**

**Marco Durante, Ph.D., PTCOG Vice-Chairman**

**Martin Jermann, MSc, PTCOG Secretary**

**Minesh P Mehta, M.D.**, Miami Cancer Institute - Baptist Health South Florida

**Alonso Gutierrez, Ph.D., MBA**, Miami Cancer Institute - Baptist Health South Florida

**Andrew Wroe, Ph.D., DABR**, Miami Cancer Institute - Baptist Health South Florida

**Nikki Mejia,** Miami Cancer Institute - Baptist Health South Florida

***PTCOG60 Scientific Program Subcommittee Members***

**Michael Story, Ph.D.**

*Co-Chairman of Scientific Program Subcommittee, Biology*

**Anita Mahajan, M.D.**

*Co-Chairman of Scientific Program Subcommittee, Clinics*

**Antony J. Lomax, Ph.D.**

*Co-Chairman of Scientific Program Subcommittee, Physics*

***PTCOG60 Educational Subcommittee Members***

**James Metz, M.D.**

*Co-Chairman of Education Subcommittee, Clinics*

**Niek Schreuder, M.Sc. DABR**

*Co-Chairman of Education Subcommittee, Physics & Technology*

### Oral Abstracts O 001 - PARP inhibition combined with proton radiation enhance cell kill and senescence leading to immune signaling

#### Mariam Ben Kacem^1^, Emma Moran^1^, David B. Flint^1^, David Martinus^1^, Broderick X. Turner^1^, Scott J. Bright^1^, Mandira Manandhar^1^, Poliana C. Marinello^1^, Tingshi Su^1^, Simona F. Shaitelman^2^, Gabriel O. Sawakuchi^1^

##### *^1^MD Anderson Cancer Center, Department of Radiation Physics, Houston, USA*

*^2^MD Anderson Cancer Center, Department of Radiation Oncology, Houston, USA*

**Introduction:** PARP inhibitors (PARPi) have improved survival for BRCA mutated breast cancers (BC). It is unclear how distinct forms of radiation differentially interact with PARPi and if they can be used to further improve outcomes. Protons induce more DNA lesions than x-rays due to their higher ionizing density. If not properly repaired, DSBs can generate micronuclei or induce cellular senescence, both affecting immune response. We investigated the effects of combining PARPi with x-rays or protons on clonogenic cell survival, micronuclei and senescence in the context of BRCA1 mutation.

**Methods**: Clonogenic survival, quantified micronuclei/nucleus and radio-induced senescence (β-galactosidase activity) data for human BC cell lines with a BRCA1 mutation (HCC1937, MDA-MB-436) and their isogenic cell lines (HCC1937-BRCA, MDA-MB-436-BRCA) were obtained. Cells were irradiated with 6 MV x-rays or 9.9 keV/mm protons (dose-weighted LET in water) ± PARPi (Olaparib).

**Results**: Survival fraction was lower after protons compared to x-rays. PARPi treatment appears to result in greater radiosensitization after proton compared to x-ray radiation as assessed by survival assay. The number of MN/nucleus was significantly higher in HCC1937 than HCC1937-BRCA. Senescence was significantly higher for HCC1937 compared to HCC1937-BRCA. Radio-induced senescence is not affected by PARPi treatment but is amplified after proton radiation compared to x-rays. Data for MDA-MB-436 (BRCA+/-) are being collected.

**Conclusions**: In vitro, PARPi treatment in BRCA1 mutated cell lines leads to a greater sensitivity to radiation. This sensitivity is higher after protons compared to x-rays (survival fraction, micronuclei and senescence), but understanding of associated immune effects warrants further investigation.

### O 002 - Defective homologous recombination predicts increased responsiveness to carbon ion radiotherapy

#### Anthony Davis^1^, Brock Sishc^1^, Janapriya Saha^1^, Katy Swancutt^1^, Lianghao Ding^1^, Elizabeth Polsdofer^1^, Angelica Facoetti^2^, Benjamin Chen^1^, Debabrata Saha^1^, Mario Ciocca^2^, Todd Aguilera^1^, Michael Story^1^

##### *^1^University of Texas Southwestern Medical Center, Radiation Oncology, Dallas, USA*

*^2^Medical Physics Unit & Research Department- Foundazione Centro Nazionale di Adroterapia Oncologica CNAO, Radiation Oncology, Pavia, Italy*

Pancreatic ductal adenocarcinoma (PDAC) is a highly lethal malignancy that is expected by 2020 to become the second most common cause of cancer-related deaths globally. The 5-year overall survival rate for patients with PDAC is <10%, which is due to the majority of pancreatic cancer patients presenting with advanced disease and the inherent resistance of PDAC to conventional chemotherapy and radiotherapy. Clinical trials have suggested that carbon ion radiotherapy (CIRT) used concurrently with gemcitabine is effective against unresectable locally advanced PDAC. We have initiated pre-clinical studies to identify patients who would most likely benefit from CIRT. In particular, we have focused on examining the role of an aberrant DNA damage response (DDR) in PDAC treatment responsiveness. This is supported by exome sequencing data of >150 surgically excised PDACs that showed that >35% of PDAC tumors have an alteration in a DDR gene and that these alterations correlate with a poor outcome. In this study, we utilized a panel of PDAC cell lines, whose survival fraction of 2 Gy (γ-rays) ranged from average to radioresistant, to determine if CIRT can overcome photon radioresistance. We identified that cell lines defective in the DNA double-strand break repair pathway homologous recombination (HR) pathway are exquisitely sensitive to CIRT. Furthermore, targeting a radioresistant PDAC cell line with the HR inhibitor B02 results in marked increased sensitivity to CIRT *in vitro*. Finally, we will present initial results showing the identification of DDR-related biomarker(s) that predict responsiveness to CIRT.

### O 003 - Inhibition of Glutaminase-1 (GLS1) increases the relative biological effectiveness of proton radiotherapy and increases levels of lipid peroxidation

#### Scott Bright^1^, Rishab Kolochina^1^, David Flint^1^, David Martinus^1^, Mandira Manandhar^1^, Mariam Ben Kacem^1^, Poliana C Marinello^1^, Tingshi Su^1^, Simona F Shaitelman^2^, Gabriel Sawakuchi^1^

##### *^1^MD Anderson, Radiation Physics, Houston, USA*

*^2^MD Anderson, Radiation Oncology, Houston, USA*

Glutaminase-1 (GLS1) is important in redox homeostasis, producing glutamate from glutamine. GLS1 downstream products include nicotinamide adenine dinucleotide phosphate (NADPH) and glutathione (GSH). GSH serves as a first line defense against oxidative stress by neutralizing reactive oxygen species and as such is an important factor in radiation response. Many cancers display dysregulated glutamine metabolism compared to normal tissue and GLS1 has therefore proved an attractive therapeutic target with several GLS1 inhibitors in development. We hypothesized the disruption of GSH by GLS1 inhibition would render cells radiosensitive. We performed clonogenic survival and lipid peroxidation assays in NCI-H460 cells exposed to 6 MV x-rays and 9.9 keV/μm protons to determine the radiosensitization and lipid peroxidation levels induced by IACS-6274 treatment, a GLS1 inhibitor discovered at MD Anderson and currently in clinical trials. We observed that IACS-6274, could radiosensitize NCI-H460 to x-rays and protons (Figure 1A). We also observed that the relative biological effectiveness (RBE) was significantly increased in the presence of IACS-6274 compared to vehicle (Figure 1B). Our data also suggest that protons combined with IACS-6274 leads to the greatest increase in lipid peroxidation (Figure 2), a known step in the cell death pathway, ferroptosis. These results suggest protons synergize with GLS1 inhibition. It is therefore an attractive combination for further development. Our results also suggest that cell death may occur through ferroptosis, which is more prevalent in cancer cells compared to normal tissue.

### O 004 - Relating proton LETd to biological response of salivary glands using PSMA-PET in clinical patients

#### Dirk Wagenaar^1^, Roel J.H.M. Steenbakkers^1^, Johannes A. Langendijk^1^, Vineet Mohan^2^, Wouter Vogel^2^, Stefan Both^1^

##### *^1^Department of Radiation Oncology- University Medical Center Groningen- University of Groningen, Radiation Oncology, Groningen, Netherlands*

*^2^Dutch Cancer Institute NKI, Radiation Oncology, Amsterdam, Netherlands*

**Purpose**: A recent study investigated the use of PSMA-PET in monitoring loss of secretory cells in salivary glands of head and neck cancer (HNC) patients. The purpose of the current study was to measure variations in RBE in salivary glands of patients treated with proton therapy.

**Methods**: Six patients treated with proton therapy were included. These patients received a PET-CT scan using ^68^Ga (N=1) or ^18^F (N=5) PSMA before treatment (baseline) and one month after the last fraction (follow-up). All analyses were performed in RayStation v9AR (v8.99.30.71). Physical dose (D_phys_), D*LET_d_ and the follow-up PSMA-PET scan were deformed to the baseline PET-CT using deformable image registration. Parotid gland delineations were adjusted to include voxels which had an uptake of ≥5 g/ml in the baseline PSMA-PET scan. Voxels were smoothed in a 6-mm cube to reduce effects of noise and registration errors. Relative PSMA uptake was defined as the ratio of uptake on the follow-up and baseline PSMA-PET scans. The LET_d_ was calculated from the D*LETd and D_phys_.

**Results**: In four out of six patients a relation between relative PSMA-uptake and LET_d_ could be observed, where the relative PSMA uptake was lower as voxels had higher LET but identical physical dose (Figure 2).

**Conclusion**: An RBE-LET_d_ relation is shown for the biological response of parotid glands as measured using PSMA-PET. This study shows PSMA-PET can be used to investigate the spatial distribution of RBE of the salivary glands in a clinical setting.

### O 005 - Early cellular response to pMBRT: a mechanistic study

#### Annaïg Bertho^1^, Ramon Ortiz^1^, Cristèle Gilbert^1^, Julie Espenon^1^, Marjorie Juchaux^1^, Annalisa Patriarca^2^, Ludovic De Marzi^2^, Yolanda Prezado^1^

##### *^1^Institut Curie, U1021/UMR 3347, Orsay, France*

*^2^Institut Curie, Hospital complex, Orsay, France*

Proton minibeam radiation therapy (pMBRT) is a novel therapeutic approach which uses spatially modulated narrow (< 1 mm) proton beams [1]. In comparison to conventional irradiations, pMBRT has displayed a remarkable reduction in neurotoxicity [2] and an equivalent or superior tumor control in small animals [3]. All of this despite the highly heterogeneous dose distributions - challenging the dogma that a homogenous dose is required to achieve tumor control. These aforementioned distributions consist of high (peak) doses along the path of the minibeams and low (valley) doses in between them. While the underlying biological mechanisms responsible for pMBRT efficiency remain elusive, our previous *in vivo* evaluation highlighted a significant involvement of the immune system in the response to pMBRT [4]. Consequently, we conducted an *in vitro* mechanistic study on cell death and immunogenicity of pMBRT using astrocyte and glioma cell lines. We observed that pMBRT induced more apoptosis compared to standard proton therapy (PT) at 24h after irradiation in the astrocytes. Late apoptosis was also increased compared to standard PT, indicating that the timing of cell death can be impacted by pMBRT. Immunogenic cell death can mimic morphological and biochemical features of apoptosis [5]. In contrast, no difference in cell death was observed in the glioma cell line as a function of the irradiation mode, thus suggesting that the mean minibeam dose might indeed be a good indicator for tumor response. The analysis of key immunogenic factors such as ATP, HMGB1 and Calreticulin is ongoing and will also be presented.

### O 006 - A multimodal 3D image registration toolkit to investigate proton radiation-induced brain injury

#### Marc Boucsein^1,2^, Sindi Nexhipi^3,4^, Robin Koch^1,2^, Theresa Suckert^3,4,5^, Elke Beyreuther^3,6^, Antje Dietrich^3,5^, Armin Lühr^3,4,5,7^, Emanuel Bahn^1,2,8,9^

##### *^1^German Cancer Research Center DKFZ, Clinical Cooperation Unit Radiation Oncology, Heidelberg, Germany*

*^2^Heidelberg University Hospital, Radiation Oncology, Heidelberg, Germany*

*^3^OncoRay - National Center for Radiation Research in Oncology, Faculty of Medicine and University Hospital Carl Gustav Carus, Dresden, Germany*

*^4^Helmholtz-Zentrum Dresden-Rossendorf, Institute of Radiooncology - OncoRay, Dresden, Germany*

*^5^German Cancer Consortium DKTK, Partner Site Dresden- and German Cancer Research Center DKFZ, Heidelberg, Germany*

*^6^Helmholtz - Zentrum Dresden-Rossendorf, Institute of Radiation Physics, Dresden, Germany*

*^7^TU Dortmund University- Faculty of Physics, Medical Physics and Radiotherapy, Dortmund, Germany*

*^8^National Center for Tumor Diseases NCT, Integrative Radiation Oncology, Heidelberg, Germany*

*^9^Heidelberger Institut für Radioonkologie HIRO, Quantitative klinische Strahlenbiologie, Heidelberg, Germany*

**Purpose**: In proton brain radiotherapy, late radiation-induced injuries represent serious complications. To investigate their formation, we use a mouse model and irradiated brain sub-regions with a highly collimated proton Bragg peak. By co-aligning the radiation input distributions (dose and LET) with fluorescently stained histological slices, we aim to perform dose-response analysis on a single-cell level throughout the whole brain. This allows detailed insights into how proton radiation causes cellular changes associated with late radiation-induced injuries.

**Methods and Results**: As manual processing is time-consuming, our goal was to accelerate, automatize and optimize the preprocessing and registration steps. We have developed a toolkit for automated co-registration of histological slices with CT, MRI and histology- or MRI-based atlases, consisting of three main parts: First the preprocessing, second, histological slices are reconstructed in 3D, and third the automated registration of the different image modalities. We used self-developed packages (https://github.com/DKFZ-TMTRR) within the python-based viewer NAPARI, which are designed for memory-efficient analysis of large image data sets, allow efficient monitoring throughout the whole process, and offer the option of manual interventions.

**Discussion**: In summary, we have developed an image registration toolkit to combine clinically relevant brain image modalities in one interactive registration pipeline. Altogether, this yields an accurate co-alignment of the brain with the administered dose distributions within one reference coordinate system, minimizing spatial inaccuracies between the modalities, as presented in Figures 1&2. As a result, radiation input parameters are defined for every single cell with high confidence, allowing for statistical analysis of radiation effects on an unprecedented level of detail.

### O 007 - Predicting radiation-induced lymphopenia in lung cancer patients across institutions after treatment with photon- and proton-based radiotherapy

#### Yejin Kim^1,2,3^, Ibrahim Chamseddine^2^, Ting Xu^4^, Steven H. Lin^4^, Zhongxing Liao^4^, Yeona Cho^3^, Jin Sung Kim^3^, Hong In Yoon^3^, Seungryong Cho^1^, Clemens Grassberger^2^

##### *^1^KAIST, Nuclear and Quantum Engineering, Daejeon, Korea Republic of*

*^2^Massachusetts General Hospital and Harvard Medical School, Radiation Oncology, Boston, USA*

*^3^Yonsei Cancer Center, Radiation Oncology, Seoul, Korea Republic of*

*^4^MD Anderson Cancer Center, Radiation Oncology, Houston, USA*

**Purpose**: Radiation-Induced Lymphopenia (RIL) has been correlated to inferior survival and hypothesized to adversely impact adjuvant immunotherapy. This study aims to develop and compare RIL prediction models after both Intensity Modulated Radiation Therapy (IMRT) or Proton Therapy (PT) for locally-advanced lung cancer.

**Methods and Materials**: In total, 178 patients were collected; 45 IMRT patients from Yonsei Cancer Center and 92 IMRT and 41 PT patients from MD Anderson Cancer Center. Clinical variables and Dose Volume Histograms (DVHs) of normal lung and heart were used to predict Grade(G)4 RIL at the end of radiotherapy. Logistic Regression (LR), Random Forests (RF) and a novel Convolutional Neural Network (CNN) that considers the entire DVHs using convolution layers were developed. Models were compared to each other using the area under the Receiver Operating Characteristics curve (AUC).

**Results**: Incidence of G4RIL was 16%/10% after IMRT/PT respectively. For IMRT, predicting G4 RIL using CNN outperformed other methods, increasing the average AUC from 0.69 to 0.74. Model performance significantly decreased when including PT patients for all models to 0.62-0.65, with only small outperformance of CNNs. Standard deviations indicate lower model robustness for mixed IMRT/PT cohorts (0.04) compared to IMRT only (0.02).

**Conclusion**: CNN approaches to include DVH information to predict RIL are successful in IMRT patients, but inhomogeneity in DVHs and baseline patient heterogeneity in combined IMRT/PT cohorts affects model training and robustness. Further research is required to determine if this is due to fundamental differences in the interaction with circulating lymphocytes between IMRT and PT.

### O 008 - 12C radiotherapy for individuals whose tumors are considered radioresistant may require personalization of RBE to be fully potent

#### Michael Story^1^, Brock Sishc^1^, Anthony Davis^1^, Lianghao Ding^1^, Elizabeth Polsdofer^1^, Angelica Facoetti^2^, Mario Ciocca^3^, Arnold Pompos^1^, Debabrata Saha^1^

##### *^1^University of Texas- Southwestern Medical Center, Radiation Oncology, Dallas, USA*

*^2^Foundation CNAO, Medical Physics and Biology, Pavia, Italy*

*^3^Foundation CNAO, Medical Physics, Pavia, Italy*

Head and neck squamous cell carcinoma is the sixth most common malignancy worldwide. 30% of patients experience locoregional recurrence with median survival less than one year. Factors contributing to treatment failure include inherent resistance to X-rays, hypoxia and immune suppression amongst others. However, the unique properties of ^12^C radiotherapy make its application well suited to the treatment of HNSCC. We examined the ^12^C radioresponse of five HNSCC cell lines, whose surviving fraction at 3.5 Gy ranged from average to resistant when compared to a larger panel of 38 cell lines to identify biomarkers predictive of low LET radioresistance. A 37 gene signature was able to place cells in their respective radiosensitivity cohort with an accuracy of 86% which can be reduced to 13 genes if only segregating radioresistant cell lines against all others. Subsequently, Cells were irradiated with ^12^C using a SOBP with an average LET of 80 keV/μm. RBE values varied depending upon endpoint used. Radioresistant cells were characterized by an enrichment of genes associated with radioresistance and survival mechanisms including but not limited to G2/M Checkpoint MTORC1, HIF1a, and PI3K/AKT/MTOR signaling. These data were used in an *in silico*-based modeling approach to evaluate tumor control probability after ^12^C irradiation that compared clinically used treatment schedules with fixed RBE values vs the RBEs determined for each cell line. The modeling suggests that there is a likelihood that using a generalized RBE will not provide a fully potent therapy to individuals with radioresistant disease irrespective of selection for ^12^C radiotherapy.

### O 009 - Boron Neutron Capture Therapy (BNCT) update: current status of clinical trials, accelerator technologies, drug carriers and treatment planning strategies

#### Will Jin^1^, Crystal Seldon^1^, Michael Butkus^1^, Wolfgang Sauerwein^2^, Huan Giap^3^

##### *^1^University of Miami Sylvester Comprehensive Cancer Center, Radiation Oncology, Miami, USA*

*^2^Deutsche Gesellschaft für Bor-Neutroneneinfangtherapie DGBNCT, Radiation Oncology, Essen, Germany*

*^3^Nancy N and JC Lewis Cancer and Research Pavilion, Radiation Oncology, Hilton Head Island, USA*

Boron neutron capture therapy (BNCT) is based on the nuclear capture and fission reactions that occur when 10B is irradiated with low-energy (0.0025 eV) thermal neutrons. The resulting 10B(n,α)7Li capture reaction produces high linear energy transfer (LET) α particles, helium nuclei (4He) and recoiling lithium-7 (7Li) atoms. The short range (5-9 μm) of the α particles limits the destructive effects within the boron-containing cells. In theory, BNCT can selectively destroy malignant cells while sparing adjacent normal tissue at the cellular levels by delivering a single fraction of radiation with high LET particles. Early clinical works on BNCT are based on unoptimized neutron sources from nuclear reactors, limited carriers with BPA and BSH, and crude treatment planning approaches. A total of more than 200 patients were reported on 17 clinical trials for GBM, head and neck, and melanoma which are shown in Table 1. Table 2 showed current prospective trials. Recent BNCT re-emergence are due to several developments: (1) small footprint accelerator-based neutron sources, (2) high specificity third generation Boron carriers based on monoclonal antibodies, nanoparticles, etc. (3) treatment planning software and patient positioning devices that optimize treatment delivery and consistency. There are currently 4 vendors (2 from US, and 2 from Japan) providing accelerators that are optimized for clinical uses with custom neutron spectrum, beam shaping assembly, patient positioning devices, IGRT, and quantitative in-vivo imaging. Current treatment planning approaches are done with pre-treatment PET/CT scan and Monte Carlo modeling with novel drug carriers with high tumor and normal tissue ratio will be discussed.

### O 010 - Global review of BNCT clinical experience and future prospects

#### Hanna Koivunoro^1^, Leena Kankaanranta^2^, Tetsuya Yamamoto^3^, Shinji Kawabata^4^, Heikki Joensuu^2^

##### *^1^Neutron Therapeutics, Medical Physics, Helsinki, Finland*

*^2^Helsinki University Hospital and University of Helsinki, Department of Oncology, Helsinki, Finland*

*^3^Yokohama City University Hospital, Department of Neurosurgery, Yokohama, Japan*

*^4^Osaka Medical and Pharmaceutical University, Department of Neurosurgery, Osaka, Japan*

In boron neutron capture therapy (BNCT), targeted high-LET radiotherapy to cancerous tissues is achieved by combining external irradiation with low energetic neutrons and a carrier drug that transports boron-10 selectively into cancer cells. First clinical BNCT studies were carried out using a nuclear research reactor as the neutron source in the absence of medical devices capable of producing a high intensity neutron beam in the clinic. The reactor-based phase I/II trials and case studies supported the concept of BNCT in the treatment of several types of human cancer including glioblastoma, head and neck cancer, lung cancer, breast cancer, hepatoma, some sarcomas, and cutaneous malignancies, and in extramammary Paget's disease and meningioma, despite the studies were often carried out in patient populations with inoperable/recurrent disease. Single-agent L-boronophenylalanine (L-BPA) was the most frequently used boron carrier compound, sometimes in combination with borocaptate sodium (BSH). In phase I/II studies on locally recurrent or advanced head and neck cancer, about 70% of the patients achieved either complete or partial response with BNCT, and many of the remaining patients had stable disease as their best response suggesting substantial activity for L-BPA-based BNCT in this patient population. At present, several types of accelerator-based neutron sources are under an approval process for medical use worldwide, and one such device has already been approved in Japan. In this review we summarize the clinical results obtained with BNCT including data from the published and still on-going accelerator-based trials and discuss of the future of BNCT delivery in hospital environments.

### O 011 - The NECTAR project: Could neutron capture therapy be the unexpected weapon to fight Alzheimer's disease?

#### Valeria Pascali^1,2^, Saverio Altieri^1,2^, Diego Alberti^3^, Valeria Bitonto^3^, Sebastiano Maria Salomone Micocci^3^, Simonetta Geninatti-Crich^3^, Annamaria Deagostino^4^, Emanuele Azzi^4^, Polyssena Renzi^4^, Nicoletta Protti^1,2^

##### *^1^University of Pavia, Physics Department, Pavia, Italy*

*^2^National Institute of Nuclear Physics INFN, Pavia Unit, Pavia, Italy*

*^3^University of Torino, Department of Biotechnology and Health Sciences, Torino, Italy*

*^4^University of Torino, Department of Chemistry, Torino, Italy*

NECTAR (NEutron Capture Enhanced Treatment of neurotoxic Amyloid aggRegates) project, funded by the European Commission, aims to study the effectiveness of low-energy neutron-induced capture reactions in the degradation of β-amyloid (Aβ) protein aggregates involved in Alzheimer's disease (AD). AD is the most common form of dementia characterized by the accumulation of Aβ aggregates in the brain. After several decades of discomforting trials, last year aducanumab, a monoclonal antibody able to block the progression of AD, was approved by USA-FDA. Nonetheless, several aspects of AD pathogenesis and progression are unclear thus further studies and innovative treatments must be pursued. The innovative idea of the NECTAR project is to exploit the secondary particles produced by the neutron capture reactions (on B-10 and Gd-157) to destroy the Aβ aggregates, which size couple well to the short range of reaction products, promoting their depolymerization. The gamma produced by neutron capture reactions will be investigated as a promoter of the Aβ clearance by the glia cells. On behalf of NECTAR Consortium, the talk will present the preliminary results achieved. In particular, the first in vitro experiments conducted at the nuclear reactor of Pavia University, accompanied by Monte Carlo simulations focused on a first evaluation of microdosimetric energy deposition. In view of the future developments of the NECTAR project, macroscopic scale simulations will also be presented in order to describe a first study concerning the irradiation effects on AD transgenic mice.

### O 012 - Contributions to BNCT dosimetry in normal lung through optical density analysis in autoradiographic images

#### María Sol Espain^1,2^, Agustina Portu^1,2^, Marcela Alejandra Garabalino^2^, María Silvina Olivera^3^, Gustavo Santa Cruz^3^, Verónica Andrea Trivillin^1,2^, Paula Ramos^2^, Ana Mailén Dattoli Viegas^1,4^, Andrea Monti Hughes^1,2^, Sergio Ferraris^5^, Guillermo Belerenian^5^, Fernando Lange^5^, Gisela Saint Martin^2^, Emiliano César Cayetano Pozzi^6^, Paula Curotto^6^, Silvia Inés Thorp^7^, Amanda Elena Schwint^1,2^, Sara Josefina González^2,4^

##### *^1^National Research Council CONICET, n/a, Buenos Aires, Argentina*

*^2^National Atomic Energy Commission, Radiobiology, Buenos Aires, Argentina*

*^3^National Atomic Energy Commisssion, BNCT Department, Buenos Aires, Argentina*

*^4^National Atomic Energy Commission, Computational Dosimetry and Treatment Planning Division- BNCT Department, Buenos Aires, Argentina*

*^5^Maimonides University, Center of Research and Development of Integral Models CIDME, Buenos Aires, Argentina*

*^6^National Atomic Energy Commission- Ezeiza Atomic Center, Research and Production Reactors, Buenos Aires, Argentina*

*^7^Ezeiza Atomic Center- National Atomic Energy Commission, Instrumentation and Control Department, Buenos Aires, Argentina*

Within the context of the multi-institutional Argentine project “Therapeutic Potential of BNCT for the Treatment of Multiple Lung Metastases: Feasibility and therapeutic efficacy study at an experimental level of a novel *ex-vivo* irradiation protocol”, the validity of the ovine model as an adequate human surrogate in terms of boron kinetics in clinically relevant tissues was established [1], and neutron autoradiography technique was extended to study the boron microdistribution in normal and metastatic lung from BDIX rats [2]. A new boron biodistribution study of a normal sheep was performed, infusing boronophenylalanine-fructose (350mg/kg) for 40 min, and samples of lung and other tissues were extracted for boron determination. ICP-OES measurements were carried out, and sections of normal lung were processed to obtain autoradiographic images. Ongoing thermographic analysis seeks to correlate the boron distribution in the organ with its thermal evolution during cryopreservation. At the microscopic level, a preferential uptake of BPA in the broncho-vascular tree was observed (Figure 1). Optical density measurements were performed in order to study this trend, as it could explain potential vascular damage in normal lung after BNCT. These results will be used to contribute to dosimetry optimization in a clinical scenario. Computational simulations were used to study the phenomenon of heat transport in the lung volume and thus understand certain observed spatial correlations between temperature and boron concentration.

### O 013 - Development of a novel accelerator system and new targeted drugs for BNCT

#### Kendall Morrison^1^, Michael Torgov^2^

##### ^1^TAE Life Sciences, Drug Development, Santa Monica, USA

BNCT development has been hindered by a number of practical hurdles, particularly the need for a nuclear reactor as a source of neutrons and the reliance on poorly soluble boronophenylalanine (BPA) for boron delivery. At TAE Life sciences we are developing a new neutron source and drugs to overcome these hurdles. We have developed a compact, low energy neutron spectrum (2.5 MeV proton energy) tandem accelerator operating at 10mA with a lithium target generating neutrons in the epithermal spectrum ideal for BNCT. We are currently completing the installation of the first of these tandem accelerators at a large hospital in Xiamen, China. In addition to developing this modern neutron source we are also testing novel boronated small molecules as alternatives to BPA. These have been designed to have good solubility, cellular uptake and retention. These compounds have been extensively in vitro and in vivo and we have shown that, in addition to their easier formulation, they can deliver 2-3 times more boron across multiple cell lines and tumor models than can be achieved with BPA. We will present design features of our tandem accelerator as well as in vitro and in vivo data regarding boron delivery using our novel compounds.

### O 014 - Variable RBE for meningioma helium ion therapy: from the LBNL experience toward state-of-the-art helium ion therapy

#### Thomas Tessonnier^1,2,3^, Stewart Mein^2,4,5,6^, Hoeltgen Line^1^, Held Thomas^1^, Eichkorn Tanja^1^, Harrabi Semi^1^, Debus Jürgen^1,5,6,7^, Haberer Thomas^7^, Mairani Andrea^2,4,8^

##### *^1^Heidelberg University Hospital, Department of Radiation Oncology, Heidelberg, Germany*

*^2^National Center for Tumor Diseases NCT, Division of Molecular and Translational Radiation Oncology, Heidelberg, Germany*

*^3^Heidelberg Ion Beam Center, Medical Physics - BioPT group, Heidelberg, Germany*

*^4^Heidelberg Ion Beam Center, BioPT group, Heidelberg, Germany*

*^5^German Cancer Research Center DKFZ, Heidelberg Institute of Radiation Oncology HIRO, Heidelberg, Germany*

*^6^German Cancer Research Center DKFZ, Clinical Cooperation Unit Radiation Oncology, Heidelberg, Germany*

*^7^Heidelberg Ion Beam Center, Ion Beam Therapy Center, Heidelberg, Germany*

*^8^National Centre of Oncological Hadrontherapy CNAO, Medical Physics, Pavia, Italy*

Following commissioning of the first clinically validated treatment planning system (TPS) for helium ion therapy, an evaluation of helium ions versus proton therapy in a 20 patient cohort for meningioma was performed. Assessment of the selected biological model (a modified microdosimetric kinetic model), corresponding inputs sensitivity (for (α/β)_x_=2Gy) and clinical impact of helium ion beams is performed in the context of bedrock clinical trial data from the Lawrence Berkeley National Laboratory (LBNL) and on-going treatment programs at the Heidelberg Ion-beam Therapy Center (HIT). Commissioning results confirm the reliability of the first clinical helium ion therapy TPS. Helium ion therapy exhibited statistically significant superior CTV coverage and reductions in low-dose bath in normal tissue compared to proton therapy. Studies indicate the selected biological model and input parameters are appropriate for clinical application and consistent with respect to known uncertainty of RBE prediction for ion therapy and against available in vivo data for radio-response. Mean RBE predictions in the target volume were ∼2.1 while ranging from 1.8 to ∼4 for clinically relevant dose level for normal tissues and OARs. RBE model predictions for the clinic were verified against in-vivo biological readouts and clinical outcome for OAR collected at LBNL. This work paves the way for further and upcoming trails and on-site studies using the established clinical procedure for treatment planning and delivery for raster-scanned helium ion beam therapy. The first helium treatment was performed at HIT in summer 2021.

### O 015 - Assessment of boron compounds on the biological effectiveness of proton therapy

#### Mandira Manandhar^1^, Scott Bright^1^, David Flint^1^, David Martinus^1^, Rishab Kolachina^1^, Mariam Ben Kacem^1^, Chad Lee^2^, Kendall Morrison^2^, Simona Shaitelman^1^, Gabriel Sawakuchi^1^

##### *^1^UT MD Anderson Cancer Center, Radiation Physics, Houston, USA*

*^2^TAE Life Sciences, TAE Life Sciences, Foothill Ranch, USA*

**Purpose:** To investigate whether alpha particles generated through proton-boron fusion reactions is sufficient to enhance the biological effectiveness of the proton beams by adding sodium borocaptate (BSH) or 4-borono-L-phenylalanine (BPA) to cells irradiated with protons.

**Methods:** We performed clonogenic survival assays in DU145 prostate cancer cells treated with 80.4 ppm BSH or 86.9 ppm BPA, or their respective vehicles, after irradiation with 6 MV x-rays, a low liner energy transfer (LET) (1.2 keV/μm) proton beam and a higher LET(9.9 keV/μm) proton beam. gH2AX and 53BP1 foci were measured at 1 h and 24 h post radiation in similar conditions.

**Results:** BSH radiosensitized DU145 cells to photons and protons. However, no difference was found in sensitization enhancement ratio (SER) or the relative biological effectiveness (RBE) for BSH-treated versus BSH-untreated cells irradiated with photons or both proton beams. No differences were found in gH2AX or 53BP1 foci or gH2AX/53BP1 colocalized foci for BSH-treated versus BSH-untreated cells across radiation types. BPA did not radiosensitize DU145 cells to either radiation types. Similarly, no difference in SER, RBE, gH2AX and 53BP1 foci or gH2AX/53BP1 colocalized foci in DU145 cells were observed in BPA-treated versus BPA-untreated DU145 cells.

**Conclusions**: Treatment with ^11^B, at concentrations of 80.4 ppm from BSH or 86.9 ppm from BPA, had no effect on the biological effectiveness of proton beams in DU145 cells. Our results agree with published theoretical calculations indicating that the contribution of alpha particles from boron-proton fusion reactions to the total absorbed dose and biological effectiveness is negligible.

### O 016 - Biosensor for Deconvolution of Individual Cell Fate in Response to Ion Beam Irradiation.

#### Martin Niklas^1^, Schlegel Julian^1^, Liew Hans^1^, Rein Katrin^1^, Abdollahi Amir^1^, Debus Juergen^2^

##### *^1^German Cancer Research Center, Molecular & Translational Radiation Oncology E210, Heidelberg, Germany*

*^2^University Hospital Heidelberg, Radiation Oncology, Heidelberg, Germany*

Clonogenic survival assay constitutes the gold standard method for quantifying radiobiological effects. However, it neglects cellular radiation response variability and heterogenous energy deposition by ion beams in ion beam cancer therapy on the microscopic scale. We introduce *Cell-Fit-HD^4D^*, a biosensor that enables a deconvolution of individual cell fate in response to the microscopic energy deposition as visualized by optical microscopy. *Cell-Fit-HD^4D^* enables single-cell dosimetry in clinically relevant complex radiation fields by correlating microscopic beam parameters with biological endpoints. Decrypting the ion beam's energy deposition and molecular effects at the single cell level has the potential to improve our understanding of radiobiological dose concepts as well as radiobiological study approaches in general.

### O 017 - Evaluation of biological scaffolds as a 3D tumour environment for studies on the impact of XRT and CIRT

#### Alexandra Charalampopoulou^1^, Amelia Barcellini^2^, Stefania Cesari^3^, Antonia Icaro Cornaglia^4^, Stefania Croce^5^, Silvia Molinelli^6^, Ester Orlandi^2^, Marco Pullia^6^, Alessandro Vanoli^7^, Angelica Facoetti^1^

##### *^1^National Center for Oncological Hadrontherapy CNAO, Radiobiology, Pavia, Italy*

*^2^National Center for Oncological Hadrontherapy CNAO, Clinical, Pavia, Italy*

*^3^Foundation I.R.C.C.S Polyclinic San Matteo, Molecular Medicine, Pavia, Italy*

*^4^University of Pavia, Public Health- Experimental and Forensic Medicine, Pavia, Italy*

*^5^Foundation I.R.C.C.S Polyclinic San Matteo, Surgical Sciences, Pavia, Italy*

*^6^National Center for Oncological Hadrontherapy CNAO, Medical Physics, Pavia, Italy*

*^7^Foundation I.R.C.C.S Polyclinic San Matteo, General Surgery, Pavia, Italy*

**Background**: 3D biologic scaffolds (Figure 1) promote cell viability, interactions and proliferation, recreating an environment suitable for the diffusion of oxygen, nutrients and cell growth factors. This work aims to: 1) assess the feasibility of bioscaffolds to reproduce the tumour environment for radiation effect studies; 2) investigate the response of radioresistant tumour cells cultured in this 3D model when exposed to XRT and CIRT.

**Materials and methods**: Mucosal melanoma HMV-II and pancreatic PANC-1 cells were seeded in bioscaffolds obtained by subunits of decellularized porcine liver and irradiated with 2 Gy and 4 Gy of photons and carbon ions. Every 7 days for 4 weeks, scaffolds were fixed, embedded in paraffin and subsequently sectioned; their histological sections were stained and evaluated (Figure 2).

**Results**: With regards to the 1^st^ aim: tumor cells exhibited infiltrative behaviour into the scaffolds maintaining different morphological features consistent with the cytotype without dedifferentiation. Concerning the 2^nd^ endpoint, HMV-II and PANC-1 irradiated cells showed a different response to LET: CIRT seemed to have a more severe effect on PANC-1 than HMV-II cells in terms of molecular alteration (binucleated cells with increased nuclei size, cytoplasm disaggregation, diapedesis). Melanin production of HMV-II cells was premature in the scaffolds treated with XRT compared to CIRT (14 days versus 21 days, respectively).

**Conclusions**: Bioscaffolds are a suitable radiobiological 3D study model. XRT and CIRT cause different morphological damages to PANC-1 and HMV-II cells in the bioscaffolds.

### O 018 - Proton therapy could offer a biologic advantage in the treatment of recurrent medulloblastoma

#### Tien Tang^1^, David Flint^2^, Scott Bright^2^, David Grosshans^3^, M. Waleed Gaber^1^

##### *^1^Baylor College of Medicine, Pediatrics, Houston, USA*

*^2^University of Texas M.D. Anderson Cancer Center, Radiation Physics, Houston, USA*

*^3^University of Texas M.D. Anderson Cancer Center, Radiation Oncology, Houston, USA*

Medulloblastoma is the most common pediatric brain tumor. Despite aggressive treatment, recurrent medulloblastoma remains a challenge. One possible approach to overcome these treatment resistant tumors is the use of protons, which potentially have differing patterns of biological damage. In this study, we aimed to determine if proton irradiation might be used to overcome photon-resistant medulloblastoma cell line survival. To recapitulate, a recurrent medulloblastoma cellular model, Daoy cells were irradiated with fractionated doses of 2Gy photon (120Kvp, 25mA, ∑50Gy). Clonogenic assays were used to verify photon radio-resistance and compare response between radiation modalities. Survival curves generated from the assay were used to calculate D_10 _(dose for 10% survival). To explore differential DNA damage, following 2Gy exposure, cells were immunostained with DNA double-strand break markers γ-H2AX and 53BP1. Results from the clonogenic assays, revealed the photon-resistant cell line had a significantly higher D_10, x-ray_ than wild-type, 7.14±0.08Gy and 4.99±0.03Gy respectively, when exposed to photon and thus confirmed the model. Proton therapy (9.9KeV/um) was found to significantly radiosensitize the photon-resistant cell line (D_10, proton_=4.42±0.25Gy, compared to photon radiation) but not wild-type (D_10, proton_=6.11±1.55Gy). As expected, there were significantly more DNA damage, more γ-H2AX foci, 2hrs after proton irradiation in both cell lines (compared to photon radiation). There were also significantly more 53BP1 foci in the photon-resistant cells compared to wild-type after proton irradiation which indicate potential differences in DNA damage response between radiation modalities. These findings seem to indicate that, in this model of photon-resistant medulloblastoma, treatment resistance may be overcome with proton irradiation.

### O 019 - Ultra-High Dose Rate Carbon Ion Irradiation: First in vivo Experiment

#### Walter Tinganelli^1^, Uli Weber^1^, Anggraeini Puspitasari^1^, Palma Simoniello^2^, Christoph Schuy^1^, Amir Abdollahi^3,4^, Claudia Fournier^1^, Helm Alexander^1^, Marco Durante^1,5^

##### *^1^GSI Helmholtzzentrum für Schwerionenforschung, Biopyhsics, Darmstadt, Germany*

*^2^University of Naples 'Parthenope', Dipartimento di Scienze e Tecnologie, Naples, Italy*

*^3^Heidelberg University Hospital, Heidelberg Ion-Beam Therapy Center HIT, Heidelberg-, Germany*

*^4^German Cancer Research Center DKFZ, Medical Physics, Heidelberg, Germany*

*^5^Technische Universität Darmstadt, Institut für Festkörperphysik, Heidelberg, Germany*

Radiotherapy indisputably plays a crucial role in cancer treatment since more than 50% of patients receive it throughout their treatment. However, the efficacy of radiation treatment has a limitation due to the toxicity in the normal healthy tissue. Short pulses of electrons or protons at ultra-high dose rates (FLASH) were shown to be less harmful to healthy tissue but just as efficient as conventional dose rate radiation for tumor growth inhibition. Radiotherapy under FLASH conditions may become a new strategy to treat cancer as it bears the potential to widen the therapeutic window of radiotherapy substantially. Our group published the first results on an in vitro FLASH experiment with carbon ions, demonstrating that FLASH irradiation with ions heavier than protons is feasible. The effects were shown to depend on the oxygenation level. However, in vitro experiments are certainly limited in their significance compared to complex in vivo experiments. Therefore, we recently performed the first in vivo experiment using ultra-high dose rate (> 40 Gy/s) carbon ions to expose a C3H/He osteosarcoma mouse model, which spontaneously develops lung metastases. Compared to conventional exposure to carbon ions, the ultra-high dose rate of carbon ion spared the healthy tissues, while providing a comparable tumor control (figure below). Furthermore, the results point to a reduced number of lung metastases in mice whose tumors were exposed under FLASH conditions compared to those treated with a conventional dose rate.

### O 020 - Effects of proton FLASH therapy in the immune response against rat glioblastoma

#### Lorea Iturri^1^, Annaïg Bertho^1^, Charlotte Lamirault^2^, Marjorie Juchaux^1^, Cristèle Gilbert^1^, Frédéric Pouzoulet^2^, Pierre Verrelle^3^, Ludovic de Marzi^4^, Yolanda Prezado^1^

##### *^1^Institut Curie, UMR 3347/U1021 – Signaling- Radiobiology and Cancer, Orsay, France*

*^2^Institut Curie, Translational Research Department- Experimental Radiotherapy Platform, Orsay, France*

*^3^Institut Curie, Centre de Protonthérapie d'Orsay- UMR9187 / U1196 - CMIB, Orsay, France*

*^4^Institut Curie, Centre de Protonthérapie d'Orsay- INSERM U1288 - LITO, Orsay, France*

FLASH radiation therapy [1] is increasingly gaining interest as it has the potential to reduce the toxicity associated to radiotherapy (RT), especially in the case of cancer treatments where the radiation is one of the key therapeutic elements. The mechanisms by which FLASH RT can control tumor growth are still under study, and several theories have been proposed, including a more efficient immune attraction to the tumor. To bridge this gap in knowledge, we evaluated the impact of proton FLASH therapy compared to conventional proton therapy given in one fraction in a rat glioblastoma model, with special attention to the immune response generated after the treatments and the neurological toxicities produced. FLASH effect was first demonstrated by means of behavioral tests: rats receiving FLASH therapy exhibited a significantly higher discrimination ratio than the ones receiving conventional irradiations. Using high-parameter flow cytometry, we characterized the immune cell heterogeneity of the tumor after the radiation, evaluating the density of different subsets of lymphocytes and myeloid cells in the tissue. We observed that both conventional proton therapy and proton FLASH RT are able to effectively mobilize a vast amount of lymphocytes to the tissue, altering the immune suppressive equilibrium that is a hallmark of glioblastoma. Studying the immune responses upon novel and less toxic types of radiotherapy will give more understanding of the radiobiological effects of these techniques. Importantly, it will give vital information that could be later used in future studies combining radio-immunotherapies.

### O 021 - Effect of FLASH versus Non-FLASH proton radiation on mice with head and neck cancer

#### Li Wang^1^, Ming Yang^2^, Qinglei Hang^1^, Xiaochun Wang^2^, Uwe Titt^2^, Yinhan Zhang^1^, Jennifer Heldring^1^, Narayan Sahoo^2^, Xiaorong Ronald Zhu^2^, Steven J. Frank^3^

##### *^1^The University of Texas MD Anderson Cancer Center, Experimental Radiation Oncology, Houston, USA*

*^2^The University of Texas MD Anderson Cancer Center, Radiation Physics, Houston, USA*

*^3^The University of Texas MD Anderson Cancer Center, Radiation Oncology, Houston, USA*

**Purpose:** FLASH-Radiotherapy (F-RT) has been identified as a promising modality to deliver curative dose to tumor instantaneously in milliseconds while mitigating normal tissue damage. We have shown that compared to conventional-proton radiotherapy (C-PRT), F-PRT caused reduced cell viability in human head and neck cancer (HNSCC) cells, and higher level of cell viability in the human normal tongue cells. However, effect of F-PRT versus C-PRT on mice bearing HNSCC are absent, these were investigated herein.

**Methods:** When SCC-VII (HNSCC) tumors (in the middle of tongue in C57/6J mice, 6/group) reached 18.1 mm (17.3−18.9 mm^3^) in average volume, a single dose of 20-Gy F-PRT (250-Gy/s) or C-PRT (0.592-Gy/s) were given to the oral cavity and surrounding normal structures of mice. The overall survival times (OSs, days from treatment initiation to mice death, were compared by a Kaplan-Meier analysis), body weight, and tumor growth of mice were compared.

**Results:** The mean OSs of mice with F-PRT versus C-PRT were 11.0±0.0 and 9.3±0.19 days, median OSs were 11.0 and 9.0 days; F-PRT (versus C-PRT) significantly prolonged OS (*P*=0.019) (Figure). No difference was observed in body weight and tumor growth changes between treatments. No mice death in control group during the observation period.

**Conclusion**: Mice with HNSCC tumors survived longer after F-PRT (20-Gy) versus C-PRT. No difference of tumor growth or body weight changes between F-PRT and C-PRT were observed. Studies in more tumor types and more radiation doses are warranted to validate this finding and to further provide rationales for clinical application.

### O 022 - Including volume effects in biological treatment plan optimization for carbon ion therapy: gEUD-driven optimization with TRiP98

#### Marco Battestini^1,2^, Marco Schwarz^3,4^, Michael Krämer^5^, Emanuele Scifoni^2^

##### *^1^University of Trento, Department of Physics, Trento, Italy*

*^2^National Institute for Nuclear Physics, Trento Institute for Fundamental Physics and Applications, Trento, Italy*

*^3^Azienda Provinciale per i Servizi Sanitari, Trento Proton Therapy Center, Trento, Italy*

*^4^University of Washington, Radiation Oncology Department, Seattle, USA*

*^5^GSI Helmholtz Center for Heavy Ions Research, Biophysics Department, Darmstadt, Germany*

TRiP98, a research software for particles treatment planning, pioneered the implementation of RBE-weighted dose optimization. However, it allowed only for voxel-based dose objectives, i.e. prescribed dose to the target and maximum dose to healthy tissues. We present a method to account for volume effects in plan optimization, using the Generalized Equivalent Uniform Dose (gEUD), which allows to convert a heterogeneous dose distribution into a uniform dose associated with the same biological effect and closely correlates to NTCP. We implemented the gEUD-based optimization in TRiP98 for carbon ion therapy, allowing to control the whole DVH shape of OAR with a single objective, reducing the volume receiving dose levels close to the mean or the maximum dose, depending on the organ type specific volume effect parameter *a* and a prescribed gEUD_0_. We studied the role of such parameters in multi-field optimization. We compared voxel-based and gEUD-based optimization in chordoma cases with different anatomies. For all plans containing multiple OARs, we obtained the same target coverage and similar DVHs for OARs with a small volume effect, while decreasing the mean dose received by the parotids, thus reducing their NTCP, as in the example shown in Figures 1–2, where NTCP decreased from 11.09% to 4.37%. This novel optimization method (Battestini et al. Front. Oncol. accepted) can be applied to different OARs, achieving the largest improvement if volume effect is large. This allows TRiP98 to perform a double level of biologically-driven optimization for ion beams, including both RBE-weighted dose and volume effects.

### O 023 - Radiobiological models in Carbon Ion Radiotherapy (CIRT): How do physical and treatment planning parameters influence the relation between clinical RBE-models?

#### Joanna Gora^1^, Sarah Grosshagauer^2^, Mansure Schafasand^1,3^, Antonio Carlino^1^, Markus Stock^1^, Piero Fossati^1^

##### *^1^MedAustron Ion Therapy Centre- Austria, Medical Department, Wiener Neustadt, Austria*

*^2^Technical University of Vienna- Austria, Atomic Institute, Vienna, Austria*

*^3^Medical University of Vienna- Austria, Radiotherapy Department, Vienna, Austria*

There are two main clinically used radiobiological models, calculating RBE-weighted dose for CIRT (MKM and LEM). Although both estimate RBE-distributions within tissue, translation between them is not straightforward. Several dose prescription and OARs constraints translations are already clinically applied. However, they carry limitations, which may potentially influence the treatment outcome. The study aims at visualizing the theoretical and practical aspects, which affect the relation between the models. It was divided into 2 sections: model dependencies (LET and dose/fx dependencies) and TP considerations (beam arrangement, dose gradients, spot placing: blocking vs constraints, range shifter contribution). Evaluations were performed in the virtual phantom and patient CT, optimizing the LEM RBE-weighted dose first, followed by MKM recomputation, averaged LET distributions were examined in addition (RayStationV9). Results illustrate how mentioned parameters influence LEM/MKM relationship, therefore any translations between them require great caution. Figure1 exemplifies one of the findings. For plateau and tail region (dose/fx independently), MKM-doses are as expected lower comparing to LEM(16-40%). SOBP region highly depends on dose/fx(-29%to+40%). However, distal target/OAR overlapping region calculation is driven by created dose gradients. MKM-dose for high-gradients is lower comparing to LEM-doses(by 17%-34%), while for low-gradients the behavior is reversed, resulting with higher MKM-doses(up to 33%). Understanding the relation between the models, allows combining European and Japanese-based experience and therefore more confident decisions during the TP process. Not every center has capability of simultaneous LEM and MKM calculations. This study guides and highlights the importance of choosing optimal planning strategies, especially when OAR constraint dose escalation is considered.

### O 024 - Exploring LET and RBE effects from pruning techniques in proton arc therapy

#### Helge Henjum^1^, Johannes Tjelta^1,2^, Lars Fredrik Fjæra^1^, Sara Pilskog^1,2^, Camilla H. Stokkevåg^1,2^, Kristian S. Ytre-Hauge^1^

##### *^1^University of Bergen, Department of Physics and Technology, Bergen, Norway*

*^2^Haukeland University Hospital, Department of Oncology and Medical Physics, Bergen, Norway*

**Purpose/objective:** Proton arc therapy (PAT) is an emerging treatment modality that holds promise to improve target coverage and reduce linear energy transfer (LET) in organs at risk. We aimed to investigate if pruning the highest energy layers in each beam direction could further reduce high LET values surrounding the target, and thus also the relative biological effectiveness (RBE)-weighted dose.

**Method and Materials:** A 240° PAT plan for a germinoma patient was created with multifield optimization in the Eclipse TPS with a prescribed dose of 54 Gy(RBE_1.1_). The PAT plan was iteratively pruned for energy layers in each direction, creating 6 PAT plans with different amounts of pruning (P-PAT) while maintaining tumor coverage. All plans were recalculated in the FLUKA Monte Carlo software, and the LET, physical dose, and variable RBE-weighted dose from the Rørvik model (ROR) and an LET weighted dose model (LWD) were evaluated.

**Results:** Almost all P-PAT plans reduced the mean RBE-weighted dose to the surrounding healthy tissue compared to the PAT plan (Figure 1), whereas the 6-layers P-PAT plan showed an increase. These findings were consistent across RBE models. The LET was increasingly higher within the PTV for each pruning iteration, where the mean LET from the 6-layer P-PAT plan was 1.5 higher than for the PAT plan. Likewise, the LET values to the healthy tissue were reduced for each pruning iteration (Figure 2).

**Conclusion:** We demonstrated a pruning method for PAT to increase LET and RBE in the target and reduce it in surrounding tissue while maintaining sufficient target coverage.

### O 025 - Reducing high LET component of dose to the brainstem in proton treatment planning for brain tumors by optimization

#### Anne Vestergaard^1^, Morten Høyer^1^, Jesper Kallehauge^1^, Ole Nørrevang^1^, Bob Smulders^1,2^, Yasmin Lassen-Ramshad^1^, Aida Muhic^1,2^, Rikke Dahlrot^1,3^, Erik Traneus^4^, Korreman Stine^1^

##### *^1^Aarhus University Hospital, Danish Centre for Particle Therapy, Aarhus N, Denmark*

*^2^University Hospital of Copenhagen / Rigshospitalet, Department of Oncology, Copenhagen, Denmark*

*^3^Odense University Hospital, Department of Oncology, Odense, Denmark*

*^4^RaySearch Laboratories, Ab, Stockholm, Sweden*

Clinical evidence of increased RBE at the distal edge of proton beams is emerging. This study explores the use of optimization to reduce the high LET dose contribution to the brainstem, by re-optimizing plans following clinical guidelines as well as more brain sparing field arrangements. Five grade 1 meningioma patients were treated (prescribed dose 54 GyRBE) according to treatment planning guidelines, that do not allow more than one of minimum 3 beams with distal edge in the brainstem. Treatment plans were optimized in a research version of Raystation (10B-IonPG, Raysearch Laboratories) using same beam angles as clinically treated plans (Clin). A plan was created to spare normal brain (DE). Both plans were re-optimized with a penalty on the dose component in the brainstem with LET>3 keV/μm. The high LET dose component (DhighLET) and the variable RBE (McNamara model, brainstem α/β=2) were calculated. For all plans with the high LET dose objective, brainstem D2cc decreased compared to the Clin and DE plans. DhighLET in the brainstem was low for Clin plans. The DE plans reduced mean dose to the normal brain by 22% and had a higher DhighLET compared to Clin plans. When DE plans were optimized with the high LET dose objective, DhighLET decreased to the same level as the Clin plan. For the clinical beam configurations, the high-LET dose component was generally low. Using the DE beam configurations decreased the mean dose to the normal brain and by optimizing with penalty on the high LET dose component the brainstem D2cc decreased.

### O 026 - Integrating the maximization of peak-to-valley dose ratio and normal-tissue survival fraction into treatment planning for proton minibeam radiation therapy

#### Weijie Zhang^1^, Wangyao Li^1^, Yuting Lin^1^, Fen Wang^1^, Ronald C Chen^1^, Hao Gao^1^

##### ^1^University of Kansas Medical Center, Radiation Oncology, Kansas City, USA

**Purpose:** Proton minibeam radiation therapy (pMBRT) is a novel proton modality of spatially fractionated RT (SFRT). pMBRT can reduce the radiation damage to normal tissues via biological dose sparing of high peak-to-valley dose ratio (PVDR). This work will integrate the maximization of PVDR into pMBRT treatment planning. This new optimization method can be combined with the hardware approach (e.g., using collimators) for maximizing PVDR.

**Methods:** The new optimization method simultaneously maximizes the normal-tissue PVDR and optimizes the dose distribution at tumor targets and organs-at-risk (OAR). The PVDR maximization is through the joint total variation (TV) and L1 regularization with respect to the normal-tissue dose. That is, at beam-eye-view projected dose slices of several depths for each beam angle, the TV of dose is maximized, corresponding to the PVDR maximization, while the L1 of dose is minimized, corresponding to the minimization of the OAR dose and maximization of survival fraction (SF).

**Results:** The new IMPT method with TV and L1 regularization (TVL1) was validated in comparison with the conventional IMPT method (CONV) for pMBRT on several clinical cases. The results show that TVL1 provided larger PVDR and SF than CONV for biological sparing of normal tissues, with preserved plan quality in terms of physical dose distribution.

### O 027 - Integrating the production of radicals from water radiolysis into the framework of microdosimetry

#### Alejandro Bertolet^1,2^, Harald Paganetti^1,2^, Jan Schuemann^1,2^

##### *^1^Massachusetts General Hospital, Radiation Oncology, Boston, USA*

*^2^Harvard Medical School, Radiation Oncology, Boston, USA*

Microdosimetry captures the local energy deposition patterns from different ionizing radiation qualities. The local distribution of energy depositions impact the complexity of damages induced to radiobiologically relevant targets such as the DNA. In a previous work [1], we showed that the energy imparted into a microscopic site correlates with the number of strand breaks (SBs) induced by direct effect, i.e., the SBs originated by direct ionization from radiation in the very early stage of radiation action (up to 1 fs). However, part of the damage induced to DNA is mediated by radicals resulting from radiolysis of molecules of water in the cellular cytosol. Microdosimetry only accounts for physical quantities such as energy imparted, so that it cannot predict the magnitude of the indirect effect, i.e., the SBs produced by these radicals at a later stage. In this work, we used the Monte Carlo multi-scale tool TOPAS-nBio to study the yield of ·OH radicals at 1 μs induced by protons with different energies in a 1 μm-diameter site of liquid water [2]. The yield of ·OH radicals was related to the dose-average linear energy () as shown in the figure below including a saturation term to account for higher radical recombination at higher local energy depositions. Our work is a first step towards linking common microdosimetric quantities to fundamental radiation damage by including time dependent effects of radiochemistry.

### O 028 - A Multiscale Generalized Stochastic Microdosimetric Model (GSM2) to describe dynamical oxygenation and fast reaction kinetics for unraveling the FLASH effect

#### Francesco Giuseppe Cordoni^1^, Marta Missiaggia^2^, Giorgio Cartechini^2^, Martina Fuss^3^, Gianmarco Camazzola^3^, Daria Boscolo^3^, Marco Battestini^2^, Andrea Attili^4^, Francesco Tommasino^2^, Michael Krämer^3^, Chiara La Tessa^2^, Emanuele Scifoni^5^

##### *^1^University of Trento, Department of Civil- Environmental and Mechanical Engineering, Trento, Italy*

*^2^University of Trento, Dep. of Physics, Trento, Italy*

*^3^GSI Helmholzzentrum für Schwerionenforschung, Biophysics division, Darmstadt, Germany*

*^4^INFN-Roma 3, Dep. of Physics, Roma, Italy*

*^5^TIFPA-INFN, Dep. of Physics, Trento, Italy*

FLASH radiotherapy has emerged in recent years as a promising new avenue for therapeutic use of radiation. It has been collected experimental evidence that ultra high dose rate (FLASH) irradiations are able to spare normal tissue being equally efficient for tumour control. Besides this evidence, the mechanism at the basis of FLASH effect remains largely unexplained to date. The Generalized Stochastic Microdosimetric Model (GSM2), [1,2], is a probabilistic model that describes the time-evolution of the DNA damages in a cell nucleus from microdosimetric principles. Among the most relevant strengths there is the capability of efficiently treating the several levels of spatio-temporal stochasticity happening during a protracted irradiation. We derive a multiscale GSM2 coupling the DNA damage evolution with fast reaction kinetics accounting for radical formations, oxygen consumption and re-oxygenation happening during a given dose delivery time structure. The resulting multiscale GSM2 describes the coupled evolution of the multiscale system composed by DNA damage and fast Reactive Oxygen Species (ROS). Via simulations of proton energy deposition in a microscopic volume using TRAXCHEM, we study the combined effects of ROS and the time evolution of DNA damages, assessing the resulting cell-survival curve at different beam structures and oxygenation conditions. We show how the multiscale GSM2 is able to justify the empirical patterns of Double Strand Breaks (DSB) yields and normal tissue sparing observed in FLASH radiotherapy, [3], see Figure 1.

### O 029 - A Monte Carlo study of the free radical production in minibeam radiation therapy

#### Thongchai Masilela^1,2^, Yolanda Prezado^1,2^

##### *^1^Institut Curie - Université PSL - CNRS UMR3347 - Inserm U1021, Signalisation radiobiologie et cancer, 91400 - Orsay, France*

*^2^Université Paris-Saclay - CNRS UMR3347 - Inserm U1021, Signalisation radiobiologie et cancer, 91400 - Orsay, France*

Minibeam radiation therapy is a novel treatment technique characterised by the use of narrow beamlets to create distributions of high (peak) and low (valley) doses. By calculating the primary yield of reactive oxygen species (ROS), our goal was to assess whether the ROS composition differed between these regions, thereby bridging the gap of knowledge between the physical and biological implications of this technique. Simulations were performed using TOPAS-nBio. A water phantom was irradiated with protons and x-rays in broad beam (BB) and minibeam (MB) configurations. The primary yield at 1000 ps of the hydroxyl radical (an indicator of indirect DNA damage) was calculated in spherical cellular targets. While we observed no inherent difference between the types of ROS produced in the peaks and valleys, the absolute amount depended on the radiation's spatial distribution. At all depths, the hydroxyl yield for x-rays was approximately 1.6 times larger in the MB peak and 18 times smaller in the valley compared to the BB configuration. For protons these ratios in the entrance region were 5.6 and 60 respectively, but approached unity towards the Bragg peak. In a target located at the Bragg peak, the peak-to-valley ROS ratios suggest a differential indirect DNA damage effect for x-rays, whereas for protons this damage is more homogenous. At this instant in time, the distribution of free radicals appears to be analogous to the macroscopic dose. Further simulations are being performed to calculate direct and indirect strand breaks, and investigate the effect of diffusion at larger time scales.

### O 030 - Accumulation of sublethal radiation damage and its impact on cell survival

#### Oleg Vassiliev^1^, Christine Peterson^2^, David Grosshans^3^, Radhe Mohan^1^

##### *^1^The University of Texas MD Anderson Cancer Center, Department of Radiation Physics, Houston, USA*

*^2^The University of Texas MD Anderson Cancer Center, Department of Biostatistics, Houston, USA*

*^3^The University of Texas MD Anderson Cancer Center, Department of Radiation Oncology, Houston, USA*

**Objective:** Determine the extent of sublethal radiation damage (SRD) in cells surviving a given dose of radiation and how it affects cell survival curves.

**Material and methods:** We have developed a cell survival model that accounts for accumulation of SRD. The model uses a microdosimetry based formalism to calculate characteristics of energy deposition events on a microscopic scale. Each event may cause lethal or sublethal damage. Probabilities of the two outcomes depend on the statistics of all preceding events. We have validated the model and evaluated its parameters using data from 341 measured cell survival curves for photons, protons and heavy ions.

**Results:** Accumulation of SRD begins at doses below 1 Gy and includes increasing the number of damaged cells and damage buildup in individual cells. At 10 Gy few cells remain undamaged, whereas the subpopulation of most damaged cells becomes the largest. Accumulation of SRD in a cell reduces its repair ability - the most damaged cells are most radiosensitive. This results in the slope of a survival curve becoming constant at high doses - a property supported by experimental data. Energy deposition events occur in cells at random. This makes cell population highly heterogeneous in terms of radiosensitivity.

**Conclusions:** Accumulation of SRD is an important factor in cell response to radiation. Starting at doses below 1 Gy this process makes a population of surviving cells highly heterogeneous in terms of radiosensitivity. This warrants development of novel cell survival and tumor control models that account for this heterogeneity.

### O 031 - Patient variation effect on tumor control probability of lung PBS treatment

#### Weijun Xiong^1^, Minglei Kang^1^, Haibo Lin^1^, Chengyu Shi^1^, J. Isabelle Choi^2^, Charles B Simone II^2^

##### *^1^New York Proton Center, Medical Physics, New York, USA*

*^2^New York Proton Center, Radiation Oncology, New York, USA*

**Background**: This study is to examine the effect of patient variation of α and β on tumor control probability (TCP) for hypofractionated and conventionally-fractionated lung PBS treatment. The goal of this study is to develop a patient-specific TCP model for future lung PBS treatment.

**Method and Materials**: The lung cancer TCP based on the linear-quadratic (LQ) model was calculated using parameters α and β. The LQ parameters were derived from clinical data for lung cancer, and variations in α and β were added through parameters σα, σβ which are the standard deviation of Gaussian distribution around α0 and β0, respectively.

**Results and Conclusion**: The patient variation σα is always necessary to be considered for the treatment. It requires an extra dose for hypofractionated treatment and a low α/β ratio to achieve a greater-than-50%TCP when σα is increasing. For low α/β ratio, the β term also plays important role in cell-killing, and the patient variation σβ is also necessary to be considered for hypofractionated treatment. Even for high α/β ratio, when moving from conventionally-fractioned to hypofractionated treatment, the variation σβ also has a moderate effect on treatment. From the analysis of the extra dose to 95% TCP with variation σβ, TCP for hypofractionated treatment is more prone to patient variation σβ.

### O 032 - Dosimetric comparison of breath-hold and free-breathing techniques in left-sided breast cancer patients treated with proton pencil beam scanning

#### Emile Gogineni^1^, Zachary Fellows^2^, William Hrinivich^1^, Heng Li^1^, Victoria Croog^1^, Jean Wright^1^, Khadija Sheikh^1^

##### *^1^Johns Hopkins University School of Medicine, Radiation Oncology and Molecular Radiation Sciences, Baltimore, USA*

*^2^Miami Cancer Institute, Radiation Oncology, Miami, USA*

**Purpose*****:*** To evaluate dosimetric differences in lung and heart doses in left-sided breast cancer patients treated with intensity-modulated proton therapy (IMPT) using Active Breathing Control (ABC) and free-breathing (FB).

**Methods*****:*** Eight left-sided breast cancer patients undergoing IMPT were planned on both FB and ABC CT simulation scans and were robustly optimized with range and setup uncertainty of 3.5% and 5 mm. A representative comparison plan is provided in Figure 1. The prescription for all patients was 50 Gy radiobiological equivalent (GyE) in 25 fractions. Dosimetric parameters, lung density, and time on table for beam were compared using paired t-tests.

**Results*****:*** A dosimetric comparison is provided in Table 1. Volume of ipsilateral and contralateral lungs receiving 5 GyE and volume of ipsilateral lung receiving 20 GyE were all significantly higher with ABC than with FB. Maximum heart dose was lower with ABC than FB, but there were no statistically significant differences in any other heart or left anterior descending artery volumetric endpoint between ABC and FB. Lung density was significantly lower with ABC than FB. Time on table for beam was significantly longer with ABC than with FB.

**Conclusions*****:*** ABC plans provided slightly higher lung dose and mostly similar cardiac dose in comparison with FB plans. Given the large increase in the time required for ABC treatment without obvious dosimetric or clinical benefit, FB techniques may be preferred when treating breast cancer with IMPT in order to maximize clinic workflow and minimize patient inconvenience, particularly in patients without cardiac risk factors.

### O 033 - Advanced Pencil Beam Scanning Bragg-peak FLASH-RT can enhance lung cancer planning treatment outcomes compared to conventional PBS techniques

#### Shouyi Wei^1^, Haibo Lin^1^, J. Isabelle Choi^2^, Charles B. Simone II^2^, Minglei Kang^1^

##### *^1^New York Proton Center, Medical Physics, New York, USA*

*^2^New York Proton Center, Radiation Oncology, New York, USA*

**Purpose**: To compare dosimetric characteristics between an advanced proton pencil beam scanning (PBS) Bragg-peak FLASH technique and conventional PBS planning technique in lung tumors.

**Method**: Single-energy PBS Bragg-peak FLASH treatment plans(a cohort of 10 patients) were optimized based on a novel distal tracking technique to enable Bragg-peaks to stop at the distal edge of the target. Inverse treatment planning using multiple-field optimization(MFO) can achieve sufficient FLASH dose rate and intensity-modulated proton therapy(IMPT)-equivalent dosimetric quality. The dose rate of organs-at-risk(OARs) and the target were calculated under realistic FLASH machine parameters. FLASH dose rate coverage(V40Gy/s) was also evaluated as a metric of the FLASH sparing effect. The standard care of PBS treatment technique using multiple energy layers was rescaled to 34Gy/fraction as reference. The dosimetric quality was compared between Bragg-peak FLASH and conventional plans based on RTOG0915 dose metrics.

**Results**: The Bragg-peak plans achieve >70% of V40Gy/s FLASH coverage for all major OARs. The lower dose rate region was associated with a low dose, occurring at the PBS field penumbra region. The PBS treatment plans yield slightly superior target dose uniformity with a D2%(%) of 108.02% versus Bragg-peak at 115.95%(*p*=0.001). No significant difference was seen between dose metrics for Bragg-peak and IMPT treatment plans for the spinal cord, esophagus, heart, or lung-GTV(all *p*=0.1).

**Conclusion**: Bragg-peak plans can achieve comparable plan quality to conventional PBS while achieving sufficient FLASH dose rate coverage for major OARs. The novel Bragg-peak FLASH technique has the potential to enhance lung cancer planning treatment outcomes compared to standard PBS treatment techniques.

### O 034 - Long-term results of pencil-beam scanning proton therapy in the Mayo 3-fraction partial breast irradiation nonrandomized phase 2 trial

#### Robert Mutter^1^, Michael Golafshar^2^, Todd DeWees^2^, Kimberly Corbin^1^, Nicholas Remmes^1^, Lisa McGee^3^, Carlos Vargas^1^, Dean Shumway^1^, Tina Hieken^4^, Sean Park^1^

##### *^1^Mayo Clinic, Department of Radiation Oncology, Rochester, USA*

*^2^Mayo Clinic, Department of Biomedical Informatics, Scottsdale, USA*

*^3^Mayo Clinic, Department of Radiation Oncology, Scottsdale, USA*

*^4^Mayo Clinic, Department of Surgery, Rochester, USA*

**Purpose:** We conducted a trial of 3-fraction photon, proton, or brachytherapy PBI, with technique selected at physician and patient discretion. Herein, we report long-term outcomes of the novel proton regimen.

**Materials/Methods:** Eligible women were age ≥ 50 years and s/p lumpectomy with negative sentinel nodes for estrogen receptor positive (ER+) invasive breast cancer (BC) without preoperative chemotherapy or any DCIS measuring ≤ 2.5 cm. CTV was post-operative bed plus 1 cm with ± 3 mm/3% robustness checks and prescribed 21.9 Gy (RBE) in 3 fractions administered consecutive business days. The regimen was designed to have comparable biologically effective dose as 40 Gy in 15 fractions, assuming an α/β ratio of 3.5.

**Results:** Between 2015-2017 49 proton patients were treated with proton PBI. Median patient age was 67 years. 82% had invasive BC and 18% had DCIS. The median of the mean heart/ipsilateral lung and breast V50% doses were <0.001/0.1 Gy and 27%.The proportion of patients with adverse cosmesis (by RN assessment) was 16% pre-PBI and 4% at last follow-up (median 4.2 years) with no patients experiencing cosmetic worsening and 96% having good/excellent cosmesis. Two patients developed local recurrence (1 marginal, 1 in-field). 4-year local recurrence-free, progression-free, and overall survival were 97.9%, 93.7%, and 93.7%, respectively. There were no treatment related grade ≥2 adverse events and no evidence of deterioration in patient-reported pain, fatigue, breast related or overall quality of life.

**Conclusions:** 3-fraction proton PBI exquisitely spared normal tissues, was associated with favorable cosmetic outcomes, disease control, and excellent tolerability.

### O 035 - Dose rate and dose robustness for proton transmission FLASH-RT in lung cancer treatment

#### Shouyi Wei^1^, Haibo Lin^1^, Chengyu Shi^1^, Weijun Xiong^1^, Chin-Cheng Chen^1^, Sheng Huang^1^, Robert H. Press^2^, Shaakir Hasan^2^, Arpit M. Chhabra^2^, J. Isabelle Choi^2^, Charles B. Simone^2^, Minglei Kang^1^

##### *^1^New York Proton Center, Medical Physics, New York, USA*

*^2^New York Proton Center, Radiation Oncology, New York, USA*

**Purpose**: To evaluate the plan quality and robustness of both dose and dose rate of proton pencil beam scanning (PBS) transmission FLASH delivery in lung cancer treatment.

**Methods**: An in-house FLASH TPS was used to optimize 10 lung cancer patients previously treated with proton SBRT to receive 3 and 5 transmission beams (Trx-5fds and Trx-3fds) to 34 Gy in a single-fraction. Perturbation scenarios (n=12) for setup and range uncertainties (5mm and 3.5%) were introduced and dose-volume histogram and dose-rate-volume histogram bands were generated. Conventional proton SBRT plans were used as reference. RTOG0915 dose metrics and 40Gy/s dose rate coverage (V40Gy/s) were used to assess the dose and dose rate robustness.

**Results**: Trx-5fds yields a comparable ICTV D2% of 35.9 Gy, whereas Trx-3fds results in inferior D2% of 38.5 Gy to the clinical SBRT plans with D2% of 35.8 Gy (p<0.05). Both Trx-5fds and Trx-3fds plans had slightly worse OAR dose metrics than SBRT plans. Trx-5fds achieved superior dosimetry robustness for ICTV, esophagus, and spinal cord dose than both Trx-3fds and conventional SBRT plans. There was no statistical difference in dose rate robustness for V40Gy/s coverage between Trx-3fds and Trx-5fds.

**Conclusion**: Transmission plans yield overall inferior plan quality compared to the conventional proton SBRT plans but provide improved robustness. With the use of more beams, both dose and dose rate robustness for the transmission plans can be achieved.

### O 036 - Accuracy of residual interfraction patient positioning in Ocular Proton Therapy (OPT)

#### Martijn Hol^1^, Myra Rodrigues^2^, Yvonne Klaver^2^, Kees Spruijt^2^, Jasper Kouwenberg^2^, Eleftheria Astreinidou^1^, Coen Rasch^1^

##### *^1^Leiden University Medical Center, Radiotherapy, Leiden, Netherlands*

*^2^Holland PTC, Radiotherapy, Delft, Netherlands*

**Introduction**: In ocular proton therapy patients are positioned upright on a robotic chair, the head is fixated with a bite frame and mask. Patients are requested to gaze towards a LED-light to fixate the affected eye. To facilitate pre-treatment verification, tantalum fiducials are attached to the eye circumventing the target volume. Before each fraction two orthogonal x-ray images are manually registered to a reference DRR, based on the fiducial position only.

**Objective**: We investigated the interfraction positioning accuracy of the current practice at HollandPTC.

**Methods**: A cohort of 60 patients was selected, each patient received 60GyRBE in 4 fractions. From each patient the reference DRR (generated by EOPP treatment planning system) and last pre-treatment orthogonal kV images were used to perform an automated offline rigid body registration determining the interfraction positioning accuracy. In total 240 fractions were analyzed, the means and standard deviations are calculated.

**Results**: The interfraction statistics of the patient population are reported. The deviations in the longitudinal direction are larger than other directions. This can be explained by use of the strap and robustness in the longitudinal direction in the treatment plan, which allows some freedom in patient positioning. Based on the statistics, we calculated the margins according to the simplified Herk margin recipe. The calculated margins are 0.7, 1.5 and 0.8 mm for the lateral, longitudinal and vertical direction.

**Conclusions**: A day-to-day patient positioning accuracy is determined for the current practice of ocular proton patients at HollandPTC. The determined margins are within the current clinical safety margin of 2.5 mm.

### O 037 - Comparison of two PBS proton CSI Treatment Techniques: single posterior-anterior beam versus two posterior oblique beams for the brain target

#### Lei Hu^1^, Anna Zhai^1^, Qing Chen^1^, Chin-Cheng Chen^1^, Francis Yu^1^, Jana L. Fox^2^, Suzanne L. Wolden^3^, Jonathan Yang^3^, Charles B. Simone II^4^, Haibo Lin^1^

##### *^1^New York Proton Center, Department of Physics, New York, USA*

*^2^Montefiore Medical Center, Radiation Oncology, New York, USA*

*^3^Memorial Sloan Kettering Cancer Center, Radiation Oncology, New York, USA*

*^4^New York Proton Center, Radiation Oncology, New York, USA*

**Background**: To compare two approaches for pencil beam scanning (PBS) CSI, Bi-lateral Posterior Oblique Beams (BPO) versus Single Posterior-Anterior Beam (SPA), for the brain target in terms of dosimetric differences and treatment efficiency.

**Materials and Methods**: Five patients were simulated in the head-first supine position on a long BOS frame and scanned using 3 mm CT slice thickness. A customized thermoplastic mask immobilized the patient's head and neck and a vac-lock for legs. The plans were robustly optimized with 3mm setup and 3.5% range uncertainties, plus 5 mm uncertainty to ensure a smooth dose match line in the 5 cm segment junctions. The brain field(s) included either BPO beams sharing the same isocenter or a SPA beam. The spine segments were planned with a single PA field. Dosimetric differences between the BPO and SPA were compared, and the treatment efficiency was analyzed according to timestamps of beam delivery as well as simulations.

**Results**: The Dmax were 111.4±2.1% vs 109.4±1.9% and the inhomogeneity (D5/D95) were 1.030±0.010 vs 1.027±0.003 in brain CTVs using SPA and BPO beam arrangements, respectively. Lens received less dose by 3.05±2.2 Gy(RBE) (left) and 2.52±1.9 Gy(RBE) (right) in the SPA plans. No significant cochlea dose change was observed. SPA may reduce the treatment time by more than 4 minutes in average and up to 10 minutes considering the beam line allocation.

**Conclusion**: SPA is dosimetrically comparable to BPO, with reduced lens doses at the cost of slightly higher dose inhomogeneity and hot spots. Implementation of SPA may improve treatment efficiency of PBS CSI.

### O 038 - Protontherapy/tomotherapy for curative dose escalation of sinonasal skull base adenoid cystic carcinoma: return on experience and literature overview

#### Antoine Mavrikios^1^, Farid Goudjil^1^, Sofia Zefkili^1^, Valentin Calugaru^1^

##### ^1^Institut Curie, Department of Radiation Therapy, Paris, France

Adenoid cystic carcinomas (ACC) represent 1% of head and neck malignancies. Sinonasal cases account for 10-25% of all cases with high propensity for skull base inflitration. Curative dose escalation for this poor prognosis subgroup of radioresistant tumors is challenging because of adjacent organs at risk. Highly conformational techniques like proton therapy and helical tomotherapy appear to be viable options because of their unique radiobiological and balistic properties but reports remain scarce. Eighteen patients treated by proton therapy and/or helical tomotherapy for sinonasal skull base ACC at Institut Curie, France, between March 2010 and August 2020 were retrospectively included. 61% of the patients did undergo surgery with positive margins prior to radiotherapy. Median delivered dose was 73.8 Gy. 33% of the patients received concomitant cisplatin chemotherapy. Evaluated outcomes were overall survival (OS), local control (LC), distant metastasis-free survival (DMFS), disease-free survival (DFS), acute and late toxicity profile.Median follow-up was at 49.6 months. 5-year OS, LC, DMFS, DFS rates were 46%, 54%, 46% and 39% respectively. 2 patients had a grade 3 acute toxicity. 1 patient had a grade 4 late optic nerve disorder. 45% of the patients had a grade 3 late toxicity of which 7 (39%) had a grade 3 late hearing impairment. Proton therapy and helical tomotherapy are effective and safe methods of dose escalation for curative treatment of sinonasal skull base ACC. Our results are coherent with available literature. Available data also suggests that carbon-ion therapy may be even more efficient because of better radiobiological properties.

### O 039 - Early clinical outcomes of spot-scanning proton beam therapy with simultaneous integrated boost technique for IDH-mutated glioma

#### Maciej Pelak^1^, Carola Lütgendorf-Caucig^1^, Petra Georg^1^, Birgit Flechl^1^, Ulrike Mock^1^, Piero Fossati^1^, Marta Mumot^1^, Antonio Carlino^1^, Markus Stock^1^, Eugen Hug^1^

##### ^1^MedAustron, Ion Therapy Center, Wiener Neustadt, Austria

**Introduction**: Gliomas with isocitrate dehydrogenase (IDH) mutation have a favorable prognosis compared to IDH-wildtype tumors. This justifies the use of proton beam therapy (PBT) as it reduces unnecessary dose to unaffected brain and, consequently, late side effects. The use of simultaneous integrated boost (SIB) technique simplifies the planning process and contributes to a more conformal dose distribution. We analyzed the early outcomes of SIB PBT in patients with IDH-mutated glioma.

**Methods**: The analysis included patients enrolled in the MedAustron prospective registry study, who were diagnosed with glioma with confirmed IDH1/2 mutation. Prescription for WHO grade II (15 patients) was 54.04 Gy RBE for PTV2 at 1.93 Gy/frct and 50.4 Gy RBE at 1.8 Gy/frct for PTV1 in 28 fractions. For WHO grade III tumors (11 patients) the dose was 60 Gy RBE for PTV2 at 2.0 Gy/frct and 54 Gy RBE for PTV1 at 1.8 Gy/frct in 30 fractions. Follow-up including MRI was obtained at 3, 6 and 12 months and then annually. Toxicity was assessed using CTCAE v4.0.

**Results**: Between October 2017 and April 2021 26 adult patients were treated. Median follow-up was 29.1 months. Overall survival (OS) and progression-free survival (PFS) were 96.2% and 88.5%, respectively. Actuarial 2-year OS was 100% and PFS was 95.8%. No acute or late toxicity > grade 2 was observed. Radiation-induced brain lesions were identified in 9 patients, 6/9 lesions resolved spontaneously without treatment.

**Conclusion**: Early clinical data demonstrate that SIB PBT is a safe and effective treatment of IDH-mutated glioma.

### O 040 - Novel unconventional immunomodulatory approach for partial tumor irradiation using protons and carbon ions exploiting the bystander and abscopal effects

#### Slavisa Tubin^1^, Piero Fossati^1^, Razvan Galalae^1^, Rastko Konstantinovic^1^, Antonio Carlino^1^, Markus Stock^1^, Giovanna Martino^1^, Michaela Daniel^1^, Joanna Gora^1^, Eugen Hug^1^

##### ^1^EBG MedAustron GmbH, Medical Department, Wiener Neustadt, Austria

Recently, a novel, unconventional radiotherapy approach, known as the “PARTICLE-PATHY” (PARTICLE-based PArtial Tumor irradiation of HYpoxic segment) has purposefully been developed for an intentional induction of immuno-stimulating radiation effects. This approach was applied for those patients affected by unresectable bulky oligometastatic tumors that were unsuitable for conventional particle therapy with the purpose to improve the radiotherapy therapeutic ratio, adding to direct radiation tumor cell killing also the component of immune-mediated killing in terms of bystander and abscopal effects. In order to generate those immune radiation effects, the loco-regional immune system cells are maximally spared from radiation as an OAR known as the peritumoral immune microenvironment-PIM (the applied dose-constraints: D30%=0Gy RBE, D50%<0.8Gy RBE, Dmean<4Gy RBE). PARTICLE-PATHY delivers extremely hypofractionated radiation doses, typically 45Gy RBE in three fractions to the hypoxic segment of those large tumors which on an average represent only 30% of the GTV. Currently, we are conducting a prospective trial to assess the immunogenic potential of carbon-ions applied to this novel treatment concept. The hypothesis implies that for an effective immune modulation leading to improved therapeutic ratio, the entire tumor volume may not need to be irradiated but only a partial tumor volume, to initiate the immune cycle in radiation-spared PIM, resulting in tumoricidal bystander and abscopal effect. The preliminary clinical results seen in the first eight consecutive patients are very encouraging confirming the high therapeutic potential in terms of tumor response devoid of any acute or late toxicity (Fig. 1 and 2).

### O 041 - Robustness of proton SBRT for prostate cancer in consideration of inter-fraction anatomy variations

#### Francis Yu^1^, Qing Chen^1^, Sheng Huang^1^, Brian Shen^1^, Daniel Gorovets^2^, Shaakir Hasan^3^, Charles Simone^3^, Haibo Lin^1^

##### *^1^New York Proton Center, Physics, New York, USA*

*^2^MSKCC, Radiation Oncology, New York, USA*

*^3^New York Proton Center, Radiation Oncology, New York, USA*

**Purpose**: To evaluate the treatment robustness of proton prostate SBRT using a pre-treatment CT and on-board CBCT imaging accessing of inter-fraction anatomy variations.

**Methods**: Ten consecutive prostate cancer patients treated with SBRT to 50 fractions at the New York Proton Center were retrospectively studied. An ultrasound bladder scanner was used to ensure patients had full bladders prior to imaging. Before setting up patients on the table with on-board images, patients were re-scanned with repeat simulation CT for better image quality and more consistent anatomy within that day. Synthetic CT based on the CBCT were generated for OARs contouring with the reference to daily pre-treatment CT. The anatomy change of critical OARs including rectum, bladder and bowel, and overall plan quality were quantitatively investigated.

**Results**: The average time interval between pre-treatment CT and CBCT was around 13.5 mins over all 50 treatments, of which the 25^th^ percentile of bladder volume change and the 10^th^ percentile of rectum volume change were ∼25% and 20%, respectively. Bladder scanner measured volume showed l0-30% difference compaed to contouring volume using pre-treatment CT and CBCT for 90% of measurements.

**Conclusion**: The rectum and bladder volume change can be extreme for some patients even within short period of time. The bladder scanner is a reliable tool in monitoring bladder volume and the plan quality consistency requires additional investigation.

### O 042 - Pencil-beam scanning proton beam therapy in gastrointestinal cancers: feasibility and early outcomes from India

#### Ashwathy Mathew^1^, Nagarjuna Burela^1^, Sapna Nangia^1^, Srinivas Chilukuri^1^, Kartikeswar Patro^1^, Dayananda Shamurailatpam^1^

##### ^1^Apollo Proton Cancer Centre, Radiation Oncology, Chennai, India

**Aim:** We report here our preliminary experience of treating gastrointestinal(GI) cancers with pencil-beam scanning (PBS) proton therapy.

**Materials and Methods**: Twenty-five consecutive patients with GI cancer, who were treated with PBS proton therapy at our facility from July 2019 to January 2022 were reviewed and patient, tumour and treatment details, toxicities and outcomes were collated.

**Results:** Of 25 patients, 19 were male, with a median age of 63 years(Range:51-80) and median Charlson comorbidity index of 5(Range: 3-9). Liver was the most common primary site treated(32%), followed by esophagus(24%), pancreas(20%), rectum(16%) and one patient each with anal canal and recurrent sigmoid cancer. Sixteen patients(64%) received proton therapy as definitive treatment while nine(36%) received it as part of neoadjuvant/adjuvant treatment. Twenty percent patients received concurrent chemotherapy alongwith proton therapy and 40% received targeted and/or chemotherapy prior. Median dose delivered was 54 GyE(Range: 41.4- 67.5) in a median of 28 fractions(Range: 5-33). Among abdominal tumors(n=13), seven were treated with deep-inspiration breath-hold, two with compression belt=2, while 4 were treated in free-breathing. The maximum grade of acute toxicity observed was Grade 3 (CTCAE V4.0) in 3 patients (dermatitis-2, hematological-1). With a median follow- up of 5.5 months(Range: 1-29 months), 19 patients are alive and 80% are locally controlled at last follow-up.

**Conclusion:** PBS proton therapy is feasible, with acceptable toxicities, without compromising local control in this cohort of GI cancer patients, many of whom were heavily pre-treated and/or received concurrent chemotherapy. Longer follow-up is needed to corroborate this encouraging early experience.

### O 043 - Oblique beam angles lead to better rectal protection but worse bladder sparing in prostate cancer receiving pencil-beam-scanning proton therapy (PBS)

#### Yunze Yang^1^, Jean-Claude Rwigema^1^, Carlos Vargas^1^, Nathan Yu^1^, Sameer Keole^1^, William Wong^1^, Steven Schild^1^, Martin Bues^1^, Wei Liu^1^, Jiajian Shen^1^

##### ^1^Mayo Clinic Arizona, Department of Radiation Oncology, Phoenix, USA

**Purpose**: To investigate the dosimetric impact of beam angle changes in prostate cancer treated with pencil-beam-scanning proton therapy (PBS).

**Methods**: Ten prostate patients were included in this study. Four plans for each patient were generated, one plan with left and right lateral parallel-opposed beams and three ones from posterior oblique angles of 5°, 10°, and 15° respectively. Dose-Linear-Energy-Transfer (LET)-volume histogram was employed to study the changes in dose and LET after beam angle changes. Volume metrics, V(d,l), defined as the cumulative normalized volume that has a dose of at least d(Gy[RBE]) and an LET of at least l(keV/μm), were calculated for both rectum and bladder. To evaluate the relative volume metrics changes, the relative volume change (RVC) was used. Three relative-biological-effective (RBE) models were employed to study the biological effect from dose and LET. Effect sizes for volume metrics of all RBE doses were evaluated.

**Results**: Both high dose and LET volumes were significantly reduced in rectum using oblique angles (Fig.1). High RBE dose volume was effectively minimized at high dose in rectum with a maximum RVC of -69.7% (Fig. 2). LETs but not doses in bladders significantly increased, which caused a maximum RVC of 34.6% at high dose. Both magnitudes and effect sizes of the increase in bladder were less than those of the decrease in rectum.

**Conclusions**: Oblique angle plans achieved better dose and LET sparing of rectum while sacrificing LET sparing in bladder. Changing beam angles could be a good strategy to redistribute LET distributions.

### O 044 - Pencil beam scanning intensity modulated proton therapy (PBS IMPT) for squamous cell anal cancer: Is toxicity related to significant consequences?

#### Pavel Vitek^1^, Jiri Kubes^1^, Vladimir Vondracek^1^, Lubomir Zamecnik^1^, Radek Zapletal^1^

##### ^1^Proton Therapy Center Czech, Proton radiotherapy, Praha 8, Czech Republic

A significant improvement in toxicity of definitive chemoradiotherapy was referred for PBS IMPT. The single institution study presented here had a primary objective to confirm high efficacy of PBS IMPT. The secondary objective was to assess what toxicity is limiting for PBS IMPT. The patients anal SCC received PBS-IMPT, 230 MeV. Treatment was administered in 2 volumes: Tumor plus involved nodes and regional node groups respectively. The dose 57,5 GyE and 45 GyE was administered to volume 1 and 2 in 25 fractions, simultaneous integrated boost, with concomitant chemotherapy cisplatinum (CDDP) plus capecitabine. Treatment effect was assessed upon DRE and MRI. Toxicity was scaled using CTCAE criteria. Forty-two patients were eligible, median follow up is 36 months, 39 patients are alive. The relapse free and colostomy free survival were 94% and 93 %. 39 patients (92,9%) achieve a complete regression. 2 patients underwent salvage resection, 1 due to severe chronic toxicity. Grade 3 and 4 myelotoxicity rate was 7,5% and 5,0%. Other grade 3-4 toxicity was dermatitis (23,3%), diarrhoea 7,5% and dehydration (7,57%). Chronic toxicity emerged as skin atrophy/ulceration – grade 3-4 (5,8%) and radiation proctitis grade 3 (2,9%). PBS IMPT achieves a high complete regression rate. The myelotoxicity grade 3-4 remains low. The acute toxicity completely resolved, had no lethal outcome and never resulted in colostomy. It is the chronic toxicity - skin ulceration, fistulation, fibrosis - what results in significant morbidity requiring salvage surgery and/or colostomy. A challenging question remains, how far is PBT able to save chronic toxicity.

### O 045 - Hybrid proton-photon and fraction optimization for combined proton-photon treatment

#### Wangyao Li^1^, Weijie Zhang^1^, Yuting Lin^1^, Ronald C Chen^1^, Hao Gao^1^

##### ^1^University of Kansas Medical Center, Radiation Oncology, Kansas City, USA

**Purpose:** Hybrid proton-photon radiotherapy (RT) can provide better plan quality than proton or photon only RT, in terms of robustness of target coverage and sparing of organs-at-risk (OAR). This work develops a hybrid treatment planning method that can optimize the number of proton and photon fractions as well as proton and photon plan variables, so that the hybrid plans can be clinically delivered day-to-day using either proton or photon machine.

**Methods:** In the new hybrid treatment planning method, the total dose distribution (sum of proton dose and photon dose) is optimized for robust target coverage and optimal OAR sparing, by jointly optimizing proton spots and photon fluences, while the target dose uniformity is also enforced individually on both proton dose and photon dose, so that the hybrid plans can be separately and robustly delivered on proton or photon machine. To ensure the target dose uniformity for proton and photon plans, the number of proton and photon fractions is explicitly modeled and optimized, so that the target dose for proton and photon dose components is constrained to be a constant fraction of the total prescription dose while the plan quality based on total dose is optimized.

**Results:** For all cases, hybrid plans provided better overall plan quality and OAR sparing than proton-only or photon-only plans, better target dose uniformity and robustness than proton-only plans, quantified by treatment planning objectives and dosimetric parameters. Moreover, for HN and brain cases, hybrid plans also had better target coverage than photon-only plans.

### O 046 - Brain stem toxicity in Carbon Ion Radiotherapy (CIRT)

#### Piero Fossati^1^, Birgit Flechl^2^, Sarah Grossahagauer^3^, Carola Lutgendorf-Caucig^2^, Eugen Hug^2^, Markus Stock^3^, Antonio Carlino^3^, Joanna Gora^3^

##### *^1^EBG MedAustron Gmbh, Radiation Oncology, Wiener Neustadt, Austria*

*^2^MedAustron Ion Therapy Center-, Radiation Oncology, Wiener Neustadt, Austria*

*^3^MedAustron Ion Therapy Center-, Medical Physics, Wiener Neustadt, Austria*

**Background***:* Brainstem toxicity is a potentially lethal complication of skull base RT. CIRT is highly precise, but dose calculations can be based on different RBE models and fractionations.

**Material and methods**: Since July 2019, 39 adult patients with tumours in the proximity of the brainstem were treated with CIRT at MedAustron. CIRT was based in 8 patients on the “German Approach” of 66 Gy (RBE) at 3 Gy(RBE), and in 31 patients on the “Japanese fractionation” (64-76.8 Gy RBE in 16 fractions of 4-4.8 Gy(RBE)). Brainstem OAR constraints were defined as Dmax < 54 Gy RBE (for 3Gy/fraction treatment) and D0.1cc < 46 Gy RBE (for 16 fraction approach), respectively. Plans of patients treated with the Japanese fractionation were recomputed with the MKM RBE.

**Results***:* With a mean follow up of 11 months (range: 3-31 months) 1 symptomatic and 2 asymptomatic brainstem events were observed. One patient developed radiographic signs of demyelination in an area with very low dose only (D0.1ccLEM < 25 Gy(RBE), D0.1ccMKM< 15 Gy(RBE)). The patient had gaze and balance disturbance (CTCAE G3) which have only minimally improved after 18 months. Its cause remains unexplained. Two additional patients developed transient, asymptomatic contrast enhancing lesions. Brainstem OAR constraints were met based on LEM – but not when recomputed according to MKM.

**Conclusion***:* Early results of high-dose CIRT for skull base tumors indicate a safe approach based on LEM-calculated brainstem OAR constraints. We recommend parallel planning strategy and evaluation using multiple RBE models for better understanding and caution.

### O 047 - Incidence of hearing impairment after proton beam therapy for malignancies of the brain/ base of skull and head and neck

#### Simona Gaito^1^, Eunji Hwang^2^, Marianne Aznar^3^, Neil Burnet^4^, Adrian Crellin^5^, Anna France^1^, Gareth Price^6^, Peter Sitch^1^, Whitfield Gillian^4^, Ed Smith^4^

##### *^1^The Christie NHS FT, Proton Clinical Outcomes Unit- Proton Beam Therapy Centre, Manchester, United Kingdom*

*^2^University of Sydney, Institute of Medical Physics- School of Physics, Sidney, Australia*

*^3^The University of Manchester, Division of Clinical Cancer Science- School of Medical Sciences- Faculty of Biology- Medicine and Health, Manchester, United Kingdom*

*^4^The Christie NHS FT, Clinical Oncology- Proton Beam Therapy Centre, Manchester, United Kingdom*

*^5^NHS England, National Clinical Lead Proton Beam Therapy, Manchester, United Kingdom*

*^6^The University of Manchester, Department of Radiotherapy Related Research, Manchester, United Kingdom*

**Aim**: To report the incidence of moderate/severe (G≥3) long-term hearing impairment (HI) and identify risk factors in patients treated for malignancies of the brain/base of skull/head and neck with Proton Beam Therapy (PBT) within the UK Proton Overseas Programme.

**Methods**: Clinical and treatment-related data from patients' files stored in a national database and curated by a dedicated outcomes unit were extracted and analysed. Toxicities were graded prospectively as per Common Terminology Criteria for Adverse Events (CTCAE 4.0), and counted if either of new onset or increased severity after PBT compared to baseline.

**Results**: Between 2008-2018, 872 UK patients received PBT overseas for the above mentioned indications. Patient demographics, toxicity and radiotherapy-related data are reported in Table 1. Of note, incidence of HI in patients <5y is 6.1%. The Relative Risk of G≥3 HI in patients <5y compared to those ≥5y and < 25 is 3.7. Of note, incidence of HI is higher in infratentorial brain tumours (Table 1), which have a higher prevalence in patients <5y (Figure 1).The incidence of HI ≥ 25y is 6.6%, which is a reflection of the high proportion of patients receiving dose-escalated treatment for radioresistant tumours in this age group (Figure 1). Granular data of cochlear radiotherapy dose and cumulative platinum dose were insufficient at this stage to develop a model to predict thresholds for HI.

**Conclusions**: Our results support PENTEC preliminary data that children < 5 years may be at highest risk of developing RT-related HI. More detailed dose-age response data will elucidate this.

### O 048 - The incidence of brainstem toxicity following high-dose conformal proton therapy for adult skull-base malignancies

#### Adam Holtzman^1^, Michael Rutenberg^1^, Alexandra De Leo^1^, Dinesh Rao^2^, Jeet Patel^2^, Daniel Indelicato^1^, William Mendenhall^1^

##### *^1^University of Florida College of Medicine, Radiation Oncology, Jacksonville, USA*

*^2^University of Florida College of Medicine, Radiology, Jacksonville, USA*

**Purpose**: Dose escalation for skull-based malignancies often presents risks to critical adjacent neural structures, including the brainstem. We report the incidence of brainstem toxicity following fractionated high-dose conformal proton therapy and associated dosimetric parameters.

**Materials and Methods**: We performed a single-institution review of patients with skull-base chordoma or chondrosarcoma who were treated with proton therapy between February 2007 and January 2020 on a prospective outcomes-tracking protocol. The primary endpoint was grade ≥2 brainstem toxicity. No patients received concurrent chemotherapy, and brainstem toxicity was censored for analysis if it coincided with local disease progression.

**Results**: We analyzed 163 patients who received a minimum of 45 GyRBE to 0.03 cm^3^ of the brainstem. Patients were treated to a median total dose of 73.8 (range, 64.5-74.4) GyRBE at 1.8 GyRBE per fraction with 17 patients undergoing twice-daily treatment at 1.2 GyRBE per fraction. With a median follow-up of 4 years, the 5-year actuarial rate of grade ≥2 brainstem injury was 1.3 (95% CI 0.25-4.3%)% (Figure 1).The median and lower and upper quartiles of the maximum dose (Dmax) to the brainstem were 65.7 GyRBE and 63.8 to 66.8 GyRBE, respectively. The median and lower and upper quartiles for the maximum dose D.03cc to the brainstem were 63.4 GyRBE and 61.2 to 64.6 GyRBE, respectively (Figure 2).

**Conclusions**: In delivering curative-intent radiotherapy for skull-base chordoma and chondrosarcoma in adults, small volumes of the brainstem can safely receive at least 64 GyRBE with minimal risk of serious brainstem injury.

### O 049 - Simultaneous integrated boost with spot-scanning proton beam irradiation for meningiomas – clinical experience of the first 100 patients from REGI-MA-002015

#### Carola Lütgendorf-Caucig^1^, Maciej Pelak^1^, Petra Georg^1^, Birgit Flechl^1^, Ulrike Mock^1^, Piero Fossati^1^, Marta Mumot^1^, Antonio Carlino^1^, Eugen Hug^1^

##### ^1^MedAustron, Ion Therapy Center, Wiener Neustadt, Austria

**Introduction**: Simultaneous integrated boost techniques based on single field optimization (SFIB) achieve superior dose distribution and shortening of overall treatment course. We report acute toxicities and clinical outcomes of the first 100 adult patients with low grade (WHO Grade I) meningioma treated with SFIB spot-scanning proton therapy at MedAustron.

**Methods**: All patients were enrolled in the MedAustron prospective registry trial (REGI-MA-002015).Target volume definition was based on CT, MR und DOTA-PETCT. CTV1 included tumour (GTV_MR/PET_), hyperostosis (if present) and 5 mm margin along the meninges, CTV2 was limited to GTV_MR/PET_. PTV margin was 3 mm. Proton PBS treatment plans were generated by applying the SFIB-optimisation method and utilizing per plan 2-4 beams with a beam spacing of >30°. Prescribed doses to PTV1 were 50.49Gy_RBE at 1.87Gy_RBE/fr and 54.0Gy_RBE at 2.0Gy_RBE/fr to PTV2 in overall 27 fractions. Follow-up visits and MRI were obtained at 6 and 12 months and then annually. Toxicity was assessed using CTCAE 4.0.

**Results**: Between August 2017 and April 2021, 100 patients with Grade I meningioma were treated. Median follow-up was 31.1 months (6.6-51.6). No > G2 acute toxicity but two G3 late events were observed (CNS necrosis). 9% of patients (n=9) developed G1/G2 radiation-induced brain lesions, which resolved spontaneously. Disease-specific survival was 100% and local control was 98%. Two local in-field failures were observed resulting in 3-year actuarial disease-specific survival of 100% and local control of 98%, respectively.

**Conclusion**: First clinical data demonstrate that SFIB is a safe and efficient technique in treatment of low grade meningiomas.

### O 050 - Protons for non-benign meningiomas

#### Stepan Vinakurau^1^, Barbora Ondrova^1^, Jiri Kubes^1^, Klara Badraoui Cuprova^1^, Vladimir Vondracek^1^, Jana Engelova^1^, Daniel Klika^1^, Sarah Falah Abass Al-Hamami^1^

##### ^1^PTC Prague, Proton Therapy, Prague, Czech Republic

**Introduction**: Malignant meningiomas (atypical [grade 2] and anaplastic [grade 3]) belong to diseases with a high rate of recurrence after surgical treatment and radiotherapy. Due to their expansive and infiltrative nature, these growths complicate the application of a sufficiently extensive surgical or radiotherapy procedure. The optimal total dose of adjuvant and radical radiotherapy is currently not precisely determined.

**Methods**: Between September 2013 and September 2021, 64 patients with non-benign meningioma were treated with intensity-modulated proton therapy (IMPT) in the Proton Therapy Center Czech in Prague. 53 of the patients presented with atypical meningiomas and 11 had anaplastic meningiomas. 20 patients (31%) received proton radiotherapy due to tumor progression after previous radiotherapy and 44 (69%) underwent primary treatment.

**Results**: The median applied dose was 61,3GyE (50,4 – 68,4) and overall local control was 89%. No signs of serious acute toxicity were observed. Late toxic effects were observed in 10 cases, where the radiation induced contrast enhancement.

**Conclusions**: Intensity-modulated proton therapy for atypical and malignant meningiomas is a well-tolerated treatment, producing long-term tumor stabilization.

### O 051 - Technical considerations for the planning and delivery of proton beam therapy for pancreas cancer: Consensus of the PTCOG Gastrointestinal Subcommittee

#### Richard Amos^1^, Eric Diffenderfer^2^, Jedediah Johnson^3^, Haibo Lin^4^, Luis Perles^5^, John Wolfgang^6^, Charles Simone^7^, Michael Chuong^8^, Huan Giap^9^, Romaine Nichols^10^

##### *^1^Unversity College London, Medical Physics & Biomedical Engineering, London, United Kingdom*

*^2^University of Pennsylvania, Radiation Oncology, Philadelphia, USA*

*^3^Mayo Clinic, Radiation Oncology, Rochester, USA*

*^4^New York Proton Center, Medical Physics and Dosimetry, New York, USA*

*^5^University of Texas MD Anderson Cancer Center, Radiation Physics, Houston, USA*

*^6^Massachusetts General Hospital, Radiation Oncology, Boston, USA*

*^7^New York Proton Center, Radiation Oncology, New York, USA*

*^8^Miami Cancer Institute, Radiation Oncology, Miami, USA*

*^9^Advanced Radiation Technologies, Radiation Oncology, Las Vegas, USA*

*^10^University of Florida Proton Therapy Institute, Radiation Oncology, Jacksonville, USA*

**Introduction**: The proximity of gastrointestinal (GI) luminal organs-at-risk (OAR) poses a challenge to dose-escalated radiotherapy (RT) for pancreas cancer (PC). Proton beam therapy (PBT) may reduce toxicities compared to photon therapy by avoiding radiation exposure to large volumes of the abdomen while enabling safe dose escalation.The PTCOG Gastrointestinal Subcommittee has developed PBT expert consensus recommendations for PC that describes simulation, treatment planning, and treatment delivery.

**Simulation**: Indexed immobilization is recommended to reduce patient setup variability. Patient should be supine with arms above the head. Additional imaging such as PET/CT and/or MRI should be considered to help target delineation. Contrast CT is recommended but should be performed subsequent to the planning CT at simulation to avoid the need to override Hounsfield Units. Dual-energy CT should be used, if available, to reduce CT-related proton range uncertainty. Motion management strategies are imperative, including 4DCT for motion accounting with deep-inspiration breath-hold or Active Breathing Control techniques for motion mitigation.

**Treatment planning**: Use of posterior and lateral beam angles is preferred to minimize impact of variable anatomy such as bowel. Robust optimization should be used for pencil beam scanning techniques. Higher linear energy transfer (LET) at the end of range should be considered in the context of sparing distal OARs. Increased beam angle separation should be used to mitigate the impact of higher LET.

**Treatment delivery**: Volumetric-image-guidance, where available, should be used daily. Verification CT scans should be acquired *ad-hoc* as deemed necessary, or at least every other week, with adaptive re-planning as required.

### O 052 - PTCOG Gastrointestinal Subcommittee lower GI tract malignancies consensus statement: proton therapy indications, treatment delivery and case discussion

#### J. Isabelle Choi^1^, Andrzej Wojcieszynski^2^, Richard Amos^3^, Huan Giap^4^, Smith Apisarnthanarax^5^, Jonathan Ashman^6^, Ramkumar Shanmugasundaram^7^, Jason Molitoris^8^, Charles Simone^1^, Michael Chuong^9^

##### *^1^New York Proton Center / Memorial Sloan Kettering Cancer Center, Radiation Oncology, New York, USA*

*^2^Denver Oncology, Radiation Oncology, Denver, USA*

*^3^University College London, Radiation Oncology, London, United Kingdom*

*^4^St. Joseph/Candler Hospital System, Radiation Oncology, Hilton Head Island, USA*

*^5^University of Washington, Radiation Oncology, Bellevue, USA*

*^6^Mayo Clinic Arizona, Radiation Oncology, Pheonix, USA*

*^7^Rutherford Cancer Centre, Radiation Oncology, Reading, United Kingdom*

*^8^University of Maryland School of Medicine, Radiation Oncology, Baltimore, USA*

*^9^Miami Cancer Institute, Radiation Oncology, Miami, USA*

**Background**: Lower gastrointestinal (LGI) tumors (rectal, anal) commonly require radiotherapy as part of multimodality treatment. While photon conformality and homogeneity have advanced, patients remain at significant risk for treatment complications. With superior normal tissue sparing of proton therapy (PBT), further toxicity reduction is feasible.(3)

**Objectives**: The international Particle Therapy Co-operative Group (PTCOG) GI Subcommittee conducted a systematic literature review on PBT for LGI malignancies. Based on level of evidence and panel voting, consensus recommendations on PBT applications for LGI tumors was determined.

**Methods**: Twelve international physicians and medical physicists were selected from the PTCOG GI Subcommittee as disease matter experts. Overall quality of evidence of identified literature was assigned for each recommendation (high, moderate, expert opinion). Scores on a 5-point Likert scale were assigned by physician (n=9) panelists from strongly agree to strongly disagree. Consensus was reached with ≥75% agree/strongly agree scoring (or ≥90% for recommendations based on expert opinion). Strong or conditional strength of recommendation was assigned based on panelist consensus and quality of evidence.

**Results**: Twelve recommendations were made (Table 1), each achieving consensus.

Seven (58%) recommendations were given a strength of “Strong,” whereas 5 (42%) were “Conditional.” Strongly recommended clinical scenarios included: reirradiation for local-regional recurrence, exceeding critical structure dose constraints with other modalities, pelvic/transplanted kidneys within/near target volume, adolescent/young adult patients, genetic predisposition to radiation-induced secondary malignancy, enrollment on prospective clinical trial, and ovarian function preservation.

**Conclusions**: PTCOG GI Subcommittee developed patient selection recommendations in which PBT may provide maximum benefit for lower GI tumors.

### O 053 - Proton beam therapy reirradiation for abdominal and pelvic cancers: Outcomes from the Proton Collaborative Group REG001-09 Trial

#### Michael Chuong^1^, Jing Zeng^2^, Jason Molitoris^3^, Henry Tsai^4^, John Chang^5^, Lane Rosen^6^, Craig Stevens^7^, Carlos Vargas^8^, James Urbanic^9^, William Hartsell^10^

##### *^1^Miami Cancer Institute, Radiation Oncology, Miami, USA*

*^2^University of Washington, Radiation Oncology, Seattle, USA*

*^3^University of Maryland, Radiation Oncology, Baltimore, USA*

*^4^ProCure New Jersey, Radiation Oncology, Somerset, USA*

*^5^Oklahoma Proton Center, Radiation Oncology, Oklahoma City, USA*

*^6^Willis-Knighton Cancer Center, Radiation Oncology, Shreveport, USA*

*^7^Beaumont Hospital, Radiation Oncology, Royal Oak, USA*

*^8^Mayo Clinic, Radiation Oncology, Scottsdale, USA*

*^9^California Protons Therapy Cancer Center, Radiation Oncology, San Diego, USA*

*^10^Northwestern Medicine, Radiation Oncology, Chicago, USA*

**Background**: Reirradiation (reRT) is associated with increased risk of severe toxicity. Proton beam therapy (PBT) is well-suited for reRT because of superior normal tissue sparing compared to x-ray therapy. However, there are limited published outcomes of abdominopelvic reRT.

**Materials and methods**: We evaluated 83 patients enrolled on the Proton Collaborative Group REG001-09 trial (NCT01255748) who received proton reRT to the abdomen/pelvis from 2010-2020. Acute and late toxicities were assessed per CTCAE v4.0.

**Results**: Median age was 64 years; most had ECOG performance status 0-1 (72.3%). Primary tumor site was colorectal (71.1%), otherwise cervix/uterus/vulva (16.9%) or anal canal (10.8%). Adenocarcinoma histology was most common (80.7%). Median interval from prior RT to reRT was 38.6 months. Median total prior RT dose was 50.4 Gy in a median 28 fractions. Median reRT dose was 49.2 GyE in a median 27 fractions. Median reRT BED_10_ was 57.5 Gy. Once daily treatment was common (77.1%). Uniform scanning (38%) and pencil beam scanning (41%) utilization was similar. Median follow-up was 12 months. Median and 1-year freedom from local failure (FFLF) were not reached and 77.7%, respectively. Median and 1-year overall survival (OS) was 21.0 months and 70.8%, respectively. Acute or late grade 3 toxicity rates were 3.6% and 1.2%, respectively.

**Conclusions**: To our knowledge, this is the largest analysis of PBT for abdominopelvic reRT. Severe toxicity was rare despite a median reRT BED_10_ of nearly 60 Gy_._ Future studies should further explore whether PBT can achieve safe dose-escalation for reRT, which may enhance FFLF and potentially OS.

### O 054 - Dose escalation for locally unresectable pancreatic cancer: feasibility of hypofractionated proton therapy

#### Piero Fossati^1^, Petra Georg^2^, Eugen Hug^2^, Rastko Kostamtinovic^2^, Markus Stock^3^, Joanna Gora^3^, Antonio Carlino^3^, Slavisa Tubin^2^, Giovanna Martino^3^

##### *^1^EBG MedAustron Gmbh, Radiation Oncology, Wiener Neustadt, Austria*

*^2^MedAustron Ion Therapy Center-, Radiation Oncology, Wiener Neustadt, Austria*

*^3^MedAustron Ion Therapy Center-, Medical Physics, Wiener Neustadt, Austria*

**Background***:* Locally advanced pancreatic cancer (LAPC) has a dismal prognosis. Protontherapy has been used without major attempts at treatment intensification. At Medaustron we have investigated hypofractionated protontherapy for LAPC followed by reevaluation for resectability.

**Material and methods**: Twelve patients were treated according to the following protocol: A 5 fractions utilizing a simultaneously integrated boost (SIB). Total dose was 25 Gy(RBE) to CTV1 and 37.5 or 40 Gy(RBE) to CTV2. CTV2 dose was selected based on expected risk on small bowel. CTV1 included tumor plus comprehensive lymphnode stations at risk plus neural plexus; CTV2 = tumor + 5 mm margin. Stomach and duodenum dose constraints (D1cc< 28 Gy(RBE)) were prioritized. Respiratory motion was managed by abdominal compression, 4D CT, robust planning and rescanning. Plans were optimized on multiple CT's to account for bowel motion.

**Results***:* With median FU of 9 (range 2– 21) months, 4 /12 patients are alive, 7/12 patient with local control, resulting in 1-Yr LC of 67% and OS 43%. 5/12 patients were surgically explored. 4/12 (33%) patients were converted to resectability resulting in 3 R0 and 1 R1 resections with major pathologic response. Side Effects: Proton Therapy alone - no ≥ Grade 3. Surgery - 1 patient died of multi-organ failure. Surgery + Proton Therapy: -1 patient died postoperatively of cholangitis with ileus.

**Conclusion***:* Proton Therapy is a versatile tool for local treatment intensification in LAPC with and can convert truly unresectable to resectable disease. Careful patient selection is warranted. A Phase II Trial is presently ongoing.

### O 055 - Does neo-adjuvant proton chemoradiotherapy for esophageal cancer improve the pathological complete response rate?

#### Christina Muijs^1^, Margriet Dieters^1^, Veronique Mul^1^, Boudewijn Van Etten^2^, Jacco De Haan^3^, Gursah Kats^4^, Pietro Pisciotta^1^, Erik Korevaar^1^, Johannes Langendijk^1^

##### *^1^University Medical Center Groningen, Radiation Oncology, Groningen, Netherlands*

*^2^University Medical Center Groningen, Surgical Oncology, Groningen, Netherlands*

*^3^University Medical Center Groningen, Medical Oncology, Groningen, Netherlands*

*^4^University Medical Center Groningen, Pathology, Groningen, Netherlands*

**Purpose:** Since March 2020, esophageal cancer (EC) patients are selected for neo-adjuvant proton chemoradiotherapy (nCRT) using the model-based approach. The aim of this study was to evaluate the impact of intensity modulated proton radiotherapy (IMPT) on pathologic complete response (pCR) after nCRT.

**Methods:** Plan comparisons (IMPT vs photon radiotherapy) were performed for all EC patients eligible for nCRT, comparing dose to critical organs and the associated complication and/or mortality risks. Patients with a Δrisk >5%, and moderate target (<1.9cm) and/or diaphragm movement (<2.5cm) were selected for IMPT. The IMPT plans were robustly optimized, including 5 times re-scanning. Initially, a 2-field beam arrangement (210/180) was used, which changed into a 3-field beam arrangement (210/180/150) after the first 6 months. The results were compared to a cohort of EC patients, treated between 2014 and 2020 with photon RT and who were included in a prospective data registry.

**Results:** Forty-five out of 59 (76%) IMPT-treated patients underwent surgical resection. The historical photon cohort consisted of 312 nCRT patients, of which 265 (85%) underwent surgery (Table 1). The pCR rate was significantly higher after IMPT: 29% vs. 17% (p=0.049). In multivariate analysis, IMPT remained a significant prognostic factor. Moreover, there was a significant difference between 3- and 2-beam IMPT with a pCR rate of 39% vs. 14%, respectively. Ultimately, major pathologic response (M1+2) was significantly higher after 3-beam-IMPT compared to photon RT (71% vs. 38%;p<0.01).

**Conclusion:** Compared to photon radiotherapy, a significant improvement of pathologic response after nCRT was observed after (3-beams) IMPT.

### O 056 - Dosimetric comparison of pencil beam scanning proton therapy and VMAT in the adjuvant treatment of gynecological tumors

#### Arpit Chhabra^1^, Michael Pennock^2^, Zahra Ghiassi-Nejad^3^, J. Isabelle Choi^1^, Robert Press^1^, Shaakir Hasan^1^, Andy Shim^1^, Kaled Alektiar^4^, Charles Simone II^1^, Keyur Mehta^2^

##### *^1^New York Proton Center, Department of Radiation Oncology, New York, USA*

*^2^Montefiore Health System, Department of Radiation Oncology, New York, USA*

*^3^Mount Sinai Health System, Department of Radiation Oncology, New York, USA*

*^4^Memorial Sloan Kettering Health System, Department of Radiation Oncology, New York, USA*

**Introduction:**While randomized trials show modern photons (IMRT) reduce toxicities over 3D-CRT in the adjuvant treatment of gynecologic tumors, >30% of patients can still experience clinically significant toxicities with IMRT. Protons have the potential to reduce this risk of toxicities. We present the first dosimetric comparison between pencil beam scanning proton therapy (PBS-PT) and Volumetric Modulated Arc Therapy (VMAT) in the setting of adjuvant pelvic only and pelvic + paraaortic (PA) treatments.

**Methods:**Comparative VMAT and PBS-PT plans were created on datasets from 11 patients who received either adjuvant pelvic RT (n=6) or adjuvant pelvic + PA RT (n=5) with photons for resected endometrial or cervical cancer. CTV included the vaginal cuff and regional lymph nodes. Plans were designed to match target volume coverage, with VMAT-plans ensuring V100% > 95% and V95% > 99% to the PTV (5mm margin on CTV) and PBS-PT plans robustly optimized to ensure CTV coverage was V100% > 95% and V95% > 99% incorporating 5mm set up and 3.5% range uncertainty. Doses to organs at risk (OAR) were compared using a paired t-test statistical analysis.

**Results:**PBS-PT, as compared to VMAT, significantly reduced dose to nearly all OARs (Table 1).

**Conclusions:**PBS-PT provides a significant reduction in OAR doses in the setting of adjuvant pelvic or adjuvant pelvic + PA treatment for gynecologic malignancies. This dosimetric improvement is evident at the low and high dose range and, therefore, has the potential to reduce the risk of toxicities(Figure 1). This work is the basis for an institutional prospective protocol in development.

### O 057 - Dosimetric characteristics of localized prostate cancer patients undergoing ultra-hypofractionated intensity modulated proton therapy: a large, single institution report

#### Robert Gao^1^, Jiasen Ma^1^, Thomas Pisansky^1^, Bradley Stish^1^, Roman Kowalchuk^1^, Brendan McMenomy^2^, Richard Choo^1^, Brian Davis^1^

##### *^1^Mayo Clinic, Department of Radiation Oncology, Rochester- MN, USA*

*^2^Mayo Clinic, Department of Radiology, Rochester- MN, USA*

**Objective:** We report dosimetric characteristics in a cohort of patients with localized prostate cancer treated with stereotactic ablative radiotherapy (SABR) using intensity-modulated proton therapy (IMPT).

**Methods:** All patients receiving IMPT SABR from 2017 to 2021 for localized prostate cancer at our institution were included. Five fractions were typically delivered every other day to the prostate +/- seminal vesicles, with 3 mm/3% robustness. Most (96.8%) patients received a four-field arrangement involving two anterior oblique and two opposed lateral beams, and most (97.9%) underwent retroprostatic hydrogel spacer placement. The Eclipse Aria® treatment planning system was used to extract treatment planning data.

**Results:** A total of 534 patients were included. Seventy-nine (14.8%) had low, 238 (44.6%) favorable intermediate, 195 (36.5%) unfavorable intermediate, 20 (3.7%) high, and 2 (0.4%) very high risk disease. Prescription dose was 36.25 Gy (166, 31.1%), 38 Gy (201, 37.6%), or 40 Gy (167, 31.3%). A summary of dosimetric parameters is provided in Table 1. Patients with hydrogel spacer had improved rectal dosimetry. Those receiving a four-beam arrangement had lower dose to the femoral heads and penile bulb.

**Conclusions:** We provide the largest report to date of prostate cancer patients treated with IMPT-based SABR. Use of a retroprostatic spacer and four-beam arrangement reduces dose to organs-at-risk. A spacer more readily permits anterior oblique fields when considering range uncertainty which might otherwise result in placement of the Bragg peak in the rectum. Anterior oblique fields increase robustness with shorter path length associated with these beams.

### O 058 - Reirradiation with proton therapy for recurrent prostate cancer: Results from the Proton Collaborative Group PCG 001-09 prospective registry trial

#### Shaakir Hasan^1^, Daniel Gorovets^2^, Stanislav Lazarev^3^, Madhur Garg^4^, William Hartsell^5^, Jonathan Chen^6^, Henry Tsai^7^, Carlos Vargas^8^, Carl Rossi^9^, Charles B Simone II^1^

##### *^1^New York Proton Center, Radiation Oncology, New York, USA*

*^2^Memorial Sloan Kettering Cancer Center, Radiation Oncology, New York, USA*

*^3^Mount Sinai Medical Center, Radiation Oncology, New York, USA*

*^4^Montefiore Medical Center, Radiation Oncology, New York, USA*

*^5^Northwestern Medicine Proton Center, Radiation Oncology, Chicago, USA*

*^6^University of Washington, Radiation Oncology, Seattle, USA*

*^7^Procure Proton Center New Jersey, Radiation Oncology, Somerset, USA*

*^8^Mayo Clinic, Radiation Oncology, Phoenix, USA*

*^9^California Protons Cancer Therapy Center, Radiation Oncology, San Diego, USA*

**Introduction**:We assessed the use of proton therapy (PT) to treat recurrent prostate cancer (RPC) patients enrolled on the Proton Collaborative Group prospective registry trial.

**Methods**: Efficacy and adverse events (AE) are reported for RPC patients reirradiated with PT.

**Results**: Thirty-one patients (median age 69) treated between 2012-2019 were included. Median interval since initial photon radiation was 63 months (9-187). Original Gleason scores were 3+3(n=3), 3+4(n=7), 4+3(n=6), 4+4(n=6), 4+5(n=2), and T stages were T1(n=15), T2(n=9), and T3(n=7). Previous radiotherapy ranged between 64.8-79.2 Gy in 1.8-2Gy/Fx. Recurrences were within the prostate(n=28), prostate and pelvic nodes(n=9), and postoperative bed(n=3). Median pre-reirradiation PSA and IPSS was 5.23(0.75-35.5) ng/ml and 10.5 (0-24), respectively. Twenty were treated with conventional fractionation (54-79.2 Gy), six with 30-35 Gy/5 Fx, three with 60 Gy/20 Fx, and two 70 Gy/28 Fx. Rectal balloons and spacers were used in 11 and 3 patients, respectively. Androgen suppression was given to 7 patients. At a median follow-up of 22 months, 28/31 patients had either a biochemical or radiographic response. The progression free survival was 86%, 69%, and 59% at 1, 2, and 3 years respectively. There were no grade 3+ AEs. Acute and late grade 2 urinary AEs were reported in 20(65%) and 8 (26%) cases, respectively (6 of 8 were retention). Grade 2 rectal bleeding was reported in 3 (10%) cases.

**Conclusion**: Although low grade toxicity may be higher compared to de novo radiation, the early efficacy outcomes and absence of high-grade toxicities with reirradiation with PT for RPC are encouraging.

### O 059 - Anatomical changes in the male pelvic region between the supine and upright orientations

#### Niek Schreuder^1^, Wen Hsi^2^, Michelle Lis^3^, Michael Kissick^3^, John Greenhalgh^4^, Tracy Underwood^5^, Rock Mackie^3^

##### *^1^LEO Cancer Care, Medical Physics, Knoxville, USA*

*^2^University of Florida, Medical Physics, Jacksonville, USA*

*^3^LEO Cancer Care, Medical Physics, Middleton, USA*

*^4^Fonar Corporation, Medical Physics, Melville, USA*

*^5^LEO Cancer Care, Medical Physics, Crawley, United Kingdom*

Treating and imaging patients in the upright orientation is gaining acceptance in radiation oncology and radiology and has distinct advantages over the recumbent position. We conducted an IRB approved study to investigate the positions and orientations of the male pelvic organs between the supine and upright positions. The study comprised scanning 15 male volunteers on a 0.6 T Fonar MRI scanner (aged 55 to 75 years) in the supine and upright positions with a full bladder and in the upright position with an empty bladder. The Pelvic study revealed that in the upright position the 1. position and shape of the prostate is not impacted significantly by bladder fill; 2. distance between the sacrum and the anterior bladder wall is significantly smaller; 3. Anterior-Posterior length and the bladder width is significantly larger; 4. seminal vesicles are pushed down by the bladder; and 5. normal breathing motion does not transfer significantly to the prostate region. These observed differences will positively impact upright prostate treatments by 1. reducing the risk of small bowel approximating the treatment volume; 2. prostate treatments can be done with a reduced focus on bladder fill; 3. radiation beams for treating intermediate risk prostrate can be made smaller or a larger portion of the seminal vesicles can be treated with the same beam size than typically used for supine treatments; and 4. less breathing motion imaging artefacts and less prostate motion. We will present these findings and the data analyses showing the statistical significance of the observed differences.

### O 060 - Proton arc therapy shows potential to further increase the benefit of proton therapy for oropharyngeal cancer patients

#### Bas De Jong^1^, Erik W. Korevaar^1^, Anneke Maring^1^, Chimène I. Werkman^1^, Dan Scandurra^1^, Guillaume Janssens^2^, Stefan Both^1^, Johannes A. Langendijk^1^

##### *^1^University Medical Center Groningen, Department of Radiation Oncology, Groningen, Netherlands*

*^2^Ion Beam Applications SA, Research and Development, Louvain-la-Neuve, Belgium*

**Introduction:** In the model based approach, patients qualify for proton therapy when the reduction in risk for toxicity obtained with IMPT relative to VMAT (ΔNTCP) is larger than predefined thresholds as defined by the Dutch National Indication Protocol. Proton arc therapy (PAT) is an emerging technology which has the potential to further decrease NTCP compared to IMPT. This work aims to investigate the impact of PAT on the number of oropharyngeal cancer (OPC) patients that could qualify for proton therapy upon its clinical adoption.

**Methods:** A cohort of 111 OPC patients subjected to the model-based selection procedure was investigated. When IMPT was employed, 96 (86%) patients qualified for protons and 15 (14%) patients did not. For these 15 patients treated with VMAT, robust PAT plans, employing 30 beams and 360 energy layers, were generated to evaluate the impact of PAT on the model-based selection.

**Results:** Target coverage was similar or better in the PAT plans compared to IMPT plans (table 1). In the PAT plans, integral dose was reduced by 15% relative to IMPT plans and by 52% relative to VMAT plans. PAT decreased the mean dose to organs-at-risk (OARs), further reducing NTCPs. The average ΔNTCP compared to VMAT was almost doubled in the PAT plans compared to the IMPT plans, resulting in 106 patients (95%) of the complete cohort of 111 patients (100%) qualifying for protons (figure 1).

**Conclusions:** PAT outperforms IMPT and VMAT, leading to higher ΔNTCP-values, significantly increasing the percentage of OPC patients selected for proton therapy.

### O 061 - Patient-reported dysphagia in oropharyngeal cancer with definitive proton chemoradiation (CRT) versus trans-oral surgery with adjuvant radiotherapy (TOS+ART) +/ chemotherapy

#### Mark McDonald^1^, James Bates^1^, Mihir Patel^2^, Brian Boyce^2^, Soumon Rudra^1^, Azeem Kaka^2^, Sibo Tian^1^, Jill Remick^1^, Nabil Saba^3^, William Stokes^1^

##### *^1^Winship Cancer Institute of Emory University, Department of Radiation Oncology, Atlanta, USA*

*^2^Winship Cancer Institute of Emory University, Department of Otolaryngology, Atlanta, USA*

*^3^Winship Cancer Institute of Emory University, Department of Hematology and Medical Oncology, Atlanta, USA*

**Purpose**: To compare short-term dysphagia outcomes for patients treated with definitive CRT versus TOS+ART.

**Methods**: We included T0-T2 N0-3 M0 oropharyngeal cancer treated 08/2019–08/2021 with CRT to 70 Gy or TOS+ART to 60–66 Gy +/- chemotherapy, all with proton therapy to bilateral necks. Chemo was weekly platinum-based (n=31), bolus cisplatin (n=2) or cetuximab (n=1). Primary tumor site was omitted from the RT target in 21% of TOS+ART. Dysphagia was assessed using the MD Anderson Dysphagia Inventory (MDADI).

**Results**: 42 patients were evaluable: 23 definitive CRT, 19 TOS+ART (8 RT, 11 RT+chemo) with no significant differences in age, comorbidity, or smoking between cohorts. On baseline imaging, CRT patients had a larger average primary tumor volume (18.3 vs 5.6cc, p=0.014) compared to TOS patients. Prior to adjuvant RT, TOS patients had a 19-point lower (worse) average MDADI score (86.1 vs 67.2, p=0.001) compared to non-operative patients prior to CRT. At the conclusion of radiotherapy, average MDADI scores were not significantly different between cohorts (57.5 vs 64, p=0.14). At 3 months post radiation, TOS+ART pts had a 12-point lower average MDADI score compared to those treated with CRT (76.8 vs 64.5, p=0.037). Patients treated with CRT had fewer average days of hospitalization for any reason (0.5) compared to those treated after TOS+ART (4.6, p<0.001).

**Conclusion**: Despite smaller average tumor volumes, lower dose RT, omission of chemo in 42% and omission of the primary site in 21%, TOS+ART patients reported worse MDADI scores 3 months after RT (6 months after TOS) compared to proton-based CRT.

### O 062 - Bragg peak FLASH proton therapy enables OAR sparing and ultra-high dose rate delivery: Proof-of-principle for recurrent head and neck tumors

#### Michael Pennock^1^, Shouyi Wei^2^, Haibo Lin^2^, Shaakir Hasan^3^, Arpit Chhabra^3^, J Isabelle Choi^3^, Charles B Simone II^3^, Sonam Sharma^4^, Richard Bakst^4^, Rafi Kabarriti^1^, Nancy Y Lee^5^, Minglei Kang^2^, Robert Press^3^

##### *^1^Montefiore Medical Center - Albert Einstein College of Medicine, Radiation Oncology, Bronx, USA*

*^2^New York Proton Center, Physics, New York City, USA*

*^3^New York Proton Center, Radiation Oncology, New York City, USA*

*^4^Mount Sinai Hospital, Radiation Oncology, New York City, USA*

*^5^Memorial Sloan Kettering Cancer Center, Radiation Oncology, New York City, USA*

**Purpose**: Reirradiation of recurrent head and neck cancers is a high-risk treatment. Bragg peak FLASH proton plans may help to mitigate this risk and achieve superior organ-at-risk (OAR)-sparing, ultra-high dose rate, and similar target coverage relative to other FLASH methods.

**Materials and methods**: Bragg peak and transmission FLASH plans were created and optimized for 3 recurrent head and neck patients treated with quad shot (14.8/3.7CGE) via an in-house platform. Dose rate was increased to FLASH threshold (≥40Gy/s) with a minimum MU/spot of 300 – 500. Hypofractionated doses and minimum-spot-time (MST) of 0.5 milliseconds (ms) and 2 ms were generated for each Bragg peak FLASH plan and transmission FLASH plan. Dose rate, OAR-sparing, and target coverage were evaluated.

**Results**: Bragg peak FLASH OAR dose rate coverage ranged from 35-92% for 2 ms MST, but increased to 99 -100% with 0.5 ms MST (Figure 1). Transmission FLASH OAR V40Gy/s ranged from 97 – 100% for 2 ms MST, and increased to 100% with 0.5 ms MST. The lower dose-rate partition of the OARs was associated with lower dose, occurring at the field edge or penumbra. Bragg peak FLASH improved OAR-sparing compared to transmission FLASH plans (Table 1).

**Conclusion**: For hypofractionated Bragg peak FLASH, shorter MST benefits FLASH dose-rate coverage and dosimetry conformity. Bragg peak FLASH plans achieve ultra-high dose rates using 0.5 ms MST and also superior OAR-sparing relative to transmission FLASH.

### O 063 - Empirical relative-biological-effectiveness for mandible osteoradionecrosis in head-and-neck patients receiving pencil-beam-scanning proton therapy was underestimated at moderate doses in clinical practice

#### Yunze Yang^1^, Oliver Muller^2^, Satomi Shiraishi^3^, Matthew Harper^4^, Martin Bues^1^, Mirek Fatyga^1^, Justin Anderson^1^, Samir Patel^1^, Robert Foote^3^, Wei Liu^1^

##### *^1^Mayo Clinic Arizona, Department of Radiation Oncology, Phoenix, USA*

*^2^Mayo Clinic Rochester, Department of Dental Specialties, Rochester, USA*

*^3^Mayo Clinic Rochester, Department of Radiation Oncology, Rochester, USA*

*^4^West Virginia University, School of Dentistry, Morgantown, USA*

**Purpose**: To retrospectively investigate empirical relative biological effectiveness (RBE) for mandible osteoradionecrosis (ORN) in head and neck (H&N) cancer patients treated with pencil-beam-scanning proton therapy (PBS).

**Methods**: We included 1,266 H&N cancer patients, of which, 931 patients were treated with volumetric-modulated arc therapy (VMAT) and 335 were treated with PBS. Among them, 26 VMAT and 9 PBS patients experienced mandible ORN (ORN group), while all others were included in the control group. To minimize the impact of the imbalance in clinical factors between VMAT and PBS, we formed a 1:1 case-matched patient cohort (335 patients each modality). Dose-volume-histogram indices and their associated critical volumes were extracted from the case-matched cohort for VMAT and PBS. We sought the equivalent constraint doses for VMAT so that the critical volumes of VMAT were equal to those of PBS at different physical doses. Empirical RBEs of PBSPT for ORN were obtained by calculating the ratio between the derived equivalent constraint doses and physical doses of PBS.

**Results**: Smoking history was found to be significantly associated with the ORN incidence in PBS patients only. V40Gy[RBE], V50Gy[RBE], and V60Gy[RBE] were statistically different (*p*<0.05) between the ORN and control group for VMAT and PBS (Fig.1). Empirical RBEs of 1.58(95%CI: 1.34-1.64), 1.34(95%CI: 1.23-1.40), and 1.24(95%: 1.15-1.26) were obtained for proton dose at 40 Gy[RBE=1.1], 50 Gy[RBE=1.1], and 60 Gy[RBE=1.1], respectively (Fig. 2).

**Conclusions**: RBEs were larger than 1.1 at moderate doses (between 40 and 60 Gy[RBE=1.1]) for mandible ORN. RBEs are underestimated in current clinical practice in PBS.

### O 064 - Patient selection approach based on NTCP models and DVH parameters for proton therapy in sinonasal cancer patients with orbital invasion

#### Alfredo Mirandola^1^, Stefania Russo^1^, Maria Bonora^2^, Barbara Vischioni^2^, Maria Rosaria Fiore^2^, Alessandro Vai^1^, Ester Orlandi^3^

##### *^1^CNAO Foundation, Medical Physics, Pavia, Italy*

*^2^CNAO Foundation, Radiation Oncology, Pavia, Italy*

*^3^CNAO Foundation, Radiation Oncologist, Pavia, Italy*

**Purpose:** this work aims to provide selection criteria based on Normal Tissue Complication Probability (NTCP) models and additional explanatory dose-volume histogram (DVH) parameters suitable for identifying sinonasal cancer patients with orbital invasion benefitting from proton therapy.

**Materials and methods:** a cohort of 22 patients was enrolled and two advanced radiation techniques were compared: intensity modulated proton therapy (IMPT) and photon volumetric modulated arc therapy (VMAT). Plans were optimized with a simultaneous integrated boost modality: 70 and 56 GyE in 35 fractions were prescribed to high risk/low risk CTV. Several endpoints were investigated, classified for their severity and used as discriminating paradigms driving the choice between the two techniques. In particular, for the endpoints for which NTCP models were already available, a selection criterion based on the delta-NTCP (photons NTCP-protons NTCP) was adopted. Moreover an overall analysis in terms of DVH parameters was also performed. Hence, a second selection criterion was adopted based on a weighted sum of delta-NTCP and delta-DVH.

**Results:** 4 patients out of 22 (18.2%) were suitable for IMPT due to delta-NTCP<3% for at least one severe toxicity, 4 (18.2%) due to delta-NTCP<20% for at least three concurrent intermediate toxicities and 16 (72.7%) due to the the mixed sum of delta-NTCP and delta-DVH criterion. Being some of the patients contemporary fulfilling both criteria, overall 17/22 patients (77.3%) would benefit from IMPT.

**Conclusions**: we showed that patients affected by sinonasal cancer could profit from IMPT compared to VMAT in terms of optical and central nervous system organs at risk sparing.

### O 065 - Total scalp irradiation using pencil beam scanning proton therapy for dermatofibrosarcoma protuberans

#### Robert Press^1^, Sheng Huang^1^, Erika Jang^1^, Haibo Lin^1^, Chin-Cheng Chen^1^, Shaakir Hasan^1^, J Isabelle Choi^1^, Arpit M Chhabra^1^, Charles B Simone II^1^

##### ^1^New York Proton Center, Radiation Oncology, New York, USA

**Purpose**: This report describes a patient with extensive dermatofibrosarcoma protuberans of the scalp treated with a technically challenging total scalp irradiation (TSI) plan using intensity-modulated proton therapy (IMPT).

**Materials and methods**: A 33-year-old female presented with a rapidly progressive extensive scalp lesion measuring 8cm. She underwent a wide local excision with rotational flap reconstruction. Pathology identified diffuse positive margins and high-risk features. Due to the extensive nature of disease, multifocal positive margins, and patient's young age, adjuvant IMPT-TSI was recommended. A IMPT plan was created using 4 non-coplanar fields with gradient dose match at field junctions (Figure 1A), multi-field optimization, and Monte Carlo dose calculation. Total dose delivered was 60 Gy(RBE) in 30 fractions. A comparison volumetric arc radiotherapy (VMAT) plan with similar Clinical Target Volume (CTV) coverage was generated (Figure 1B); doses to organs-at-risk (OAR) were compared.

**Results**: The patient successfully completed treatment and remains clinically without evidence of disease at 7 months. IMPT achieved acceptable CTV coverage (V95 >99%) with adequate dose homogeneity and robustness. OAR doses with IMPT were significantly reduced compared to VMAT, particularly for the normal brain, hippocampi, pituitary gland, cochlea, and optic structures (Table 1). In-vivo dosimetry with OSLD showed dose agreement with calculations.

**Conclusion**: TSI using IMPT is dosimetrically favorable with unique technical considerations. It achieves dramatic dose sparing to critical OARs compared to modern photon techniques and should be considered when irradiation of an extensive scalp volume is indicated to reduce the long-term toxicity risk of unintentional integral dose.

### O 066 - Clinical experiences with robustly optimised IMPT for nasopharyngeal carcinoma

#### Daniel Scandurra^1^, Tineke Meijer^1^, Jeffrey Free^1^, Anne van den Hoek^1^, Edwin Oldehinkel^1^, Roel Steenbakkers^1^, Hans Verbeek^1^, Stefan Both^1^, Johannes Langendijk^1^

##### ^1^University Medical Centre Groningen and University of Groningen, Department of Radiation Oncology, Groningen, Netherlands

**Background and purpose***:* To evaluate the dosimetric changes occurring over the treatment course for nasopharyngeal carcinoma (NPC) patients treated with robustly optimised intensity modulated proton therapy (IMPT).

**Materials and methods***:* 25 NPC patients were treated to two dose levels (CTV1: 70Gy, CTV2: 54.25Gy) with robustly optimised IMPT plans. Robustness evaluation was performed over 28 error scenarios using voxel-wise minimum distributions to assess target coverage and voxel-wise maximum distributions to assess possible hotspots and critical organ doses. Daily CBCT was used for positioning and weekly repeat CTs (rCT) were taken, on which the plan dose was recalculated and robustly evaluated. Deformable image registration was used to warp and accumulate the nominal, voxel-wise minimum and maximum rCT dose distributions. Changes to target coverage, critical organ and normal tissue dose between the accumulated and planned doses were investigated.

**Results***:* 2 patients required a plan adaptation due to reduced target coverage. The D98% in the accumulated voxel-wise minimum distribution was higher than planned for CTV1 in 24/25 patients and for CTV2 in 20/25 patients. Maximum doses to the critical organs remained acceptable in all patients. Other normal tissue doses showed some variation as a result of soft tissue deformations and weight change. Normal tissue complication probabilities for grade ≥2 dysphagia and grade ≥2 xerostomia remained similar to planned values.

**Conclusion***:* Robustly optimised IMPT plans, in combination with volumetric verification imaging and adaptive planning, provided robust target coverage and acceptable OAR dose variation in our NPC cohort when accumulated over longitudinal data.

### O 067 - Setup uncertainties assessed by daily CBCT for head and neck cancer: the KCGMH experience

#### Yu-Ming Wang^1^, Jen-Yu Cheng^1^, Kuo-Chiang Sung^1^, Bing-Sheng Huang^1^, Meng-Wei Ho^1^, Eng-Yen Huang^1^

##### ^1^Kaohsiung Chang Gung Memorial Hospital, Department of Radiation Oncology & Proton and Radiation Therapy Center, Kaohsiung City, Taiwan

**Purpose**: Setup uncertainties are critical for particle therapy and a value of 3mm on all directions is empirically used for HNC patients. While 3D-IG systems are adopted more widely, 2D-IG systems remain the mainstream for most centers. This study was conducted to evaluate the differences of daily setup error between 3D and 2D IG under the routine use of CBCT.

**Method and materials**: The PBT Center at KCGMH has been in operation since October 2018. All treatment rooms are equipped with 360-degree gantry and CBCT. During daily treatment, HNC patients were immobilized with customized cast. Since CBCT offers 3D images for adjustment and hence better setup evaluation, our institutional IG protocol for IMPT requires routine use of CBCT after 2D adjustment. The initial experience of setup error values for HNC assessed by CBCT after 2D were reported.

**Results**: A total of 52 HNC patients and 1280 fractions of HNC treatments at KCGMH were analyzed. The setup differences between CBCT and 2D by our institutional protocol were 0.68±0.19mm, 0.55±0.89mm, and 0.81±1.09mm in x-,y-,and z-axis, respectively; and 0.41±0.45degrees, 0.35±0.35degrees, and 0.26±0.27degrees in roll, yaw, and pitch for rotational errors. When 3mm setup uncertainties were used for RO, it could cover 99.98%, 99.22%, and 97.89% in x-,y-,and z- directions of setup errors when 2D image guidance were used.

**Conclusion**: A real-world data of IG results were reviewed, current use of 3mm for transitional setup uncertainties is sufficient for HNC patients under daily 2D IG. However, the value of rotational errors warrants further evaluation during daily setup and TPS software development.

### O 068 - Gross tumor volume margin and local control in p16-positive oropharynx cancer patients treated with proton therapy

#### Ariel Pollock^1^, Danielle Arons^2^, Gregory Alexander^1^, David Alicia^3^, Jason K. Molitoris^2^, William F. Regine^2^, Matthew Witek^2^

##### *^1^University of Maryland Medical Center, Department of Radiation Oncology, Baltimore, USA*

*^2^University of Maryland School of Medicine, Department of Radiation Oncology, Baltimore, USA*

*^3^Maryland Proton Treatment Center, Department of Radiation Oncology, Baltimore, USA*

**Purpose:** To determine if the extent of high-dose gross tumor volume (GTV) to clinical target volume (CTV) expansion is associated with local control in patients with p16-positive oropharynx cancer (p16+OPC) treated with definitive proton therapy (PT).

**Methods:** We performed a retrospective analysis of patients with p16+OPC treated with definitive PT at a single institution between 2016-2021. Patients with a pre-treatment PET/CT and post-treatment PET/CT within 4 months following completion of PT were included. An in-field failure was defined as ≥ 95% of the failure occurring within the 95% isodose (ISD) line of the high dose CTV.

**Results:** A total of 44 patients were included for analysis with a median follow up of 13 months. The median GTV to CTV expansion was 5 mm (IQR: 3.5). Twenty-five percent of patients (11 of 44) had no GTV to CTV expansion. There were no local failures.

**Conclusion:** Excellent local control was achieved using proton therapy for p16-positive oropharynx cancer independent of high dose gross tumor volume expansion. These data support judicious reduction of high dose target volume expansions with proton therapy as a harm-minimization technique for p16-positive oropharynx cancer.

### O 069 - Sacral insufficiency fractures after CIRT for sacral chordoma: dosimetric and LET analysis

#### Maria Rosaria Fiore^1^, Angelica Ghirelli^2^, Silvia Molinelli^2^, Giuseppe Magro^2^, Agnieszka Chalaszczyk^2^, Sara Imparato^3^, Antonella Donatelli^4^, Mario Ciocca^2^, Ester Orlandi^2^

##### *^1^National center of hadrontherapy CNAO, Radiotherapy, Pavia, Italy*

*^2^National Center Oncological Hadrontherapy CNAO, Radiotherapy, Pavia, Italy*

*^3^National Center Oncological Hadrontherapy CNAO, Radiology, Pavia, Italy*

*^4^Diagnostic Radiology Resicency School- University of Pavia, Radiology, Pavia, Italy*

**Introduction**: To analyze the occurrence of sacral insufficiency fractures (SIFs) after carbon-ion RT (CIRT) for sacral chordomas and its correlation with RBE-weighted dose (DRBE) and dose-averaged LET distributions (LETd).

**Methods**: Between 2013 and 2021, 76 patients underwent CIRT. The dose was 70.4 to 73.6 Gy(RBE), in 16 fractions. The volume of fractures (VF) reported during follow-up were contoured on the corresponding MRI and graded according to CTCAE v 5.0. We analyzed DVH D_RBE_ and LET_d_ distributions of the sacral bone, looking for a correlation between VF and the volume of sacrum (V_D_) receiving a dose D (ranging from 20 to 75 Gy(RBE), in steps of 5 Gy(RBE). For the VF, the minimum, median and maximum D_RBE_ and LET_d_ and the volume percentage receiving a LET_d_ > 50 keV/μm (V_50keV/μm_) were compared to the healthy bone average values.

**Results**: SIF showed in 27 patients (36%). Multiple or single fractures were found in 69% and 31% of patients respectively, the majority located in the sacral wings (Figure 1). Twenty-three (85%) patients were scored G1, 4 (15%) were classified G2. The V_D_ of sacrum receiving doses D ≥ 55 Gy(RBE) were significantly higher in the fractured patient cohort. LET_d_ distributions in the fracture volume, against the healthy bone region, the average minimum LET_d_ was significantly higher, while no difference was found in V_50keV/mm_.

**Conclusion**: The volume of sacral bone receiving high doses significantly affects the risk of developing SIF.

### O 070 - PROSPER Study: A comparative effectiveness trial of carbon ion therapy, surgery, and proton therapy for pelvic sarcomas involving the bone

#### Bradford Hoppe^1^, Todd DeWees^2^, Matthew Houdek^3^, Reiko Imai^4^, Eugen Hug^5^, Maria Rosaria Fiore^6^, Qing Zhang^7^, Juergen Debus^8^, Jon Ashman^2^, Ivy Petersen^9^, Safia Ahmed^9^

##### *^1^Mayo Clinic, Radiation Oncology, Jacksonville, USA*

*^2^Mayo Clinic, Radiation Oncology, Scottsdale, USA*

*^3^Mayo Clinic, Surgery, Rochester, USA*

*^4^QST Hospital, Radiation Oncology, Chiba, Japan*

*^5^MedAustron, Radiation Oncology, Wiener Neustadt, Austria*

*^6^CNAO, Radiation Oncology, Pavia, Italy*

*^7^Shanghai Proton and Heavy Ion Center, Radiation Oncology, Shanghai, China*

*^8^University of Heidelberg, Radiation Oncology, Heidelberg, Germany*

*^9^Mayo Clinic, Radiation Oncology, Rochester, USA*

**Background**: Standard of care treatment for pelvic sarcomas involving the bone is surgery, however, this can be associated with significant morbidity, including urinary, bowel, sexual, and mobility toxicities and decline in functional quality of life (fQOL). Yet, definitive treatment with standard photon and proton radiotherapy is associated with significant risk for relapse. Carbon ion therapy (CIT) may be able to provide low rates of relapse without the significant morbidity of surgery. The PROSPER study is being conducted to evaluate the management of these patients with these different treatment approaches.

**Methods**: The PROSPER study is a tri-continental, non-randomized, prospective three arm pragmatic trial. Eligible patients are males and females ≥ 15 years of age, Eastern Cooperative Oncology Group (ECOG) Performance status (PS) ≤ 2, newly diagnosed, histologic confirmation of pelvic chordoma, chondrosarcoma, osteosarcoma, Ewing sarcoma with bone involvement, rhabdomyosarcoma (RMS) with bone involvement or non-RMS soft tissue sarcoma with bone involvement. The arms of the study include 1) CIT (n=30) delivered in Europe or Asia, while arms 2) surgery +/- adjuvant RT(n=30), and arm 3) proton therapy (n=30), will be conducted at Mayo Clinic Florida, Rochester, and Arizona. The primary endpoint of PROSPER is to compare the 1-year change in fQOL as measured by the PROMIS-29 instrument between CIT and surgery. Additional comparisons among the 3 cohorts will include treatment toxicities, local control, and alternate fQOL (EORTC CRCQ29).

### O 071 - Evolution of radiotherapy techniques for mediastinal Hodgkin lymphoma at Institut Curie between 2005 and 2022

#### Pierre Loap^1^, Radouane El Ayashy^2^, Louisa Abbassi^3^, Alice Boilève^1^, Bénédicte Deau Fischer^4^, Lise Willems^4^, Farid Goudjil^2^, Remi Dendale^2^, Arnaud Beddok^1^, Youlia Kirova^1^

##### *^1^Institut Curie, Department of Radiation Oncology, Paris, France*

*^2^Institut Curie, Department of Radiation Oncology, Orsay, France*

*^3^Institut Curie, Department of Radiation Oncology, Saint-Cloud, France*

*^4^Hôpital Cochin, Department of Hematology, Paris, France*

**Introduction**: Proton therapy (PT) for mediastinal Hodgkin lymphoma (HL) should reduce late toxicities. This study reports the evolution of radiotherapy techniques for mediastinal HL irradiation since 2005 at Institut Curie (France).

**Methods**: Since 2005, an institutional database recorded all lymphoma patients referred to the Department of Radiation Oncology (Institut Curie, France) for radiotherapy. The aim of this study was to describe the temporal evolution of radiotherapy techniques for mediastinal HL at Institut Curie from 2005 to 2022. Normofractionated involved-site PT was implemented in this indication since 2018 and is delivered on a gantry with spirometer-controlled DIBH.

**Results**: Since 2005, 68 mediastinal HL patients were included in the institutional database, treated with 3D-RT (n=16), IMRT (n=37) or PT (n=15). The proportion of PT treatments was 0% before 2018, 33.3% in 2018, 28.9% in 2019, 28.9% in 2020, and 58.9% in 2021 (Figure 1). Among the 15 institutional patients treated with PT, 13 were women and the median age was 26 years [18–37]. There were 12 grade II unfavorable HL, 2 grade II favorable HL and 1 “grey zone” lymphoma. Median prescription dose was 30 Gy [30 Gy-36 Gy]. Grade 3 toxicity was reported in 1 patient (esophagitis), grade 2 toxicity in 1 patient (acute esophagitis), and 7 patients (46.7%) experienced grade 1 radiodermitis. With a follow-up of 3 months [0-32 months], no patient relapsed.

**Conclusion**: Less than four years since its introduction, PT has replaced IMRT as the main radiotherapy technique for mediastinal HL irradiation.

### O 072 - Efficacy of the protons beam alone or with high-energy photons in the treatment of inoperable sacral chordoma

#### Hamid Mammar^1^, Katarzyna Holub^1,2^, Thibault Passeri^3^, Loic Feuvret^1,4^, Sylvie Helfre^1^, Farid Goudjil^1^, Isabelle Pasquie^1^, Remi Dendale^1^, Sébastien Froelich^3^, Valentin Calugaru^1^

##### *^1^Institut Curie - Protontherapy Center, Radiation Oncology Department, Paris, France*

*^2^University of Barcelona, SEOR-CRIS Foundation, Barcelona, Spain*

*^3^Lariboisière Hospital- Assistance Publique – Hôpitaux de Paris- University of Paris, Department of Neurosurgery, Paris, France*

*^4^Hôpital La Pitié Salpêtrière, Radiation Oncology Department, Paris, France*

**Background**: Sacral chordomas are rare, radio-resistant, aggressive and locally invasive notochordal tumors treated with surgery followed by high-dose adjuvant radiotherapy (RT) in case of subtotal resection. Large lesions may be inoperable or present an important risk of neurological sequelae. Exclusive proton therapy (PT) +/- photons (Ph) is proposed in selected patients. Here, we compare the outcomes of patients treated with exclusive or postoperative RT.

**Methods**: Data of all consecutive patients (n=34) with primary sacral chordoma treated with exclusive RT (n=10) or postoperative RT (n=24) at the Orsay Protontherapy Center – Curie Institut (ICPO) with mean dose of 73.8 GyRBE administered to CTV between March 2013-November 2018 were retrospectively reviewed. Thirty-one patients were treated with double component radiation PT + Ph and 3 patients were treated with PT component alone. Local control (LC), overall survival (OS), chordoma-related survival (CRS) were estimated using Kaplan-Method method and toxicity using CTCAE v5.0.

**Results**: After the median follow-up of 39.5 months (range 7.2-85.9), patients treated with exclusive RT [PT (n=1); PT + Ph (n=9)] presented OS 80.0%, CRS 90.0% and LC 77.8%, while patients treated with surgery+ RT [PT (n=2); PT + Ph (n=22)] showed OS 79.2%, CRS 83.3% and LC 73.9%. Postoperative sequelae were observed in 15/24 surgically treated patients, while post-RT toxicity in 5 non-operated patients.

**Conclusions**: Exclusive RT is an effective and safe treatment option in inoperable sacral chordoma or in case of a high risk of postoperative sequelae. Surgery had no impact on patients' survival and local control, but increased toxicity.

### O 073 - Proton therapy in mediastinal lymphoma patients in a nationwide cohort, using model-based selection

#### Anne Niezink^1^, Stefan Hutschemaekers^2^, Pietro Pisciotta^1^, Richard Canters^3^, Patricia Cambria Lopes^2^, Gloria Vilches Freixas^3^, Johannes Langendijk^1^, Marco van Vulpen^2^, Liesbeth Boersma^3^, Anne Crijns^1^, Bastiaan Ta^3^

##### *^1^University Medical Center Groningen, Radiation Oncology, Groningen, Netherlands*

*^2^HollandPTC, Radiation Oncology, Delft, Netherlands*

*^3^Maastricht University Medical Center+-, Radiation Oncology, Maastricht, Netherlands*

**Purpose**: Since 2019, mediastinal lymphoma (ML) patients in the Netherlands are treated with IMPT after model-based selection (MBS). MBS is based on the difference in normal tissue complication probability (ΔNTCP) for lifetime acute coronary events (ACE). The objective of this study was to evaluate dose advantages of both qualifying and non-qualifying patients.

**Material and methods**: All 61 ML patients referred for plan comparisons to the Dutch proton centres were included. The estimated clinical benefit in terms of ΔNTCP was derived from (par)VMAT and IMPT treatment plans using the ACE NTCP-model which includes mean heart dose (MHD), age, gender and risk factors for ACE as predictors. The ΔNTCP threshold for MBS is set on ≥ 2%. The differences in mean dos to the heart (MHD), lungs (MLD), oesophagus (MED) and breast (MBD) were evaluated.

**Results**: Thirty-two out of 61 patients (52%) met the predefined ΔNTCP threshold and were eligible for IMPT *(Table)*. In these cases, IMPT resulted in statistically significantly lower MHD, MLD, MED, MBD-right and MBD-left. Using the ACE NTCP-model, the mean NTCP for VMAT was 12.1% compared to 8.6% for IMPT, an average ΔNTCP with IMPT for ACE of 3.4% Also, in non-qualifying patients the dose to all OARs was significantly reduced *(Figure)*.

**Conclusion**: IMPT resulted in significant MHD reductions and a subsequent 3.4% ΔNTCP for lifetime risk of ACE. However, in patients not qualifying for IMPT, significant dose reductions to several OARs were obtained that are relevant for prevention of secondary tumour induction. Tools are needed to further optimise patient selection.

### O 074 - MR-based follow-up after radiotherapy in uveal melanoma

#### Jan-Willem Beenakker^1,2,3^, Michael Tang^1,2^, Teresa Ferreira^2^, Myriam Jaarsma-Coes^1,2^, Nanda Horeweg^3^, Yvonne Klaver^3,4^, Myra Rodrigues^3,4^, Khanh Vu^1^, Marina Marinkovic^1^, Gregorius Luyten^1^

##### *^1^Leiden University Medical Center, Ophthalmology, Leiden, Netherlands*

*^2^Leiden University Medical Center, Radiology, Leiden, Netherlands*

*^3^Leiden University Medical Center, Radiation Oncology, Leiden, Netherlands*

*^4^HollandPTC, n/a, Delft, Netherlands*

**Purpose:** After ocular proton therapy (PT), treatment response is conventionally defined as a reduction of tumour prominence. This can take more than half a year to become apparent. As MRI is increasingly used in the diagnosis and therapy planning of these patients, we aimed to assess if it can provide valuable information in their follow up.

**Methods:** 29 uveal melanoma patients were scanned before, 3, 6 and 12 months after brachytherapy(n=13) or PT(n=16) at 3Tesla MRI. Tumor prominences were compared between MRI and ultrasound and tumour perfusion was assessed.

**Results:** Pretreatment prominences were comparable on MRI and ultrasound (mean absolute difference 0.43mm). MRI showed larger reductions in prominence compared to ultrasound at all time points (e.g mean reduction 1.2mm vs 0.8mm at 3months). Pretreatment, 18 of 29 (62%) UM showed a washout perfusion curve. At 3 months post treatment 86% of patients showed a reduction in washout (average 40.2% reduction in outflow), also when no decrease in prominence was yet apparent, Figures 1,2.

**Conclusions:** MRI- and ultrasound-based prominences are generally in agreement. Decrease in perfusion on MRI is an early response indicator in UM patients, and can already be observed 3 months after treatment.

### O 075 - Proton therapy vs. CyberKnife for eye melanoma: How do they differ?

#### Emmanuelle Fleury^1,2^, Petra Trnková^3^, Emine Kiliç^4,5^, Wilhelm den Toom^1^, Caroline van Rij^1^, Andras Zolnay^1^, Kees Spruijt^2^, Joël Herault^6^, Jean-Philippe Pignol^7^, Mischa Hoogeman^1,2^

##### *^1^Erasmus Medical Center, Department of Radiotherapy, Rotterdam, Netherlands*

*^2^HollandPTC, Radiation Oncology, Delft, Netherlands*

*^3^Medical University of Vienna, Radiation Oncology, Rotterdam, Netherlands*

*^4^Erasmus Medical Center, Department of Ophthalmology, Rotterdam, Netherlands*

*^5^Erasmus Medical Center, Department of Clinical Genetics, Rotterdam, Netherlands*

*^6^Centre Antoine Lacassagne, Department of Radiotherapy, Nice, France*

*^7^Dalhousie University, Department of Radiation Oncology, Halifax, Canada*

**Purpose/Objective:** Both proton beam therapy and fractionated stereotactic radiotherapy have reported a high local tumor control for uveal melanomas, but comparative data regarding the likelihood of post-therapy complications derived from imaging-based planning are lacking. For the first time, we report an imaging-based dosimetrical comparison of both treatments. Dosimetric parameter values were selected as predictors of potential radiation-induced toxicity.

**Material and methods:** Retrospective CT-based CyberKnife treatment plans of 39 patients were used to generate proton plans. Dosimetric findings were analyzed for three patient groups based on tumor-optic nerve distance and uveal melanoma AJCC staging. Population-averaged spider maps were generated to compare the dosimetric parameters between both modalities.

**Results**: The spider maps are shown in Fig1. Across the 3 groups, the proton plans decreased the D_2% _of the optic nerve compared to the CyberKnife plans (median=17.9 vs. 38.0GyRBE (group A); 3.1 vs. 37.9GyRBE (group B); 0.1 vs. 11.8GyRBE (group C)). However, a decrease in D_2%_ of the anterior segment was obtained with the CyberKnife compared to protons (median=12.9 vs. 42.2GyRBE (group A); 32.2 vs. 55.0 GyRBE (group B); 13.6 vs. 37.3GyRBE (group C)). Dose surface maps of the optic nerve are shown in Fig2 for a patient example belonging to group A.

**Conclusion:** By generating CT-based proton therapy planning, both treatments could be compared to each other for the first time in radiotherapy. Whereas proton therapy has the potential to decrease the risk of optic neuropathy compared to the CyberKnife, radiation-induced complications occurring in the anterior segment of the eye might be lower with the CyberKnife.

### O 076 - EYEPLAN vs. OCTOPUS: a treatment plan comparison for ocular proton therapy in a matched-pair cohort of 1268 patients

#### Alexandra Hochreiter^1^, Jens Heufelder^2^, Dino Cordini^2^, Roland Stark^2^, Andreas Weber^2^, Antonia M. Joussen^3^, Johannes Gollrad^1^

##### *^1^Charité - Universitätsmedizin Berlin, Department of Radiation Oncology and Radiotherapy, Berlin, Germany*

*^2^Charité - Universitätsmedizin Berlin, BerlinProtonen am HZB, Berlin, Germany*

*^3^Charité - Universitätsmedizin Berlin, Department of Ophthalmology, Berlin, Germany*

**Aim:** Proton beam radiation therapy is an established primary treatment of uveal melanoma. In 2006 our institution replaced EYEPLAN, the most widely used treatment planning software for ocular proton therapy by OCTOPUS - firstly allowing the full integration of 3D image datasets (CT/MRI) and more accurate eye modelling. However, the possible advantages of OCTOPUS have not been evaluated during routine clinical practice.

**Methods:** We reviewed 3735 patients treated for uveal melanoma between 1998 and 2021. After performing a propensity-score matching between patients planned with EYEPLAN and OCTOPUS based on tumor characteristics and distance of the tumor model to ocular risk structures, we compared dose-volume characteristics grouped by the CTV distance to the respective critical structure.

**Results:** A total 1268 patients fulfilled the matching criteria. In a distance above 2 mm from the optic disc (macula) 96.9% (91.9%) of patients planned with OCTOPUS and 79.9% (71.8%) planned with EYEPLAN showed no dose exposure to the respective structure. In distance of 1.5-2 mm, we observed a significantly reduced mean dose to the macula (16.0 vs 1.0 CGE) and optic disc (7.0 vs 2.0 CGE) in patients planned with OCTOPUS (p<0.009). In contrast, these patients showed a higher dose exposure to risk structures of the anterior segment, especially when the tumor prominence exceeded 4.0 mm.

**Conclusion:** Dose calculation in both planning systems is practically identical. Therefore, a detailed analysis of the results will be presented. Some reasons might be experience, more confidence in eye modelling with OCTOPUS, and increased use of wedges.

### O 077 - Fusion of MRI and Fundus imaging for eye modelling in ocular proton therapy

#### Riccardo Via^1^, Alessia Pica^1^, Chiara Paganelli^2^, Giovanni Fattori^1^, Antony Lomax^1^, Ann Schalenbourg^3^, Damien Weber^1^, Guido Baroni^2^, Jan Hrbacek^1^

##### *^1^Paul Scherrer Institut, Center for Proton Therapy, Villigen, Switzerland*

*^2^Politecnico di Milano, Dipartimento di Elettronica Informazione e Bioingegneria, Milano, Italy*

*^3^Jules-Gonin Eye Hospital- FAA, Department of Ophthalmology- University of Lausanne, Lausanne, Switzerland*

**Introduction**: A novel approach for tumour modelling in ocular proton therapy (OPT), using fused magnetic resonance imaging (MRI) and panoramic colour fundus photographs, has been compared to the conventional, clips-based modelling.

**Materials and methods**: MRI scans were acquired on 12 uveal melanoma patients undergoing OPT at PSI. For each, Digitally Reconstructed Fundus Photography (DRFP) were generated from MRI volumes and directly compared to panoramic fundus photography to achieve 2D-3D fusion with an MRI-based eye model (see Fig. 1). In addition to providing a more complete definition of the target volume base (MRI+F_TV_) in comparison to MRI alone (MRI_TV_), this approach allows for the precise determination of the fovea position in the 3D eye model. MRI+F_TV_ and MRI_TV_ were compared to the conventional tumour model (CONV_TV_).

**Results**: MRI+F_TV_ exhibited a lower discrepancy, in terms of clips-to-tumour distances, than MRI_TV_ when compared to clips-based definition of target volumes (CONV_TV_) (0.94mm±1.36 and 1.97mm±1.46mm, respectively). Importantly, tumour regions invisible on MRI scans were successfully included in the target volume definition through the fusion of fundus images and MRI (see Fig. 2). In addition, the developed macula definition approach improved the personalisation and anatomical accuracy of the eye model.

**Discussion**: The limit in resolution of ophthalmic MRI imaging prevents its use as the sole source of information for target volume definition in OPT. The additional integration of panoramic fundus imaging allows for the complete inclusion of the lesion in the target volume, up to now only possible with the implantation of surgical markers.

### O 078 - Treating ocular melanoma on a modified pencil beam scanning system: evaluation of plan quality

#### Roelf Slopsema^1^, Anees Dhabaan^1^, Matthew Goette^1^, David Qian^1^, Richard L.J. Qiu^1^, Hui-Kuo Shu^1^

##### ^1^Emory University, Emory Proton Therapy Center, Atlanta, USA

The Ocular Option (OO) is a conceptual add-on to the ProBeam pencil beam scanning system that allows for multi-beam, intensity-modulated treatments of ocular lesions by introduction of beam-specific apertures. (Figure 1) We implemented a 3D contouring and treatment-planning process in RayStation to optimize robust treatment plans for this system. An MRI scanning protocol using a 70-mm loop coil was developed to maximize tumor contrast. (Figure 2) The MRI images were combined with CT, optometric measurements, and fundus photographs in the RayOcular module to create an eye model and delineate the tumor. A deformable registration script rotated the CT eye anatomy to the desired gaze. OO plans were inversely optimized on patient CTs minimizing dose to both ocular and extra-ocular OARs. Robust optimization ensured target coverage under 1mm / 5% uncertainties. The OO plan quality was evaluated against both iodine-125 plaque brachytherapy as well as a dedicated proton eyeline. (Figure 2) For thirteen uveal-melanoma patients these three types of plans were created, converted to biologically-effective dose, and evaluated against published dose limits for different types of toxicities (specifically optic neuropathy, neo-vascular glaucoma, and dry eye syndrome). Compared to the eyeline and plaque plans, OO plans allow better sparing of the anterior segment at the expense of an increase in extra-orbital irradiated volume and increased max dose to OARs directly abutting the tumor. The increased flexibility of the OO inverse plan optimization allows for a more deliberate balancing of trade-offs and often results in a better compromise between competing dose constraints.

### O 079 - Proton whole lung irradiation

#### Danielle Cunningham^1^, William Breen^1^, Trey Mullikin^2^, Thomas Bradley^1^, Kasie Sorenson^1^, Sheri Spreiter^1^, Jedediah Johnson^1^, Safia Ahmed^1^, Nadia Laack^1^, Anita Mahajan^1^

##### *^1^Mayo Clinic, Radiation Oncology, Rochester, USA*

*^2^Duke University, Department of Radiation Oncology, Durham- NC, USA*

All patients who received proton whole lung irradiation (PWLI) at a single institution were reviewed. Patients were immobilized with a thermoplastic mask with arms down. 4DCT was performed and if diaphragmatic motion exceeded 1.0cm, pts received motion management, often with layer-based rescanning. The goal was to limit water-equivalent thickness variation from diaphragmatic motion while reducing dose heterogeneity from the interplay effect. Two posterior oblique fields from opposing sides were used for IMPT planning with multi-field optimization to achieve maximal sparing of surrounding organs at risk, with a 2.5-cm range shifter to ensure full dose to the proximal regions of the target. Twelve patients received PWLI, with a median age of 13.7y (range 11-17y) with a median follow-up of 16.5 months (range 0.2-40.4 months). Primary diagnoses were Ewing sarcoma (n=9), rhabdomyosarcoma (n=2), and Wilms tumor (n=1). Seven pts had multiple bilateral pulmonary metastases at diagnosis, and five had single pulmonary metastases. PWLI was tolerated well with minimal toxicity. Thus far, n=3 have been found to have recurrent disease in the lung post-PWLI, which was lung only metastases in 1/3 patients and concurrent with distant metastases in 2/3 patients. Three additional patients experienced non-pulmonary distant recurrence. Eleven of 12 patients are currently alive. No pts have experienced pneumonitis or clinically apparent cardiac toxicity. IMRT comparison plans were generated. PWLI demonstrated benefit for heart and esophagus sparing. Mean heart dose 7.1 Gy PWLI, and 10.8 Gy IMRT (p=0.001). Mean esophagus dose 8.5Gy PWLI and 12Gy IMRT (p=0.001).

### O 080 - Volumetric computation of post-radiotherapy hippocampal atrophy as a function of radiation dose: Results from a prospective pediatric trial

#### Matthew Hall^1^, Katherine Von Werne^1^, Alexander Mohler^2^, Carolina Rojas^1^, Rupesh Kotecha^1^, Noah Kalman^1^, Martin Tom^1^, Edgar Gelover Reyes^1^, Alonso Gutierrez^1^, Minesh Mehta^1^

##### *^1^Miami Cancer Institute- Baptist Health South Florida, Radiation Oncology, Miami, USA*

*^2^Miami Cancer Institute- Baptist Health South Florida, Neuro-Oncology, Miami, USA*

**Purpose/Objectives:** MRIs were prospectively collected at baseline and during follow-up in pediatric and young adult brain tumor patients (Age<35) to measure volumetric changes in multiple brain substructures with neurocognitive, endocrine, and quality-of-life assessments. In this planned interim analysis, we model change in hippocampus volume at 6 and 12 months following intensity-modulated proton therapy (IMPT).

**Materials and methods:** As of 2/6/2021, 60 patients had enrolled on this prospective study and 45 completed 6 and 12-month assessments after IMPT. Left and right hippocampus volumes were independently measured on T1 precontrast MRI at baseline, 6-months, and 12-months after IMPT using automated software and physician-delineated contours. The relationship between mean hippocampus dose and change in volume was assessed by Pearson's correlation coefficient. A linear mixed-effects model was applied to evaluate other predictors associated with change in hippocampus volume, assuming random effects of subjects.

**Results:** Mean hippocampus dose was strongly correlated with change in hippocampus volume at 12 months after IMPT (r=−0.754, 95% CI [-0.837,-0.638], p<0.001). Hippocampus volumes were similar between software and physician contours. In the mixed-effects model, only mean hippocampus dose was significantly associated with hippocampus volume change at 6 and 12 months (p<0.001). The final model predicted changes in hippocampus volume of -3.6% and -6.2% for every 10 Gy increase in mean dose at 6 and 12 months, respectively.

**Conclusions:** Change in hippocampus volume was correlated with hippocampus mean dose at 6 and 12 months following IMPT. Future analyses will assess volumetric changes in additional brain substructures as a function of radiation dose and correlate with late effects.

### O 081 - Survey-based report on clinical practice for proton pencil beam scanning irradiation of pediatric posterior fossa tumors across Europe

#### Laura Toussaint^1^, Witold Matysiak^2^, Claire Alapetite^3^, Carme Ares^4^, Stéphanie Bolle^3^, Felipe Calvo^5^, Charlotte Demoor-Goldschmidt^6^, Jerome Doyen^7^, Jacob Engellau^8^, Semi Harrabi^9^, Ingrid Kristensen^8^, Fernand Missohou^6^, Ludvig Muren^10^, Barbara Ondrova^11^, Ester Orlandi^12^, Barbara Rombi^13^, Marco Schwarz^13^, Beate Timmermann^14^, Karen Van Beek^15^, Sabina Vennarini^13^, Anne Vestergaard^10^, Marie Vidal^7^, Vladimir Vondráček^11^, Damien Charles Weber^16^, Gillian Whitfield^17^, John Maduro^2^, Yasmin Lassen-Ramshad^10^

##### *^1^Aarhus University, Clinical Medicine/Danish Centre for Particle Therapy, Aarhus N, Denmark*

*^2^University Medical Center Groningen, Radiotherapy, Groningen, Netherlands*

*^3^Institut Curie, Department of Radiation Oncology, Paris, France*

*^4^Proton Therapy Center Quirónsalud, Department of Radiation Oncology, Madrid, Spain*

*^5^Clínica Universidad de Navarra, Proton therapy unit, Madrid, Spain*

*^6^Centre Regional Francois Baclesse, Department of Radiation Oncology, Caen, France*

*^7^Centre Antoine Lacassagne, Department of Radiation Oncology, Nice, France*

*^8^Skane University Hospital, Department of Hematology- Oncology and Radiation Physics, Lund, Sweden*

*^9^Heidelberg Ion Beam Therapy Center, Department of Radiation Oncology, Heidelberg, Germany*

*^10^Aarhus University Hospital, Danish Centre for Particle Therapy, Aarhus N, Denmark*

*^11^Proton Therapy Center Czech, Department of Radiation Oncology, Prague, Czech Republic*

*^12^CNAO National Center for Oncological Hadrontherapy, Department of Radiation Oncology, Pavia, Italy*

*^13^Trento Proton Therapy Center, Department of Radiation Oncology, Trento, Italy*

*^14^West German Proton Therapy Centre Essen WPE- West German Cancer Center WTZ- German Cancer Consortium DKTK, Department of Particle Therapy- University Hospital Essen, Essen, Germany*

*^15^Particle UZ Leuven, Department of Radiation Oncology, Leuven, Belgium*

*^16^Paul Scherrer Institute, Center for Proton Therapy, Villigen, Switzerland*

*^17^The Christie NHS Foundation Trust, Manchester Academic Health Science Centre, Manchester, United Kingdom*

**Purpose**: To evaluate proton therapy (PT) pencil beam scanning (PBS) standard of care across European PT centers for the treatment of pediatric posterior fossa tumors.

**Material and methods**: In September 2021, a web-based survey of 50 questions was distributed to 18 European PT centers treating pediatric patients with PBS. The questions explored clinical experience, patient positioning and imaging, delineation, treatment planning, dose constraints (including relative biological effectiveness (RBE) considerations) and clinical follow-up.

**Results**: Answers from 16/18 centers (89%) were received. Most centers delineated the brainstem according to the European Particle Therapy Network definition and eleven (69%) further distinguished between core and surface (2 to 5 mm threshold). Dose objectives for target and brainstem varied across centers, as well as the clinical compromises applied between target coverage and brainstem sparing. Robust optimization was used in eleven centers (69%) (3% or 3.5%/3mm uncertainty settings). Most of the centers used three-beams configuration (75%) and multi-field optimization (75%). Clinically, all centers used a fixed RBE value of 1.1. Nine centers (56%) had linear energy transfer (LET) calculation possibilities, with all but two centers using the dose-averaged LET formalism. For clinical follow-up, thirteen centers (81%) were prospectively assessing clinical toxicity and image changes in the brainstem.

**Conclusion**: Surveying clinical practice across European PT centers illustrated current differences in the treatment of pediatric posterior fossa tumors. A follow-up symposium including treatment-planning exercise is being planned, and the enthusiasm of all survey-participants in attending it reflect a high interest on this topic in the pediatric PT community.

### O 082 - Timing of adaptive proton therapy for pediatric parameningeal rhabdomyosarcoma implicated by on-treatment changes in anatomy and dosimetry

#### Jinsoo Uh^1^, Jacob Jordan^1,2^, Matthew Krasin^1^, Chia-ho Hua^1^

##### *^1^St Jude Children's Research Hospital, Department of Radiation Oncology, Memphis, USA*

*^2^University of Tennessee Health Science Center, College of Medicine, Memphis, USA*

**Purpose:** To characterize on-treatment morphologic changes in children with parameningeal rhabdomyosarcoma treated with upfront proton therapy and concurrent chemotherapy for guiding on-treatment imaging and adaptive replanning.

**Methods:** GTV was delineated on 21 simulation and 65 weekly MRI of 15 prospectively enrolled patients (aged 1–21 years). Temporal changes from baseline in volume and surface (95% Hausdorff distance) were analyzed in relation to the need for plan verification and its dosimetric consequence.

**Results:** The median time was 6 days from chemotherapy initiation to CT/MR simulation and 15 days from simulation to radiotherapy start. All but one patient showed continuously decreasing GTV (0.16–1.52%/day) after simulation. At 3 weeks from simulation, 10 patients exhibited a significant reduction by a median of 21% in volume (12–32%) and/or 7 mm in surface (4–10 mm). In the absence of replanning, these changes could lead to a reduction in CTV V95 by 7–12% (n=2) and/or an increase in D0.01cc/Dmean of optic nerve/chiasm, eye, or brainstem by 5–18% (n=6). Significant dosimetric consequences occurred in cases with 1) a considerable weight gain, 2) shift of the skin surface, or 3) tumor regression in the nasal/sinus/oral cavity that altered air/tissue components in the beam path. The subsequent GTV and dosimetry after 3 weeks from simulation demonstrated a relatively stable trend.

**Conclusions:** Alterations in proton dosimetry from tumor regression in children treated for parameningeal rhabdomyosarcoma became substantial at 3 weeks from radiotherapy simulation, indicating the need to monitor with on-treatment imaging and potential benefits of replanning.

### O 083 - Clinical outcome of pediatric, adolescents and young adults head and neck sarcoma patients treated with pencil beam proton therapy

#### Miriam Vázquez^1^, Amaia Ilundain^1^, Dominic Leiser^1^, Damien C Weber^1^

##### ^1^Paul Scherrer Institute- ETH Domain- Villigen PSI- Switzerland, Center for Proton Therapy, Villigen, Switzerland

**Background and aims:** To assess the clinical outcome of pediatric, adolescents and young adults (AYAs) patients with head and neck (HN) sarcomas treated with pencil beam scanning proton therapy (pbsPT).

**Methods:** Seventy-one patients treated between January 2001 and July 2021 were included. Median age was 9 years (0.3-37.6). Twenty-one (29.6%) were AYA patients (15-39 years). Fifty-eight (81.7%) patients were treated according to a protocol. Median radiation dose was 54 Gy (RBE) (36-73.8). Rhabdomyosarcoma (71.8%), and Ewing sarcoma (15.5%) were the most prevalent diagnosis. Kaplan–Meier, log-rank test and Cox-regression were used for the analysis.

**Results**: With a median follow-up of 37.2 months (range, 3.5 – 216.6), 12 (16.9%) patients died, one death was not related to the disease. Eight patients (11.2%) had local failure, 7 (9.8%) had distant failure and 5 (7%) had both. Actuarial 3-year OS and LC were 85% and 80%, respectively. Use of concomitant chemotherapy showed an increased likelihood of OS of 80% (HR 0.2, 95%CI: 0.05; 0.76) and 75% of LC (HR 0.25, 95%CI: 0.07; 0.92). Progressive disease after induction chemotherapy was associated with worse OS (HR 39.61, 95%CI: 4.27; 367.09). Thirteen (18.3%) patients presented acute toxicity grade 3. Four (5.6%) patients presented grade 3 late toxicity: cataract, sinusitis, otitis media and hearing impairment. No acute or late grade 4-5 toxicity was observed.

**Conclusions:** Excellent clinical outcomes were observed for children and AYAs with HN sarcoma treated with pbsPT. Concomitant chemotherapy and progressive disease after induction chemotherapy were major prognostic factors for outcome in these patients.

### O 084 - Feasibility study to achieve ultra-fast treatment delivery to treat moving targets using universal and dynamic ridge-filter and high-intensity beams

#### Vivek Maradia^1,2^, Isabella Colizzi^1,2^, David Meer^1^, Damien Charles Weber^1,3,4^, Antony John Lomax^1,2^, Oxana Actis^1^, Serena Psoroulas^5^

##### *^1^Paul Scherrer Institut, Center for Proton Therapy, Villigen, Switzerland*

*^2^ETH Zurich, Physics, Zurich, Switzerland*

*^3^University Hospital Zurich, Radiation Oncology, Zurich, Switzerland*

*^4^University Hospital Bern, Radiation Oncology, Bern, Switzerland*

*^5^Paul Scherrer Institut, Center for Proton Therapy, Zurich, Switzerland*

To treat moving tumors effectively with PBS proton therapy, it is required to use motion mitigation techniques such as breath-hold, rescanning, and gating. However, these techniques are not efficient in terms of delivery time. Treatment delivery time in PBS proton therapy depends on beam-on time and the dead time (time required to change energy layers and/or lateral position). To shorten the beam-on time, we increased the beam current reaching the patient by developing new beam optics for PSI's PROSCAN beamline and Gantry 2. Experimentally, we obtained factor 5 high beam current while achieving clinical beam size at the isocenter (Fig-a). To reduce the dead time, we developed a novel energy modulation unit that comprises two identical ridge filters placed just before the isocenter. Both ridge-filters are movable relative to each other to change the Bragg peak's characteristics dynamically (Fig-b and Fig-c). By using a dynamic ridge-filter we could reduce the number of energy layers and spots required to generate dose distribution while being independent of energy. For the feasibility study, we evaluated the effect of the ridge filter on three different geometrical shaped homogeneous water phantoms assuming single-beam plans with 1Gy/fraction (Fig-d). We calculated the dose delivery time using high beam currents and ridge-filter and compared it with the clinical setting. For all shapes, we managed to reduce the delivery time at least by 60% compared to clinically used settings (Fig-e). These methods to reduce delivery time are easily adaptable for other proton therapy facilities to treat moving targets.

### O 085 - Clinical necessity of multi-image based (4DMIB) optimization for targets affected by respiratory motion and treated with scanned particle therapy

#### Antje Knopf^1^, Christian Graeff^2,3^, Antoni Rucincki^4^, Petra Trnkova^5^, Joe Y Chang^6^, Huan Giap^7^, Wei Liu^8^, Charles B Simone II^9^, Antony John Lomax^10,11^, Arturs Meijers^1^

##### *^1^Paul Scherrer Institute, Centre for Proton Therapy, Villigen, Switzerland*

*^2^GSI Helmholtzzentrum für Schwerionenforschung GmbH, Biophysics, Darmstadt, Germany*

*^3^Technical University, Department of Electrical Engineering and Information Technology, Darmstadt, Germany*

*^4^Polish Academy of Sciences, Institute of Nuclear Physics, Krakow, Poland*

*^5^Medical University of Vienna, Department of Radiation Oncology, Vienna, Austria*

*^6^The University of Texas, M.D. Anderson Cancer Center, Houston, USA*

*^7^St. Joseph's/Candler, Nancy N and JC Lewis Cancer and Research Pavilion, Savannah, USA*

*^8^Mayo Clinic Arizona, Department of Radiation Oncology, Phoenix, USA*

*^9^New York Proton Center, Department of Radiation Oncology, New York, USA*

*^10^Paul Scherrer Institute, Center for Proton Therapy, Villigen, Switzerland*

*^11^ETH Zurich, Department of Physics, Zurich, Switzerland*

In collaboration between PTCOG Thoracic Subcommittee and ESTRO EPTN WP5, the evidence for clinical necessity of 4D multi-image-based (4D_MIB_) optimization, when treating moving targets, was reviewed*. 4D_MIB_ optimization is a form of robust optimization where different uncertainty scenarios, due to anatomy variations, are considered via multiple image sets (e.g., 4DCT). In this review, we focused on providing an overview of different 4D_MIB_ optimization implementations, introduced various frameworks to evaluate the robustness of scanned particle therapy affected by breathing motion, and summarized the existing evidence on the necessity of using 4D_MIB_ optimization clinically. Expected benefits of 4D_MIB_ optimization include more robust and/or interplay-effect-resistant doses for target volumes and organs-at-risk for indications affected by anatomical variations (e.g., breathing). Although considerable literature is available on the research and technical aspects of 4D_MIB_, clinical studies are rare and often contain methodological limitations, such as limited patient number, motion amplitude, motion and delivery time structure considerations, number of repeat CTs, etc. Therefore, the data are not conclusive. In addition, multiple studies have reported that robust 3D optimized plans result in dose distributions within the set clinical tolerances and, therefore, are suitable for a treatment of moving targets with scanned particle therapy. We, therefore, consider the clinical necessity of 4D_MIB_ optimization, when treating moving targets with scanned particle therapy, as still to be demonstrated. * AC Knopf et al., Clinical necessity of multi-image based (4D_MIB_) optimization for targets affected by respiratory motion and treated with scanned particle therapy – a comprehensive review, Radiother Oncol., 2022, doi.org/10.1016/j.radonc.2022.02.018.

### O 086 - Real-time motion-including dose reconstruction for pencil-beam scanning proton therapy

#### Simon Skouboe^1^, Thomas Ravkilde^2^, Per Rugaard Poulsen^1,3^

##### *^1^Aarhus University Hospital, Danish Center for Particle Therapy, Aarhus N, Denmark*

*^2^Aarhus University Hospital, Department of Medical Physics, Aarhus N, Denmark*

*^3^Aarhus University Hospital, Department of Oncology, Aarhus N, Denmark*

**Purpose**: Dose deterioration caused by target motion is a concern for proton pencil-beam scanning (PBS). We created and experimentally validated a real-time motion-including proton dose reconstruction algorithm that can detect dose-deviations during treatments.

**Methods**: An in-house developed pencil-beam algorithm received a simulated data stream with the spot energy, position and MU as well as the target position and calculated the dose in real-time to a moving target. Density correction was done by real-time ray-tracing through a CT volume. The pencil-beam kernels were established by Monte Carlo (TOPAS) simulation and parameterized as a depth dose curve and a depth-dependent 2D Gaussian beam profile. For experimental validation, a single-energy proton PBS plan was delivered to an ionization chamber array (MatriXX) placed on a motion stage (Quasar) (Fig 1). The plan was delivered twice with and once without 1D sinusoidal motion (40mm peak-to-peak amplitude, 5s period). Machine log files and recorded motion were used to create the data stream for post-treatment broadcasting to the dose reconstruction software, which calculated the dose. It used a synthetic CT of the experimental setup geometry for real-time ray-tracing. :The reconstructed doses were compared with the 2D MatriXX doses by 2%/2mm gamma analysis.

**Results**: The absolute dose matched well with and without motion (Fig 2). The gamma failure-rate was 0.0% (static), 1.1% (motion 1) and 2.3% (motion 2). The dose for 98.5% of the spots was calculated within 3.0ms.

**Conclusions**: Real-time proton dose reconstruction for dynamic motion was developed and experimentally validated. It reproduced measured doses well.

### O 087 - Experimental demonstration of conformal carbon ion therapy for irregularly moving tumors

#### Timo Steinsberger^1^, Marco Donetti^2^, Michelle Lis^1^, Lennart Volz^1^, Moritz Wolf^1^, Christian Graeff^1^, Marco Durante^1^

##### *^1^GSI Helmholtzzentrum für Schwerionenforschung GmbH, Biophysics, Darmstadt, Germany*

*^2^Fondazione CNAO, Research Department, Pavia, Italy*

Particle therapy of locally advanced NSCLC is challenged by irregular tumor motion, associated range changes and tumor deformations. We present a new motion mitigation strategy, synergizing 4D treatment planning with tumor tracking. It was implemented and tested at a clinical carbon facility, facing irregular motion and motion imaging errors. In 'multiphase 4D synchronized delivery (MP4D)', a dedicated conformal treatment plan is delivered in each motion phase. In the new method ‘MP4D with residual tracking' (MP4DRT), deviations between observed and planned motion are tracked laterally. We implemented MP4DRT into the dose delivery system of CNAO. It was tested experimentally for a lung tumor plan based on a periodic subset of a virtual XCAT 4DCT (planned motion amplitude 20mm). Log-file based dose reconstructions were performed on the full non-periodic XCAT CT and verified using a moving IC array. We considered variable motion amplitudes and baseline drifts. MP4DRT was compared to MP4D and rescanned tracking and ITV plans. The required precision of tumor imaging was evaluated by adding noise to the ground truth motion signal. Fig. 1 shows dose reconstructions for depicted motion scenarios with 25% amplitude variation +/- baseline drift. Only MP4DRT fully restores target coverage and conformality. Fig. 2 shows the effect of noise on the detected tumor position for MP4DRT treatments. Errors up to 1.5mm led to clinically acceptable results. We demonstrated in a clinical setting that MP4DRT is able to deliver highly conformal and homogeneous dose distributions to irregularly moving targets, even in the presence of clinically relevant imaging uncertainties.

### O 088 - Target and organ-at-risk robustness of clinical adaptive intensity-modulated proton therapy for esophageal cancer

#### Sabine Visser^1^, Cássia O.Ribeiro^1^, Margriet Dieters^1^, Pietro Pisciotta^1^, Adrian Thummerer^1^, Johannes A. Langendijk^1^, Antje Knopf^1,2^, Erik W. Korevaar^1^, Stefan Both^1^, Christina T. Muijs^1^

##### *^1^UMCG, Radiotherapy, Groningen, Netherlands*

*^2^University Hospital of Cologne, Department of Internal Medicine- Center for Integrated Oncology Cologne, Cologne, Germany*

**Purpose:** To reduce complications and prolonged hospitalization after neo-adjuvant chemoradiotherapy in esophageal cancer, Dutch health care providers allowed selecting patients for intensity-modulated proton therapy (IMPT) following the model-based approach during the COVID pandemic. We evaluated the first 20 patients treated at our proton center with IMPT, concerning target and organs-at-risk dose along the treatment course.

**Materials**: Robustly optimized IMPT plans were created on the averaged 4DCT, and robustly evaluated on averaged weekly 4DCTs. Replanning was performed if target coverage degraded. Dose accumulation over the entire treatment was performed with and without replanning to evaluate target coverage, mean heart dose (MHD) and mean lung dose (MLD) robustness. For the ten largest movers (>10mm), target coverage was additionally evaluated fraction-wise including motion and interplay effects in the adaptive setting.

**Results:** Replanning was performed 15 times in total, but could have been omitted 9 times based on the accumulated dose (Figure 1). More specifically, only replanning in the first treatment week had clinical impact. The adaptive treatment trajectories resulted in adequate accumulated target dose coverage (mean D98 95.6%), and remained adequate when considering breathing motion-induced errors (mean D98 98.4%) (Figure 2). The MHD (mean±SD) was 8.9±2.7 Gy at planning stage, compared to 9.9±3.1 Gy in the accumulated dose (p<0.05), which could be explained by diaphragm displacements. The MLD did not change (mean±SD 3.4±1.1 Gy).

**Conclusion:** Anatomical changes in esophageal cancer patients were already observed in the first treatment week, affecting target and heart doses. Adaptive workflows restored the dose distribution and assured adequate target dose coverage.

### O 089 - Monte Carlo derived correction factors for a proton graphite calorimeter operating at clinical facilities

#### John Cotterill^1^, Sam Flynn^1,2^, Russell Thomas^1,3^, David Shipley^1^, Michele Togno^4^, Sairos Safai^4^, Eunsin Lee^5^, Anthony Mascia^5,6^, Hugo Palmans^1,7^, Ana Lourenco^1,8^

##### *^1^National Physical Laboratory, Medical Radiation Science, Teddington, United Kingdom*

*^2^University of Birmingham, School of Physics and Astronomy, Birmingham, United Kingdom*

*^3^University of Surrey, Faculty of Engineering and Physical Science, Guildford, United Kingdom*

*^4^Paul Scherrer Institute, Center for Proton Therapy, Villigen, Switzerland*

*^5^Cincinnati Children's Hospital Medical Center, Medical Physics, Cincinnati, USA*

*^6^University of Cincinnati, Department of Radiation Oncology, Cincinnati, USA*

*^7^MedAustron Ion Therapy Center, Medical Physics Group, Wiener Neustadt, Austria*

*^8^University College London, Department of Medical Physics and Biomedical Engineering, London, United Kingdom*

Accurate dosimetry in proton radiotherapy is critical for the upholding of safe and consistent dose delivery to patients. The National Physical Laboratory (UK) has developed a graphite calorimeter which can accurately measure the dose delivered based on the heating of its small graphite core. Experimental campaigns were performed at the Cincinnati Children's Hospital (USA) and Paul Scherrer Institute (Switzerland) proton facilities. The Cincinnati campaign aimed to demonstrate the feasibility of operating the calorimeter in a FLASH modality to support its clinical implementation. The PSI campaign aimed to experimentally determine beam quality correction factors (kQ), which remain the main source of uncertainty in proton reference dosimetry. Beam model parameters were optimised iteratively to obtain good agreement between Monte Carlo simulated (using TOPAS v3.6.1 and FLUKA v2021.2) and experimental dose distributions, as shown in Figure 1, as well as lateral profiles. These optimised parameters were then used in simulations to calculate the correction factors k-imp, k-gap, and Dw/Dg required for the calorimeter. k-imp accounts for the non-solely graphite core, as it contains additional components such as thermistors, epoxy and wires. k-gap accounts for the vacuum gaps present in the calorimeter to minimise heat transfers by using a compensated geometry, and Dw/Dg is a dose conversion factor for converting dose-to-graphite to dose-to-water. Provisional values for k-imp and k-gap were consistently within 0.5% from unity, whereas Dw/Dg was of the order of 1.08. Optimisation of the beam parameters showed good consistency between TOPAS and FLUKA, building confidence in the calculated values for the correction factors.

### O 090 - Validation of analytical dose calculation algorithm for small field spot-scanning proton therapy

#### Sheng Huang^1^, Minglei Kang^1^, Chincheng Chen^1^, Yunjie Yang^1^, Haibo Lin^1^

##### ^1^New York Proton Center, Medical Physics, New York, USA

**Purpose**: The measurement and calculation of proton spot-scanning small fields are challenging and rarely reported. This study aims to explore the limitations of the pencil-beam convolution superposition (PCS) algorithm in Eclipse for small fields spot-scanning proton therapy through comparison to measurements and Monte Carlo calculation.

**Methods**: A series of spot-scanning spread-out-Bragg-peak (SOBP) proton plans with different field sizes (FS) (0.5×0.5cm^2^, 1.0×1.0cm^2^, 2.0×2.0cm^2^ and 4.0×4.0cm^2^ ) and proton ranges (7 cm to 20 cm) were generated. Measurements at the middle of SOBP in a water phantom were performed in a gantry beamline using a Razor compact ionization chamber (diameter=2mm, height=3.6mm) and a Razor diode (diameter=1 mm, height=0.4 mm). Razor diode was cross-calibrated with a parallel plate chamber (PPC05) at a field size of 4.0×4.0cm^2^. The PCS doses were compared with an in-house fast Monte Carlo calculation algorithm (MC) and measurements.

**Results**: The dose deposition efficiency, defined as *dose× treated volume/total MU,* decreased dramatically as field size decreased, from ∼1.6 cGy*cc/MU at 4.0×4.0cm^2^ to ∼0.25 cGy*cc/MU at 0.5×0.5cm^2^. Two Razor detectors showed consistent measurements, with a maximum difference of 1.8%. PCS agreed well with measurements for different FS with maximum deviations of 1.4% (FS4), 2.8%(FS2), 4.4%(FS1), and 4.6% (FS0.5). MC showed a better agreement with measurements.

**Conclusion**: Both Razor diode and ion chamber are suitable for proton small field dosimetry with caution of LET-dependence on the diode. Eclipse PCS algorithm generates reasonable results for small SOBP fields using our current clinical beam model.

### O 091 - Validation and testing of a graphite calorimeter in passively scattered and scanned beams for reference proton dosimetry

#### Ana Lourenco^1,2^, Nigel Lee^1^, Andrzej Kacperek^2^, Frances Charlwood^3^, Jamil Lambert^4^, John Pettingell^4^, Juan Vera^5^, Alejandro Mazal^5^, Hugo Palmans^1,6^, Russell Thomas^1,7^

##### *^1^National Physical Laboratory, Medical Radiation Science Group, Teddington, United Kingdom*

*^2^University College London, Department of Medical Physics and Biomedical Engineering, London, United Kingdom*

*^3^The Christie NHS Foundation Trust, Christie Medical Physics and Engineering, Manchester, United Kingdom*

*^4^Rutherford Cancer Centre South Wales, Medical Physics Department, Newport, United Kingdom*

*^5^Centro de Protonterapia Quirónsalud, Medical Physics Department, Madrid, Spain*

*^6^MedAustron Ion Therapy Center, Medical Physics Group, Wiener Neustadt, Austria*

*^7^University of Surrey, Faculty of Engineering and Physical Science, Guildford, United Kingdom*

A calibration service based on a primary-standard calorimeter for proton beam reference dosimetry does not exist. In this work, the feasibility of using a graphite calorimeter was investigated for passively scattered and scanned proton beams. The calorimeter has a nested construction of disc shaped graphite components separated by high-quality vacuum to minimise heat transfer between components and the environment. Measurements were performed at the Clatterbridge Cancer Centre and at The Christie, UK, equipped with 60 and 250MeV proton cyclotrons, respectively, and in three centres with a 230MeV synchrocyclotron at the Rutherford Cancer Centres in South Wales and the North East, UK, and at the Centro de Protonterapia Quirónsalud, Spain. The calorimeter was tested using two modes of operation: active isothermal and quasi-adiabatic modes. Measurements were performed in the middle of the SOBP. For the scattering beam, a collimator of 3 cm diameter was used with full modulation while for the scanned beams, measurements were performed in the centre of 10×10×10cm^3^ homogenous dose volumes. Absolute dose measurements derived using the graphite calorimeter were compared against those obtained with calibrated PTW Roos-type ionisation chambers according to the TRS-398 protocol. Absorbed dose values determined with the calorimeter where 0.5%-1.5% lower in comparison with the dose derived from ionisation chambers although the measurement uncertainty using the calorimeter route is about half of that reported using TRS-398 based on ^60^Co calibrations. The provision of a direct absorbed dose calibration service using a calorimeter will reduce uncertainties and improve consistency in the dose delivered to patients.

### O 092 - Characterization of the secondary radiation field in proton therapy using a Timepix detector

#### Racell Nabha^1,2^, Marijke De Saint-Hubert^1^, Olivier Van Hoey^1^, Christian Bäumer^3,4,5,6,7^, Nico Verbeek^3^, Lara Struelens^1^, Johanes Esser^3,8^, Edmond Sterpin^2,9^, Beate Timmermann^3,5,6,7^, Filip Vanhavere^1,2^

##### *^1^Belgian Nuclear Research Center SCK CEN, Radiation Protection Dosimetry and Calibration Expert Group, Mol, Belgium*

*^2^KU Leuven, Laboratory of Experimental Radiotherapy- Department of Oncology, Leuven, Belgium*

*^3^West German Proton Therapy Centre Essen, West German Proton Therapy Centre Essen, Essen, Germany*

*^4^TU Dortmund University- Department of Physics, n/a, Dortmund, Germany*

*^5^German Cancer Consortium DKTK, n/a, Heidelberg, Germany*

*^6^West German Cancer Center WTZ, n/a, Essen, Germany*

*^7^University Hospital Essen, n/a, Essen, Germany*

*^8^Heinrich-Heine-Universität Düsseldorf, n/a, Düsseldorf, Germany*

*^9^UCLouvain- Institut de Recherche Expérimentale et Clinique- MIRO Lab, n/a, Brussels, Belgium*

Protons have superior ballistics, however, secondary particles can mediate distant energy depositions. The out-of-field dose created by complex fields and dominated by neutrons, requires a detector enabling a full characterization. The goal of this work is to characterize the mixed secondary radiation field produced during the treatment of a pediatric brain tumor and to compare state of the art detector systems. Measurements were performed with a miniaturized Timepix detector placed inside an anthropomorphic pediatric phantom receiving a brain tumor treatment. The plan consisted of three pencil beam scanning fields with a range shifter. The detector was placed in different slices of the phantom outside the primary target at 3 distances from the isocenter, namely 7, 13 and 18 cm. By analyzing the pixel clusters created in the detector, particle identification, track reconstruction and spectral response allowed to make a clear distinction between the 3 positions in terms of fluence, particle type, trajectory and contribution (Fig.1). A wide-range distribution of energy deposition was observed at all the positions with a significant contribution of protons, and their count decreased by about 95% between positions 1 and 3 (fig. 3). The calculated dose equivalent in water converted from absorbed dose in silicon showed a good agreement with state of the art detectors such as bubble detectors (BD-PNDs) and thermoluminescent detectors (MTS-7) and TOPAS simulations. This work demonstrates the feasibility of secondary field characterization using a Timepix detector, which allows for comparison between treatment modalities, treatment plan optimization and assessment of secondary risks.

### O 093 - Dose, flux and LET measurements of scattered radiation including thermal neutron detection in proton therapy using active pixel detectors Timepix3

#### Cristina Oancea^1^, Lukas Marek^1^, Jiri Pivec^1^, Granja Carlos^1^, Jan Jakubek^1^, Elisabeth Bodenstein^2^, Jörg Pawelke^2,3^, Jaroslav Solc^4^, Zdeněk Vykydal^4^

##### *^1^ADVACAM, Research, Prague, Czech Republic*

*^2^OncoRay - National Center for Radiation Research in Oncology- Faculty of Medicine and University Hospital Carl Gustav Carus- Technische Universität Dresden- Helmholtz-Zentrum Dresden-Rossendorf- Dresden- Germany, OncoRay - National Center for Radiation Research in Oncology, Dresden, Germany*

*^3^Helmholtz-Zentrum Dresden-Rossendorf- Institute of Radiooncology - OncoRay- Dresden- Germany, Laser Radiooncology, Dresden, Germany*

*^4^Czech Metrology Institute CMI- Radiova 1136- 102 00 Prague 15- Czech Republic, Metrology Department, Prague, Germany*

This work aims to quantify the contribution of secondary particles including neutrons produced in low- and ultra-high-dose rate (UHDR) proton beams. Spectral and component characterization of the mixed field is performed with a miniaturized MiniPIX-Timepix3-Flex pixel detector with a silicon sensor and thermal neutron converter (^6^LiF) – see Fig. 1. Reference calibrating measurements were performed at well-defined radiation fields including thermal neutron source (Fig. 1) at CMI Prague. Measurements in proton beams were performed in water-phantoms (Fig. 2) at the University Proton Therapy Dresden. Particle flux, dose rate (DR) and linear energy transfer (LET) spectra are determined. At low-intensity beams, single tracks are registered in 3D (Fig. 2b) including LET and single-particle track analysis (Fig. 2d). At UHDR Timepix3 measures the integrated dose (Fig. 2c). A method and a calibration function for dose measurements in conventional and UHDR proton beams are provided.

### O 094 - Mailed dosimetry audit of active scanning proton beams in ten proton therapy centers

#### Marie Davidkova^1,2^, Claus E. Andersen^3^, Christina Ankjærgaard^3^, Alexandru Dasu^4^, Cinzia De Angelis^5^, Ludovic De Marzi^6^, Marijke De Saint-Hubert^7^, Daniela Ekendahl^8^, Nicholas Henthorn^9^, Anna Jelinek Michaelidesova^1^, Željka Knežević^10^, Dawid Krzempek^11^, Pawel Kukolowicz^12^, Małgorzata Liszka^4^, Stefano Lorentini^13^, Amelia Maia Leite^6^, Marija Majer^10^, Barbara Michalec^11^, Matěj Navrátil^14^, Brigitte Reniers^15^, Wioletta Ślusarczyk-Kacprzyk^12^, Marc Jan Van Goethem^16^, Anne Vestergaard^17^, Gloria Vilches-Freixas^18^, Vladimír Vondráček^19^, Michele Togno^20^, Liliana Stolarczyk^17^, Pawel Olko^21^

##### *^1^Nuclear Physics Institute of the CAS, Department of Radiation Dosimetry, Řež, Czech Republic*

*^2^National Radiation Protection Institute, Section for Research and Development, Prague, Czech Republic*

*^3^Technical University of Denmark, DTU Health Tech, Roskilde, Denmark*

*^4^Skandionkliniken, Skandionkliniken, Uppsala, Sweden*

*^5^Istituto Superiore di Sanità, Servizio tecnico scientifico grandi strumentazioni e core facilities, Rome, Italy*

*^6^Institut Curie, Centre de Protonthérapie d'Orsay, Orsay, France*

*^7^Belgium Nuclear Research Centre SCK-CEN, Institute of Environment- Health and Safety, Mol, Belgium*

*^8^National Radiation Protection Institute, Dosimetry Department, Prague, Czech Republic*

*^9^Proton Beam Therapy Centre, Christie NHS Foundation Trust, Manchester, United Kingdom*

*^10^Ruđer Bošković Institute, Radiation Chemistry and Dosimetry Laboratory, Zagreb, Croatia*

*^11^Institute of Nuclear Physics PAN, Cyclotron Center Bronowice, Krakow, Poland*

*^12^Maria Sklodowska-Curie National Research Institute of Oncology, Medical Physics Department, Warsaw, Poland*

*^13^Centro di Protonterapia di Trento, Department of Medical Physics, Trento, Italy*

*^14^Proton Therapy Center Czech, Medical Physics Department, Prague, Czech Republic*

*^15^Hasselt University, Research group NuTeC- Research institute CMK, Hasselt, Belgium*

*^16^UMCG, Radiotherapy Department, Groningen, Netherlands*

*^17^Aarhus University Hospital, Danish Centre for Particle Therapy, Aarhus, Denmark*

*^18^Maastro Clinic, Dept. of Medical Physics, Maastricht, Netherlands*

*^19^Proton Therapy Center Czech, Dept. of Medical Physics, Prague, Czech Republic*

*^20^Paul Scherrer Institute, Center for Proton Therapy, Villigen, Switzerland*

*^21^Institute of Nuclear Physics PAN, Division of Applications of Physics, Krakow, Poland*

Ten European proton therapy centers participated in a mailed dosimetry audit of spot scanning treatment units organized by EURADOS WG9. An assembly for passive detectors positioning in a water phantom was developed and tested in a pilot experiment in CCB IFJ PAN (Poland) and PTC (Czech Republic). In 2021, in the frame of INSPIRE joint research activity, audit manuals, detectors and holders were distributed to the participating centers. Alanine, radiophotoluminescent and thermoluminescent detectors from seven dosimetry laboratories were used. Detectors were calibrated in Co-60, an additional LET correction was not applied. The audit consisted of two plans: a single 170 MeV scanned 10 × 10 cm^2 ^field (Plan P) and a homogenous 10 × 10 × 10 cm^3 ^target volume (Plan S). An effective point of detectors was placed at the isocentre at 2 and 15 cm for Plan P and Plan S, respectively. Absorbed dose was verified using an ionization chamber and plan rescaling to deliver prescribed dose (2 or 15 Gy) was allowed. Preliminary results provide an indication of a good dosimetric agreement between participating centers. The response of alanine detectors falls within +/-1.7% and +/-2.7% of the average value for plan P and plan S, respectively. Results for TLDs are within +/-2.0% and +/-4.4% of the average value for plan P and plan S, accordingly. After a successful initial experience, mailed audit of scanning proton beams may become a part of clinical routine helping in standardization of proton beam dosimetry.

### O 095 - Ionization chamber response to protons in presence of magnetic field

#### Mathieu Marot^1,2^, Sonja Surla^1,3^, Stephan Brons^4,5^, Armin Runz^1^, Steffen Greilich^6^, Christian Karger^1,5^, Oliver Jäkel^1,5^, Lucas Burigo^1^

##### *^1^German Cancer Research Center DKFZ, Medical Physics in Radiation Oncology, Heidelberg, Germany*

*^2^University of Heidelberg, Faculty of Medicine, Heidelberg, Germany*

*^3^University of Heidelberg, Faculty of Physics and Astronomy, Heidelberg, Germany*

*^4^University Hospital Heidelberg, Heidelberg Ion-Beam Therapy Center HIT, Heidelberg, Germany*

*^5^National Center for Radiation Research in Oncology NCRO, Heidelberg Institute for Radiation Oncology HIRO, Heidelberg, Germany*

*^6^Berthold Technologies GmbH & Co. KG, Business Units Radiation Protection / Bioanalytics, Bad Wildbad, Germany*

Magnetic resonance-guided proton therapy is promising, as it combines high-contrast imaging of soft tissue with highly conformal dose delivery. However, proton dosimetry in magnetic fields using an ionization chamber is challenging because the dose distribution as well as the detector response are perturbed. This work investigates the chamber response in presence of a magnetic field aiming at the determination of correction factors, which is essential for reference dosimetry. A Farmer-type cylindrical ionization chamber (PTW 30013) is placed at 2cm depth of an in-house developed 3D printed water phantom. The detector response is measured with mono-energetic proton beams (157.43 and 202.14 MeV) having a field size 3x10cm^2^. The magnetic field is generated by an experimental electromagnet (Schwarzbeck Mess - Elektronik) providing field strengths of 0.1 to 1.0 Tesla. Both energies showed similar behavior, with a decrease of the chamber response of up to 0.3% (SE = 0.1%) at 0.2 Tesla, followed by an attenuated effect at higher magnetic field strength. The effect of the magnetic field on the polarity and recombination correction factor was ≤ 0.1%. The chamber response is affected by the magnetic field, with a decreased response for low magnetic fields. Further measurements are necessary to test ionization chambers of different volumes as well as different magnetic field directions. The response of the detectors and the correction factors have to be compared with results of Monte Carlo simulations.

### O 096 - Microdosimetric investigation of the conversion from the patient-specific anatomy to water using the CASPER phantom

#### Marta Missiaggia^1,2^, Yasmin Hamad^1,2^, Enrico Pierobon^1,2^, Francesco Giuseppe Cordoni^2,3^, Chiara La Tessa^1,2^

##### *^1^University of Trento, Department of Physics, Trento, Italy*

*^2^Trento Institute for Fundamental Physics, Istituto Nazionale di Fisica Nucleare Italian Institute for Nuclear Physics, Trento, Italy*

*^3^University of Trento, Department of Civil- Environmental and Mechanical Engineering, Trento, Italy*

Current treatment plans employ density-based conversion methods to translate patient-specific anatomy into a water system, where the dose distribution is calculated. This approach neglects differences in nuclear interactions stemming from the elemental composition of each tissue. For this reason, the tissue-to-water conversion introduces non-negligible uncertainties in the estimate of the radiation field quality, which is a necessary ingredient to calculate the dose profile. The validity of this approximation is especially relevant when the primary beam passes through tissues that are heterogeneous in thickness and density, and whose composition is very different from water. In this work, we investigated the limits of the density-based conversion for protons and carbon ions, using a Tissue Equivalent Proportional Counter (TEPC) microdosimeter in combination with the CASPER phantom (*Figure* 1). Microdosimetry is an established methodology for precisely assessing the radiation quality, providing spectra of energy deposited over the detector length between 0.1 and 1000 keV/μm. As the TEPC active volume is equivalent to a 2 μm tissue-like sphere, it can characterize the dose seen by a single cell, considering stochastic fluctuations. CASPER is a modular phantom able to simulate different anatomical regions of the human body. In this study, we focused on two geometries, brain and thorax, and used CASPER to obtain a microdosimetric characterization of the radiation field quality not only in the tumor but also in the healthy tissue. The results will be compared to standard LET_d_ values, as well as predictions from TOPAS, calculated both in water and in the specific material.

### O 097 - Microdosimetry applied to hadron therapy: towards microdosimetry standard protocols and codes of practice

#### Gabriele Parisi^1,2^, Giuseppe Schettino^1,2^, Francesco Romano^3^

##### *^1^University of Surrey, Department of Physics, Guildford, United Kingdom*

*^2^NPL - National Physical Laboratory, Medical Radiation Science Group, London, United Kingdom*

*^3^INFN - Istituto Nazionale di Fisica Nucleare- Sezione di Catania, Dipartimento di Fisica, Catania, Italy*

Microdosimetry is a dosimetry technique measuring the energy deposited by radiation into cell-like volumes, taking into account the stochastic nature of radiation interaction. It has been successfully used in radiation protection and due to the latest technological developments, it can now be applied to hadron therapy. Uniquely characterising any radiation beam, microdosimetry could provide an invaluable tool for quality assurance in treatment planning and could be successfully used to estimate the Relative Biological Effectiveness linking microdosimetric quantities to dedicated radiobiological models. However, standard protocols and codes of practice, necessary for a clinical transition of microdosimetry, are still missing. Different diamond-based detectors were characterised with low energy ions using micro-beam scanning techniques, showing promising results further supported by Geant4 Monte Carlo simulations. Different single-stage detectors showed good repeatability below 5% and could withstand fluences up to 1e7/1e8 cm-2s-1 without pile-up issues, with a resolution of 60/70keV FWHM when irradiated with mono-energetic ions. A two-stage telescope detector demonstrated the capability to discriminate the particle depositing energy. This is extremely useful when characterising mixed radiation fields found around the Bragg Peak of a typical clinical beam. A systematic uncertainty analysis focused on the effects of counting statistics was carried out using data from Monte Carlo simulations. The study revealed and quantified the effect of rare nuclear interaction events. Measurements with clinical hadron beams and intercomparison with other microdosimetry technologies are foreseen shortly. The outcomes of these studies aim at contributing to the microdosimetry transition towards its clinical use, from which treatment planning could benefit.

### O 098 - Passive detector characterization for OSLD and TLD in carbon ion beams

#### Paige Taylor^1^, Alfredo Mirandola^2^, Mario Ciocca^2^, Shannon Hartzell^1^, Stephen Kry^1^

##### *^1^MD Anderson Cancer Center, Radiation Physics, Houston, USA*

*^2^Fondazione CNAO, Fisica Medica, Pavia, Italy*

While OSLD and TLD have been studied in carbon beams, they have not been routinely used for carbon dose measurements due to the variable dose response by LET. This research characterized the dosimeter correction factors for dose calculation in a therapeutic carbon beam. The carbon linearity, fading, beam quality, and depletion (OSLD only) correction factors were determined for OSLD nanoDots and TLD-100 powder capsules used by the IROC QA Center, and were compared to the correction factors used for photon beams. The OSLD and TLD linearity were statistically different between photon and carbon beams up to 7 Gy (Figure 1). Further research will investigate the effect of LET and higher doses on linearity. The OSLD and TLD carbon fading corrections were not statistically different from the photon beam corrections. The OSLD and TLD carbon beam quality corrections could each be characterized by a third order polynomial based on the distance from measurement point to the practical range, known as the Rres value used in proton beam calibration (Figure 2). This fit applied to irradiations in both pristine and spread-out Bragg peaks. This fit accommodates the differences from LET response without requiring direct calculation of LET. The OSLD depletion was determined to be statistically different between photon and carbon beams over 60 sequential readings, but not statistically different over the course of the standard 5 sequential readings. This characterization of OSLD and TLD response in therapeutic carbon beams will facilitate easy second-checks and remote audits of carbon beam output.

### O 099 - Design of porous structure with plug-ins to broaden the carbon ion Bragg peak: A Monte Carlo simulation study

#### Sixue Dong^1^, Xiaobin Xia^1^, Yinxiangzi Sheng^2^

##### *^1^Shanghai Institute of Applied Physics- Chinese Academy of Sciences, Department of Nuclear and Radiation Safety Technology, Shanghai, China*

*^2^Shanghai Proton and Heavy Ion Center, Department of Medical Physics, Shanghai, China*

**Purpose:** Original porous structure (OPS) may cause a sizeable distal falloff width (DFW) when broadening the Bragg peak (BP) width [Inaniwa 2021]. This study aims at designing and evaluating a porous structure with plug-ins (PSP) to broaden the BP width of the carbon ion beam while keeping a sharp DFW.

**Method and material:** The PSP combines the OPS and solid sticks. The OPS was defined using a binary voxel model in Monte Carlo code FLUKA (4-1.1, CERN). The voxel size and air/water ratio were adjusted to match the similar broadening ability as the 2D-ripple filter. For the PSP, stick length (*l*) and the cross-sectional area ratio (*α*) of sticks to OPS affect the pullback ability of the BP at the distal area. The two parameters (*α* = 25%, *l* = 7 mm) were determined using an in-house iteration code to obtain the DFW as small as possible while keeping the same or better broadening ability than the OPS. DFWs, spot size, and fluence homogeneity were compared. A 3D printable POROUS-2.0 model was also provided.

**Results:** For the 234.1 MeV/u carbon ion beam, the DFW reduced 0.99 mm (31.9%) using the PSP compared to the OPS. Smaller spot sizes and better depth-dose homogeneity for the PSP than the 2D-ripple filter were observed.

**Conclusions:** The PSP possesses the required BP broadening ability while providing sharper DFW than the OPS.

### O 100 - Energy layer optimization for proton arc therapy

#### Gezhi Zhang^1^, Haozheng Shen^1^, Yuting Lin^2^, Ronald C Chen^2^, Yong Long^1^, Hao Gao^2^

##### *^1^Shanghai Jiao Tong University, University of Michigan-Shanghai Jiao Tong University Joint Institute, Shanghai, China*

*^2^University of Kansas Medical Center, Radiation Oncology, Kansas City, USA*

**Purpose:** Spot-scanning arc therapy (SPArc) is an emerging proton modality that can potentially offer a combination of advantages in plan quality and delivery efficiency, compared to IMPT with fixed beam directions. Unlike IMPT, frequent low-to-high energy layer switching (so called switch-up (SU)) can degrade delivery efficiency for SPArc. This work will consider the energy layer optimization (ELO) problem for SPArc and develop a new ELO method via energy matrix (EM) regularization to improve plan quality and delivery efficiency.

**Methods:** The major innovation of EM method for ELO is to design an energy matrix that encourages desirable energy-layer switching pattern with minimal SU during SPArc, and then incorporate this energy matrix into the SPArc treatment planning to simultaneously minimize the number of SU and optimize plan quality. The EM method is solved by the fast iterative shrinkage-thresholding algorithm and validated in comparison with a state-of-the-art method, so-called energy sequencing (ES).

**Results:** Regarding delivery efficiency, EM had fewer SU than ES (e.g., a 20% decrease of SU for abdomen). Regarding plan quality, compared to ES, EM had generally better target dose conformality (e.g., an increase in conformity index from 0.39 to 0.61 for HN), and better sparing of normal tissues (e.g., a 37% decrease for brainstem mean dose in brain) and lower integral dose for body (e.g., a 25% decrease for body mean dose in brain). Regarding computational efficiency, EM was substantially more efficient than ES, and took only about 1/10 of computational time.

### O 101 - A novel emittance selection system to maximize beam transmission for low-energy beams in cyclotron-based proton therapy facilities with gantry

#### Vivek Maradia^1,2^, David Meer^1^, Damien Charles Weber^1,3,4^, Antony John Lomax^1,2^, Jacobus Maarten Schippers^1^, Serena Psoroulas^1^

##### *^1^Paul Scherrer Institut, Center for Proton Therapy, Villigen, Switzerland*

*^2^ETH Zurich, Physics, Zurich, Switzerland*

*^3^University Hospital Zurich, Radiation Oncology, Zurich, Switzerland*

*^4^University Hospital Bern, Radiation Oncology, Bern, Switzerland*

In proton therapy, the potential of using high dose rates in cancer treatment is being explored. High dose rates could improve efficiency and throughput in standard clinical practice, allow efficient utilization of motion mitigation techniques for moving targets, and potentially enhance normal tissue sparing due to the FLASH effect. However, high dose rates are difficult to reach when lower energy beams are applied in cyclotron-based proton therapy facilities, because they result in large beam sizes and divergences downstream of the degrader, incurring large losses from the cyclotron to the patient. In current facilities the emittance after the degrader is reduced using circular collimators (emittance selection collimators after the energy degrader); this however does not provide an optimal matching to the acceptance of the following beamline, causing a low transmission for these energies. We, therefore, propose to use a collimation system asymmetric in both beam size and divergence, which allows maximizing the transmission of low energy beams, while resulting in symmetric emittance in both planes as required for a gantry system. Using a specifically designed asymmetric collimation system, we found in simulations that low energy (70 MeV) beam transmission increases almost 6 times compared to the currently used circular collimator system (Table 1)[1]. Such a system could easily be implemented in facilities interested in increasing dose rates for efficient motion mitigation and FLASH experiments alike. [1] Maradia et. al., “A new emittance selection system to maximize beam transmission for low-energy beams in cyclotron-based proton therapy facilities with gantry,” doi: https://doi.org/10.1002/mp.15278.

### O 102 - TURBO: A novel beam delivery system with fast energy switching for rapid depth scanning

#### Jacinta S. L. Yap^1^, Adam Steinberg^1,2,3^, Suzie Sheehy^1,4^

##### *^1^University of Melbourne, School of Physics, Melbourne, Australia*

*^2^University of Manchester, School of Physics and Astronomy, Manchester, United Kingdom*

*^3^Cockcroft Institute, Accelerator Science and Technology, Daresbury, United Kingdom*

*^4^Australian Nuclear Science and Technology Organisation ANSTO, Centre for Accelerator Science, Lucas Heights, Australia*

Charged particle therapy (CPT) has become a well-recognised therapeutic modality worldwide, where modern facilities can deliver high quality treatments with state-of-the-art technology such as active pencil beam scanning (PBS), rotational gantries, advanced imaging, and other clinical innovations. With new and emerging techniques such as FLASH and arc therapies, greater demands are placed on clinical beam delivery systems (BDS) and related technologies, requiring significant improvements. The beam delivery time (BDT) can account for a large amount of the total treatment time, where a limiting factor is the dead time to switch energies when irradiating repeated layers: the energy layer switching time (ELST). This is also driven by the BDS and its capability to transport and deliver beams quickly which impacts both treatment efficacy and efficiency. Improvements in the BDS could be achieved by having a large energy acceptance (LEA), enabling the transport of beams of varying energies with the same fixed magnetic field settings therefore reducing the ELST. Here we present the ‘Technology for Ultra Rapid Beam Operation' (TURBO) project in development at the University of Melbourne. Simulation and treatment planning studies will explore the system capabilities and benefits of a fixed-beamline, fast ELST solution. A scaled-down design with fixed-field alternating gradient (FFA) optics, a fast energy degrader and other features will be built for low energy hadrons, demonstrating the potential of a BDS for CPT with a LEA and rapid depth scanning for PBS delivery and other applications.

### O 103 - Improving proton IMPT delivery efficiency by optimizing the initial energy layer and spot placement parameters

#### Mingyao Zhu^1^, Katja Langen^1^, Stella Flampouri^1^, Alexander Stanforth^2^, Zachary Diamond^2^, William LePain^2^

##### *^1^Emory University School of Medicine- EPTC, Radiation Oncology, Atlanta, USA*

*^2^Emory Healthcare, Radiation Oncology, Atlanta, USA*

**Purpose**: Intensity modulated proton therapy (IMPT) plans provide high dosimetric quality but often result in long delivery time, which is typically not incorporated into the optimization process. The purpose of this study is to improve the delivery efficiency, without sacrifice plan quality, by optimizing the initial proton spot parameters.

**Methods**: Seven patients previously received IMPT in thorax and abdomen with breath-hold were included. In the clinical plans, the energy layer spacing (ELS) and spot spacing (SS) were set to the default values with a scale factor of 0.6-0.8. For each patient, we created four plans with ELS increased to 1.0, 1.2, 1.4 and SS to 1.0 while keeping all other parameters unchanged. All 35 plans (130 fields) were delivered on a clinical proton machine and the beam delivery time was recorded for each field.

**Results**: Compared to the clinical plans, increasing ELS and SS did not cause target coverage reduction (nominal or worst-case-scenario). Increasing ELS had no effect on critical organ-at-risk (OAR) doses or the integral dose, while increasing SS resulted in slightly higher integral and selected OAR doses (table1). Beam-on times were 48.4±9.2 [range: 34.1-66.7] seconds for the clinical plans. Time reductions were 9.2±3.3s (18.7±5.8%), 11.6±3.5s (23.1±5.9%), and 14.7±3.9s (28.9±6.1%) when ELS was changed to 1.0, 1.2, and 1.4, respectively, which correspond to 0.76-0.80 second/layer (figure1). SS change had minimal effect (1.1±1.6s, or 1.9±2.9%) on beam-on time.

**Conclusion**: Increasing the energy layers spacing can significantly reduce the beam delivery time without compromising IMPT plan quality.

### O 104 - Increased beam delivery accuracy with PBS by inversing beam energy sequence

#### Oxana Actis^1^, Oumeymah Cherkaoui^2^, Lena Nenoff^3^, Alexandre Mayor^1^, Francesca Albertini^1^, David Meer^1^, Antony John Lomax^1^, Damien Charles Weber^1^

##### *^1^Paul Scherrer Institute, Center for Proton Therapy, Villigen, Switzerland*

*^2^University of Basel, Department Biomedical Engineering DBE, Basel, Switzerland*

*^3^Massachusetts General Hospital, Department of Radiation Oncology, Boston, USA*

PBS proton therapy is an efficient cancer treatment technique requiring precise beam delivery. In gantries and along beam lines however, minimal variations in magnetic field strengths can result in beam position errors at the patient. If possible therefore, large beam energy changes, which could result in substantial hysteresis effects in the magnets, should be avoided or minimized. Conventionally, beam energies are applied in a descending sequence from high to low. In contrast, bi-directional energy sequences between 70 and 230 MeV were commissioned at PSI Gantry 2 [1], starting from descending energy scan **(D)**. However, due to human anatomy and optimized field directions, typical treatment energies range from 70 to 170 MeV. Therefore, we decided to reverse the energy sequence starting irradiation with ascending energy scan **(A)** in order to avoid large magnet changes at the beginning of the field. We delivered 22 patient plans for different tumor indications using **(D)** and **(A)** energy directions. Analyzing log files we calculated percentage of spots, and their corresponding dose, delivered with higher than 0.5 mm precision for each plan. In addition, we reconstructed the delivered dose distributions and compared them to the planed dose voxel by voxel. Sequence **(A)** plans showed better dose spot delivery precision (Table 1). The voxel-wise dose difference between planned and delivered dose with dose deviations >1% dropped by 22% using sequence **(A)** (Figure 1). Based on these results we clinically implemented the **(A)** energy sequence in March 2021. [1] https://iopscience.iop.org/article/10.1088/1742-6596/1067/9/092002

### O 105 - Evaluation of field disturbance by scanning magnet for MR-guided proton therapy

#### Nagaaki Kamiguchi^1^, Kenzo Sasai^2^, Yasuhiko Terada^3^, Tomoyuki Haishi^4^, Yukio Mikami^1^

##### *^1^Sumitomo Heavy Industries- Ltd., Technology Research Center, Yokosuka, Japan*

*^2^Sumitomo Heavy Industries- Ltd., Industrial Equipment Division, Tokyo, Japan*

*^3^University of Tsukuba, Faculty of pure and applied science, Ibaraki, Japan*

*^4^International University of Health and Welfare, Department of Radiological Sciences, Chiba, Japan*

The technique of tumor tracking with X-ray image has been developed in proton therapy due to importance of IGRT. Currently, linacs integrated with MRI were released and provide tumor tracking with MR images. MR image guide is expected to be helpful to track mobile targets like liver and pancreatic cancers that are difficult to be detected with X-ray image because MRI is superior to distinguish soft tissue. Our group has started considering the MR-guided PTS. When integrating MRI into PTS, the disturbance of main MR magnetic field by ferromagnets and current source in gantry and nozzle must be solved. Fast imaging sequences of MRI like SSFP method generally require homogeneous magnet field in order of a few μT. In this study, the influence of scanning magnets to main magnetic field was evaluated. The both yoke and exciting current of scanning magnets affected the order of 100μT to main field in case of active shield.Because the disturbance involved scanning magnet is not symmetry and the current is loaded dynamically, the shimming is not efficient. The passive shield method was considered so that the influence from outside of MRI is better to be isolated. Main field should be low enough, for example, 0.3T-0.5T to reduce shield weight and amount of bending angle. The effect of passive shield was confirmed with low field. The influence from outside of passive shield became negligible. As next steps, the design of shim, RF and gradient coils will be considered. After that, prototyping static field is planned.

### O 106 - Investigation of beam delivery time for a proton pencil beam scanning system with a novel scanning mode

#### Xiaoying Liang^1^, Chunbo Liu^1^, Keith Furutani^1^, Jiajian Shen^2^, Michele Dougherty^1^, Heng Li^3^, Martin Bues^2^, Alessio Parisi^1^, Deepak Shrestha^1^, Chris Beltran^1^

##### *^1^Mayo Clinic - Florida, Rad Onc, Jacksonville, USA*

*^2^Mayo Clinic - Arizona, Radiation Oncology, Phoenix, USA*

*^3^Johns Hopkins Medicine, Radiation Oncology, Washington DC, USA*

**Purpose**: To investigate proton pencil beam scanning (PBS) beam delivery time (BDT) using novel scanning modes.

**Methods**: A BDT calculation model was developed for the Hitachi ProBeat particle therapy system. The model was validated against the measured BDT of 36 representative clinical proton PBS plans with discrete spot scanning (DSS). BDTs was calculated using three different scanning modes: conventional DSS, and two novel scanning modes: dose driven continuous scanning (DDCS), and mixed scanning. In DDCS, the beam is not turned off between consecutive spots, but the beam current is lowered to control the transit dose in between spots. In mixed scanning, DDCS or DSS mode are applied depending on whether the spot delivery time is longer or shorter than the time it takes to transition from one spot to the next. The mixed scanning BDT as a function of 12 different threshold for DDCS/DSS was investigated.

**Results**: For 100% DDCS, the BDT was unacceptably long due to the low beam current to control the transient dose (Figure 1). Mixed scanning was able to maintain a reasonable beam current while controlling transient dose and achieved on average a 10% BDT reduction compared to DSS (Figure 2). The reduction of BDT with mixed scanning increased with the beam current threshold but reached a plateau for beam currents threshold larger than 10 MU/s.

**Conclusion**: One hundred percent DDCS uses low beam current to control the transient dose and does not improve BDT. Mixed scanning provides a good balance between dose fidelity and BDT.

### O 107 - An iterative resampling approach for CSI dynamic irradiation method

#### Lewei Zhao^1^, An Qin^1^, Gang Liu^2^, Shupeng Chen^1^, Xiaoqiang Li^1^, Weili Zheng^1^, Di Yan^1^, Rohan Deraniyagala^1^, Xuanfeng Ding^1^

##### *^1^Beaumont Health System, Proton Treatment Center, Royal Oak, USA*

*^2^Huazhong University of Science and Technology- Wuhan- PR China, Cancer Center- Union Hospital- Tongji Medical College, Wuhang, China*

**Purpose/Objectives:** The study introduces a new treatment modality of utilizing continuous couch movement in the treatment of CSI pediatric patient.

**Methods and methods:** For simplicity, disabled blind golfer mechanism (no burst) is considered for IBA ProteusONE^®^ compact superconducting synchrocyclotron proton therapy system. This study iteratively resamples the spots to control points in several sequences (same energy as original IMPT plan from high energy to low energy). The control point with the spot number greater than threshold will be split into two adjacent controls sharing the same energy. The control point with small spot number will be added additional dynamic to lower its velocity. A two-iso spine phantom is generated in a clinical treatment planning system (TPS) (RayStation version 6, Stockholm, Sweden) for testing. Total delivery time is calculated on the basis of a 20 cm couch moving distance. The initial control point distance within a sequence is 5mm. The couch moving velocity plot is created to examine its motion variation. Output plan is imported into RayStation for checking dose.

**Results:** The CSI dynamic irradiation method showed potential efficiency advantage. Compared with IMPT taking 5.11 ± 0.05 min on average to perform procedures for the 2^nd^ isocenter shift, this dynamic irradiation method takes 1.78 min total beam delivery time at a stable velocity (Figure 1). The plan quality is well preserved (Figure 2).

**Conclusion:** The algorithm introduced a new resampling mechanism to convert the original IMPT plan into a dynamic CSI treatment plan which allows the treatment to be delivered while couch is moving.

### O 108 - Magnetically focussed minibeams at a clinical proton linac

#### Tim Schneider^1^, Annalisa Patriarca^2^, Alberto Degiovanni^3^, Manuel Gallas^3^, Yolanda Prezado^1^

##### *^1^Institut Curie, Centre de recherche, Orsay, France*

*^2^Institut Curie, Centre de protonthérapie, Orsay, France*

*^3^Advanced Oncotherapy AVO, Application of Detectors and Accelerators to Medicine ADAM SA, Meyrin, Switzerland*

Proton minibeam radiation therapy (pMBRT) is a novel therapeutic strategy [1] that combines the tissue sparing potential of submillimetric, spatially fractionated beams (minibeams) with the improved ballistics of protons to enhance the tolerance of normal tissue and allow a dose escalation in the tumour. This could allow a more effective treatment of radioresistant tumours, as already demonstrated for rat gliomas [2,3]. To exploit the full potential of pMBRT, it should be delivered using magnetically focussed and scanned minibeams. We therefore recently proposed a new nozzle design optimised for the generation of such minibeams at clinically relevant energies [4]. In this study [5], we evaluated the performance of the minibeam nozzle in combination with the linear accelerator LIGHT developed by AVO-ADAM, the world's first linac for proton therapy [6]. Through Monte Carlo simulations performed with TOPAS [7], we could show the suitability of this combination for the delivery of clinically relevant, magnetically focussed minibeams (beam sizes 0.6-0.9 mm full width at half maximum) as well as conventional pencil beam scanning treatment plans. Moreover, our simulations showed a good robustness of the nozzle-linac combination against manufacturing, installation and operational uncertainties and beam current estimations indicate that FLASH dose rates (50-1500 Gy/s) could be obtained, both in PBS and minibeam mode. References*:* [1] Prezado et al., Med. Phys., 2013. [2] Prezado et al., IJROBP, 2019. [3] Bertho et al., Cancers, 2021. [4] Schneider et al., Sci. Rep., 2020. [5] Schneider et al. Cancers, 2021. [6] Degiovanni et al., IPAC 2018. [7] Perl et al., Med. Phys., 2012.

### O 109 - MonteRay: A fast Monte Carlo engine for helium ion dose calculation

#### Peter Lysakovski^1,2^, Benedikt Kopp^1^, Judith Besuglow^1,2,3,4^, Stewart Mein^1,3,4^, Thomas Tessonnier^1^, Alfredo Ferrari^1^, Thomas Haberer^1^, Jürgen Debus^1,3,4^, Andrea Mairani^1,4,5^

##### *^1^Heidelberg University Hospital, Heidelberg Ion-Beam Therapy Center- Department of Radiation Oncology, Heidelberg, Germany*

*^2^Heidelberg University, Faculty of Physics and Astronomy, Heidelberg, Germany*

*^3^National Center for Radiation Oncology NCRO, Division of Molecular and Translational Radiation Oncology- Heidelberg Institute of Radiation Oncology HIRO, Heidelberg, Germany*

*^4^German Cancer Research Center DKFZ, Translational Radiation Oncology- German Cancer Consortium DKTK- National Center for Tumor Diseases NCT, Heidelberg, Germany*

*^5^National Centre of Oncological Hadrontherapy CNAO, Medical Physics, Pavia, Italy*

In July 2021, the Heidelberg Ion-Beam Therapy Center treated the world-wide first patient with actively scanned helium ions. For treatment planning, an analytical dose calculation engine is used while independent plan verification is performed using the gold-standard Monte Carlo (MC) code FLUKA. While FLUKA provides accurate dose calculation, it is computationally demanding with runtimes longer than required by the pace of the clinic. For a clinically viable dose calculation engine, a simplified, specifically tailored modelling of physics must be used to meet time constraints while maintaining accurate dose estimates. The fast MC code MonteRay was recently developed for proton therapy, achieving good accuracy compared to other MC simulations and measurements. Here, MonteRay is extended to helium ion beams, including new models for complex nuclear interactions and fragmentation processes. The accuracy of MonteRay will be evaluated against measurements in homogenous and heterogeneous phantoms. Good agreement was found when comparing MonteRay simulations against measured depth dose distributions (Figure 1a) and lateral profiles (Figure 1b) of quasi-monoenergetic helium ions incident on water. Furthermore, MonteRay showed excellent agreement when compared against measured 2D dose distributions behind an anthropomorphic head-phantom (Figure 2), with computed 3%/3mm gamma-analysis yielding passing rates >98%. Timing comparisons with FLUKA have shown a speedup of ∼30 for helium ions. Comparisons with measurements and other MC simulations demonstrate MonteRay's accuracy and reliability in dose prediction. Extensive experimental verification of MonteRay is currently underway, paving the way for fast and independent patient verification for actively scanned helium therapy using a MC platform.

### O 110 - Superfast proton-ion dose engine based on Recurrent Neural Networks (SPIDER)

#### Ahmad Neishabouri^1^, Stewart Mein^2^, Jürgen Debus^3^, Andrea Mairani^2^

##### *^1^German Cancer Research Center DKFZ, Radiation Oncology, Heidelberg, Germany*

*^2^Heidelberg University Hospital, Heidelberg Ion-Beam Therapy Center, Heidelberg, Germany*

*^3^Heidelberg University Hospital, Radiation Oncology, Heidelberg, Germany*

A fast, end-to-end proton-ion dose calculation engine based on Recurrent Neural Networks (SPIDER) was designed, trained, and tested for the first time for proton treatments in the Heidelberg Ion-Beam Therapy (HIT) center. SPIDER is based on the previously published pencil-beam approach that produces ‘optimizable' biophysics output, i.e. dose influence matrix, allowing subsequent optimization of the pencil-beams fluence. Moreover, SPIDER can estimate three-dimensional pencil-beam dose distributions end-to-end, merely by providing the stopping power ratio distribution of the impinging protons' trajectory (Figure-1). For our supervised learning task, a total number of 35′000 single pencil-beams were sampled and the corresponding three-dimensional dose distributions were simulated using FLUKA Monte Carlo (MC) code. Simulations were carried out and sampled from a domain of 90 low-grade-glioma patients' cohort of the head-and-neck region, treated previously in HIT. Six patients were excluded from the training procedure and were set aside as ‘unseen' test cases. Figure-2 presents SPIDER's dose distributions for a representative test case, in comparison to the ground-truth FLUKA MC simulation. With accuracy comparable to FLUKA MC simulation (96.9% gamma-Index pass rate), SPIDER was able to carry out the full-patient dose calculation task under 77 seconds (desktop workstation; Nvidia RTX-6000). The favorable trade-off between speed and accuracy in SPIDER can pave the way for realizing a real-time dose calculation engine in adaptive proton therapy. Moreover, SPIDER can provide immediate three-dimensional dose distributions for decision support purposes, automated treatment planning, and uncertainty quantification calculations in clinical workflows. To achieve sub-second runtimes, further research in optimizing pencil-beam cubes' pre/post-processing is warranted.

### O 111 - Implementation of an octree-based CT Compression for GPU-based Monte Carlo dose calculations

#### Costanza Panaino^1^, Hok Seum Wan Chan Tseung^1^

##### ^1^Mayo Clinic, Radiation Oncology, Rochester, USA

For proton therapy (PT) dose calculations, the Monte Carlo technique is the gold standard in terms of accuracy. At our institution a fast GPU-based Monte Carlo (GPUMC) dose engine has been developed and clinically deployed [1]. We report on the use of octree compression (OC) of CT images for speeding up the GPUMC. Using a fast GPU-based OC algorithm, CT voxels with material-to-water stopping power ratio values within 3-5% are merged. HU values of voxels outside the external contour were overridden to air. Dose is scored only within selected structures (e.g. CTV) using the native CT voxel size, and computational times are recorded. Comparisons are made with the no-OC case. With OC, protons can be propagated with larger step sizes outside of structures requiring dose scoring. The compressed CT and CTV dose are demonstrated in Fig.1 for prostate, liver, and head-and-neck cases. The prostate example is reported for 3 and 5% OC. Fig.2 shows the dose difference distributions (fitted to Gaussians) between the native and the 3% OC voxelization cases, d_nativeCT _– d_OC-CT_, within the CTV for the same sites. When scoring CTV dose only, the time gain with the 3% OC voxelization is 11%, 12%, 30% for prostate, liver, and head-and-neck, respectively. To our knowledge this is the first attempt to introduce OC in GPUMC simulations. The OC was able to decrease the computational time while maintaining the same dosimetric accuracy in the CTV. [1] Wan Chan Tseung H, *et al*. Med. Phys. 42(2015)2967.

### O 112 - Dose and dose rate optimization for FLASH proton therapy with automated Bragg peak and shoot-through beam selection

#### Pavitra Ramesh^1^, Wenbo Gu^2^, Dan Ruan^1^, Ke Sheng^1^

##### *^1^University of California Los Angeles, Radiation Oncology, Los Angeles, USA*

*^2^University of Pennsylvania, Radiation Oncology, Philadelphia, USA*

The combined use of Bragg peak and shoot-through beams at ultra-high dose rates has been shown to reduce normal-tissue toxicities while maintaining dose conformality. Both physical dose and the FLASH effect depend on the beam orientation selection, but the joint optimization problem for all three elements has neither been proposed nor solved. To maximize the proton FLASH effect, here, we incorporate dose rate objectives into our beam orientation optimization (BOO) framework. From our previously developed group-sparsity dose objectives, we add upper and lower dose rate terms using the dose-averaged dose rate (DADR) definition and solve using the fast-iterative shrinking threshold algorithm. We compare the dosimetry for three head-and-neck cases between four techniques: 1) Bragg peak (BP) 2) Bragg peak with dose rate optimization (BP-DR), 3) shoot-through with dose rate optimization (ST-DR), and 4) combined BP and ST (BPST-DR), with the goal of sparing OARs without loss of tumor coverage and maintaining high dose rate within a 10mm region of interest (ROI) surrounding the CTV. For [BP, BP-DR, ST-DR, BPST-DR], CTV HI and Dmax were found to be on average [0.934, 0.950, 0.670, 0.894] and [100%, 99.2%, 134%, 116%] of prescription, respectively. Mean body dose was [0.82, 0.96, 2.1, 1.4] Gy. Volume of ROIs receiving greater than 40 Gy/s was [57.5%, 92.3%, 97.2%, 98.1%] on average. The dose rate techniques, particularly BPST-DR, were able to significantly increase dose rate without compromising physical dose. Our algorithm efficiently selects beams that are optimal for both dose and dose rate.

### O 113 - A novel approach for obtaining Monte Carlo computed dose and variable proton RBE from a treatment planning system

#### Johannes Tjelta^1^, Lars Fredrik Fjæra^2^, Kristian S Ytre-Hauge^2^, Camilla Boer^1^, Camilla Hanquist Stokkevåg^1^

##### *^1^Haukeland university hospital, Department of Oncology and Medical Physics, Bergen, Norway*

*^2^University of Bergen, Department of Physics and Technology-, Bergen, Norway*

**Background**: A constant relative biological effectiveness (RBE) is employed during clinical proton therapy, although RBE varies across volume, tissue, LET and fractionation. This work provides a generalized method for calibrating a Monte Carlo (MC) code to a treatment planning system (TPS) to obtain parameters for calculating LET and variable RBE in proton treatment plans (Figure1).

**Methods**: Pristine Bragg peaks (BPs) were calculated in Eclipse TPS (ProBeam360 data) and FLUKA MC code. A rearranged Bortfeld energy-range relation was applied to the initial energy of the beam to fine-tune the range of the MC at 80% range distal to BP. We tuned the energy spread by using its relation to 80%-20% distal range of the BPs. Density and relative proton stopping power were adjusted in the MC code to correspond with the TPS. To find the relationship of dose per particle from the MC to dose per monitor unit in the TPS, trapezoidal integration was applied. The calibration was validated in a spread-out BP (SOBP) in water and pediatric treatment plans, where RBE-weighted/LET models were calculated.

**Results**: For different depths, the BPs were within ±0.5mm (Figure1) and a max dose difference of 3% for SOBPs. The patient validation showed good agreement between the TPS and MC (Figure2), with varying doses from the RBE-weighted/biological dose, calculated with the McNamara model (Figure1).

**Conclusion**: A procedure for calibrating a MC code to a specific TPS was presented and achieved excellent agreement between TPS and the MC. The procedure enables MC-based calculation of dose, LET and variable RBE from proton treatment plans.

### O 114 - Microdosimetry with a tissue-equivalent proportional counter at the MedAustron light ion beam therapy facility

#### Sandra Barna^1^, Cynthia Meouchi^2^, Giulio Magrin^3^, Anna Bianchi^4^, Valeria Conte^4^, Anna Selva^4^, Markus Stock^3^, Andreas Franz Resch^1^, Dietmar Georg^1^, Hugo Palmans^3^

##### *^1^Medizinische Universität Wien, Universitätsklinik für Radioonkologie, Vienna, Austria*

*^2^Technische Universität Wien, Atominstitut, Vienna, Austria*

*^3^MedAustron Ion Therapy Center, Medical Physics, Wr. Neustadt, Austria*

*^4^Istituto Nazionale di Fisica Nucleare, Laboratori Nazionali di Legnaro, Legnaro, Italy*

The aim is to establish an experimental procedure for the microdosimetric characterization of the radiation quality of ion beams in the specific clinical environment of the MedAustron therapy facility. The first microdosimetric spectra measured with a gas detector at the proton beams of MedAustron are presented. A miniaturized tissue-equivalent proportional counter (mini-TEPC) was used to collect microdosimetric spectra at different positions along the depth dose profile of a monoenergetic 62.4 MeV single-spot proton beam with a full width at half maximum of approximately 2.5 cm. A reduced particle rate of approximately 4 MHz was maintained to minimize pile-up and saturation effects. The mini-TEPC has a cylindrical sensitive volume with equal diameter and height of 1 mm and is filled with low pressure propane gas to simulate a size of 1 μm at unit density. The spectra obtained for the MedAustron beam were compared to those measured at other facilities at approximately the same nominal beam energy [1]. The comparison was performed at similar positions along the depth dose curve. Additionally, Monte Carlo simulations have also been benchmarked with experimental data. In general, experimental spectra measured at different centres with approximately the same nominal beam energy show very similar shapes in the distal region of the depth dose profile. In contrast, some deviations can be seen in the entrance region, potentially reflecting differences in the radiation fields and/or detectors used. [1] V. Conte et al., Microdosimetry at the CATANA 62 MeV proton beam with a sealed miniaturized TEPC, Physica Medica 64 (2019) 114–122

### O 115 - A shielding design for the first U.S. carbon multi-ion hybrid synchrotron facility

#### Jingjing Dougherty^1^, David Bolst^2^, Susanna Guatelli^2^, Anatoly Rosenfeld^2^, Chris Beltran^1^

##### *^1^Mayo Clinic, Radiation Oncology, Jacksonville, USA*

*^2^University of Wollongong, Centre for Medical Radiation Physics, Wollongong, Australia*

To present a comprehensive shielding design for the first carbon-ion hybrid-synchrotron facility in the US. The Mayo Clinic Florida Integrated Oncology Building (IOB) will be the home of the first carbon/proton hybrid therapy system by Hitachi, Ltd. It will provide proton beams up to kinetic energies of 230 MeV and carbon beams up to 430 MeV/n for clinical treatment. To provide adequate radiation protection, the Geant4 (v10.6) Monte Carlo toolkit was utilized to quantify the ambient dose equivalent at a 10 mm depth (H*(10) for photons and neutrons. The scoring ICRU soft tissue sphere was 30 cm in diameter. 3D computer-aided design files of the entire planned facility were imported into Geant4 using the STL format. Furthermore, various beamline and system components (bending magnets, FFC, gantry, to name a few) were imported into Geant4 to simulate realistic neutron interactions and self-shielding effects. The Glauber Griboy (GG) model was chosen to determine the elastic and inelastic cross-sections. For inelastic interactions, the conservative Bertini cascade model was chosen to model the secondary particle interactions. We have optimized each shielding slab thickness through iterative processes with more than 20 CAD file designs. The 430 MeV/n carbon beams play the most significant role in concrete thickness calculation. The primary wall thickness for the carbon fixed beam room is 4 meters. All shielding goals were met. Leveraging 3D architectural renderings, particle therapy system components, and Geat4 Monte Carlo simulation techniques, the IOB facility meets all shielding goals mandated by the state regulations for clinical operations in 2025.

### O 116 - MedAustron commissioning experience for an active scanning proton gantry beam line

#### Alessio Elia^1^, Jhonnatan Osorio Moreno^1^, Ralf Dreindl^1^, Marta Bolsa Ferruz^1^, Patrick Roisl^1^, Markus Stock^1^, Loïc Grevillot^1^

##### ^1^EBG MedAustron GmbH, Medical Physics, Wiener Neustadt, Austria

**Introduction:** The MedAustron Ion Therapy Centre (Wiener Neustadt, Austria) is a dual particle synchrotron-based facility which has been equipped with an active scanning proton gantry beside its three fixed beam line rooms. For Treatment Planning System (TPS) commissioning purposes, it is of paramount importance to establish a stable beam delivery among different gantry angles. In this work, we report on the assessed dosimetric capabilities for the gantry beam line.

**Material and Methods:** a unique optics rotator system was installed in the high energy beam transfer line so that similar optical properties were set for each gantry angle. The proton pencil beam was then characterized over one-dimensional, two dimensional and three-dimensional delivery mostly by means of isocentric measurements. Lynx (IBA-dosimetry) and peakfinder (PTW, Freiburg) devices were exploited for FWHM and ranges acquisitions, respectively. For dose measurements, a RW3 plastic phantom equipped with an Ionization Chamber Roos-type was mounted directly at the gantry nozzle. The three-dimensional dose delivery was characterized in water in an independent study. A range shifter (RaShi) placed at different air gaps was also taken into consideration.

**Results:** The rotator system ensured FWHM variations among different gantry angles within 3.5% (see Figure 1). Similar results were obtained when the RaShi was in place. Ranges were always within 0.1mm from specifications. Dose was found within 1.5% from reference conditions (see Figure 2).

**Conclusion:** A stable and angle-independent beam delivery is found at every commissioned gantry angle. The MedAustron gantry room will be ready for patient treatments in the second quarter of 2022.

### O 117 - The new Particle Therapy Research Center (PARTREC) at UMCG Groningen: Performance, infrastructure, treatment modalities and timeline of further upgrades

#### Alexander Gerbershagen^1^, Lara Barazzuol^2^, Sytze Brandenburg^1^, Rob Coppes^2^, Peter Dendooven^2^, Marc-Jan van Goethem^1^, Emil van der Graaf^1^, Peter van Luijk^2^, Jacobus Maarten Schippers^1^, Stefan Both^1^

##### *^1^University Medical Center Groningen - University of Groningen, Particle Therapy Research Center PARTREC- Department of Radiation Oncology, Groningen, Netherlands*

*^2^University Medical Center Groningen - University of Groningen, Department of Biomedical Sciences of Cells and Systems, Groningen, Netherlands*

PARticle Therapy REsearch Center (PARTREC) is a newly established research center in Groningen, the Netherlands. It utilizes the superconducting cyclotron AGOR, previously successfully used at KVI-CART for 25 years for research in the nuclear and radiation physics domains. AGOR provides the possibility to deliver highly customized beams of protons and a large variety of ion types (currently from He to Xe, and an upgrade for heavier ions is envisaged). Upon integration in the University Medical Center Groningen, PARTREC is used for research in experimental particle therapy physics, medical physics, biology and radiotherapy, such as LET and RBE studies for biological treatment planning, studies using various tumour and normal tissue *in vitro* and *in vivo* models, and emerging technologies such as FLASH, proton radiography and short lived isotopes PET. In addition, a new research infrastructure for image-guided preclinical irradiations (IMPACT) has been assembled, including an advanced small animal irradiation facility. The close cooperation with the clinical Groningen Proton Therapy Center enables fast bed to bench and back research, aiding the therapeutic window optimization. PARTREC continues to welcome international academic and industrial collaborators since its establishment in 2020.

### O 118 - Spot-scanning Hadron Arc (SHArc) Therapy: development of single and multi-particle arc delivery using protons, helium, carbon, oxygen and neon ions

#### Stewart Mein^1^, Thomas Tessonnier^1^, Benedikt Kopp^1^, Christian Schömers^2^, Jakob Liermann^3^, Semi Harrabi^3^, Jürgen Debus^3^, Amir Abdollahi^3^, Thomas Haberer^2^, Andrea Mairani^1^

##### *^1^Heidelberg Ion-beam Therapy Center HIT, Biophysics in Particle Therapy BioPT, Heidelberg, Germany*

*^2^Heidelberg Ion-beam Therapy Center HIT, Physics, Heidelberg, Germany*

*^3^Heidelberg Ion-beam Therapy Center HIT, Radiation Oncology, Heidelberg, Germany*

**Purpose:** At the Heidelberg Ion-beam Therapy Center (HIT), novel single and multi-ion treatment strategies are under development and investigation using protons (p), helium (4He), carbon (12C), oxygen (16O) and/or neon (20Ne) ion beams. Here, we present spot-scanning hadron arc (SHArc) therapy, the first arc delivery technique for light and heavy ions.

**Materials/Methods:** Treatment design and optimization procedures for SHArc were developed for efficient and robust delivery (Fig 1a). SHArc treatments were optimized for various clinical indications using single (p, 4He, 12C, 16O, or 20Ne) and multi-ion (12C+4He, 16O+4He or 20Ne+4He) strategies: glioblastoma, chordoma, prostate carcinoma, pancreatic adenocarcinoma, and non-small cell lung carcinoma. Dosimetric and biophysical features were evaluated. A model for hypoxia-induced radio-resistance was developed to investigate impact of LET redistribution. Treatment times for step-and-shoot delivery were estimated

**Results:** SHArc plans exhibited a low-dose bath outside the target volume with a reduced maximum dose in normal tissues compared to conventional planning. Substantial increases in min and max LET_GTV_ were possible with SHArc. Multi-ion arc plans achieved similar distributions with highly homogenous physical dose and RBE in the target volume (Fig 1b). Estimated delivery time ranged from 6-15 minutes.

**Conclusion:** The first arc treatment delivery technique for light and heavy ions beams is proposed. Results suggest that SHArc and multi-ion therapy (MIT) may offer unique clinical advantages. SHArc treatments could potenitally improve local control by mitigating impact of radio-resistance factors such as tumor hypoxia.

### O 119 - Configuring a flexible beam line for in-vivo/in-vitro FLASH proton radiation therapy treatments

#### Rachael Hachadorian^1^, Ethan Cascio^1^, Thomas Ruggieri^1^, Marc Bussiere^1^, Jan Schuemann^1^

##### ^1^Harvard Medical School - Massachusetts General Hospital, Radiation Oncology, Boston, USA

This study configures a more efficient, double-scattering, Bragg peak FLASH beamline that is capable of delivering both conventional and FLASH treatments *in vitro* and *in vivo*. The beam spot size and spread was measured with film and then implemented into and confirmed in TOPAS (TOol for PArticle Simulation), (Fig 1a,b). Using TOPAS, Monte Carlo simulations were run and optimized to verify the ideal positioning, dimensions, and material type of the scattering foil, second scatterer, ridge filter, range compensator, and aperture (Fig. 1c). Our in-house experimental FLASH beamline will be used to verify these simulations. The configuration and throw which produced the largest field size of acceptable flatness, without drastically compromising dose rate was determined to be an oval field of up to 2 cm x 1.5 cm. The shoot-through dose rate was measured to be greater than 100 Gy/s. A spread out Bragg peak (SOBP) was generated using a ridge filter of three discrete heights and experimentally-verified weights, yielding three distinct but connected spikes in dose with flatness under 5% (Fig. 1d, Fig. 2a-g). Reducing the width of the scattering foil by a factor of two was found to increase efficiency by 48% (Fig 1e). We have experimentally established FLASH dose rates at our proton beamline for several beneficial configurations, and achieved dose rates >100 Gy/s using the shoot-through technique. Experimentally, we anticipate the maintenance of FLASH dose rates for SOBP irradiations with larger field sizes and confirmed profilometry for a flexible small animal proton FLASH irradiator.

### O 120 - Modeling radiation dose to circulating lymphocytes: the impact of ultra-high-dose rate (FLASH) radiotherapy and fractionated, conventional dose rate exposure

#### Abdelkhalek Hammi ^1^

##### ^1^TU Dortmund University, Physics, Dortmund, Germany

**Purpose:** To evaluate the potential impact of ultra-high-dose rate of intensity-modulated proton therapy (IMPT) radiotherapy FLASH-RT on radiation dose received from circulating lymphocytes (CL).

**Materials and methods:** Two independent dynamic models were implemented: one describing the spatially varying instantaneous dose-rate of radiation field based on parameters of a commercially available proton system and a second describing the spatiotemporal distribution of blood particles (BPs) based on ICRP hemodynamics references. For the brain, arterial branches and bifurcations were quantified from MRA data of 10 subjects. The whole-body blood counts 50*10^6 ^BPs. A discrete-time Markov chain was used to model the probability of BPs to traverse bifurcations.Single-fraction (50Gy×1Fx) and two different hypofractionated FLASH-RT plans (25Gy×2Fx; 10Gy×10Fx) were simulated using beam intensity of I=300nA and a minimum spot-weight of 0.4*10^6^ protons. The FLASH-RT deliveries were compared to fractionated (2Gy) treatments scenarios (IMPT and photons). The effects of treatment regimes on CL depletion were investigated.

**Results:** The irradiated blood volume (V>_0Gy_) after entire treatment increases from 1.5% for single-fraction FLASH-RT to 3.1%, 42.4% and 99.4% for hypofractionated FLASH-RT, IMPT and photons, respectively. For dose higher than 1Gy, FLASH-RT irradiates more circulating blood than fractionated treatment regimes, while D_2%_ is high for FLASH-RT and decreases with the number of fractions. FLASH-RT reduces the depletion of CL by a factor of 12.7.

**Conclusion:** We present a first attempt to assess the adverse effect of FLASH-RT on the immune system. FLASH-RT spares CL in comparison to fractionated regimes. The number of fractions impacts the radiation dose to CL.

### O 121 - A novel ultra-high dose rate proton therapy technology: FLASH spot-scanning proton arc therapy (SPLASH)

#### Gang Liu^1^, Lewei Zhao^2^, Xiaoqiang Li^2^, Zhang Sheng^1^, Shuyang Dai^3^, Xiliang Lu^3^, Ding Xuanfeng^2^

##### *^1^Huazhong University of Science and Technology, Cancer Center-Union Hospital-Tongji Medical College, Wuhan, China*

*^2^Beaumont Health System, Department of Radiation Oncology, Royal Oak, USA*

*^3^Wuhan University China, School of Mathematics and Statistics, Wuhan, China*

**Purpose:**The flash dose rate on the order of 40 Gy/s has the potential to spare normal tissue with iso-effective tumor growth delay. Meanwhile, the spot-scanning proton arc therapy(SPArc), demonstrated its superior dosimetric conformity and plan robustness compared to the conventional intensity modulated proton therapy(IMPT). To take the full advantage of flash dose rate and the high dose conformity, we introduce a novel optimization and delivery technique, FLASH-SPArc (SPLASH).

**Methods:**SPLASH framework was implemented in an open-source proton planning platform (matRad). It optimizes the spot weighting and cyclotron beam current per spot simultaneously. More specifically, it simultaneously optimized with the clinical dose-volume constraint based on dose distribution and optimized the dose-average dose rate by minimizing the constraint on spot weighting and maximum cyclotron beam current to minimize the cost function value combined by dose and dose rate. A intracranial target case with prescription(50Gy/25F) was used for testing purpose. Dose-volume histogram and dose averaged dose rate histogram were compared between SPArc and SPLASH.

**Results:**The dose-averaged dose rate histogram indicated that flash dose rate (40Gy/s) could not be achieved using the original SPArc with the current clinical beam current configuration. With a same plan quality, SPLASH improved dose-averaged dose rate of V*r*>40Gy/s in PTV, optic chiasm, optic nerve and brainstem to 100%,100%,100%,75%, respectively.The optimal cyclotron beam current per spot is simultaneously generated(Fig1).

**Conclusion:**The SPLASH offers simultaneously optimizing dose distribution and dose rate. It could generate a treatment plan with a superior conformal dose distribution and ultra-high dose rate, which has never been demonstrated before.

### O 122 - Feasibility of proton SBRT FLASH treatment with dose, dose rate, and LET optimization using patient specific 3D ridge

#### Ruirui Liu^1^, Serdar Charyyev^1^, Niklas Wahl^2^, Wei Liu^3^, Jun Zhou^1^, Xiaofeng Yang^1^, Kristin Higgins^1^, Jeffrey Bradley^1^, Liyong Lin^1^

##### *^1^Emory University, Radiation Oncology, Atlanta, USA*

*^2^German Cancer Research Center – DKFZ, Department of Medical Physics in Radiation Oncology, Heidelberg, Germany*

*^3^Mayo Clinic College of Medicine, Radiation Oncology, Phoenix, USA*

**Purpose:** Patient specific 3D ridge can cover SBRT target with IMPT-like dose in one single field without multi-fields. By removal of proton energy switching time, it can potentially achieve FLASH timing. We investigate the feasibility of proton SBRT FLASH treatment with dose, dose rate, and LET optimization using 3D ridge filter.

**Methods:** An in-house inverse planning software is developed to design the patient-specific ridge filter which is used to spread fixed 250 MeV beam to proximal beam-specific planning target volume (BSPTV) to ensure IMPT plan robustness (Fig 1c) . Ridge pin positions are defined in beam eye view (BEV) of BSPTV. We developed an in-house interface to Geant4, which provides Monte Carlo calculated dose and LET influence matrixes and modify matRad optimization to accommodate integrated biological optimization IMPT (IBOIMPT) objective function, considering simultaneous dose, dose rate, and LET terms. Minimum MU constraint was implemented. Dose averaged dose rate (DADR) and dose averaged LET (LETd) of a lung case were improved with similar target coverage.

**Results:** Fig 2a shows the dose, DADR, and LETd for the IMPT plan and IBOIMPT plan with ridge filter. Both plans meet the clinical dose constraints. However, IBOIMPT better spared heart that has marginal risk by improved DADR > 40 Gy/s and reduced LETd. The dose coverage of SBRT target and low lung dose were maintained for in IBOIMPT as IMPT.

**Conclusion:** Our preliminary data shows that it is feasible to achieve in SBRT more FLASH and reduced LET for organs at risk in IBOIMPT than IMPT.

### O 123 - Adaptation and dosimetric commissioning of a synchrotron-based proton beamline for FLASH experiments

#### Ming Yang^1^, Xiaochun Wang^1^, Fada Guan^2^, Uwe Titt^1^, Kiminori Iga^3^, Dadi Jiang^4^, Tadashi Katayose^5^, Masumi Umezawa^5^, Steven Frank^4^, Ronald Zhu^1^

##### *^1^UT MD Anderson Cancer Center, Radiation Physics, Houston, USA*

*^2^Yale University School of Medicine, Therapeutic Radiology, New Haven, USA*

*^3^Hitachi America Ltd., Particle Therapy Division, Houston, USA*

*^4^UT MD Anderson Cancer Center, Radiation Oncology, Houston, USA*

*^5^Hitachi Ltd., Smart Life Business Management, Tokyo, Japan*

The goal of this study was to (1) achieve FLASH irradiation conditions suitable for pre-clinical biology experiments using our synchrotron-based proton beamline and (2) commission the FLASH irradiation conditions achieved. To achieve FLASH conditions, we made a series of adaptations to our proton beamline, including modifying the spill length and size of accelerating cycles, repurposing the reference monitor for dose control, and expanding the field size with a custom double-scattering system. We performed the dosimetric commissioning with measurements using an Advanced Markus chamber (AMC) and EBT-XD films as well as with Monte Carlo simulations. Through adaptations, we have successfully achieved FLASH conditions, with an average dose rate of up to 375 Gy/s. The AMC was shown to be appropriate for absolute dose calibration under our FLASH conditions with a recombination factor ranging from 1.002 to 1.006 because of the continuous nature of our proton beams. In addition, the absolute dose measured using the AMC and EBT-XD films agreed well, with average and maximum differences of 0.32% and 1.63%, respectively. We also performed a comprehensive temporal analysis for FLASH spills produced by our system, which helped us identify a unique relationship between the average dose rate and the dose in our FLASH irradiation. In summary, we have established a synchrotron-based proton FLASH irradiation platform with accurate and precise dosimetry that is suitable for pre-clinical biology experiments. The unique time structure of the FLASH irradiation produced by our synchrotron-based system may shed new light onto the mechanism behind the FLASH effect.

### O 124 - A novel daily quality assurance pattern for proton pencil-beam scanning FLASH radiotherapy

#### Yunjie Yang^1^, Chin-Cheng Chen^1^, Pingfang Tsai^1^, Jiayi Liu^1^, Minglei Kang^1^, Haibo Lin^1^

##### ^1^New York Proton Center, Medical Physics, New York, USA

**Purpose:** A QA pattern was designed and irradiated on a high spatiotemporal resolution 2D strip ionization chamber array (SICA) to assess the beam performance of scanning proton beams under ultra-high dose rates (UHDRs).

**Methods:** A 135×135 mm^2^ daily QA pattern with a central 50×50 mm^2^ field and 4 asymmetrical lines along the perimeter was irradiated on the SICA with 250 MeV proton beam at nozzle currents of 100 nA and 215 nA, respectively. An advanced Markus ionization chamber and a Faraday cup were used concurrently. The spot scanning speed, spot sizes and positions, central field flatness and symmetry, and average dose rates were obtained in a single-shot to characterize the beam performance. Daily QA using designed pattern produced 64 measurements and the consistencies of beam performance were analyzed.

**Results:** Compared to the mean values of the measurements, the deviations of the spot scanning speed were less than 12 %. The deviations of the spot sizes were <4 % and the deviations of the relative spot positions were <0.2 mm. The consistency of the central field flatness and symmetry are <1.0% and <0.5%. The average dose rate at isocenter was 53.1±4.5% Gy/s and 114.4±4.2% Gy/s, and more than 75% and 95% of the measured points reached the FLASH threshold (40 Gy/s) at 100 nA and 215 nA, respectively.

**Conclusions:** Less than 5% deviation of the absolute dose rate can be achieved for the current beam delivery system. The QA process demonstrated the designed pattern can be used to assure the UHDR beam performance.

### O 125 - Oxygen depletion for FLASH: key considerations to make or break the effect

#### Bethany Rothwell^1^, Matthew Lowe^1,2^, Norman Kirkby^1,3^, Michael Merchant^1,3^, Amy Chadwick^1,3^, Ranald Mackay^1,2^, Karen Kirkby^1,3^

##### *^1^University of Manchester, Division of Cancer Sciences, Manchester, United Kingdom*

*^2^The Christie NHS Foundation Trust, Christie Medical Physics and Engineering, Manchester, United Kingdom*

*^3^The Christie NHS Foundation Trust, Manchester Academic Health Science Centre, Manchester, United Kingdom*

FLASH radiotherapy is rapidly advancing towards clinical translation. However, the mechanisms behind the effect remain poorly understood. Amongst those proposed, oxygen depletion stands out as the most prevalent, but also the most disputed. Many studies have shown an oxygen-dependent sparing effect for FLASH, suggesting its depletion to be a likely contributing factor, yet others have demonstrated insufficient depletion levels to explain the phenomenon. In this work we have used a computational model of oxygen diffusion and reaction in tissue to assess the extent to which variations in experimental conditions could lead to these different conclusions. We have also addressed several common assumptions in oxygen depletion models, and incorporated additional biological processes, to determine whether this could bring oxygen depletion into the realm of feasibility as a mechanism for FLASH. Results show that a FLASH-induced change in radiosensitivity is particularly sensitive to the rate constant for oxygen depletion. Using depletion rate measurements (oxygen consumed per Gy of radiation absorbed) across the literature under different experimental conditions, our results show that the change in radiosensitivity from ultra-high-dose-rate radiation can increase 4-fold for measurements in cell-like solutions compared to those in water (see Figure 1). Furthermore, our results show that changes in radiosensitivity caused by a given level of oxygen depletion can be significantly affected by small changes in cellular metabolic demand and perturbations in the blood supply during irradiation. Our work suggests more understanding is needed before oxygen depletion can firmly be taken off the table as a key mechanism behind FLASH.

### O 126 - SOBP ultra-high dose rate irradiation at a VMS ProBeam using a 3D printed range modulator

#### Yuri Simeonov^1^, Petar Penchev^1^, Per Poulsen^2^, Mateusz Sitarz^2^, Simon Busold^3^, Christoph Schuy^4^, Miriam Krieger^3^, Michael Folkerts^5^, Klemens Zink^1^, Uli Weber^4^

##### *^1^THM, Institute for Medical Physics and Radiation Protection, Giessen, Germany*

*^2^Danish Center for Particle Therapy DCPT, Danish Center for Particle Therapy DCPT, Aarhus, Denmark*

*^3^Varian Medical Systems Particle Therapy, Varian Medical Systems Particle Therapy, Troisdorf, Germany*

*^4^GSI Helmholtzzentrum für Schwerionenforschung, Department of Biophysics, Darmstadt, Germany*

*^5^Varian Medical Systems, Varian Medical Systems, Palo Alto, USA*

There has been increased interest in FLASH particle therapy recently. Several studies suggest that a sub-second irradiation with ultra-high dose rates over 40 Gy/s may reduce toxicities and increase the therapeutic window. Presently, the major manufacturers of particle therapy facilities are trying to increase their accelerators' intensity and improve the dose delivery and monitoring systems to enable FLASH therapy. When comparing the different commercially available delivery techniques, a multi iso-energy irradiation is too slow due to the time needed to actively change the primary beam energy. Range modulators in combination with only one energy and a laterally scanned beam offers the opportunity for tumor conformal and extremely fast FLASH-IMPT treatments. Recently a SOBP ultra-high dose rate irradiation with a Varian ProBeam facility was performed using a range modulator. The modulator was designed to irradiate a cuboid-shaped target volume with dimensions 3x3x5 cm^3^, i.e. the width of the SOBP was 5 cm and was centered at a depth of 13.5 cm in water. The applied dose was 8 Gy, modeling dose rate [1] in Eclipse showed >40Gy/s to 95% of the entrance region. The energy of the primary proton beam was 250 MeV. To shift the SOBP to the mentioned depth, 19 cm PMMA were placed in front of the modulator. A comparison of measured and Monte Carlo based dose distributions is given in Fig.1, the reconstructed dose rate in Fig.2. This study demonstrates the feasibility of SOBP-FLASH on a Varian ProBeam system with the use of customizable 3D-range modulators. [1]:https://doi.org/10.1002/mp.14456.

### O 127 - Multi-site treatment planning comparison utilizing electrons, VMAT, IMPT and proton ConformalFLASH

#### Andrew Wroe^1^, Alonso Gutierrez^1^, Lucian Hotoiu^2^, Nicole McAllister^1^, Zachary Fellows^1^, Carolina Fuentes^2^, Noah Kalman^1^, Michael Kasper^3^, Michael Chuong^1^, Minesh Mehta^1^

##### *^1^Miami Cancer Institute, Radiation Oncology, Miami, USA*

*^2^Ion Beam Applications IBA S.A., R&D- Clinical Solutions Group, Louvain-la-Neuve, Belgium*

*^3^Lynn Cancer Institute, Radiation Oncology, Boca Raton, USA*

**Purpose**: Perform a treatment-planning study to identify the treatment sites that show the greatest dosimetric benefit to proton ConformalFLASH delivery.

**Methods and materials**: Clinical target sites investigated as part of this study include skin, sarcoma, lung, CNS, head & neck, and abdomen with 3-4 subjects evaluated per site. ConformalFLASH plans were generated using the UCLouvain/IBA-developed MIROpt FLASH TPS with resultant dose and dose-rate distributions visualized in RayStation. Comparative electron, VMAT and IMPT plans were generated using Eclipse or RayStation treatment planning systems in current clinical use. Dose distributions, target coverage and OAR dose metrics were evaluated within RayStation for both clinical acceptability and inter-modality comparison.

**Results**: A preliminary temple case is presented in figure 1 which has been planned with both ConformalFLASH and electrons. Both plans were scaled such that 98% of the GTV received 35Gy. Dosimetrically, the proton plan has a steeper penumbra and distal fall off than the electron plan allowing for sparing of the eye, lacrimal gland and brain (figure 2). These characteristics are most likely a direct result of the aperture and low proton energy, and further case analysis is needed to determine if this will hold true for deeper seated targets. It is clear from this case that the proton FLASH plan is dosimetrically comparable to the clinical electron plan, while providing the potential radiobiological benefits of very high dose rates.

**Conclusion**: Given the positive results, additional cases will be presented at the meeting to verify and support the conclusions across multiple disease sites.

### O 128 - A high spatiotemporal resolution 2D strip ionization chamber array for proton pencil beam scanning FLASH radiotherapy

#### Yunjie Yang^1^, Chengyu Shi^1^, Chin-Cheng Chen^1^, Pingfang Tsai^1^, Minglei Kang^1^, Sheng Huang^1^, Chih-Hsun Lin^2^, Fu-Xiong Chang^3^, Charles Simone^4,5^, Haibo Lin^1,6^

##### *^1^New York Proton Center, Medical Physics, New York, USA*

*^2^Academia Sinica, Institute of Physics, Taipei City, Taiwan*

*^3^Liverage Biomedical Inc., Research and Development, Hsinchu, Taiwan*

*^4^New York Proton Center, Radiation Oncology, New York, USA*

*^5^Memorial Sloan Kettering Cancer Center, Radiation Oncology, New York, USA*

*^6^Memorial Sloan Kettering Cancer Center, Medical Physics, New York, USA*

**Purpose:** The lack of 2D detectors with adequate temporal performance limits the quality assurance of proton pencil beam scanning (PBS)-based FLASH radiotherapy (RT). In this study, we characterize a newly designed 2D strip-segmented ionization chamber array (SICA) with high spatiotemporal resolution and demonstrate its applications in a modern proton PBS delivery system at ultra-high dose rates.

**Methods:** A dedicated research beamline of the Varian ProBeam system was employed to deliver a 250 MeV proton PBS beam with nozzle currents up to 215 nA. The spatial, temporal, and dosimetric performance of the SICA was characterized and compared with measurement using an Advanced Markus ionization chamber, EBT-XD Gafchromic films, and a Faraday Cup. A novel approach was proposed to perform 2D dose reconstruction with such strip-type detectors.

**Results:** The SICA demonstrated a position accuracy of 0.12 ± 0.02 mm at a 20 kHz sampling rate (50 μs per event) and a linearity of R^2 ^> 0.99 for both dose and dose rate with nozzle beam currents ranging from 1 nA to 215 nA. The 2D dose comparison to the film measurement resulted in a gamma passing rate of 99.8% (2 mm/2%). A first measurement of the proton PBS 2D FLASH dose rate distributions was presented, compared to simulation results, and showed a gamma passing rate of 97.3% (2 mm/2%).

**Conclusions:** The newly designed SICA demonstrated excellent spatial, temporal, and dosimetric performance and is well suited for a wide range of clinical applications in commissioning and quality assurance in proton PBS FLASH-RT.

### O 129 - Real-time gated proton therapy: commissioning and clinical workflow for the Hitachi system

#### Hao Chen^1^, Emile Gogineni^1^, Yilin Cao^2^, John Wong^2^, Curtiland Deville^1^, Heng Li^1^

##### *^1^Johns Hopkins Unverisity, Radiation Oncology and Molecular Radiation Sciences, Washington- D.C., USA*

*^2^Johns Hopkins Unverisity, Radiation Oncology and Molecular Radiation Sciences, Baltimore, USA*

**Purpose**: To describe the commissioning of real-time gated proton therapy(RGPT) with the Hitachi Probeat and the establishment of an appropriate clinical workflow for the treatment of patients.

**Methods**: RGPT utilizes a matching score to provide instantaneous system performance feedback and quality control for patient safety as an assessment of intra-fraction motion. The matching score is influenced by several factors, including but not limited to: fluoroscopic image system parameters, real-time target tracking technique, and target motion. The CIRS Dynamic System combined with RSD Thorax Phantom or CIRS plastic water was utilized to mimic target motion. The OCTAVIUS 1500XDR was utilized to measure end-to-end dosimetric accuracy for a moving target across a range of simulated situations with the relevant RGPT parameters. Using this dosimetry data, the gating threshold was carefully evaluated and selected based on the intended treatment sites and planning techniques. An image-guidance workflow was developed and applied to patient treatment.

**Results**: Dosimetry data shows that proton plan delivery uncertainty could be within 2 mm for a moving target. Eight prostate cancer patients with implanted platinum fiducials markers have been treated with RGPT utilizing the derived workflow. Target motion and gating signal data are available for intra-fraction motion analysis.

**Conclusion**: RGPT with the Hitachi System has been fully investigated and commissioned for clinical application. RGPT could provide advanced and reliable real-time image guidance to enhance patient safety and inform important treatment planning parameters, such as planning target volume internal margins.

### O 130 - In-beam PET imaging of radioactive carbon ion beams with high-resolution detectors for the BARB project

#### Giulio Lovatti^1^, Munetaka Nitta^1^, Mohammad Safari^1^, Tim Binder^1^, Peter Thirolf^1^, Andreas Zoglauer^2^, Taiga Yamaya^3^, Christoph Scheidenberger^4^, Marco Durante^5^, Katia Parodi^1^

##### *^1^Ludwig-Maximilans-Universität, Medical Physics, Munich, Germany*

*^2^UC Berkeley, Space Science Laboratoty, Berkeley- CA, USA*

*^3^National Institutes for Quantum Science and Technology, National Institute of Radiological Sciences, Chiba, Japan*

*^4^Justus-Liebig University, Faculty of Mathematics and Computer Science, Giessen, Germany*

*^5^Helmholtzzentrum für Schwerionenforschung GSI, Biophysics division, Darmstadt, Germany*

The project Biomedical Applications of Radioactive ion Beams (BARB) aims to investigate the advantage of radioactive ion beams for image-guided therapy (see abstract D. Boscolo et al.), with emphasis on positron-emission-tomography (PET). In this first experiment C-10 and C-11 ion beams were produced at the fragment-separator (FRS) of GSI (see abstract D. Kostyleva et al.). For high-resolution in-beam PET imaging, we employed prototype detectors developed within the LMU project Small Animal Proton Irradiator for Research in Molecular Image-guided Radiation-oncology (SIRMIO) in collaboration with QST. This technology will serve as the basis for a hybrid PET-Compton detector to be realized in the framework of BARB. The 3-layers depth-of-interaction PET detectors feature scintillator blocks of pixelated (0.9 mm) LYSO (M. Nitta, IEEE, 2021). We used 8 detectors forming opposite units of 4 blocks [fig. 1]. The PMMA target was adjusted to set the Bragg peak position at the center of the PET field-of-view. The Geant4 and Medium-Energy Gamma-ray-Astronomy library (MEGAlib) toolkits were used for data analysis and simulation. The Toolkit for Multivariate-Data-Analysis machine learning was used for the classification of events, reconstructed with a Maximum-Likelihood-Expectation-Maximization iterative algorithm. The first results show the capability of the detector to visualize the beam stopping [fig. 2], even with a limited solid angle coverage. Comparison of measurements with simulations, limitations and ongoing improvements of the PET system will be discussed.

### O 131 - Flat panel proton radiography with a patient specific imaging field for accurate WEPL assessment

#### Carmen Seller Oria^1^, Jeffrey Free^1^, Gabriel Guterres Marmitt^1^, Barbara Knäusl^2^, Sytze Brandenburg^1^, Antje C. Knopf^1,3^, Arturs Meijers^4^, Johannes A. Langendijk^1^, Stefan Both^1^

##### *^1^University Medical Center Groningen- University of Groningen, Radiation Oncology, Groningen, Netherlands*

*^2^Medical University of Vienna, Radiation Oncology, Vienna, Austria*

*^3^Center for Integrated Oncology Cologne- University Hospital of Cologne, Internal medicine, Cologne, Germany*

*^4^Paul Scherrer Institute, Center for proton therapy, Villigen, Switzerland*

**Purpose:** Water-equivalent path length (WEPL) measurements using flat panel proton radiography (FP-PR) could be used for proton range verification. Accurate WEPL measurements via FP-PR are achievable using several energy layers. To minimize imaging dose, we propose a FP-PR method for accurate WEPL determination based on a patient specific imaging field with a reduced number of energies (n).

**Materials and methods:** Patient specific FP-PRs were simulated and measured for a head and neck phantom. An energy selection algorithm estimated per spot coordinate (i,j) the lowest energy Emin(i,j) required to cross the anatomy using a water equivalent thickness (WET) map and a calibration dataset. Starting from Emin(i,j), higher energies were determined up to Emax(i,j), in accordance with specific numbers (n=26, 24, 22,…, 2 for simulations, n=10 for measurements)(figure 1). WEPL accuracy of patient specific FP-PRs was assessed in the base of skull, neck and brain of the phantom (figure 2(a)) using mean relative WEPL errors (MRE) and standard deviations against multi-layer ionization chamber PR (MLIC-PR) simulations and measurements.

**Results:** Via simulations, MREs were up to 11.4±2.8% for n<10, while for n≥10 MREs were below 0.7±1.6% in the three anatomical regions (figure 2(b)).Measured FP-PRs (n=10) showed MREs of -1.1±1.8%, -0.1±1.0%, -0.1±0.5% against MLIC-PR measurements in the base of skull, neck and brain.

**Conclusion:** A method to accurately measure WEPL using FP-PR with a reduced number of energies tailored to the patient anatomy was established in silico and verified experimentally. Patient specific FP-PRs could assist range verification processes within online adaptive proton therapy workflows.

### O 132 - Use of CBCT and plan robustness for determining the time of adaptive planning in IMPT: Head and neck cases

#### Weiguang Yao^1^, Baoshe Zhang^1^, Dong Han^1^, Jerimy Polf^1^, Sastry Vedam^1^, Giovanni Lasio^1^, Byongyong Yi^1^

##### ^1^University of Maryland School of Medicine, Radiation Oncology, Baltimore, USA

Anatomic variation has a significant dosimetric impact in intensity modulated proton therapy (IMPT). Routine CT scans, called QACTs, have been used to determine if an adaptive plan (AP) is needed. This work proposes to use a CBCT, which is acquired during the treatment setup, and plan robustness to determine the necessity of AP. We retrospectively analyzed 27 head and neck patients, associated with 594 CBCTs, 136 QACTs, and 19 adaptive plans. For each CBCT, water equivalent thickness (WET) along the pencil beam path between the target surface and beam source was calculated. The WET of the first day CBCT (1^st^ CBCT) was used as the reference. A linear regression model was generated to predict the change of WET (ΔWET) on the QACT day. The impact of range change on target dose was calculated as the predicted ΔWET weighted by the monitor units of each fields. In addition, the plan robustness was estimated from the robust dose-volume histograms (DVHs) and combined with ΔWET to determine if an AP is needed. When the estimated mean WET change was < 6.5 mm, or < 7.5 mm but the robustness > 95%, AP was not needed. Otherwise the QACT for AP was required. Overall, 41% or 56% of QACTs could be reduced when ΔWET < 6.5 mm, or < 7.5 mm but the robustness (*R*) > 95%. This study shows that CBCT can be used to determine the time of AP which reduces QACT frequency greatly, and further reduced by combining the analysis on plan robustness.

### O 133 - Validation of CBCT-Based online evaluation for proton therapy

#### Chih-Wei Chang^1^, Rasmus Nilsson^2^, Sebastian Andersson^2^, Duncan Bohannon^1^, Liyong Lin^1^, Sagar Patel^1^, Pretesh Patel^1^, Jeffrey Bradley^1^, Tian Liu^1^, Xiaofeng Yang^1^, Jun Zhou^1^

##### *^1^Emory University, Radiation Oncology, Atlanta, USA*

*^2^RaySearch Laboratories AB, RaySearch Laboratories AB, Stockholm, Sweden*

**Purpose:** To validate the accuracy and efficiency of cone-beam CT (CBCT) based online evaluation workflows for different treatment sites in proton therapy.

**Methods:** Twenty-three patients (eight head and neck (HN), seven lung, and eight prostate with nodes patients) were evaluated. The online CBCT evaluation workflow based on a research version of RayStation 11B planning system includes two imaging generation methods: corrected CBCT (cCBCT) and deformed planning CT (vCT). The same day deformed quality assurance CT (QACT) (to the CBCT) was used as the reference. Computing time was analyzed for the workflows with different images. The gamma index (1%/1 mm) were evaluated to quantify the dosimetric accuracy of the cCBCT and vCT.

**Results:** Mean online evaluation time among all patients were 98.9±18.0s and 117.1±19.5s for workflows with cCBCT and vCT, respectively. The cCBCT workflow time for the prostate is slightly higher than that for the lung and HN, 102.6s±24.6s vs 99.9±18.2s vs 93.7±6.3s. Monte Carlo dose calculation and deformable image registration accounted for ∼64% of the cCBCT workflow time. The generation of cCBCT and ROI mapping accounted for ∼17% and ∼8% of the evaluation time. The mean gamma passing rates for HN, lung, and prostate patients using cCBCT were 95.5%±2.2%, 91.9%±4.0%, and 97.9%±1.4%, respectively. The corresponding rates using vCT were 94.3%±1.8%, 94.0%±4.2%, and 98.3%±1.0%.

**Conclusion:** The cCBCT-based online evaluation can be achieved within two minutes for most cases. The vCT workflow needs additional 18.2s and it achieves a better gamma passing rate for the lung cases.

### O 134 - Patterns Of Practice for Adaptive and Real-Time Particle Therapy (POP-ART PT), part II: Plan adaptation for interfractional changes

#### Petra Trnkova^1^, Ye Zhang^2^, Toshiyuki Toshito^3^, Ben Heijmen^4^, Christian Richter^5^, Marianne Aznar^6^, Albertini Francesca and Alessandra Bolsi^2^, Juliane Daartz^7^, Antje Knopf^2^, Jenny Bertholet^8^

##### *^1^Medical University of Vienna, Department of Radiation Oncology, Vienna, Austria*

*^2^Paul Scherrer Institut, Center for Proton Therapy, Villigen-PSI, Switzerland*

*^3^Nagoya City University West Medical Center, Nagoya Proton Therapy Center, Nagoya, Japan*

*^4^Department of Radiotherapy, Erasmus Medical Center, Rotterdam, Netherlands*

*^5^OncoRay – National Center for Radiation Research in Oncology- Faculty of Medicine and University Hospital Carl Gustav Carus, Technische Universität Dresden- Helmholtz-Zentrum Dresden - Rossendorf, Dresden, Germany*

*^6^Division of Cancer Sciences- Faculty of Biology- Medicine and Health, University of Manchester, Manchester, United Kingdom*

*^7^Department of Radiation Oncology, Massachusetts General Hospital, Boston, USA*

*^8^Inselspital- Bern University Hospital, Division of Medical Radiation Physics and Department of Radiation Oncology, Bern, Switzerland*

Real-time respiratory motion management (RRMM, intra-fraction geometrical intervention) and Adaptive Particle Therapy (APT, inter-fraction adaptation of treatment plans) enable to account for anatomical variations and changes to optimize target coverage and organs-at-risk sparing. However, their current clinical implementation is unclear and expected to be highly heterogeneous. An institutional questionnaire, *Patterns Of Practice for Adaptive and Real-Time Particle Therapy (POP-ART-PT)*, was distributed between 2021/01-06 to evaluate current clinical practice and wishes and barriers of the implementation. Here, we summarise the international survey results on APT for mitigation of interfractional anatomical changes from 70 particle therapy centers in 17 countries. The response rate was 100% for Europe, 96% for Japan and 53% for USA. Of the 68 centers in operation, 84% (Figure1a) were APT users for at least one treatment site, with head and neck being the most common. APT was mostly performed offline (ad-hoc or per protocol) with only two users of online APT (plan library) and none using online daily replanning. Plan adaptation was in all cases motivated by both, target and OAR dose considerations (Figure1b). The most common imaging modality guiding APT was X-ray computed tomography. Sixty-eight percent of users plan to increase or change their APT technique. The greatest barriers to implementation were lack of integrated and efficient workflows and human resources (Figure2) Offline APT has been widely implemented internationally, but online APT is still very rarely used. More research and development for integrated and efficient workflow is needed to facilitate the use of offline APT and enable online APT.

### O 135 - Can we avoid complex daily online replanning via online adaptive plan library strategies for head and neck IMPT?

#### Michelle Oud^1^, Sebastiaan Breedveld^1^, Marta Giżyńska^2^, Zoltán Perkó^3^, Ben Heijmen^1^, Mischa Hoogeman^1,2^

##### *^1^Erasmus MC Cancer Institute, Department of Radiation Oncology, Rotterdam, Netherlands*

*^2^Holland Proton Therapy Center, Department of Medical Physics & Informatics, Delft, Netherlands*

*^3^Delft University of Technology, Faculty of Applied Sciences- Department of Radiation Science and Technology, Delft, Netherlands*

**Purpose:** To compare two clinically feasible online adaptive (OA) plan library (PL) strategies (OA-1 and OA-2) to conventional robust treatment planning (CONV) and full daily online adaptive replanning (OA-3) for 15 H&N patients treated with IMPT.

**Materials/Methods:** CONV consisted of a treatment plan generated for the planning CT (pCT) using 3 mm setup robustness settings (SRS) and 3% range robustness settings (RRS). OA-1 was a PL filled with 5 plans created using the pCT, each plan was generated with different SRS: 0, 1, 2, 3 or 5 mm and RRS=3% for all. OA-2 followed the same concept as OA-1, but the PL was (bi-)weekly extended based on the previous repeat CT (rCT) (progressive PL). OA-3 consisted of full daily online adaptive replanning with SRS=1mm, RRS=3%. For each patient, 25 fully fractionated treatment courses were simulated for all 4 strategies with realistic setup and range errors using 3-6 rCTs. For OA-1&2, daily online plan selection was based on CTV coverage on the rCT. Fraction doses were accumulated.

**Results:** In CONV, 82.1% of the simulated treatments complied with clinical CTV constraints compared to 90.9%, 94.4% and 97.6% for OA1-3 (Tab. 1). Compared to OA-2, the risk of xerostomia grade ≥ II decreased on average with 1.3±1.7%-point for OA-3, while for dysphagia grade ≥ II this was 1.7±2.5%-point (Fig. 1).

**Conclusion:** Plan library strategies (OA-1&2) improved adherence to CTV constraints and reduced NTCPs compared to conventional planning (CONV). Approaching the quality of daily online replanning (OA-3), the progressive plan library (OA-2) may be an already feasible alternative.

### O 136 - Clinical implementation of Dual Energy CT calculated proton stopping power ratio: treatments with reduced range uncertainty margins

#### Kevin Teo^1^, Wei Zou^1^, Taoran Li^1^, Lei Dong^1^, Robert A Lustig^1^, Michelle Alonso-Basanta^1^, Goldie Kurtz^1^, John Lukens^1^, Alexander Lin^1^, Shannon O'Reilly^1^

##### ^1^University of Pennsylvania, Radiation Oncology, Philadelphia, USA

**Purpose**: We report on the first clinical implementation of Dual Energy CT (DECT) for proton plan optimization and treatment with reduced range uncertainty margins. Single energy CT (SECT) typically use 3.5% proton range uncertainty in robust plan optimization but with DECT, a reduced margin of 2% is used. Between September 2020 and February 2022, 258 brain and 174 head and neck patients were treated with DECT plans with 2% range uncertainty. Additionally, head and neck simulations can utilize a single DECT contrast scan to generate a contouring CT and a proton stopping power ratio (SPR) CT for planning without the need for a non-contrast scan.

**Methods**: The Siemens SOMATOM Drive and Edge were used for the DECT scans. An in house software was used to generate the DECT SPR which is then converted to a planning CT by reversing the HU-to-SPR conversion table in the planning system (Eclipse v16.1). Differences in SPR between SECT and DECT calculation were analyzed for muscle, adipose, brain and dense bone. The impact of reduced range uncertainty on several organs at risk were computed.

**Results**: DECT predicted SPR was about 0.75% higher for muscle, 2.8% lower for adipose, 0.93% higher for brain and 2.0% lower for dense bone compared to SECT calibration. Small improvements were seen in brain stem, optical structures, submandibular, mandible and oral cavity doses in the DECT plans.

**Conclusions**: DECT planning and treatment with reduced margins has been demonstrated. The dosimetric improvement in OAR doses is patient specific and dependent on beam orientation as well as proximity to the target.

### O 137 - The potential of including anatomical error scenarios for nasal cavity filling in robust optimization

#### Nadine Vatterodt^1,2^, Raúl Argota-Pérez^3,4^, Maja Bendtsen Sharma^3^, Ulrik Vindelev Elstrøm^2^, Kenneth Jensen^2^, Stine Sofia Korreman^1,2,3^

##### *^1^Aarhus University, Department of Clinical Medicine, Aarhus, Denmark*

*^2^Aarhus University Hospital, Danish Center for Particle Therapy, Aarhus, Denmark*

*^3^Aarhus University Hospital, Department of Oncology, Aarhus, Denmark*

*^4^Herlev and Gentofte Hospital, Department of Oncology, Herlev, Denmark*

**Purpose:** We investigated the dosimetric cost of including anatomical error scenarios to account for changes in nasal cavity filling compared to conventional robust optimization for sinonasal cancer.

**Material and methods:** A retrospective pilot study on 10 sinonasal cancer patients was performed in RayStation v8.99.0.3. Conventional robust optimized (cRO) plans were generated with robustness parameters of ±2mm setup and ±3.5% range uncertainty. Full robust optimized (fRO) plans were obtained by additionally including two artificial CTs with filled and cleared nasal cavities as error scenarios. Plans were approved according to passing criteria in Table 1. Recalculated doses on synthetic CTs derived from two daily CBCT with distinct differences in cavity filling were mapped to the planning CT (recal1/2). Results were evaluated by comparison of target coverage and dose to OAR using Wilcoxon signed-rank tests to identify significant differences (p<0.05).

**Results**: Nominal plans provided comparable target coverage without significant differences (Figure 2a). For OAR the only significant increase for fRO plans was 4.4 Gy in maximum dose for the brainstem (difference between medians). The fRO plans showed superior robustness in target coverage and in most OARs when comparing the differences in median of maximum dose of recalculated to nominal plans (Figure 2b).

**Conclusion:** Including anatomical robustness scenarios for planning did not increase dose to most OARs compared to conventional robust optimization. The recalculations indicate increased robustness towards actual changes in cavity filling for fRO plans. Dosimetric robustness for fRO plans by dose accumulation over all fractions will be presented at the conference.

### O 138 - CBCT based online adaptive proton therapy using the direct field-specific water-equivalent-length correction approach: a validation study

#### Guo Mengya^1,2^, Michelle Van Heerden^1^, Francesca Albertini^1^, Sairos Safai^1^, Damien Weber^1^, Antony Lomax^1^, Zhiling Chen^2^, Ye Zhang^1^

##### *^1^Paul Scherrer Institut, Center for Proton Therapy, Villigen-PSI, Switzerland*

*^2^Chinese Academy of Sciences, Shanghai Institute of Applied Physics, Shanghai, China*

The pronounced discrepancy in Hounsfield-Unit between Cone-Beam CT (CBCT) and pre-treatment planning CT (pCT) hinders its application for online adaptive proton therapy (APT). A water-equivalent-length (WEL) correction approach is proposed and validated to enable CBCT based online plan re-calculation and re-optimization. At pre-treatment phase (Figure1a), IMPT plan is constructed using pCT. Planning CBCT (pCBCT) is acquired and normalized to the pCT using histogram matching algorithm [Kidar, *RadiatOncol* **13-**217]. For individual field, WEL distributions (WEL_pCT and WEL_pCBCT) are respectively calculated for each dose-calculation-grid from pCT and normalized pCBCT, whose ratio is computed as WEL-correction-map. During treatment, fractional dose is re-calculated or re-optimized using derived WEL_frCBCT based on the normalized frCBCT (to pCT) and the WEL-correction-map. We validated this approach using paired CBCT-CT images of a physical head phantom with anatomic variation (Figure1b). The derived fractional dose from frCBCT with WEL correction was compared to the reference when fraction CT (frCT) was used. Dosimetric accuracy of this correction approach is then evaluated for four skull-based patients. Phantom validation (Figure2a) showed the excellent dosimetric similarity between corresponding dose distributions derived from frCT and frCBCT using WEL correction, in presence of pronounced anatomic variations. The 3%-3mm-gramma-pass-rates were 98.4%/98.2% and 97.76%/99.08% (single-/three-field plans) for re-calculated and re-optimized plans*,* in comparison to 80.6%/84.7% and 73.97%/82.56% when no WEL correction was applied. For clinical cases (Figure2b) the averaged 3%-3mm-gramma-pass-rate can also reach 97.7% with comparable DVHs for most critical structures. The field-specific water-equivalent-length correction approach is demonstrated to be effective and accurate for online APT.

### O 139 - On-line and post irradiation imaging of proton treatment with the PETITION PET detector

#### Keegan McNamara^1,2^, Shubhangi Makkar^1,2^, Marina Béguin^3^, Günther Dissertori^3^, Judith Flock^3^, Cristian Fuentes^3^, Christian Ritzer^3^, Damien C. Weber^1,4,5^, Antony Lomax^1,2^, Carla Winterhalter^1,2^

##### *^1^Paul Scherrer Institut, Center for Proton Therapy, Villigen, Switzerland*

*^2^ETH Zürich, Department of Physics, Zürich, Switzerland*

*^3^ETH Zürich, Institute for Particle Physics and Astrophysics, Zürich, Switzerland*

*^4^University Hospital of Zürich, Radiation Oncology Department, Zürich, Switzerland*

*^5^University Hospital of Bern, Radiation Oncology Department, Bern, Switzerland*

We present a simulation workflow to investigate on-line and post irradiation PET imaging of head and neck cancer patients using the PETITION PET detector. The production of positron emitting isotopes within a patient is calculated using the GPU accelerated Monte Carlo code FRED (v3.59.9), allowing fast calculation of the complex activation distribution within the patient for individual spots, energy layers, and fields. Decay of the isotopes and measurement of the coincident gamma rays by the open ring detector is simulated in GATE (v9.1). Low resolution imaging of short-lived isotopes such as 12N during energy switching periods of around 100 ms provides immediate feedback during treatment through 2D projection of the recorded coincidences, shown in Fig. 1. 12N contributes to 89.8% of recorded interactions after delivery of the first layer, but only 12.7% after the final layer due to ongoing production of longer-lived isotopes such as 15O, shown in Fig. 2. Post irradiation acquisition for several minutes after beam off provides sufficient number of detected coincidences for full 3D reconstruction of the activity generated. Acquisition immediately after beam off may enable dynamic imaging of 15O and 11C over different time scales, and provide greater information for verification of proton range as opposed to a single image. Fast GPU simulation of the activity in the patient allows for quick recalculation of plans and expected activity based on possible deviations in patient anatomy, differences in delivery, and patient positioning errors. The workflow will enable broader application of PET imaging for treatment verification.

### O 140 - Multi-institutional benchmark experiment of prompt-gamma imaging and spectroscopy for treatment verification using an anthropomorphic head phantom

#### Jonathan Berthold^1,2,3^, Fernando Hueso-González^3,4,5^, Patrick Wohlfahrt^4,6^, Thomas Bortfeld^4^, Chirasak Khamfongkhruea^1,7^, Sebastian Tattenberg^4^, Melek Zarifi^4,8^, Christian Richter^1,2,3,9,10^, Joost Verburg^3,4^

##### *^1^OncoRay - National Center for Radiation Research in Oncology, Faculty of Medicine and University Hospital Carl Gustav Carus- Technische Universität Dresden- Helmholtz-Zentrum Dresden - Rossendorf, Dresden, Germany*

*^2^Helmholtz-Zentrum Dresden - Rossendorf, Institute of Radiooncology - OncoRay, Dresden, Germany*

*^3^Authors contributed equally, Unknown Country*

*^4^Department of Radiation Oncology, Massachusetts General Hospital and Harvard Medical School, Boston MA, USA*

*^5^Now with: Instituto de Física Corpuscular CSIC / UVEG, Paterna, Spain*

*^6^Now with: Siemens Healthineers, Forchheim, Germany*

*^7^Princess Srisavangavadhana College of Medicine, Chulabhorn Royal Academy, Bangkok, Thailand*

*^8^Now with: Australian Nuclear Science and Technology Organisation ANSTO, Lucas Heights NSW, Australia*

*^9^Department of Radiotherapy and Radiation Oncology, Faculty of Medicine and University Hospital Carl Gustav Carus- Technische Universität Dresden, Dresden, Germany*

*^10^German Cancer Consortium DKTK - partner site Dresden, Germany and German Cancer Research Center DKFZ, Heidelberg, Germany*

**Introduction**: In-vivo range verification has been clinically implemented using either prompt-gamma imaging (PGI) or spectroscopy (PGS). Here, two proton therapy centers, currently investigating the clinical benefit of PGI and PGS, collaborated to systematically compare both techniques in a set of benchmark experiments under equalized conditions.

**Materials and methods**: The same anthropomorphic head phantom (CIRS, USA) was used for treatment planning, beam delivery and prompt-gamma monitoring in both centers. Two pencil-beam-scanning fields (1GyE) targeting a brain lesion were optimized on a dual-energy CT using the same spot positions and energies in both centers, enabling spot-wise comparison (Fig1). The horizontal, *short-range* field was clinically realistic. The *long-range,* oblique field served as stress test. Absolute range verification accuracy against a blinded ground truth (3D stopping-power-ratio map) for both fields as well as the capability to detect relative range shifts, introduced by plastic slabs (2 and 5mm) on half of the short-range field, were assessed.

**Results** (μ±σ): The absolute accuracy of PGI and PGS were (-0.5±0.8)mm or (2.4±0.8)mm for the short-range (Fig2A) and (2.4±1.9)mm or (1.3±1.5)mm for the long-range field, respectively. Relative range shifts were detected with (2.0±0.9)mm or (4.2±0.8)mm accuracy for PGI, and (1.8±0.5)mm or (4.8±0.4)mm for PGS (Fig2B). Both systems show a performance worthy of clinical application for the detection of range deviations. Future improvements of their sensitivity are ongoing.

**Conclusion**: For the first time, two independent prompt-gamma range verification systems utilizing different prompt-gamma information have been successfully benchmarked under equalized conditions in two proton therapy centers. This marks an important milestone for translational research on proton treatment verification.

### O 141 - Patient QA fully based on machine logfiles via independent Monte Carlo in our proton therapy facility

#### Ilaria Rinaldi^1^, Jan Gajewski^2^, Angelo Schiavi^3^, Nils Krah^4^, Antoni Rucinski^2^, Vincenzo Patera^3^, Bas Nijsten^1^

##### *^1^MAASTRO, Proton therapy center, Maastricht, Netherlands*

*^2^Polish Academy of Sciences, Institute of Nuclear Physics, Krakow, Poland*

*^3^Sapienza University, Dipartimento di Scienze di Base e Applicate per l'Ingegneria, Rome, Italy*

*^4^University of Lyon, CREATIS-CNRS UMR5220- Inserm U1044- INSA-Lyon- Université Lyon 1- Centre Léon Bérard, Lyon, France*

In our proton facility, the Maastro proton therapy center in Maastricht, machine logfile-based patient QA using the independent GPU-accelerated Monte Carlo Fred fully replaces measurements.We design a new hybrid method to implement the Mevion S250i Hyperscan system in Fred due to its peculiarities: The proton beam is described via a phase space model upstream of the nozzle and then tracked explicitly across the nozzle. Validation of Fred passed all clinical acceptance criteria. A pipeline is fully operative, and review of automatic verification reports has become part of daily clinical routine. Recalculation of patient dose distributions takes only a few minutes allowing for quick on-the-fly execution of the pipeline. For the first months after the introduction of this pipeline in our clinical routine in summer 2020, the full 3D Monte Carlo dose recalculation of QA plans complemented the 2D patient QA measurements. Nowadays, patient QA measurements are performed only for plans failing the automatic verification. Until now we have used Fred to recalculate over one-thousand machine logfile-based patient QA in our proton therapy center. This corresponds to 500 hours less routine QA work, given that one QA measurement takes about 30 min (on average). Less than 5 patient QA have so far prompted manual intervention. A comprehensive analysis of the results will be presented including statistics about the Monte Carlo performance and workflow. Impact on daily routine is high because the recalculation pipeline provides staff with valuable 3D dosimetric information complementary to 2D QA measurements without the need of dedicated beam time.

### O 142 - Safety assessment of measurement-based and log file-based patient-specific quality assurance: A report of the PTCOG Treatment Efficiency Subcommittee

#### Daniel Robertson^1^, Christina Dahlgren^2^, Chin-Cheng Chen^3^, Heng Li^4^, Frank Emert^5^

##### *^1^Mayo Clinic Arizona, Radiation Oncology, Phoenix, USA*

*^2^The Skandion Clinic, Department of Radiation Oncology, Uppsala, Sweden*

*^3^New York Proton Center, Radiation Oncology, New York, USA*

*^4^Johns Hopkins Medicine, Department of Radiation Oncology, Washington- DC, USA*

*^5^Paul Scherrer Institut, Radiation Oncology, Villigen, Switzerland*

**Introduction**: The PTCOG Treatment Efficiency Subcommittee has studied the benefits and challenges of using treatment logs for patient-specific quality assurance (PSQA). A risk analysis was performed, including a detailed look at the role of the radiation oncology information system, treatment planning system, and delivery system, as well as communication between those systems.

**Methods**: A process map was developed for the proton treatment process, beginning after simulation and extending through the end of treatment. The process map included separate tracks for no PSQA, second calculation only, measurement-based PSQA, and log file-based PSQA. Failure modes were identified for each process step, and a FMEA was carried out for each track. The twenty highest-scoring failure modes with scores for all four tracks were selected for comparison, and the total RPN score was calculated for each PSQA track and normalized against the “no PSQA” track. High-scoring failure modes without scores for all four tracks were also selected for evaluation.

**Results**: The use of a second calculation decreased the overall risk of the twenty selected failure modes by 18%. Measurement-based PSQA decreased the overall risk by 38% relative to no PSQA. Log file-based PSQA decreased the overall risk by 43% relative to no PSQA, but it added new failure modes that can increase overall risk.

**Conclusions**: Log file-based PSQA provided the greatest decrease in overall risk. Measurement-based PSQA provided a similar risk reduction to log file-based PSQA in the common failure modes, but it also introduced several additional high-risk failure modes.

### O 143 - Proton Bragg peak detection by acoustically-modulated nanodroplets at body temperature enables direct in vivo range verification

#### Sophie Heymans^1^, Gonzalo Collado-Lara^2^, Marta Rovituso^3^, Hendrik J. Vos^2^, Jan D'hooge^4^, Nico de Jong^2^, Koen Van Den Abeele^1^

##### *^1^KU Leuven, Department of Physics, Kortrijk, Belgium*

*^2^Erasmus Medical Center, Biomedical Engineering, Rotterdam, Netherlands*

*^3^Holland Proton Therapy Center, Physics, Delft, Netherlands*

*^4^KU Leuven, Department of Cardiology, Leuven, Belgium*

The efficacy of proton therapy is currently limited by range uncertainties. A novel *in vivo* range verification method was recently proposed, based on ultrasound imaging of the vaporization of injectable superheated nanodroplets upon proton irradiation. At 50°C, nanodroplet vaporization by primary protons leads to a vaporization peak at the proton range (R_80_), allowing to directly retrieve the range with sub-millimeter accuracy. However, at 37°C, vaporization is triggered by rare high-LET secondary particles, limiting the technique to indirect range verification. To enable direct nanodroplet vaporization by protons* in vivo*, the nanodroplet degree of superheat at body temperature must be increased. This can be achieved by decreasing the pressure inside the nanodroplet liquid core. Here, we investigated whether acoustic waves could modulate the nanodroplet degree of superheat, enabling proton-induced vaporization during the rarefactional phase. To that aim, a tube containing a nanodroplet dilution was irradiated with a 158 MeV pencil proton beam (Fig. 1a). Starting from 50°C, the temperature was gradually decreased until vaporization by primary protons was no longer observed (41°C, Fig. 1b). Next, nanodroplets were irradiated and simultaneously sonicated with sequences of 1.1 MHz acoustic waves at increasing pressure. At 37°C, nanodroplet vaporization by protons was observed using acoustic pressures ≥400 kPa (Fig. 1c), while ultrasound alone did not induce vaporization. Since the acoustic pressure threshold is well within the safety limits for diagnostic ultrasound, these proof-of-concept results suggest that acoustic modulation can potentially enable direct *in vivo* proton range verification through nanodroplet vaporization at the proton range.

### O 144 - Single-shot thermoacoustic measurements during conventional delivery – pristine VS. planned

#### Sarah Patch^1^, Yao Hao^2^, Tianyu Zhao^2^

##### *^1^Acoustic Range Estimates, all, Chicago, USA*

*^2^Washington University in St Louis, Radiation Oncology / physics, St Louis, USA*

**Purpose:** To determine feasibility of thermoacoustic range verification during conventional delivery with Mevion Hyperscan.

**Materials/Methods:** Mevion Hyperscan S250i delivered pristine pulses at each energy utilized by a 2-field plan that delivered 3 Gy to the left kidney in the CIRS 057a multi-modality anthropomorphic phantom made of hydrogels (Fig. 1a-e). A PMT assembly and 4-channel thermoacoustic prototype captured nuclear and thermoacoustic emissions (TAE), respectively (Fig. 2a-b). Both pristine and planned beam were delivered with a 750 Hz repetition frequency. PMT pulses were centered via Fourier shift and area under curve (AUC) of each pulse was computed.

**Results:** Strongest TAE were detected from Field A (180° gantry angle) with prototype positioned distal to the Bragg peak. Averaging 50 thermoacoustic emissions (TAE) generated by pristine beam show arrival times from the Bragg peak shifted with beam energy, whereas TAE from the beam entry point were consistent across all energies (Fig. 1f). Single-shot TAE corresponding to strong nuclear emissions (AUC>) were correlated (Fig. 2c-d), despite variations in nuclear emissions (Fig. 2e-f). Fig. 2e highlights variable ramp-up of pristine pulses, sampled at 10 MSPS. Fig. 2f is sampled at only 1 MSPS, which failed to capture variations during planned delivery.

**Conclusions:** Close proximity of the Hyperscan's accelerator does not cause overwhelming electromagnetic noise. Acoustic reverberations during pristine and planned delivery should have been comparable. Shifting to align PMT ramp-up might improve coherence of TA signals and requires sampling faster than 1 MSPS, so clinical systems will require a fast data acquisition board.

### O 145 - Absolute and direct energy measurement of clinical proton beams

#### Anna Vignati^1,2^, Mohammed A. A. Abujami^1,2^, Davide Bersani^1,2^, Cosimo Galeone^1,3^, Simona Giordanengo^2^, Oscar Ariel Martì Villarreal^1,2^, Felix Mas Milian^1,4^, Elisabetta Medina^1,2^, Roberto Sacchi^1,2^, Vincenzo Monaco^1,2^

##### *^1^Università degli Studi di Torino, Physics Department, Turin, Italy*

*^2^INFN, Torino, Torino, Italy*

*^3^GSI Helmholtzzentrum fur Schwerionenforschung, Physics, Darmstadt, Germany*

*^4^Universidade Estadual de Santa Cruz, Physics, Ilheus, Brazil*

**Purpose.** The Torino University and INFN developed an innovative instrument, exploiting Ultra-Fast-Silicon-Detectors' (UFSDs) excellent time resolution (tens of ps), to identify clinical beams' single protons and accurately determine the energy, quickly and with minimal beam perturbation. The technology, now meant for fast pre-treatment controls, could be applied for online beam energy monitoring in future adaptive therapy approaches.

**Materials and Method.** The beam energy is determined by measuring the time-of-flight of coincident protons passing through two UFSDs (11 strips of 2.2 mm^2^ each, 70/50 μm total/active thickness) aligned along the beam. The distance between sensors, measured with an optical encoder, is varied up to 950 mm by motors. The sensors signals are readout by a multichannel board, optimized for timing measurement, and acquired by a digitizer (16 ch, 5 GS/s). A calibration procedure, object of an Italian Patent Application, allows determining the unknown parameters (time offset, absolute distances) needed for measuring the absolute energy independently of any a priory beam information.

**Results.** Tests of a prototype were performed at CNAO (Pavia, synchrotron) and at TPTC (Trento, cyclotron) at clinical energies (60-227 MeV) and fluence rates (5·10^8 p/cm^2^·s). For <10 s irradiation time, deviations between nominal and measured energies between tens of keV and 420 keV were found at 95 cm distance between sensors, within a corresponding 1 mm range deviation and therefore in compliance with clinical requirements.

**Conclusions.** The capability of the proposed detector to fast and directly measure the energy of therapeutic proton beams without any external reference has been demonstrated.

### O 146 - QuARC: A Quality Assurance Range Calorimeter for proton therapy

#### Saad Shaikh^1^, Raffaella Radogna^2^, Fern Pannell^1^, Ruben Saakyan^1^, Spyros Manolopoulos^3^, Derek Attree^1^, Connor Godden^1^, Simon Jolly^1^

##### *^1^University College London, Physics and Astronomy, London, United Kingdom*

*^2^University of Bari Aldo Moro, Physics, Bari, Italy*

*^3^University College London, Medical Physics and Biomedical Engineering, London, United Kingdom*

Range uncertainties remain the largest source of uncertainty in proton therapy and prevent taking full advantage of its superior dose conformity. To optimise patient safety, daily quality assurance (QA) procedures are carried out each day before treatment begins, which are often time-consuming. In FLASH proton therapy, short treatment delivery times and high dose rates mean that typical ionisation chamber-based dosimetry methods become unusable. These dose rates are currently estimated to be approximately 40 Gy/s or 600 nA to the patient. The Quality Assurance Range Calorimeter (QuARC) is currently under development at UCL to provide fast, accurate, water-equivalent proton range measurements to speed up daily QA, with the capability to operate at FLASH dose rates. The detector is a series of optically isolated plastic scintillator sheets that sample the proton dose deposition along its path length. Each scintillator sheet is coupled to a photodiode that measures its light output directly. An analytical depth-light model is used to fit the data and measure the proton range. Two preliminary beam tests at UCLH with pencil beams between 70-110 MeV found the QuARC able to consistently recover proton ranges with good accuracy, even at low dose rates. Due to its large dynamic range – able to measure at least a 100-fold increase in dose – this will scale up to FLASH dose rates. Live fitting of the captured data enables stable real-time range reconstruction at 40 Hz. Further measurements are required to fully characterise the detector performance and light output with FLASH.

### O 147 - Centre specific organ dose constraints for carbon ion radiotherapy: translations between European and Japanese radiobiological models and preliminary clinical results

#### Joanna Gora^1^, Sarah Grosshagauer^2^, Mansure Schafasand^3^, Antonio Carlino^1^, Tobias Radakovits^2^, Markus Stock^1^, Eugen Hug^1^, Petra Georg^1^, Maciej Pelak^1^, Piero Fossati^1^

##### *^1^MedAustron Ion Therapy Center- Austria, Medical Department, Wiener Neustadt, Austria*

*^2^Technical University of Vienna- Austria, Atomic Institute, Vienna, Austria*

*^3^MedAustron Ion Therapy Center- Austria- Medical University of Vienna- Austria, Medical Department, Wiener Neustadt, Austria*

Carbon ion radiotherapy (CIRT) treatment planning is based on relative biological effectiveness (RBE)-weighted dose calculations. Majority of clinical evidence for CIRT was collected in Japan with RBE estimated by the microdosimetric kinetic model (MKM) while all European centers apply the local effect model (LEM). Several European clinics adapted Japanese treatment schedules, which requires adaptations in RBE-weighted dose prescriptions and constraints. Based on the previous publications, MedAustron implemented less conservative OARs constrains for brain parenchyma, brainstem and optic system. The aim of this study was to analyze them, considering both RBE models and evaluating early clinical data. Thirty-one patients receiving CIRT at MedAustron were analyzed using the RayStationV9 planning system by recalculating clinical LEM-based plans in MKM. Dose statistics (D1cm^3^, D5cm^3^, D0.1cm^3^, D0.7cm^3^, D10%, D20%) were extracted for relevant critical OARs. Curve fitting for those values was performed, resulting in linear quadratic translation models. Clinical and radiological toxicity was evaluated. Currently applied LEM constraints matched recommended MKM constraints with deviations between -3.2 and 1.1 Gy RBE, except of few cases, where data did not follow the expected LEMvsMKM trends. Radiological (asymptomatic) toxicity was detect in those outliers. This study proved limited applicability of translation-based constraints. *R*especting LEM constraints does not automatically guarantee fulfilling MKM constraints, therefore careful planning strategies selection is essential. Our current, updated clinical practice ensures that OARs constraints must be fulfilled for both models. To our knowledge, it is the first time that simultaneous evaluation of multiple RBE models is employed for the clinical decision making process in CIRT.

### O 148 - Automatic decision tool for head and neck patient referral based on dose prediction with deep learning

#### Margerie Huet Dastarac^1^, Ana Barragán-Montero^1^, Steven Michiels^1^, Sara Teruel Rivas^1^, Edmond Sterpin^2,3^, John Lee^1^

##### *^1^UCLouvain, Miro, Brussels, Belgium*

*^2^UCLouvain, Miro, Brussens, Belgium*

*^3^KULeuven, KULeuven, Leuven, Belgium*

**Background and purpose**: Patient selection for proton therapy (PT) over conventional radiotherapy (XT) currently entails comparing manually generated treatment plans, which requires time and expertise. We used deep learning (DL) models to directly predict for a given patient the optimal XT and PT dose distributions, which are then fed to Normal Tissue Complication Probability (NTCP) models to perform the selection in a fast and automatic manner.

**Material and methods**: Two databases of oropharyngeal cancer patients (60 PT and 90 XT patients) were used to train the XT and PT dose prediction models, based on a UNET architecture. The NTCP protocol used in the Dutch model-based approach, including grade II and III xerostomia and dysphagia, was later used to perform automatic patient referral. 48 patients were used to test our method.

**Results**: The patient selection based on the DL-predicted doses reached an accuracy of 87.5%: out of 48 patients, 42 received the same treatment indication when using DL predicted dose. Moreover, no overall NTCP increase in the population of patients (below 0.7% difference). A clear relation between side-effects passing the NTCP thresholds in predicted and planned doses was observed (83.7%).

**Conclusion**: Using DL dose prediction in combination with NTCP models for PT patient selection is feasible and can drastically save time and resources. They can help in the treatment choice and give estimation of patient specific side-effect probability. Moreover, DL models are easily transferable, sharing experience to centers that would not have PT planning expertise.

### O 149 - Fast, automated and high quality robust IMPT planning for oropharyngeal cancer patients

#### *Ilse Van Bruggen^1^, Roel Kierkels^2^, Mats Holmström^3^, Stefan Both^1^, Johannes Langendijk^1^, Erik Korevaar^1^*

*^1^UMCG, Radiotherapy, Groningen, Netherlands*

*^2^Radiotherapiegroep Deventer, Radiotherapy, Deventer, Netherlands*

*^3^RaySearch laboratories, Machine learning planning, Stockholm, Sweden*

**Objective**: The aim of this study was to achieve comparable or better plan quality than manual robust IMPT plans with a fast, artificial intelligence driven automated treatment planning method for robustly optimized IMPT plans for oropharyngeal carcinoma (OPC) patients.

**Method**: For new patients , an IMPT dose distribution was predicted using a Deep Learning Optimization DLO model which was trained using contours and doses of 60 OCPs. A robust mimicking optimization algorithm using voxel-based mimicking and 21 perturbed scenarios was then used to generate a machine deliverable plan from the predicted dose distributions. DLO validation and tuning was performed with 5 patients to tune prediction and mimicking settings. The independent test set consisted of 10 patients. Plans were considered clinically acceptable when robust target coverage and normal tissue doses were within the plan quality limits of table 1.

**Results**: All the DLO plans resulted in comparable or better plan quality as the manual plans. In the DLO plans the sum of NTCPs decreased with 2.5 and 0.9 percentage points for grade 2 and grade 3, respectively (table 2). DLO plans were generated including robustness evaluation in 27 +/- 3 minutes, offline time. Manual plan generation took around 2 to 3 days hands-on time per plan.

**Conclusion**: DLO automatically generates clinical acceptable robust IMPT plans for all (n=10) OPC patients and reduced treatment planning resources. DLO planning is therefore planned to be implemented in clinical practice.

### O 150 - Energy layer optimization for carbon ion arc therapy

#### Lennart Volz^1^, Marco Durante^1^, Christian Graeff^1^

##### ^1^GSI Helmholtz Center for Heavy Ion Research GmbH, Biophysics, Darmstadt, Germany

Carbon ion arc therapy (CART) yields potential to concentrate a high LET region within the tumor center, while mitigating range and LET uncertainty. Towards highly efficient arc delivery, we investigated novel approaches for CART at a single energy per field. CART plans spanning 38 angles separated at 5° were generated for a lung patient using the TRiP98 treatment planning software (Figure 1a). Heuristic energy selection utilizing a-priori information on the target water equivalent depth (WED) was investigated (Figure 1b): Firstly, the energies were selected to correspond to the central tumor WED (cWED). Secondly, a field was assigned its predecessors energy, if it would still hit the tumor, and the cWED otherwise (lWED). Finally, a novel optimizer was implemented: starting at the cWED, fields were iteratively assigned their neighbors' energy, if that lowered the voxel-wise variance of the dose correlation matrix without reducing the minimum (optWED). The cWED approach could not produce a reasonable target coverage (D95=87%;V95=84%; Figure 2a). The lWED approach improved dose homogeneity, due to the smearing out of the ions' stopping point within the tumor (D95=90%;V95%=89%; Figure 2b). The optWED approach improved conformity, at similar coverage as lWED (D95=89%;V95%=88%; Figure 2c). The cWET approach results in cold spots for partial arcs and is infeasible for non-spherical tumors. The new iterative optimization showed potential for deriving CART plans and is subject to further improvements. Interestingly, the lWED approach with only three energies reached equal quality as optWED. This indicates potential for few-energy CART, which could enable time efficient delivery.

### O 151 - Scenario-free probabilistic proton dose optimization using expected dose influence and total variance

#### Niklas Wahl^1,2^, Hans-Peter Wieser^1,2,3^

##### *^1^German Cancer Research Center - DKFZ, Medical Physics in Radiation Oncology, Heidelberg, Germany*

*^2^Heidelberg Institute for Radiation Oncology HIRO, Medical Physics in Radiation Oncology, Heidelberg, Germany*

*^3^Ludwig-Maximilians-Universität München, Department for Medical Physics, Garching, Germany*

**Introduction:** Robust and stochastic optimization are now established in particle treatment planning. However, mostly few error scenarios are used, since storing and processing the respective scenario dose matrices during optimization is computationally demanding. We propose a “meta-probabilistic” optimization method on the basis of a pre-computed mean dose matrix together with total variance matrices for each volume-of-interest (VOI), thus circumventing storage and runtime challenges when many error scenarios are desired.

**Methods:** For our approach, scenarios are computed during dose calculation but need not be maintained during optimization. Instead, we directly aggregate the mean dose matrix as well as their transposed matrix product to obtain a square matrix Ω for each VOI (compare Figure 2(d)). The computationally expensive sparse matrix products are executed on the GPU. In optimization, standard planning objectives (least-squares & DVH) are used, accompanied by minimizers of total variance, see Figure 2(e). All computations were implementend in the open-source treatment planning toolkit matRad.

**Results:** For a hypothetical liver SBRT treatment, conventional and robust plans are created with our meta-probabilistic and stochastic optimization with 25 (range & set-up) error scenarios. Figure 1 shows mean dose and standard deviation for the nominally, meta-probabilistically, and stochastically optimized plan. Figure (2) presents the corresponding DVHs. Meta-probabilistic optimization was 5x faster than stochastic optimization at comparable robustness.

**Conclusion:** Our meta-probabilistc approach facilitates probabilistic proton planning with a larger number of error scenarios, i.e., more accurate uncertainty estimates. Further, the use of total variance minimizers makes robustness a scalable objective in the underlying multi-criteria decision making problem.

### O 152 - Combined proton-photon therapy for head and neck cancer patients in presence of anatomical changes

#### Florian Amstutz^1,2^, Barbara Bachtiary^1^, Damien C Weber^1,3,4^, Antony J Lomax^1,2^, Jan Unkelbach^4^, Ye Zhang^1^

##### *^1^Paul Scherrer Institute, Center for Proton Therapy, Villigen PSI, Switzerland*

*^2^ETH Zurich, Department of Physics, Zurich, Switzerland*

*^3^University Hospital Bern, Department of Radiation Oncology, Bern, Switzerland*

*^4^University Hospital Zurich, Department of Radiation Oncology, Zurich, Switzerland*

The advantages of simultaneously optimized combined proton-photon therapy (CPPT), by integrating a fixed horizontal proton beam line (FHB) with a photon linac in one room, have previously been demonstrated for head & neck cancer (HNC) [Fabiano et al. 2020]. However, it is unclear whether these benefits persist under conditions of inter-fractional anatomical changes. A HNC case, consisting of a planning CT (*planCT*) and seven repeated CTs (*repCT*) acquired during therapy, was selected for assessment (Fig.1a). Simultaneous integrated boost plans were optimized using IMRT, IMPT-FHB, CPPT-FHB, and additionally CPPT-“Arc” assuming a fixed beam proton “Arc” treatment (e.g. upright patient rotation) (Fig.1b). Two treatment strategies were investigated: (I) Non-adaptive - plans optimized on *planCT* and then recalculated for each of the seven *repCTs*. (II) Adaptive - plans reoptimized on each individual *repCT*. DVHs, dose parameters and NTCP values for Xerostomia and Dysphagia were then evaluated and compared on daily structures (Fig.1d). Without plan adaption, dose degradation is pronounced for target and organs-at-risk for all four treatment scenarios, including CPPT-FHB, due to anatomical changes (Fig.2). With plan adaption however, the advantages of CPPT-FHB can be retrieved, leading to a reduction of ≈3% NTCP compared to IMRT. This could be further reduced using CPPT-“Arc” to ≈4-5%, due to its increased degrees of freedom and larger proton contribution in the combined plan. Plan adaption is necessary for clinical CPPT treatment of HNC. Increasing the flexibility of proton beam angles can result in slightly better CPPT plans, stressing the importance of finding the balance between cost-effectiveness and quality gain.

### O 153 - The algorithm using dual energy CT to find chemical constituents for accurate dose calculation of particle therapy

#### Donghyeok Choi^1^, So Hyun Ahn^2^, Kwangwoo Park^3^, Min Cheol Han^2^, Jin Sung Kim^2^

##### *^1^Yonsei University College of Medicine, Department of Medicine, Seoul, Korea Republic of*

*^2^Yonsei Cancer Center- Yonsei University College of Medicine, Department of Radiation Oncology, Seoul, Korea Republic of*

*^3^Yongin Severance Hospital- Yonsei University College of Medicine, Department of Radiation Oncology, Yongin, Korea Republic of*

**Purpose**: In this study, we tried a new approach to accurately find out the proportion of constituent elements of the human body material from dual energy CT and calculate SPR for proton and heavy particle therapy. This algorithm was developed using a simple theory that the total mass attenuation coefficient (MAC) of mixture or compound can be obtained by linear combination of MACs of constituent elements.

**Methods:** Our algorithm uses the MACs obtained from dual energy CT with Geant4 simulation as input, and finds the element of compounds or mixtures sequentially from atomic number 1 to 20 by using an optimization function. Using the 80/140 kVp energy spectrum combination, the constituent elements of nine biomaterials such as adipose and five compounds consisting of C, H, and O were found, and the results were compared with the value provided by the National Institute of Standards and Technology (NIST).

**Results:** As a result of algorithm test, the types of constituent elements were accurately found in all compounds and mixtures. And the proportion of constituent elements was accurately calculated within 6.03% for biomaterials and 2.98% for compounds compared with the values provided by NIST.

**Conclusions:** We verified that the algorithm developed in this study can find the constituent elements of a compound or mixture and their ratios using dual energy CT, and based on this, it is expected to improve the accuracy of SPR calculations of protons and carbon beams.

### O 154 - A knowledge-based planning tool for proton planning of brain tumours at treating and referring centres in Denmark

#### Bob Smulders^1,2^, Jailan Alshaikhi^3^, Liliana Stolarczyk^1^, Klaus Seiersen^1^, Ole Nørrevang^1^, Yasmin Lassen-Ramshad^4^, Morten Høyer^4^, Aida Muhic^2,4^, Anne Vestergaard^1^

##### *^1^Danish Centre for Particle Therapy DCPT / Aarhus University Hospital, Department of Medical Physics, Aarhus, Denmark*

*^2^University Hospital of Copenhagen / Rigshospitalet, Department of Oncology, Copenhagen, Denmark*

*^3^The Saudi Proton Therapy Center, Medical Physics Department, Riyadh, Saudi Arabia*

*^4^Danish Centre for Particle Therapy DCPT / Aarhus University Hospital, Department of Oncology, Aarhus, Denmark*

**Background/Purpose**: Proton radiation therapy has become increasingly the treatment of choice for brain tumours, due to the decreased integral dose to the brain and sparing of organs at risk. To produce a consistent quality of proton planning in Denmark, RapidPlan^TM^ (Eclipse^TM^,Varian Medical Systems) is used as a knowledge-based planning tool. This study compares the dose to the OAR generated by RapidPlan^TM^ with the original manual created treatment plans.

**Materials and methods**: The RapidPlan^TM^ brain model is based on 80 brain tumours manually-optimized dose distributions comprising prescription doses of 50.4, 54 or 59.4 GyRBE from patients treated at the DCPT. 10 patients of each prescription dose were re-planned with the same field/Range Shifter/Robustness arrangements using RapidPlan^TM^. In this model, the maximum dose to OAR are set manually, but the model produces automatic line constraints to reduce the dose throughout the OAR. The difference in mean doses of OAR from the clinical plan and RapidPlan^TM^ were compared.

**Results**: The plans produced by the model were comparable to the clinical plans on target coverage, robustness and maximum doses to the OAR. The difference in mean dose and range for all plans are presented in table 1. On average, the mean doses are comparable to the clinical plan and slightly in favour of the RapidPlan^TM^ model, especially for Chiasm, Optic Nerves and Pituitary.

**Conclusion**: The model produces treatment plans comparable to the clinical plans. This can optimize the workflow on the treating proton facility and possibly the workflow in the referring departments where proton/photon plan comparisons are made.

### O 155 - Evaluation of a proton knowledge-based auto treatment planning model for advanced nasopharynx cancer

#### Pingfang Tsai^1^, Casey Johnson^1^, Jack Tung^1^, Andy Shim^2^, Chavanon Apinorasethkul^2^, Haibo Lin^1^, Francis Yu^1^, Anna Zhai^1^

##### *^1^New York Proton Center, Physics, New York, USA*

*^2^New York Proton Center, Dosimetry, New York, USA*

**Purpose**: To evaluate the performance of a proton knowledge-based auto planning model for advanced nasopharynx cancer.

**Methods**: Forty nasopharynx patients with bilateral neck nodes and optic structures involvement were selected, and their treatment plans were used to train the RapidPlan module in the Eclipse treatment planning system (V 16.1). The model was evaluated by analyzing the goodness-of-fit summary statistics and identifying possible outliers. The established model was applied to five new patients with a single-round optimization in an average of 25 minutes. The RapidPlan plans (RPP) were compared to the clinical expert plans (CEP) in terms of target coverage and organs-at-risks (OARs) sparing to assess the model's performance.

**Results**: The coefficient of determination and the chi-squared of the training were 0.79±0.09 and 1.1±0.03. The RPP target achieved on average 96.5% on D_95%_ and a conformity index of 1.29. Decreases of the maximal dose by average 1.5Gy and 4Gy to cord and brainstem. The parotids sparing was an average 3Gy in mean dose on RPP. RPP also showed a 10Gy reduction in maximum dose under the best scenarios and, in general, a small deviation around 1Gy to the optics (optical nerves and chiasm) compared to CEP. Overall, the p-values from the *t*-test indicate no significant difference between the CEP and RPP.

**Conclusion**: The RapidPlan model generates competent clinical plans with comparable CTV coverage and superior or equivalent OAR sparing for complex nasopharynx cases.

### O 156 - A Deep Learning-based approach for statistical robustness evaluation in proton therapy treatment planning

#### Ivan Vazquez^1^, Mary Gronberg^1^, Xiaodong Zhang^1^, Laurence Court^1^, Ronald Zhu^1^, Steven Frank^2^, Ming Yang^1^

##### *^1^The University of Texas MD Anderson Cancer Center, Radiation Physics, Houston, USA*

*^2^The University of Texas MD Anderson Cancer Center, Radiation Oncology Department, Houston, USA*

Robustness evaluation is critical in particle radiotherapy due to its high susceptibility to uncertainties. However, the customary method for robustness evaluation only considers a limited number of uncertainty scenarios and fails to provide a clear and consistent statistical interpretation. We propose an AI-based approach that overcomes this limitation by predicting a set of statistically meaningful percentile dose values at every voxel and allows the evaluation of planning objectives at specific confidence levels. To achieve this, we built a deep learning model based on the 3D-UNet that leverages the benefits of dense connectivity and dilated convolutions. In this feasibility study, the model was trained to predict the 5th and 95th percentiles directly from the nominal dose distribution. The data consisted of proton radiotherapy plans from 543 prostate cancer patients. Ground truth percentile values were estimated for each patient using 600 dose recalculations representing randomly sampled uncertainty scenarios. We found an average gamma passing rate (2mm, 2%) of 99.08% and 99.05% and an average root-mean-square deviation of 0.70Gy and 0.74Gy by comparing the predicted and ground truth 5th and 95th percentiles of 107 test patients, respectively. This high degree of accuracy was also reflected by the close match between the predicted and ground truth dose-volume histograms of the bladder, rectum, femoral heads, and clinical target volumes. Predictions of both percentiles took ∼5s and bypassed the need for hundreds of dose recalculations. The proposed method produces accurate and fast predictions with intuitive statistical interpretation and presents a promising way to improve robustness evaluation.

### O 157 - Patient numbers and descriptive statistics during the first 17 months of a single-room proton center operating entirely during covid-19 pandemic

#### Michael Butkus^1^, Maytee Diaz-Williams^1^, Ailyn Hernandez^1^, Tatyana Santsevich^1^, Mariluz De Ornelas^1^, Josh Jardenil^1^, Tejan Diwanji^1^

##### ^1^University of Miami, Radiation Oncology, Miami, USA

**Purpose**: Present an operational metric for future centers based on a review of patient statistics for a first-year single-room proton center.

**Methods**: Retrospective analysis of patient statistics for a proton center commencing treatments in 09/2020 and extending through 02/2022. Facility is at an academic institution and is in full ownership (no 3^rd^ party liabilities) of proton equipment.

**Results**: 290 patients completed courses of treatment. A simple in-house derived “complexity unit” was used to track patient numbers. CNS (29%), Breast (21%), and H&N (20%) comprised the most treated complexity units. The average number of patients on beam was 22.7 with average complexity units of 29.3 and ending treatments on average at 6:47 p.m. The average age of patients was 53.0±17.2 (range: 13.2-98.0). Re-irradiation patients comprised 25.1% of treatments. Simulation to treatment time was, on average, 17.3 days. Due to machine downtime 14.1% of patients received at least one fraction on conventional linacs. Full-day downtime occurred on 14 occasions (3.9%), which was primarily due to shielding door mechanical malfunctions, not cyclotron or beam issues. Only 7.1% of patients did not complete their full prescribed course of radiation. 38.8% of patients did not miss any treatment days. Of those that missed treatment days, an average of 3.6 days were missed. Re-plans were required for 14.5% of patients, with a maximum of 4 re-plans for one patient.

**Discussion**: Review presented as a reference for future centers. Intrinsic and extrinsic factors would influence operating statistics of other centers.

### O 158 - The downstream “halo effect” of opening a single-vault proton therapy facility at a cancer center

#### Jacob Parzen^1^, Tracey Wilson^1^, Craig Stevens^1^, Prakash Chinnaiyan^1^, Peyman Kabolizadeh^1^

##### ^1^Beaumont Proton Therapy Center- Department of Radiation Oncology- Oakland University William Beaumont School of Medicine, Radiation Oncology, Royal Oak, USA

**Purpose/Objectives***:* There have been limited reports evaluating the effects of opening a proton therapy center on a radiation oncology department at a medical center.

**Materials and methods***:* The impact of a new single-vault proton center (PC) on patient volume at a medical center (MC) was evaluated from fiscal year 2016 (FY16) through FY19, representing the year prior to induction of the PC through the first 2.5 years of operation. Patient starts and fractions were obtained for our primary site (MC1), which shares a campus with our PC, and two additional sites (MC2 and MC3) on separate campuses.

**Results***:* Photon-based treatment starts at MC1 increased by 4% (*p* = 0.49) from FY16-FY19. Combined photon and proton starts at MC1/PC increased by 27% (*p* < 0.0001). The total combined number of photon starts at MC1, MC2, and MC3 increased by 11% (*p* = 0.002). Proton starts were 38 in FY17, 174 in FY18, and 191 in FY19. There was a cumulative 104% increase in HDR prostate implants from FY16-FY19 at MC1 (*p* = 0.0005), with PC consultations directly resulting in 69 HDR prostate implants in 36 patients. There was a 96% increase (from 25 in FY16 to 49 in FY19) in pediatric yearly case volume at MC1 from FY16-FY19 (*p* = 0.007).

**Conclusions***:* The opening of our single-vault PC has resulted in an approximate doubling in HDR prostate implants and pediatric treatment starts. Clinicians and administrators should be cognizant of this “halo” effect when balancing the costs and benefits of opening a particle-based therapy center.

## Poster Abstracts

### P 001 - In vitro effects of ionizing radiation on glioblastoma multiforme and high grade serous ovarian cancer cells

#### Marcelo Vazquez^1^, Antonella Bertucci^2^, Ann Morcos^1^

##### *^1^Loma Linda University, Radiation Medicine, Redlands, USA*

*^2^Loma Linda University, Radiation Medicine, Loma Linda, USA*

Glioblastoma multiforme (GBM) and high grade serous ovarian cancers (HGSOC) are two of the most aggressive cancers. The prognosis of these cancer patients is extremely poor. Current treatments are inadequate due to radio-resistance and relapse, urging the search for more effective therapies. Proton therapy offers numerous advantages in treating cancers compared to photon therapy. However, the biological effects of protons, may differ from those of photon radiation. Therefore, there is a need to study the cellular mechanisms of proton radiation on aggressive cancer cell lines. Here, we report the use GBM (T98G and LN18) and HGSOC (OVSAHO and OVCAR8) cell lines to characterize the effects of protons and photons in vitro. We hypothesize that cell lines have different radiation response, with protons being more effective in inducing cell damage. Cells were exposed to 0, 1, 2, 4 and 8 Gy of 250 MeV protons and 6 MeV photons to evaluate changes in cell proliferation, cell survival, cell cycle, apoptosis/necrosis induction, and DNA damage using immunocytochemistry. Results showed that both photons and protons induced a dose-dependent decrease of cell survival/proliferation and increase in foci formation, and apoptosis/necrosis as well as G2/M phase arrest across all the cells tested and that protons are more effective in inducing cell death. Overall, T98G is the more resistant among the GBM cell lines considered, and OVSAHO is the more resistant among the HGSOC cell lines used. These studies benchmark the basic radiation responses as part of ongoing studies to develop combined targeted therapy treatments.

### P 002 - A comprehensive method to design optimal cell irradiation with a scanning beam protontherapy machine considering LET dispersion

#### Natalia Chamorro Claver^1^, Pedro Arce Dubois^1^, Juan Ignacio Lagares^1^, Marta Ibáñez Moragues^1^, Miguel Ángel Morcillo Alonso^1^, Diego Azcona Armendáriz^2^, Marta Oteo Vives^1^, Alberto Viñals^2^, José Manuel Pérez Morales^3^

##### *^1^CIEMAT Center of Energy- Environment and Technology Research, Medical Applications of Ionizing Radiation Unit, Madrid, Spain*

*^2^Clínica Universidad de Navarra, Radiophysics and radiological protection, Madrid, Spain*

*^3^CIEMAT Center of Energy- Environment and Technology Research, Technology, Madrid, Spain*

**Introduction:** In the frame of the RADPROTIM project, we compare the irradiation with X-rays and an active scanning protontherapy machine at CUN of five cell lines. The first step is to optimize the treatment doing Monte Carlo simulations. We have not found in the literature any study that considers the LET dispersion in each cell well, despite it is widely recognised that this dispersion may hamper the understanding of the biological effect.

**Material and methods:** The design is based on placing a jig of material before the multiwell plates for cell culture to irradiate different points along the Bragg peak. The main constraints are a fix set of energies, a fixed beam width for each beam energy, a limited field of view, a minimum separation between spots, a maximum dose rate together with the need to limit the treatment time, a minimum cell well radius, the time it takes to seed the cells in each well, the precision of cell placements, the precision of dose and LET calculation of the Monte Carlo code. Factors to optimize: Beam initial width plus proton dispersion, maximum LET reached in a cell well, slope of the Bragg peak descent, and homogeneity of dose in one cell well, which will be better if the beam is wider

**Results and conclusion:** We selected a 70.2 MeV energy as the one who best optimize all the factors including LET dispersion and taking into account the constraints. The cells will be irradiated soon, and we expect the first analysis results by the conference.

### P 003 - A comparison of proton vs. photon planning in patients treated with Stereotactic Body Radiotherapy for spine metastases

#### Jody Beaumont^1^, Louise Dawdall^1^, Rachael Hall^1^, Matthew Lowe^1^, Rovel Colaco^2^

##### *^1^The Christie NHS Foundation Trust, Christie Medical Physics and Engineering, Manchester, United Kingdom*

*^2^The Christie NHS Foundation Trust, Proton Beam Therapy Centre, Manchester, United Kingdom*

**Introduction**: Spine Stereotactic Body Radiotherapy (SABR) uses high doses per fraction to treat vertebral metastases. However, proximity of the target volume to the spinal cord or cauda equina can result in significant compromise of coverage.

**Methodology**: Three patients treated under the NHS England Commissioning Through Evaluation (CTE) program for spinal metastases at our centre with photon volumetric arc modulated therapy (VMAT) were re-planned using pencil beam scanning proton therapy (PBT) to assess the potential benefits of PBT in this site. Patient selection: 1. Patient with metastatic breast cancer and a lesion involving L2 vertebral body and pedicle (Figure 1a); 2. patient with lung cancer and a lesion involving the spinous process at T12 (Figure 1b); and 3. patient with L3 lesion involving the L3 vertebral body (Figure 1c). Target volumes were contoured according to the International Stereotactic Radiosurgery (ISRS) guidelines and UK SABR Consortium dose constraints were adopted. Photon plans were created with two arcs for delivery using the 6MV SRS mode on a Varian Novalis Linear Accelerator fitted with high definition MLC. PBT plans used 2-3 fields with a Varian ProBeam beam model. The results of plan comparison are shown in Table 1 and Figure 1.

**Conclusion**: PBT is a potential treatment option for patients undergoing spinal SABR with comparable coverage to VMAT. Improved target dose homogeneity and superior anterior sparing can be seen with PBT, but steeper dose gradients are observed with photons. Further study is needed to evaluate the potential benefit of PBT in this situation.

### P 004 - Simultaneous integrated boost (SIB) vs. two phase planning with protons in paediatric/TYA posterior fossa ependymoma

#### Maeve Keys^1^, Frances Charlwood^1^, Anna France^2^, Russell Dawson^3^, Nicky Thorp^1^, Michelle Li^1^

##### *^1^The Christie NHS Foundation Trust, Clinical Oncology, Manchester, United Kingdom*

*^2^The Christie NHS Foundation Trust, Proton Clinical Outcomes Unit, Manchester, United Kingdom*

*^3^The Clatterbridge Cancer Centre NHS Foundation Trust, Clinical Oncology, Liverpool, United Kingdom*

**Introduction**: Post-operative proton therapy delivering a dose of 59.4Gy in 33 fractions to the tumour bed is the UK standard for completely resected paediatric ependymoma. For posterior fossa ependymomas, close proximity of brainstem and spinal cord often causes a reduction of target coverage for the final 5.4Gy. Dosimetric comparisons indicate that optimal target coverage and OAR doses may be achieved using SIB rather than 2 phases. This approach is also less resource intensive. The aim of this study is to retrospectively compare target coverage and doses to OARs for SIB vs. 2 phases.

**Materials and methods**: Paediatric and TYA patients with posterior fossa ependymoma treated at The Christie Proton Beam Therapy Centre were identified. Patients were treated with a 2 phase technique on the SIOP Ependymoma II trial or treated off trial with SIB technique. CTV coverage and doses for brainstem and spinal cord were compared using both trial and institutional planning parameters. T-test and Mann-Whitney U tests were used for significance testing.

**Results**: Twenty-two patients were identified, with median age of 4.5years. 12 were planned with 2 phases and 10 with SIB. CTV_59.4Gy mean, V98%, V95% were evaluated (table 1). CTV 59.4Gy mean was significantly improved between cohorts, 57.86Gy and 58.46Gy respectively (p=0.0499). Brainstem and spinal cord doses were compared with a trend across all parameters for lower doses, although did not reach significance (table 2).

**Conclusion**: CTV mean coverage was statistically better with at least equivalent OAR doses despite the small patient cohort and retrospective nature of this study.

### P 005 - Comparison of tumor control outcomes and dosimetry for craniospinal irradiation by treatment gantry

#### *Suvidya Lakshmi Pachigolla^1^, Alessandra Bolsi^1^, Claudio De Angelis^1^, Miriam Vazquez Varela^1^, Nicolas Sven Bachmann^1^, Frank Johannes Ahlhelm^1^, Alessia Pica^1^, Anthony John Lomax^1^, Damien Charles Weber^1^*

*^1^Paul Scherrer Institute, Center for Proton Therapy, Villigen, Switzerland*

**Introduction**: Craniospinal Irradiation (CSI) using pencil beam scanning has been delivered routinely to pediatric and adult cancer patients, using two treatment units (Gantry 1 (G1) and 2 (G2)). In this study, we evaluate the outcomes and dose metrics of CSI patients for G1 and G2.

**Methods**: We performed a retrospective review of CSI patients from 2004-2021. The median (range) prescribed CSI dose was 24 GyRBE (range,18–36.8), and 54 GyRBE (range,18-60.4) boost to the primary tumor. Fisher's exact test and Mann-Whitney-U were used for categorical and continuous data. Local control and distant control were estimated using the Kaplan-Meier method.

**Result**: Sixty-eight patients met inclusion criteria of which 19 (28%) and 49 (72%) patients were treated on G1 and G2, respectively. Median follow-up was 17 months (range, 0 -195). G1 patients were significantly younger than G2 patients (median, 4/9.3 years; Table 1). Additionally, the CSI and boost dose of G1 (35.2/19.8 GyRBE) was significantly different than G2 (23.4/30.6 GyRBE; Table 1). Eight (12%) patients experienced local failure and 13 (19%) patients distant failure. The estimated 2-year local control (Fig. 1A) and distant control (Fig. 1B) for G1 vs G2 was 74% vs 91% (p=0.273) and 78% vs 79% (p=0.887). The Planning Target Volume (PTV) V95% for the full CSI series for G1 and G2 patients was 91%(77%–95%) and 97%(83%–99%) (p<0.001) (Fig. 1C).

**Conclusion**: Although the clinical outcome of CSI patients treated with Gantry 1 and 2 was not statistically significant despite different patients' characteristics and delivered dose, better PTV coverage was achieved with Gantry 2.

### P 006 - ^11^C-Methionine and ^18^F-3,4-Dihydroxyphenylalanine PET-CT for target delineation in patients treated with proton therapy for primary and recurrent brain tumors

#### Jacobo Palma Delgado^1^, Martín Pastor Santiago M.^1^, Calvo Manuel Felipe A.^1^, Aguilar Redondo P. Borja^1^, Serrano Andreu Francisco Javier^1^, Vega Suárez Víctor Manuel^2^, Cabello García José Pablo^1^, Bejarano Herruzo Bartolomé^3^, Gallego Pérez de la Larraya Jaime^4^, Aristu Mendioroz José Javier^1^

##### *^1^Clínica Universidad de Navarra- Madrid., Radiation Oncology, Madrid, Spain*

*^2^Clínica Universidad de Navarra- Madrid., Radiology, Madrid, Spain*

*^3^Clínica Universidad de Navarra- Pamplona, Neurosurgery, Pamplona, Spain*

*^4^Clínica Universidad de Navarra-Pamplona, Neurology, Pamplona, Spain*

**Background:** ^11^C-Methionine Positron Emission Tomography (MET-PET) and ^18^F-3,4-Dihydroxyphenylalanine Positron Emission Tomography (DOPA-PET) may contribute to a more precisely delineation of the target volume. The use of PET planning images can lead to decrease neurological adverse effects in patients treated with Proton therapy (PT).

**Methods:** Between 04/2020 and 01/2022 17 patients diagnosed of primary or recurrent CNS tumors, with a median age of 52 years (range 25-74) were treated with PT. CT, MR and MET-PET/DOPA-PET images were utilised for radiation planning. The GTV-boost was generated using the MET-PET/DOPA-PET metabolically active regions (Figure 1) and the CTV boost were designed from the GTV-boost plus a 5mm of margin. The CTV no Boost were delineated using MR T2 FLAIR/T1Gad sequences.

**Results:** Nine (53%) were glioblastoma, 5 (29%) AA, 3 (18%) miscellaneous. Eight patients were reirradiated. The ratio between the PET delineated CTV-boost and the T1Gad generated CTV-boost was 0.7. The median prescribed dose was 60Gy RBE (35-60) and the median number of fractions was 25 (10-25). Immediate tolerance has been excellent and no acute toxicity grade 2 or higher was recorded.

**Conclusion:** The use of PET in CNS tumors saves a significant volume of healthy brain tissue from unnecessary irradiation. Patients requiring reirradiation are a remarkable model to explore metabolic imaging planning in PT. The use of hypofractionation is feasible and acceptable in terms of acute tolerance.

### P 007 - Reducing radiation-induced smell disturbance during pencil beam scanning (PBS) proton craniospinal irradiation (CSI)

#### Limin Song^1^, Victor Mangona^2^, Kaifang Du^3^, Shikui Tang^3^

##### *^1^TCPT, medical physics, Irving, USA*

*^2^Texas Center for Proton Therapy, Physician Group, Irving, USA*

*^3^Texas Center for Proton Therapy, Physics division, Irving, USA*

**Introduction**: Young patients receiving proton CSI (p-CSI) often experience significant radiation-induced smell disturbance (RISD) and nausea when delivering the whole brain fields using proton PBS, at times requiring cessation of treatment delivery. Interruptions during treatment prolong overall treatment time. Partial treatments result in adaptive plans to compensate for missing doses. In severe cases, patients require general sedation. We explore a technique to delay Olfactory Structure (OS) irradiation to the completion of brain-field delivery to minimize partial treatment.

**Materials and methods**: PBS gradient-matched p-CSI was planned in RayStation 6. Two brain-field delivery approaches were examined: (1) “Reverse-Energy-Layer” (low-to-high) delivery and (2) “Two-Segment” delivery. Reverse-Energy-Layer planning required export of beam layers, reversal of energy layers using an in-house script, and re-import. With Two-Segment delivery, the OS segment included all spots within 1 cm of the cribriform plate and olfactory nerves (extending just anterior to the brainstem) and was delivered second. The initial segment including all remaining brain spots and entire brainstem was delivered first. Anti-nausea/anti-anxiety premedication and olfactory stimulants (odors/mints) were used as needed.

**Results**: Both methods delay SD to the end of brain-field treatment. With Reverse-Energy-Layer delivery, beam time to the whole brain and OS were protracted due to longer energy layer switching. With Two-Segment delivery, the OS segment was delivered in < 20 seconds, and no SD has been experienced during delivery of initial segment.Conclusions: To mitigate RISD during PBS p-CSI, Two-Segment delivery delays OS irradiation to the end of beam delivery over a short duration, maximizing compliance with completion of treatment.

### P 008 - Separation effect and development of implantation technique of hydrogel spacer for prostate cancers

#### Nobuyoshi Fukumitsu^1^, Masayuki Mima^1^, Yusuke Demizu^1^, Takeshi Suzuki^2^, Takaki Ishida^3^, Kei Matsushita^3^, Raizo Yamaguchi^3^, Masato Fujisawa^4^, Toshinori Soejima^1^

##### *^1^Kobe proton center, Radiation oncology, Kobe, Japan*

*^2^Kobe proton center, Anesthesiology, Kobe, Japan*

*^3^International Clinical Cancer Research Center- Kobe University, Urology, Kobe, Japan*

*^4^Kobe University Graduate School of Medicine, Urology, Kobe, Japan*

The purpose was to improve the placement of a hydrogel spacer in prostate cancer patients receiving radiotherapy. One hundred and sixty patients with prostate cancer were classified into 3 groups (group 1, no spacer (n = 30); group 2, spacer placed using conventional technique (n = 100); group 3, spacer placed using new technique (n = 30)). When placing the spacer, the needle tip was placed at the middle of the prostate gland (group 2) or at a level corresponding to a cranial:caudal ratio of 6:4 and as close to the prostate gland as possible (group 3). The separation effect was compared. The separation in group 2 was larger than that in group 1 from the base to the apex (4 mm) level of the prostate, while the separation in group 3 was larger than that in group 2 from the middle to the apex (4 mm) level of the prostate. The separation values for the middle to the apex, the spacer thickness from the apex (10 mm) level to the apex, the rectal exclusion from the middle to the apex, and the laterality were correlated with the 50 and 60 Gray relative biological effectiveness (Gy(RBE)) rectal dose (*p* = 4.1 × 10^-9 ^– 0.046). The rectal dose is affected by separation, spacer thickness and rectal exclusion from the middle to the apex of the prostate and the laterality of the hydrogel spacer and can be further reduced by implanting a spacer on the caudal and the prostate side.

### P 009 - Maintaining local control tumor of head and neck paragangliomas with protontherapy

#### Julie Chartier^1^, Kim Cao^2^, Loïc Feuvret^3^, Philippe Herman^4^, Stéphanie Bolle^5^, Farid Goudjil^2^, Malika Amessis^6^, Olivier Choussy^7^, Remi Dendale^2^, Valentin Calugaru^2^

##### *^1^Institut Curie, Oncologie Radiotherapie, Paris, France*

*^2^Institut Curie, Oncologie Radiothérapie, Paris, France*

*^3^Hôpital Pitié-Salpétrière, Oncologie Radiothérapie, Paris, France*

*^4^Hôpital Lariboisière, Oto-Rhino-Laryngologie, Paris, France*

*^5^Institut Gustave Roussy, Oncologie Radiothérapie, Villejuif, France*

*^6^Institut Curie, Onocologie Radiothérapie, Paris, France*

*^7^Institut Curie, Oto-Rhino-Laryngologie, Paris, France*

**Purpose**: Head and neck paragangliomas are rare benign neuroendocrine tumors with local extension and a functional impact on essential intracranial organs. This monocentric study investigated the efficacy and toxicities of protontherapy in these tumors.

**Patients and methods**: Between 2001 and 2020, seventeen patients with head and neck paragangliomas received a treatment by protontherapy. Retrospected data on neurological, ear nose throat symptoms, ophtalmologic and audiometric testings were collected before and after treatment. The Common Terminology Criteria for Adverse Events (CTCAE) v5 was used to assess acute and late toxicities.

**Results**: Median dose used in this cohort was 50.4 Gy relative biological effectiveness (RBE, range 45.0-54.0 Gy). Median age at diagnosis was 54.6 years (range: 16.0–68.2 years). Protontherapy was used as an adjuvant treatment for five patients and as an exclusive one for twelve patients. Local tumour control rate was estimated at 100% (stability, n=17) after a median follow-up of 37,2 months. Functional benefit was reported in two patients while fifteen patients had hearing controlled symptoms (improved, n = 2, stabilized, n = 13). Protontherapy safety was confirmed with no acute toxicity above grade 2.

**Conclusion**: This prolonged follow-up supports the position of proton therapy as an effective and well-tolerated treatment option for local control of head and neck paragangliomas and control of functional symptoms.

### P 010 - Proton pencil beam scanning radiotherapy in the postoperative treatment of tonsillar cancer – evaluation of toxicity and effectivity

#### Jiri Kubes^1^, Silvia Sláviková^1^, Pavel Vítek^1^, Vladimír Vondráček^1^, Barbora Ondrová^1^, Alexandra Haas^1^, Kateřina Dědečková^1^, Michal Andrlík^1^, Matěj Navrátil^1^, Jozef Rosina^2^

##### *^1^PTC Prague, Proton therapy dept., Prague, Czech Republic*

*^2^Czech Technical University, Faculty of Biomedical Engineering, Prague, Czech Republic*

**Introduction:** Patients with tonsillar cancer (TC) have excellent prognosis and long life expectancy. Deintensification of therapy is discussed. Proton radiotherapy is one way to reduce radiation exposure and thus reduce acute and late side effects. The aim is to evaluate treatment outcome and toxicity of postoperative treatment with intensity modulated proton therapy (IMPT).

**Material and methods:** Between Sept 2013 and Jan 2021 46 patients were treated postoperatively for TC with IMPT. Median age was 54.1 years, 32 (69.6%) males and 14 (30.4) females. All patients had spinal cell cancer and surgery as primary treatment. Demographic parameters are in Table 1. Median of radiotherapy dose was 68 GyE in 34 fractions. Bilateral neck irradiation was used in 41 (89.1%) pts and unilateral in 5 (10.9%) pts. Concomitant chemotherapy was applied in 29 (63%). Median follow up time was 24 (1.5-62) months.

**Results**: Median follow up time is 34 (12-101) months. 3-years OS, DFS and LC were 97.1%, 97.2% and 100%. Acute and late toxicity are in Table 2. The most common acute toxicities were dermatitis and mucositis, with Grade 2+ in 80.4% and 76.1% of pts. No acute PEG insertion was necessary and intravenous rehydratation was used in 13% of pts. The most common late toxicity was xerostomy with Grade 1 in 58.7% of pts. and Grade 2 in 6.5% of pts. One patient developed late swallowing toxicity and became PEG-dependent.

**Conclusion:** IMPT for the postoperative treatment of TC is feasible with excellent efficiency and acceptable acute and late toxicity.

### P 011 - Comparison of two setup optimization approaches in ocular proton therapy at CNAO

#### Giulia Sellaro^1^, Andrea Pella^1^, Rosalinda Ricotti^1^, Maria Rosaria Fiore^2^, Agnieszka Chalaszczyk^2^, Gabriele Belotti^3^, Matteo Rossi^3^, Ester Orlandi^2^, Mario Ciocca^4^, Guido Baroni^1,3^

##### *^1^National Center of Oncological Hadrontherapy CNAO, Clinical Department - Bioengineering Unit, Pavia, Italy*

*^2^National Center of Oncological Hadrontherapy CNAO, Clinical Department, Pavia, Italy*

*^3^Politecnico di Milano University, Department of Electronics Information and Bioengineering, Milan, Italy*

*^4^National Center of Oncological Hadrontherapy CNAO, Clinical Department - Medical Physics Unit, Pavia, Italy*

In ocular proton therapy, the repeatability of setup is guaranteed by patient-specific immobilization tools and confiding on the capability of patients to maintain a steady view direction according to the treatment plan. For this purpose, a source of light (LED) is aligned on a patient specific base along the gaze direction. The Eye Tracking System (ETS) in use at CNAO features spherical passive markers on its surface, used as references during setup refinement. Our study aimed at comparing two different approaches for ETS positioning. We collected the clinical data of four patients treated at CNAO (two right, two left eyes). Dataset was increased to cover a range of dummy clinical-like polar and azimuthal angles. For each gaze directions, ETS was aligned using: (i) the standard clinical approach; (ii) the setup optimized approach in laboratory with a dedicated optical tracking system. For each alignment, we acquired a CT scan of the ETS to obtain the coordinates of passive markers. 3D distance of corresponding markers in the two reached ETS configurations was in median (interquartile) 2.81 (1.88) mm. We estimated a median difference (IQ) of -0.24°(0.93°) for polar and 0.21°(2.13°) for the azimuthal angles. Figure 1 summarizes the positioning of fixation light extracted from the ETS geometry in left and right eye cases. Further investigations will be devoted to minimize the residual misalignments. The optimization of setup geometry and ETS position in a dedicated area may produce a twofold advantage of an increased flexibility and releasing time in CT and treatment rooms.

### P 012 - Dosimetric feasibility of hypofractionation for pediatric small metastatic periobital lesions treated with and without aperture-enhanced PBS proton therapy

#### Witold Matysiak^1^, Enrica Seravalli^2^, Petra Kroon^2^, Xiaoying Liang^3^, Geert Janssens^2^, Stefan Both^1^, Johannes Langendijk^1^, John Maduro^1^

##### *^1^University Medical Center Groningen, Radiotherapy, Groningen, Netherlands*

*^2^University Medical Center Utrecht, Radiotherapy, Utrecht, Netherlands*

*^3^Mayo Clinic, Radiotherapy, Jacksonville, USA*

**Background**: Hypofractionation with protons facilitates the simultaneous treatment of multiple metastatic sites in combination with the primary location. This approach eliminates the need for separate conventional radiotherapy sessions to treat the metastatic sites, hence the first goal of this work was to explore if hypofractionation for these target types is dosimetrically feasible. The shallow location of the periorbital targets requires the use of range shifter and relatively low proton energies which results in bigger spot sizes that degrade lateral dose fall-off, so the second goal was to investigate if patient-specific apertures offer additional dosimetric advantages by sharpening lateral penumbra.

**Materials and methods**: To achieve both goals, we performed an in-silico study with 16 pediatric cases previously treated with photons. For each case we generated PBS two-field plans with and without patient specific apertures as well as four fractionation regimens: a conventional 20 fractions x 1.8 Gy/fx scheme and three hypofractionated schedules with equivalent EQD2 target coverage in 10, 5, and 3 fractions assuming α/β ratio = 10 Gy.

**Conclusions**: When considering hypofractionated regimens the risk associated with higher dose to brain (Fig. 2) must be weighed against the benefit of treating multiple metastatic sites in combination with the primary location. While the brain EQD2 was mostly determined by the fractionation scheme, the lateral penumbra enhancement provided by patient specific apertures improved conformality for conventionally fractionated plans, and in addition, improved SRS-specific plan metrics for hypofractionated schedules which provided significant sparing of neighboring OARs (see examples in Fig. 1).

### P 013 - Proton beam craniospinal reirradiation for patients with recurrent medulloblastoma

#### Natalia Martynova^1^, Nikolay Vorobyov^1,2,3^, Roman Frolov^1^, Denis Antipin^1^, Yulia Gutsalo^1^, Georgy Andreev^4^, Andrey Lyubinsky^4^, Ekaterina Smirnova^4^, Ekaterina Spiridenko^4^, Karina Safiullina^4^

##### *^1^Dr. Berezin Medical Institute MIBS, Proton Therapy Department, Saint-Petersburg, Russian Federation*

*^2^Saint-Petersburg State University, Medical Oncology, Saint-Petersburg, Russian Federation*

*^3^North-Western State Medical University named after I.I.Mechnikov, Medical Oncology, Saint-Petersburg, Russian Federation*

*^4^Dr. Berezin Medical Institute MIBS, Medical Physics, Saint-Petersburg, Russian Federation*

**Introduction**: Craniospinal irradiation (CSI) is an essential part of medulloblastoma treatment even in M0 stages. Recurrence with leptomeningeal dissemination needs reirradiation of the entire craniospinal axis. Craniospinal reirradiation is limited mostly because of its potential toxicity. Proton therapy may reduce toxicity and may help to apply potentially curative doses.

**Materials and methods**: Proton craniospinal reirradiation was conducted to 18 patients with recurrent medulloblastoma. Median dose to craniospinal axis was 30.6 Gy (24-36) with or without additional local boost. Median dose of primary CSI was 23.7 Gy (23.4-36). In all cases we've made a dose gradient in the border zone to avoid irradiation of vertebral bodies. Eight patients had High Dose Chemotherapy previously. Two patients already had craniospinal reirradiation with cumulative dose of 41.4 Gy for each patient.

**Results**: Median time between two CSI courses was 33 months (19-126). Median follow-up was 9 months (2-18). Six patients (33%) had disease progression. Average time to progression was 13.3±1.6 months (95% CI 10.1-16.4). During the treatment we observed strong evidence of progressing thrombocytopenia with minimal values (avg=95*10^9^) during third week (p<0.001). One patient had severe bone marrow hypoplasia during treatment which required blood and platelet transfusion and G-CSF injections. The rest of the patients' hematological toxicity resolved without medical intervention. None of the patients had any evidence of radiation necrosis or other radiological changes during the whole observation period.

**Conclusion**: Proton beam craniospinal reirradiation was well tolerated using the doses of 36 Gy to entire craniospinal axis.

### P 014 - Carbon ions radiotherapy for Sacral chordoma at Medaustron

#### Piero Fossati^1^, Eugen Hug^1^, Sarah Grosshagauer^2^, Mansure Schafasand^2^, Slavisa Tubin^1^, Antonio Carlino^2^, Markus Stock^2^

##### *^1^MedAustron Ion Therapy Center-, Radiation Oncology, Wiener Neustadt, Austria*

*^2^MedAustron Ion Therapy Center-, Medical Physics, Wiener Neustadt, Austria*

**Background***:* Carbon ion radiotherapy (CIRT) is used for the curative treatment of sacral chordoma. Sacral plexus neuropathy and sacral insufficiency fractures are the most relevant observed toxicities. Prescription dose, contouring and planning strategies are not yet perfectly defined.

**Material and methods***:* 20 patients have been treated with CIRT at MedAustron from July 2019 to September 2022. Prescription dose was 73.6 Gy RBE (LEM) in 16 fraction, for patients with macroscopic disease and 70.4 Gy RBE (LEM) x 16 fractions in operated patients. The corresponding mMKM total dose for non operated patient is 67.2 Gy RBE (mMKM). The plans optimized with LEM were recomputed with mMKM, moreover the LET distribution was examined.

**Results***:* Median follow up is short (5 months, range 1-31). There has been one local progression and one distant failure. The patient with local progression eventually passed away, the other 19 patients are alive. There has been one CTCAE G3 toxicity (subcutaneous abscess that had to be drained surgically). Dosimetric analysis has demonstrated adequate target coverage also in the MKM recomputed plans. With typically 95% of prescription covering CTV2. Rectum constraints were respected without compromising target coverage (also thanks to the use of surgically inserted spacers). LET was lower in the middle of large tumors. Beam arrangement played a major role in simultaneously achieving good target coverage with both RBE models.

**Conclusion***:* There is the need to specifically address number and orientation of beams to drive LET distribution in CIRT and achieve good plans with multiple RBE models.

### P 015 - Comparative in silico analysis of ultrahypofractionated preoperative proton versus photon therapy in adult soft tissue sarcoma

#### Rehema Thomas^1^, Hao Chen^2^, Emile Gogineni^2^, Aditya Halthore^2^, Bethlehem Floreza^2^, Temiloluwa Esho^2^, Arcelia Weaver^2^, Heng Li^2^, Curtiland Deville- Jr.^2^

##### *^1^George Washington University, School of Medicine, Washington, USA*

*^2^Johns Hopkins University School of Medicine, Department of Radiation Oncology and Molecular Radiation Sciences, Baltimore- MD, USA*

**Purpose**: Recent evidence has demonstrated the efficacy of ultrahypofractionated photon therapy (RT) for the preoperative treatment of soft tissue sarcoma (STS). Our purpose was to evaluate the dosimetric benefits of modern scanning proton therapy (PBT) compared to intensity-modulated RT (IMRT) for the neoadjuvant treatment of adult STS of the proximal lower extremity, considering this is the site at greatest risk for postoperative wound healing complications.

**Methods**: CT data from 11 consecutive, previously treated STS patients were used to create IMRT vs PBT plans. The prescribed dose was 30 Gy in 5 fractions. PBT doses are reported in GyRBE =1.1 Gy. Target coverage goals and organs at risk (OAR) constraints (Figure 1), conformity and heterogeneity indices, and integral dose were compared for IMRT vs PBT by student T-test with P<.05 considered significant.

**Results**: At least 99% CTV coverage and all OAR dose constraints were achieved for all plans (Figure 1). Mean doses to the femur (10.69±8.48 vs. 16.06±7.67 GyRBE), femoral head (3.65±4.41 vs. 6.66±6.37 GyRBE), and proximal joint (3.32±2.34 vs. 6.41±4.36 GyRBE) were all significantly lower with PBT vs. IMRT (P<0.05), as was the integral dose for each case (Figure 2) and overall. The conformity and heterogeneity indices were significantly better for PBT.

**Conclusion**: PBT maintained target coverage while significantly reducing dose to proximal OARs and integral dose to the body compared to IMRT, while also showing better conformity and heterogeneity indices. Further investigation is warranted to validate these dosimetric findings and potential clinical benefits in the management of adult STS.

### P 016 - Improving proton treatment for spinal chordomas and chondrosarcomas: a comparison planning study between sequential boost and simultaneous integrated boost technique

#### Michelle Li^1^, Peter Sitch^1^, Matthew Lowe^1^, Harriet Appleyard^1^, James Wylie^1^, Catherine Coyle^1^, Daniel Saunders^1^, Stephen Kennedy^1,2^

##### *^1^The Christie NHS Foundation Trust, Proton Therapy, Manchester, United Kingdom*

*^2^Lancashire Teaching Hospitals NHS Foundation Trust, Clinical Oncology, Preston, United Kingdom*

**Objective:** Spinal chordomas and chondrosarcomas are difficult to treat due to their proximity to the spinal cord and the high dose radiation required. Proton beam therapy (PBT) using sequential boost technique (SeqB) has been shown to be a safe and effective treatment for spinal sarcomas. There is increasing use of simultaneous integrated boost technique (SIB) PBT in other settings to improve target coverage and reduce dose to organs at risk (OAR). This comparative planning study aimed to investigate the potential dosimetric benefits of post-operative PBT using SIB compared with SeqB for patients with localised chordoma or chondrosarcoma involving the mobile spine.

**Methods:** Three patients treated with SeqB at our institution were randomly selected. SIB plans were constructed to deliver two dose levels, 70.2Gy(RBE=1.1) and 55.8Gy(RBE=1.1) in 39 fractions. The same dose constraints including spinal cord tolerances were employed to achieve the desired target coverage and normal tissue sparing. Dose to CTV and OAR were compared.

**Results:** In all cases, coverage of CTV (V95%, V98%) was better using SIB than SeqB (Figure 1). The spinal cord point maximum doses were similar in both approaches, however the BED was reduced in SIB compared with SeqB. Dose distribution in the SIB plans were more conformal and homogenous (Figure 2).

**Conclusion**: Based on our findings, SIB PBT could benefit patients with localised spinal sarcoma. Further work is needed prior to clinical implementation and followup to evaluate the translation into clinical outcomes.

### P 017 - Feasibility of dose escalation for non-small-cell lung cancer using proton therapy

#### Arno Hessels^1^, Sabine Visser^1^, Stefan Both^1^, Erik Korevaar^1^, Hans Langendijk^1^, Robin Wijsman^1^

##### ^1^University Medical Center Groningen, Radiation Oncology, Groningen, Netherlands

**Background**: Dose escalation (with 3D-CRT or IMRT) for NSCLC has been attempted previously but resulted in increased mortality, most likely due to increased radiation doses to organs at risk (OAR) such as the heart. The aim of this planning study was to determine whether intensity-modulated proton therapy (IMPT) based dose escalation is feasible in NSCLC without increasing OAR doses.

**Methods**: Robustly optimized and evaluated IMPT plans were created for 10 patients prescribing 73 Gy to IGTVpEsc (the internal gross tumor volume of the primary tumor ≥1 cm outside the mediastinal planning risk volume (mPRV)), 64 Gy to IGTVpPRV (the IGTVp overlapping the mPRV), and 60 Gy to the remaining CTV, in 25 fractions (Fig. 1). Dose maximum in the mPRV was restricted to 70 Gy. IGTVp and OAR (heart, esophagus, lung) doses were optimized and compared to clinical IMPT and VMAT plans prescribing 60 Gy to the entire CTV.

**Results**: All objectives on nominal and robust evaluation were met. For the escalated plans, doses to lung-GTV, heart and esophagus were similar to the 60 Gy IMPT plans and compared favorably to the 60 Gy photon plans (Fig. 2). Median V95% (of 73 Gy prescribed to IGTVpEsc) for IGTVp was 63% (IQR 46-77%), increasing for patients with an IGTVp lying mostly outside the mPRV.

**Conclusion**: Using IMPT, IGTVp-dose escalation in NSCLC is feasible without increasing dose to OARs compared to current clinical IMPT and VMAT plans. Further clinical validation of these findings requires an RCT.

### P 018 - Dosimetric evaluation and short-term outcomes of proton SBRT using pencil beam scanning for solitary NSCLC

#### Charles Shang^1^, Grant Evans^1^, Mushfiqur Rahman^1^, Timothy R. Williams^1^

##### ^1^South Florida Proton Therapy Institute, Radiation Oncology, Delray Beach, USA

**Purpose:** To evaluate dosimetric charateristics and short-term clinical efficacy of proton SBRT for solitary non-small cell lung cancer (NSCLC) using PBS and beam repainting technique.

**Methods and materials:** Retrospectively reviewed 26 patients of 58 to 93 years old, with a total of 32 lesions diagnosed as solidary NSCLC and treated with proton SBRT. 2 mm sliced average 4DCT set was used for proton planning with 3-5 mm/3.5% robustness, computed on Varian Eclipse planning system with AcurosPT algorithm. The resultant PTV's range from 8.2 to 172.7 cm^3^ being treated with two coplanar modulated proton scanning beams with one set repaint on a ProBeam Compact™ (Varian Medical, CA).

**Results and discussions:** The treated lesions include 13 (40.6%) centrally located and 10 (31.3%) for retreatments post local SBRT. 17 (53%) peripheral targets and favorable central lesions received 45 to 50 GyE in 5 consecutive fractions, while retreatments and remaining cases were delivered 35 to 40 GyE in 5 fractions. The mean follow-up time is 5.3 months. 11 (34%) patients showed grade ≤ 2 symptoms of pneumonitis. Each beam has 172 to 3250 proton spots and was delivered over 27.5 ± 9.6 seconds, corresponding to 6.3 ± 2.2 respiratory cycles. No local recurrency and severe adverse effects were identified within this serial.

**Conclusions:** Lung proton SBRT with pencil beam scanning for both centrally and peripherally located solitary NSCLC in this study appeared to be an effective modality with its satisfactory near-term local tumor control and low acute toxicities.

### P 019 - Patient specific breath hold approaches for treating moving target in pencil beam scanning (PBS) proton therapy

#### Limin Song^1^, Jared Sturgeon^2^, Kaifang Du^3^, David Cummings^4^, Shikui Tang^3^

##### *^1^TCPT, medical physics, Irving, USA*

*^2^Texas Center for Proton Therapy, Physician Group, Irving, USA*

*^3^Texas Center for Proton Therapy, Physics division, Irving, USA*

*^4^Texas Center for Proton Therapy, Dosimetry Group, Irving, USA*

**Introduction**: The treatment of targets with motion is a great challenge in proton therapy, especially for IMPT. Breath hold (BH) has shown to be an effective motion management approach. Here we present clinical data to investigate which BH technique is the best in a given clinical situation.

**Method**: The study focuses on patients with diseases in thorax and abdomen, whose 4DCT all indicate motion mitigation is clinically necessary or per physician's request. First, patients try deep inhale BH (DIBH) followed by BH at the end of exhale (EoE BH). If both are not feasible, they will repeat DIBH or EoE BH but with supply of oxygen. Once the optimal BH technique is chosen based on patient safety and comfort, BH duration and reproducibility, multiple BH CT scans are acquired. Robust optimization and evaluation are used during planning and repainting is utilized during beam delivery.

**Results**: Our preliminary results show all 10 patients currently collected tolerated their specific BH technique well. Seven patients were treated with DIBH, one with EoE BH plus oxygen and two without oxygen. Their daily CBCTs and verification CTs (VFCTs) showed good reproducibility of the target location. The clinic plans were still valid on the VFCTs.

**Conclusion**: BH with different techniques unique to a specific patient is effective for reducing target motion and mitigating interplay effect. Multiple factors should be considered carefully when choosing patient specific BH technique, e.g. patient safety and comfort, BH time and reproducibility, plan robustness, image verification during treatment, and duty cycle etc.

### P 020 - Predicting the impact of proton beam therapy technology on pulmonary toxicities for patients enrolled on the clinical trial NCT01255748

#### Gilmer Valdes^1^, Tomi Nano^1^, Jessica Scholey^1^, Efstathios Gennatas^1^, Nazir Mohammed^2^, Jing Zeng^3^, Rupesh D Kotesha^4^, Pranshu Mohindra^5^, Lane R. Rosen^6^, John Chang^7^, Henry K Tsai^8^, James J Urbanic^9^, Carlos E. Vargas^10^, Nathan Y Yu^10^, Lyle H Ungar^11^, Eric Eaton^11^, Charles B. Simone^12^

##### *^1^University of California- San Francisco, Radiation Oncology, San Francisco, USA*

*^2^North Western University, Radiation Oncology, Chicago, USA*

*^3^University of Washington and Seattle Cancer Care Alliance Proton Therapy Center, Radiation Oncology, Seatle, USA*

*^4^Miami Cancer Institute, Radiation Oncology, Miami, USA*

*^5^University of Maryland School of Medicine and Maryland Proton Treatment Center, Radiation Oncology, Baltimore, USA*

*^6^Willis-Knighton Medical Center, Radiation Oncology, Shreveport, USA*

*^7^Oklahoma Proton Center, Radiation Oncology, Oklahoma, USA*

*^8^New Jersey Procure Proton Therapy Center, Radiation Oncology, Somerest, USA*

*^9^California Protons Therapy Center, Radiation Oncology, San Diego, USA*

*^10^Mayo Clinic Proton Center, Radiation Oncology, Phoenix, USA*

*^11^University of Pennsylvania, Computer and Information Science, Philadelphia, USA*

*^12^New York Proton Center, Radiation Oncology, New York, USA*

**Purpose:** To predict the probability of grade ≥2 pneumonitis or dyspnea within 12 months of receiving proton beam therapy (PBT).

**Methods:** Consecutive patients enrolled and treated with PBT from 17 institutions were evaluated. Machine learning algorithms were used to predict the mentioned toxicities. Balanced Accuracy (BA) and Area under the Curve (AUC) were calculated. 95% Confidence intervals were obtained using bootstraps.

**Results:** In total, 965 lung cancer patients were analyzed. Patients had predominantly stage III disease or higher, were a median of 70 years old and 46.4% received concurrent chemotherapy. They were treated to a median prescription dose of 60 Gy and 2 Gy/fraction. 256 (28.2%) had grade ≥2 pulmonary toxicity. The most important demographic and dosimetric variables are shown in Table 1A and 1B together with the p-values comparing the populations defined by their cutoffs. The probability of pulmonary toxicity was 0.08 among the centers treating primarily with pencil beam scanning (PBS) and 0.34 from those treating with older techniques. Use of abdominal compression was also a highly significant predictor of less toxicity. As expected, higher total radiation delivered dose and dose to the ipsilateral lung increased the likelihood of pulmonary toxicities (See Table 1 B). When we combined demographic with dosimetric features, an AUC 0.75 was obtained, Figure 1.

**Conclusion:** Advanced machine learning methods have identified that proton treatment technique PBS, abdominal compression, and reduction of the dose received by the normal lung can lead to a significantly smaller probability of developing grade ≥2 pneumonitis or dyspnea.

### P 021 - Early toxicity in patients treated with ultrahypofractionated postoperative proton therapy for localized stage breast cancer

#### Roman Frolov^1^, Nikolay Vorobyov^1,2,3^, Nataliya Martynova^1^, Denis Antipin^1^, Yulia Gutsalo^1^, Georgy Andreev^1^, Andrey Lyubinsky^1^, Ekaterina Smirnova^1^, Ekaterina Spiridenko^1^, Karina Safiullina^1^

##### *^1^Sergey Berezin Medical Center, Radiation Oncology, Saint-Petersburg, Russian Federation*

*^2^North-Western State Medical University named after I.I. Mechnikov, Medical Oncology, Saint-Petersburg, Russian Federation*

*^3^Saint-Petersburg State University, Medical Oncology, Saint-Petersburg, Russian Federation*

**Purpose:** To report early toxicity and dosimetry data in breast cancer patients treated with ultrahypofractionated proton therapy (Ultra-HF-PT).

**Materials and methods:** Patients with localized breast cancer (Tis-T2, N0) who required postoperative whole-breast radiotherapy were eligible. The prescribed dose was 26 Gy in 5 daily fractions ± single fraction 5,2 Gy boost. Treatment planning was performed with 1 or 2 anterior fields (0°±40°) using multi-field optimization. For 69% of patients with left-sided cancer deep inspiration breath hold technique (DIBH) was used. The target coverage was defined as V_100%_>98% (>98% of volume receiving 100% of the prescribed dose). Toxicity was scored using CTCAE ver. 5.0.

**Results:** From July 2020 to November 2021, 63 patients were treated with intensity-modulated proton therapy. Median follow-up was 9 months (2 to 18). During radiotherapy Grade 1 and 2 dermatitis occurred in 45 (71%) and 4 (6%) patients respectively. After the end of treatment Grade 1 or 2 dermatitis occurred in all patients, resolving in 4 weeks.

Mean dose to the left anterior descending coronary artery (LAD) was 1,28 Gy (0,12-8,2) for left-sided breast cancer patients. Mean ipsilateral lung dose was 2,66 Gy (0,55-5,8). Compared to free breathing DIBH significantly reduced mean dose to the heart (0,4 to 0,1 Gy, p=0,03) and LAD (2,5 to 1,2 Gy, p=0,03) in patients with left-sided cancer.

**Conclusion:** Ultra-HF-PT in patients with localized breast cancer was well tolerated, with acceptable rates of skin toxicity, providing low dose to normal tissue without compromising target coverage.

### P 022 - A treatment planning study comparing IMRT and IMPT pseudo-brachytherapy boosts (SUPRA) to HDR brachytherapy for locally advanced cervical cancer

#### Shahed Badiyan^1^, Michael Watts^2^, Kenneth Walker^2^, Jessica Hilliard^2^, Yi Huang^1^, Michael Altman^1^, Matthew Schmidt^1^, James Kavanaugh^1^, Tianyu Zhao^1^, Jacqueline E. Zoberi^1^, Stephanie Perkins^1^, Stephanie Markovina^1^, Perry Grigsby^1^

##### *^1^Washington University School of Medicine St. Louis, Radiation Oncology, St Louis, USA*

*^2^Barnes-Jewish Hospital, Radiation Oncology, St Louis, USA*

**Purpose**: High-dose rate (HDR) brachytherapy for locally advanced cervical cancer (LACC) is limited by the spatial positioning of applicators. We hypothesized that an IMPT boost mimicking the internal dose-escalation of brachytherapy (pseudo-brachytherapy boost, SUPRA) improves tumor coverage and decreases dose to the bladder and rectum compared to HDR and IMRT SUPRA.

**Methods**: Plans were generated for eleven LACC patients treated at our institution. PET/CT-directed split pelvis IMRT was prescribed (20 Gy in 28 fractions to the cervix gross tumor volume (GTV), 50.4 Gy in 28 fractions to the pelvic lymph nodes). For boost plans, GTV and normal tissues were delineated on MRI from the first fraction of HDR. HDR prescription was 39 Gy in 6 fractions using tandem and ovoids. IMRT and IMPT SUPRA prescriptions were the low-dose rate radiobiological equivalent (65 Gy in 6 fractions) with internal dose-escalation to 165 Gy using 4 full arcs, and 6 MFO beams, respectively (Figure 1). Equivalent dose at 2 Gy (EQD2) was calculated to compare tumor and OAR dose from composite boost and split pelvis IMRT plans.

**Results**: The mean GTV EQD2 and mean GTV EQD2 D90% were greater for IMPT SUPRA and IMRT SUPRA than for HDR. Rectal EQD2 D2cc was lower for IMPT SUPRA as compared to HDR and as compared to IMRT SUPRA. The bladder EQD2 D2cc was not different between the three boost modalities (Table 1).

**Conclusions**: There may be dosimetric advantages to IMPT SUPRA compared to VMAT SUPRA or HDR for patients with locally advanced cervical cancer.

### P 023 - Intensity modulated particle therapy and intensity modulated radiotherapy utilization within the United States: a National Cancer Database 2018 analysis

#### Daniel Ebner^1^, Yasamin Sharifzadeh^1^, Jessica Burlile^1^, Benjamin Kamdem Talom^1^, Taylor Weiskittel^1^, Roman Kowalchuk^1^, William Breen^1^, David Routman^1^, Mark Waddle^1^, Robert Foote^1^

##### ^1^Mayo Clinic, Radiation Oncology, Rochester, USA

**Background**: Phase II/III clinical trials evaluating intensity modulated radiation therapy (IMRT) and intensity modulated proton therapy (IMPT) are ongoing. Utilization trends are unknown. The National Cancer Database (NCDB) was updated in 2018, providing new insight into current practice trends in the United States.

**Methods**: Data for all patients who received IMRT or IMPT for any disease site were collected from the National Cancer Database (NCDB) across all available years (2004 to 2018), with additional focus on proton centers reporting 50+ cases per year.

**Results**: The 995,372 intensity modulated (IM) cases identified accounted for 22.7% of total external beam radiation therapy (EBRT) cases. 3,105 (0.3%) utilized IMPT. Use of IM increased 5.5-fold from 2004 (20,963 cases) to 2018 (114,656). Few IMPT cases were reported prior to 2010 (2-16 per year). Approximately 60 cases per year were reported from 2011-2016, 174 in 2017, and 2,542 in 2018, which totals 2.2% of 2018 IM cases. Among all 2018 proton cases, IMPT was utilized in 14.2% of breast, 45.5% of head and neck, and 47.2% of prostate cases. Facilities treating 50+ proton cases in 2018 demonstrated variable IMPT utilization from 3.4% to 90.3% on a per-facility basis.

**Discussion**: Adoption of IMRT and IMPT increased from 2004-2018. In 2018, 2.2% of IM cases were delivered with IMPT. Among all proton cases, 40-50% used IMPT. Facility (in)ability to perform IMPT appears to limit technique penetrance. While technique utilization is anticipated to increase, IMPT trial results may need careful translation for non-IMPT centers.

### P 024 - Neutrophil to Lymphocyte ratio (NLR) as a predictor of outcomes in adenoid cystic carcinomas treated with carbon ion radiotherapy

#### Amelia Barcellini^1^, Giulia Fontana^2^, Maria Bonora^1^, Sara Ronchi^1^, Pierre Loap^1,3^, Maria Daria Filippini^4^, Guido Baroni^2,5^, Maria Rosaria Fiore^1^, Barbara Vischioni^1^, Ester Orlandi^1^

##### *^1^National Center for Oncological Hadrontherapy CNAO, Clinical Department- Radiation Oncology Unit, Pavia, Italy*

*^2^National Center for Oncological Hadrontherapy CNAO, Clinical Bioengineering Unit, Pavia, Italy*

*^3^Institut Curie, Department of Radiation Oncology, Paris, France*

*^4^IRCCS Azienda Ospedaliero-Universitaria Policlinico Sant'Orsola Malpighi-Bologna, Division of Medical Oncology, Bologna, Italy*

*^5^Politecnico di Milano, Department of Electronics- Information and Bioengineering DEIB, Milano, Italy*

**Background**: Neutrophil-to-lymphocyte ratio (NLR) is an emerging inflammatory biomarker widely studied in oncology as a potential predictor of treatment response and clinical outcome. However, its prognostic value in adenoid cystic carcinomas (ACCs) is still unknown. We aimed to assess the prognostic significance of baseline NLR in patients treated with carbon ion radiotherapy (CIRT).

**Material and methods**: We retrospectively retrieved the clinical and laboratory data of 49 consecutive ACC patients treated with CIRT (total dose: 57.6 GyE-68.8 GyE delivered in 12-16 fractions, 4 fractions/week) at the National Center for Oncological Hadrontherapy (CNAO, Pavia, Italy) between January 2015 and January 2021. The optimal cut-off value for NLR was defined. Univariate and multiple Cox regression analyses were performed to look for a potential association between NLR value and local control (LC) rate, metastasis-free survival (MFS), disease-free survival (DFS), or overall survival (OS).

**Results**: No significant association between NLR > 2.5 and LC, DFS, MFS and OS was found in univariate analysis; a trend was however reported for DFS (hazard ratio [HR] 2.10, p=0.11). In multiple Cox regression analysis adjusted for the absolute number of monocytes (ANM>0.4×10^9^/L), age>50 years, and margin status, NLR >2.5 was associated with a worse DFS (HR 3.70, p=0.01), but not with MFS, LC or OS. Univariate analysis reported better LC for patients with age > 50 (HR 0.21, p = 0.02).

**Conclusions**: Higher NLR was found to be associated with reduced DFS in ACCs patients treated with CIRT. Further studies are awaited to confirm these findings.

### P 025 - Spatially Fractionated Radiation Therapy (SFRT) using pencil beam scanning proton beams: LATTICE radiotherapy versus GRID radiotherapy

#### Mingcheng Gao^1^, Majid Mohiuddin^1^, Mark Pankuch^1^

##### ^1^Northwestern Medicine, Chicago Proton Center, Warrenville, USA

**Objectives**: LATTICE is a new type of 3-dimensional SFRT for bulky tumor. This study investigated LATTICE planning using protons, and compared its dosimetry with conventional SFRT (GRID).

**Methods**: Patient had a retroperitoneal liposarcoma (940cc). GRID plan used one field. LATTICE plans with one-, two-, four- and six-fields were created. Lattice of hexagonal closest packing was adopted and different orientations were studied. Organs within 1.5cm of tumor were grouped according to their location with respect to the GRID field: proximal, distal and lateral to tumor respectively. Robustness of SFRT was evaluated by shifting proton range by ±3%.

**Results**: Orientation of lattice is critical, best when coplanar vertices were minimized. 1-2 fields couldn't yield desired 3d dose falloff due to pileup of entrance doses. 6 equally-spaced fields achieved a good LATTICE plan with 62 vertices of 2.5cm apart. Mean tumor dose is 8.1Gy (GRID) and 4.7Gy (LATTICE). Peak tumor dose is 18Gy (prescription) for both. GRID gives higher dose (D0.5cc) than LATTICE to proximal organs (liver: 17.80Gy vs 3.34Gy, right-kidney: 17.19Gy vs 3.09Gy), but gives lower dose to distal organs (bowels: 1.05Gy vs 3.39Gy, left-kidney: 0.14Gy vs 3.24Gy) and to lateral organ (spinal cord: 0.45Gy vs 1.61Gy). Peak-to-Valley ratio of LATTICE is 4.76, but deteriorated to 3.23 with range uncertainty. Peak-to-Valley ratio of GRID is 4.17 and remains robust.

**Conclusions**: When a good beam entrance angle is available, proton GRID will deliver more dose to tumor and minimal dose to distal organs. If tumor is surrounded by critical organs, proton LATTICE helps to limit peripheral dose.

### P 026 - Financial toxicity in cancer patients undergoing proton therapy in Switzerland

#### Barbara Bachtiary^1^, Léonie Vivonne Grawehr^1^, Damien Charles Weber^1^

##### ^1^Paul Scherrer Institut, Center for Proton Therapy, Villigen, Switzerland

**Purpose**:This prospective observational cross-sectional study aimed to evaluate the out-of-pocket costs (OPCs) and the resulting financial toxicity of cancer care in Swiss patients undergoing proton beam therapy at the Center for Proton Therapy of the Paul Scherrer Institute (CPT/PSI) located in Switzerland which is high-income country.

**Methods**: One-hundred-forty-six Swiss patients participated in the study, of which 90 were adults with a median age of 48.5years (range,18-78) and 56 parents of pediatric patients with a median age of 9.5years (range,1-17).For all patients, the costs for proton therapy were entirely covered by health insurance.OPCs during PT were recorded with a diary that patients completed weekly.Financial toxicity was evaluated using the Comprehensive Score for financial Toxicity (COST) measure.The score ranges from 0 (financial ill-being/distress) to 44 (perfect financial well-being).Financial coping strategies were explored using polar questions (yes-no questions).

**Results**:The mean score for financial toxicity was 28.2(range,6-44) for all patients, 29(range,7-44) for adults, and 26.9(range,6-44) for parents of pediatric patients, respectively.In total, 34(24.7%)patients presented a COST figure<20 indicating financial distress.Due to increased financial burdens,42% of patients had to spend their savings, and, due to lack of savings, 10% had to borrow money. Of note,37% had to reduce their leisure activities, and 14% had to reduce their costs for food and clothing during treatment.

**Conclusion**:Despite having general health coverage in Switzerland, the COST questionnaire identified a substantial proportion of patients with financial toxicity after proton therapy. Additionally, a significant proportion of patients had to save money on food and clothing and were at higher risk of poverty.

### P 027 - Pediatric anesthesia for external beam proton therapy: local experience

#### Mikhail Cherkashin^1^, Alexey Nikolaev^2^, Nikolay Vorobyov^3^, Natalia Berezina^4^, Albina Gokoeva^2^, Fatima Valieva^2^, Konstantin Boiko^5^

##### *^1^Medical Institute n.a. Berezin Sergey, Surgery, St Petersburg, Russian Federation*

*^2^Medical Institute n.a. Berezin Sergey, Intensive Care, St Petersburg, Russian Federation*

*^3^Medical Institute n.a. Berezin Sergey, Radiation Oncology, St Petersburg, Russian Federation*

*^4^Medical Institute n.a. Berezin Sergey, Radiology, St Petersburg, Russian Federation*

*^5^Medical Institute n.a. Berezin Sergey, Pediatric Oncology, St Petersburg, Russian Federation*

**Purpose**: To discuss anesthesia management for proton beam therapy in pediatric oncology.

**Methods**: From 2018 – 2021, 876 children with CNS and body tumors received proton therapy in our hospital. In 360 cases, radiation treatment was delivered with anesthesia. Mean fractions number was 34 (anesthesias number - 34 for 1 patient). The mean age was 6 years (6 months - 17 years). 76% of patients had CNS tumors, 9% lymphomas, 15% soft tissue and other body tumors. Five children with stem tumors received sevofluran anesthesia with mechanical ventilation, others received propofol with nasal oxygenation. In all patients ports or PICCs were used. For treatment time optimization, revolver-style anesthesia was implemented: The first team woke children after treatment, the second team provided anesthesia during treatment and in parallel, the third team started sedation in the anesthesia room, then all teams moving for new patients.

**Results:** The mean number of patients receiving anesthesia numbered 23 (20-32) per day. In 2 stem tumors patients during propofol anesthesia, desaturation was revealed and mechanical ventilation started. In 24 children, radiation treatment was interrupted due to radiation-induced hydrocephalus; patients were transferred to the neurosurgery department for ventriculoperitoneal shunting and started again with radiotherapy. In 56 patients (15.5%), due to tumor decreasing and strong clinical psychologist support on 15-20 fractions, we were able to refuse anesthesia and children continued proton therapy without sedation.

**Conclusion**: Implementation of pediatric anesthesia during proton therapy at our hospital is characterized by strong infrastructure creation (anesthesia rooms, awakening wards, patient trolleys with oxygen, monitoring and ventilator etc). For optimal treatment and clinical room time management it is critical to implement revolver-style patient anesthesia.

### P 028 - Comparative planning and Letter of Medical Necessity in head and neck cancer: implementation and impact of a resident physician-led program

#### Vishal Dhere^1^, Erica Swinney^1^, Subir Goyal^2^, Amy Breakstone^1^, Mark McDonald^1^

##### *^1^Emory University, Radiation Oncology, Atlanta, USA*

*^2^Emory University, Biostatistics Shared Resource- Winship Cancer Institute, Atlanta, USA*

**Purpose**: Proton insurance authorizations in the US often receive an initial denial. The appeals process can lead to delays in care. We created a voluntary, compensated extra duty program for our resident physicians to assist in timely generation of comparative treatment plans and letters of medical necessity (LOMNs) using a standardized methodology, and report on the impact of this “moonlighting” program.

**Methods**: Residents approved for moonlighting previously demonstrated essential clinical competencies in head and neck (H&N) radiation and completed individual training to evaluate anticipated benefits of proton versus photon treatment plans using a battery of NTCP models assessing risk of high-grade mucositis, dysphagia, aspiration, xerostomia, and hearing loss as well as trismus, hypothyroidism, and secondary malignancy.

**Results**: From program start in February 2020 through November 2021, 410 H&N proton authorizations were initiated. Prior to the program, the average time between radiation oncology consultation and completion of a H&N LOMN was 9.6 days, reduced to 5.2 days with moonlighting (p=0.001). In the 6 months prior to the program, an average of 16.7 H&N authorizations were submitted per month compared to 20.5 after the program (p=0.065). The ultimate H&N proton insurance approval rate was 88.7% before and 92.3% with the program (p=0.172).

**Conclusion**: A resident physician-led program using a consistent evaluation of the anticipated benefits of proton therapy reduced the time to completion of a LOMN, achieved a high rate of approvals, and demonstrated a trend towards increased capacity to seek more authorizations. We subsequently expanded this approach to other disease sites.

### P 029 - Indications, treatment algorithms, innovative strategies and early data following Carbon ion Radiotherapy (CIRT) at the MedAustron Ion Therapy Center

#### Piero Fossati^1^, Eugen B. Hug^1^, Petra Georg^1^, Antonio Carlino^2^, Markus Stock^2^

##### *^1^MedAustron Ion Therapy Center-, Radiation Oncology, Wiener Neustadt, Austria*

*^2^MedAustron Ion Therapy Center-, Medical Physics, Wiener Neustadt, Austria*

CIRT is available only in few centres worldwide. Since July 2019, 240 patients were treated with CIRT at MedAustron. Initially we demonstrated safety and reproducibility based on dose/fractionation protocols established in Germany and Japan (correcting prescription doses and OARs constraints to account for different RBE models according to the Italian approach). The most common indications have been Re-Irradiation (39%), non-SCC H&N Ca (28%), Sarcomas (27%) and Prostate Ca (10%). For skull base and paraspinal sarcoma we selected the German fractionation whereas for other sarcoma we used the more aggressive Japanese hypofractionation. Reirradiation was performed with moderate hypofractionation and 3-4.8 Gy(RBE) dose per fraction. For H&N Ca without elective node irradiation (ENI) we used exclusive CIRT according to Japanese schedule. In cases of ENI we treated ENI with low LET RT (protons for unilateral and photons for bilateral ENI) followed by CIRT boost. In the last 2 years we introduced innovative treatment concepts: a) extreme hypofractionation for selected reirradiation and oligometastatic patients; b) high-dose CIRT salvage for patient with tumour progression during low LET irradiation; c) routine use of combining protons (CTV) with CIRT boost (GTV); d) CIRT for high-risk Prostate CA including ENI.; e) Start of a clinical trial of Partial Tumour Irradiation with CIRT to elicit antitumoral immune response. We will report on early toxicity and outcome.

### P 030 - Proton radiation therapy utilization within the United States: A NCDB 2018 analysis

#### Yasamin Sharifzadeh^1^, Daniel Ebner^1^, Benjamin Kamdem Talom^1^, Taylor Weiskittel^2^, Roman Kowalchuk^1^, William Breen^1^, David Routman^1^, Mark Waddle^1^, Robert Foote^1^

##### *^1^Mayo Clinic- Rochester, Radiation Oncology, Rochester, USA*

*^2^Mayo Clinic- Rochester, Molecular Pharmacology & Experimental Therapeutics, Rochester, USA*

**Background:** Proton radiation therapy (RT) was approved by the US Food and Drug Administration (FDA) in 1988. In 1990, the first hospital-based proton center opened in California. Over the next 18 years, 30 more proton centers with 87 gantries were opened, evolving from passive scatter to pencil beam scanning facilities. Although the proliferation of proton centers is evident, the patterns and extent of proton RT utilization across the United States remains unclear.

**Methods:** Data for all patients who received any form of RT to any disease site were collected from the National Cancer Database (NCDB) across all available years (2004 to 2018). Radiation data was further divided into radiation modality and type, including proton RT.

**Results:** The percent of cases receiving proton RT within the NCDB increased from 0.5% in 2004 to 2.2% in 2018. This represents a six-fold increase in absolute case numbers (1241 to 7571 cases). There was a simultaneous increase in all external beam radiation therapy (EBRT) cases (n = 247,150 in 2004 to 347,539 in 2018 total). Proton RT accounted for 6% of the total increase in EBRT cases. The largest increases in proton utilization were seen in breast (178 to 1835 patients, 10.3x), CNS (55 to 767, 13.9x), head and neck (63 to 855, 13.6x), lung (89 to 835, 9.4x), prostate (616 to 1271, 2.1x), and upper GI (13 to 273, 21x) malignancies.

**Discussion**: From 2004 to 2018, proton radiation therapy utilization increased six-fold and accounted for 6% of the total increase in EBRT use nationwide.

### P 031 - Acceptance testing and commissioning of two multiple room proton units in China: issues and lessons learned

#### Yuanshui Zheng^1^, zuofeng li^1^, Hualing Lv^2^, Yajun Jia^1^, Yifeng Yang^1^, Zhangmin Li^1^, Li Shen^1^, xuewen chang^3^, Taize Yuan^1^

##### *^1^Guangzhou Concord Cancer Center, Radiation Oncology, Guangzhou, China*

*^2^Varian, Installation, Guangzhou, China*

*^3^IBA, Installation, Guangzhou, China*

**Purpose**: The purpose of this abstract is to present issues and lessons we have learned during acceptance testing and commissioning for two multiple room proton units at the Guangzhou Concord Cancer Center in China.

**Methods**: Two multiple room proton units, the IBA Proteus Plus with 3 Gantry rooms and 2 fixed beam rooms, and the Varian ProBeam with 4 gantry rooms, are being installed at our center. Starting from when the beam was available in treatment room, we have arranged physicists to shadow the vendor team and actively participate for beam tuning and testing (e.g. 24 hours a day for a period of time), and record progresses and issues as they arise. Also, we have arranged multiple training modalities as early as possible to prepare the physics team, including journal club, proton lectures, institution provided planning training, vendor training remotely and onsite.

**Results**: Currently all physicists have been trained and are able to perform basic proton planning. Acceptance testing is completed for one IBA gantry room and close to completion with one Varian gantry room. Many issues and solutions during beam tuning and acceptance testing as well as training have been recorded, and will be used for future room acceptance testing and commissioning. Type testing for the Varian proton unit is scheduled to start in March.

**Conclusions**: Acceptance testing and commissioning two multiple room proton units at one center are challenging and require careful planning. Our issue database and experience could be helpful for other proton center acceptance and commissioning.

### P 032 - Characterization of a 2.7x2.7 cm2 particle beam monitor based on a silicon detector segmented in 146 strips

#### Mohammed A. A. Abujami^1,2^, Emanuele Data^1,2^, Sara Garbolino^2^, Simona Giordanengo^2^, Oscar Ariel Martì Villarreal^1,2^, Felix Mas Milian^1,3^, Anna Vignati^1,2^, Roberto Cirio^1,2^, Vincenzo Monaco^1,2^, Roberto Sacchi^1,2^

##### *^1^University of Turin, Department of Physics, Turin, Italy*

*^2^National Institute for Nuclear Physics INFN, Turin division, Turin, Italy*

*^3^Universidade Estadual de Santa Cruz, DCEt, Ilheus, Brazil*

**Purpose:** Design and characterization of a prototype detector developed to monitor therapeutic proton beams online.

**Materials and methods**: Thin silicon sensors were developed, characterized and used in a prototype detector aiming to discriminate and count single protons or carbon ions of therapeutic beams at a maximum fluence rate of 10^8^ p/(cm^2^*s) with an uncertainty of less than 1%. The sensor is segmented in 146 strips with a sensitive area of 2.7×2.7 cm^2^ to cover the beam cross-section of about 1 cm FWHM at the isocenter. The detector is read out by six custom ASICs housed on a dedicated frontend board connected to 3 FPGAs (Xilinx Kintex7). A LabVIEW program is used to display online counting rate of each strip and to store the data. An extensive characterization was performed first in the laboratory with a pulse generator and then with proton beams at the Italian National Center of Oncological Hadrontherapy (CNAO).

**Results:** Data were collected and analyzed at different beam energies and intensities. The beam profile was studied by measuring the number of protons as a function of the strip number and fitting the corresponding distribution with a Gaussian (Figure 1). The beam FWHM measured for different energies is compared with expected values at the isocenter (Figure 2).

**Conclusions:** The tests performed on synchrotron beams proves the feasibility of directly measuring the particle rate during treatments. Further studies towards using this technology for beam monitoring in clinical practice require improving the radiation resistance, using finer segmentation and increasing the detector sensitive area.

### P 033 - Compact rotating gantry mounted with superconducting magnets for East Japan Heavy Ion Center

#### Takashi Yazawa^1^, Masaki Asano^1^, Yasuhiko Sugimoto^1^, Yasushi Iseki^1^, Sinya Matsuda^1^, Takayama Shigeki^2^, Yutaka Hirata^1^, Hikaru Souda^3^, Takeo Iwai^3^, Kosuke Nakanishi^4^

##### *^1^Toshiba Energy Systems & Solutions Corporation, Power Systems Div., Kawasaki, Japan*

*^2^Toshiba Energy Systems & Solutions Corporation, Energy Systems Research and Development Center, Yokohama, Japan*

*^3^Yamagata University, East Japan Heavy Ion Center- Faculty of Medicine, Yamagata, Japan*

*^4^Toshiba Energy Systems & Solutions Corporation, Particle Radiotherapy Business Project Engineering Department, Kawasaki, Japan*

This paper describes the design and test result of the world's most compact rotating gantry for carbon-ion therapy, which has been successfully installed at the East Japan Heavy Ion Center (EJHIC), Faculty of Medicine, Yamagata University, Japan. Therapeutic irradiation flexibility from any angle in 360 degrees is quite common in photon and proton therapy systems. However, a carbon-ion therapy system with this capability would require a substantially large mechanical structure due to the greater bending radius of carbon-ion beams. Toshiba has incorporated two cutting-edge technologies for downsizing the rotating gantry structure. The first is mounting superconducting combined function magnets in the beam line on the gantry, which results in a stronger magnetic field, and therefore a substantial decrease in the bending radius of the beams. The second is a sophisticated structure of the scanning magnet for the carbon-ion beams, which has shortened the distance from the scanning magnet to the iso-center. As a result, this compact superconducting rotating gantry is downsized to 2/3 of the original rotating gantry that was installed at QST Hospital. The compact rotating gantry was installed at EJHIC, followed by the successful completion of the beam adjustment in 360 degrees and the final acceptance testing. The rotating gantry treatment room is under clinical commissioning for the commencement of the clinical treatment (as of Feb., 2022). It is also noted that this type of compact rotating gantry will be installed in two on-going heavy-ion therapy projects in Korea.

### P 034 - Converging proton minibeams with magnetic fields for opti-mized radiation therapy: a proof of concept

#### Marco Cavallone^1^, Yolanda Prezado^2^, Ludovic De Marzi^1^

##### *^1^Institut Curie, Orsay Proton Therapy center, Orsay, France*

*^2^Institut Curie, PSL university, Orsay, France*

Healthy tissue tolerance to radiation is one of the main limitations in radiotherapy treatment. To widen the therapeutic index, innovative approaches using non-conventional spatial and temporal beam structure are currently being investigated. Among them, proton minibeam radiation therapy is a promising solution combining the benefits of minibeam radiation therapy with the more precise ballistics of protons to further optimize the dose spatial fractionation and reduce radiation side effects. The purpose of this study is to investigate possible strategies to couple pMBRT with dipole magnetic fields to generate a converging minibeam pattern and increase the center-to-center distance between minibeams at shallow depth. Magnetic field optimization was performed so as to obtain the same transverse dose profile at the Bragg peak position as in a reference configuration with no magnetic field. Monte Carlo simulations reproducing realistic pencil beam scanning settings were used to compute the dose in a water phantom. We analyzed different minibeam generation techniques, such as the use of a static multislit collimator or a dynamic aperture, and different magnetic field positions, i.e. before or within the water phantom. For some configurations we obtained an increase of peak-to-valley dose ratio and decrease of valley dose at shallow depth above 50%, which indicates that magnetic fields can be effectively used to improve the spatial modulation at shallow depth for greater healthy tissue sparing. 1. Cavallone M, Prezado Y, De Marzi L. Converging Proton Minibeams with Magnetic Fields for Optimized Radiation Therapy: A Proof of Concept. Cancers (Basel). 2021 Dec 22;14(1):26.

### P 035 - Beam commissioning of Superconducting Cyclotron (SC230) for proton therapy

#### Takehisa Tsurudome^1^, Takuya Miyashita^2^, Hiroshi Tsutsui^3^, Yuta Ebara^1^, Jun Yoshida^1^, Shu Nakajima^2^, Shuhei Hara^2^, Toshiki Tachikawa^2^, Yukio Mikami^1^, Yukio Kumata^1^

##### *^1^Sumitomo Heavy Industries- Ltd., Technology Research Center, Yokosuka, Japan*

*^2^Sumitomo Heavy Industries- Ltd., Industrial Equipment Division, Niihama, Japan*

*^3^Sumitomo Heavy Industries- Ltd., Industrial Equipment Division, Tokyo, Japan*

Superconducting Cyclotron (SC230) for proton therapy has been developed at Sumitomo Heavy Industries, Ltd.. The cyclotron weight is about 65 tons, and a diameter is 2.8 meters. A power consumption is less than 250 kW. It was designed to meet various clinical needs with a beam energy of 230 MeV and a maximum current of 1000 nA. Such high beam current enables to realize very short irradiation time, and it can be also applied for FLASH radiotherapy. The RF system was confirmed to perform at its design value of 95.2 MHz frequency and 50 kV DEE voltage in 2020. Based on the results, beam commissioning tests have been started. By the end of 2021, the maximum beam current of 1000 nA, extraction efficiency of over 63 %, beam position stability of less than 0.1 mm and the power consumptions of 221 kW were confirmed through the beam commissioning. These results proved that SC230 has sufficient performance of various clinical needs. In addition, in order to reduce down time, maintenance of cryocoolers while maintaining the cooling coil temperature also have been developing by applying our similar technology as other superconducting magnet applications. In this paper, the development results of the SC230 are presented.

### P 036 - High-gradient magnetically focused planar proton minibeams

#### Grant McAuley^1^, Anthony Teran^2^, Eric Ramirez^1^, Jerry Slater^1^, Andrew Wroe^1,3^

##### *^1^Loma Linda University, Radiation Medicine, Loma Linda, USA*

*^2^Orange County CyberKnife and Radiation Oncology Center, Radiation Oncology, Fountain Valley, USA*

*^3^Miami Cancer Institute, Radiation Oncology, Miami, USA*

**Introduction:** This research describes the dosimetric properties of proton mini beams generated experimentally using high-gradient quadrupole Halbach cylinders.

**Methods:** Proton pencil beams of initial diameter 10 mm and with a range in water of approximately 10, 15 and 20 cm, were created in a clinical passive scattering nozzle and focused by two quadrupole magnets into planar beamlets. The magnets consist of 24 segments of radiation-hard Sm2Co17 adhered into Halbach cylinders with a 10 mm bore diameter, and lengths and field gradients of 40 mm and 191 T/m, and 68 mm and 251 T/m, respectively. Beamlet depth and transverse dose distributions were measured in a water tank using a diode detector and EBT3 film. Dose distributions composed of three beamlets were created by shifting the water tank with respect to the beam axis using a beamlet center-to-center (ctc) separation chosen to produce quasi homogeneous transverse dose profiles at target depth (Table 1 and Figure 1). Modulated composite beams were also investigated.

**Results:** Composite dose distributions showed quasi homogeneous dose at target depths and a high degree of spatial fraction at entrance consisting of widely separated narrow planar beamlets (Figure 1). At entrance depth, FWHM of the central beamlet of the unmodulated composite distributions were 1.6, 1.3, and 1.3 mm, and PVDR were 19, 53, and 66, for the 127, 157 and 186 MeV beams, respectively (Table 1).

**Conclusion:** Our experiments serve as proof of concept that high-gradient quadrupole magnets can create spatially fractionated proton minibeams suitable for further pre-clinical investigation.

### P 037 - Micron-scale dosimetry for radiobiological studies at the Holland Proton Therapy Center

#### Celebrity Groenendijk^1^, Marta Rovituso^2^, Danny Lathouwers^1^, Wouter Van Burik^2^, Linh Tran^3^, Anatoly Rosenfeld^3^, Jeremy Brown^1,3,4^

##### *^1^Technical University Delft, Radiation Science & Technology, Delft, Netherlands*

*^2^Holland Proton Therapy Center Delft, Research & Development, Delft, Netherlands*

*^3^University of Wollongong, Centre for Medical Radiation Physics, Wollongong, Australia*

*^4^The Australian Nuclear Science and Technology Organisation ANSTO, Detection and Imaging, Sydney, Australia*

The lack of knowledge regarding DNA damage induction and involved repair pathways from proton and photon irradiation in cancer patients presents a clinical challenge during patient treatment selection. A greater fundamental understanding of these processes on the molecular and cellular level is required to develop a patient specific and biologically informed radiotherapy treatment selection approach. This work aims to combine biophysical modelling with experimental measurements to investigate dose-weighted mean lineal energy (yD) values across and through a passively scattered 10x10cm field, and link it to DNA damage complexity and repair pathways. To assess variation in yD through the Bragg Peak and Spread-Out Bragg Peak we used a solid-state microdosimeter (MicroPlus probe). The measurements in the plateau region showed an average yD across the field of 3.59 keV/μm and increased from 4.74 keV/μm in the pristine peak to 7.46 keV/μm in the distal edge reaching a value of 8.62 keV/μm further down the distal edge (Figure1). Other than that we observe a rapid increase in yD towards the distal edge of the Bragg peak (Figure2), the results show less variation in yD across the field at larger depths. This points out that a highly uniform field in dose (98.0% at 40mm) does not indicate uniformity in yD (SD = 0.38 keV/μm). We even observe more uniformity in yD (SD = 0.07 keV/μm) in a less uniform field in dose (91.7% at 95mm), which emphasises the importance of studying yD variation across the field to assess DNA damage induction and repair pathways.

### P 038 - A prediction model for monitor units in proton therapy using a dedicated ocular beamline

#### Emmanuelle Fleury^1,2^, Joël Herault^3^, Kees Spruijt^2^, Sven Meijer^2^, Gaëlle Angellier^3^, Petter Hofverberg^3^, Jean-Philippe Pignol^4^, Mischa Hoogeman^1,2^, Petra Trnková^5^

##### *^1^Erasmus Medical Center, Department of Radiotherapy, Rotterdam, Netherlands*

*^2^HollandPTC, Radiation Oncology, Delft, Netherlands*

*^3^Centre Antoine Lacassagne, Department of Radiotherapy, Nice, France*

*^4^Dalhousie University, Department of Radiation Oncology, Halifax, Canada*

*^5^Medical University of Vienna, Department of Radiation Oncology, Vienna, Austria*

**Purpose/Objective:** In ocular proton therapy using a dedicated eye nozzle, the current commercial treatment planning systems do not provide the number of monitor units (MUs) to be delivered during treatment. Therefore, the number of MUs is measured for each individual treatment plan. This study aims to propose a prediction model for calculating plan-specific MUs so that plan-specific output measurements are no longer needed.

**Material and methods:** As inputs for the prediction model, output factors as a function of maximal range, spread-out Bragg peak, air-gap and field size characterizing the dedicated 62-MeV ocular beamline of Nice (Centre de Protonthérapie, France) were measured. A 3^rd^-order best-fit curve was used to model the MUs as a function of the abovementioned parameters. For validation, the required MUs for 599 ocular melanoma patients previously treated in Nice between 2019 and 2021 (485 choroidal melanomas, 74 conjunctival melanomas, 12 hemangiomas and 28 other ocular treatments) were calculated with Monte Carlo simulations using MCNPX code and were compared to the prediction model.

**Results**: Across all patients, relative errors between simulated and predicted MUs fall within 1.5% on average. Figure 1 displays the relative error distributions per group. For choroidal melanomas, we observed a relative mean error of -1.37% (SD=3.14%); for conjunctival melanomas, a mean of 1.16% (SD=4.82%); for hemangiomas, a mean of -0.95% (SD=2.21%), and for the remaining group a mean of -0.73% (SD=2.72%).

**Conclusion:** We developed a model to calculate the number of MUs of an ocular proton therapy treatment plan. A multicenter validation is currently underway.

### P 039 - The assessment of in-vivo neutron dose to the fetus in pregnant patient during brain tumor irradiation with proton scanned beam

#### Katarzyna Czerska^1^, Marta Bałamut^1^, Marijke De Saint-Hubert^2^, Hubert Jabłoński^1^, Wiktor Komenda^1^, Dawid Krzempek^1^, Natalia Mojżeszek^1^, Paweł Rogalski^1^, Marzena Rydygier^1^, Renata Kopeć^1^

##### *^1^Institute of Nuclear Physics Polish Academy of Sciences, Cyclotron Centre Bronowice, PL-31342 Krakow, Poland*

*^2^Belgium Nuclear Research Center, Research in Dosimetric Applications, Mol BE-2400, Belgium*

Pregnancy is the main contraindication to radiotherapy. However, in specific cases further delay in treatment may not be clinically acceptable and minimization of an embryo exposure is then of the highest importance. This study presents the assessment of neutron dose equivalent to the fetus and evaluates the use of bubble detectors for in-vivo dosimetry in pregnant patients. The Alderson Rando anthropomorphic phantom and RW3 slabs employed a pregnant patient geometry (Fig.1). To irradiate the brain target to the prescribed dose of 54Gy(RBE), the lateral beam with range shifter and proton PBS technique were used. The out-of-field fetus neutron doses were measured using bubble detectors (BD-PND) and compared with WENDI-2 detector results. The maximum neutron dose equivalent measured within the fetus and in the fetus center achieved accordingly, 1.3 and 1.1μSv/Gy and comparison to WENDI-2 showed ∼60% dose underestimation. The doses measured at the chest and belly button reached 13.3 and 1.6 μSv/Gy, respectively. As expected, the neutron doses measured in several locations within the fetus decreased with distance, reaching 1.2, 1.1, 0.6μSv/Gy (-6.5cm, fetus, +12cm) and 0.9μSv/Gy in depth (-6.5cm). The results show that chest single fraction dose corresponded to the dynamic range of the bubble detectors, which indicates their possible use for in-vivo dose measurements in pregnant patients. The discrepancies between bubble and WENDI-2 detectors might result from their uncertainty and different sizes, implying certain measurement geometry. Therefore, further studies are necessary to expand and confirm these assumptions.

### P 040 - Effects of data selection and model assumptions for in vitro-based modelling of proton relative biological effectiveness

#### Erlend Lyngholm^1^, Kristian Smeland Ytre-Hauge^1^

##### ^1^University of Bergen UiB, Department of Physics and Technology, Bergen, Norway

**Background and motivation**: Several models have been developed to account for the potential effects of a variable proton relative biological effectiveness (RBE). There are significant variations between RBE estimates from different models. Therefore, none of them have been applied in clinics, and a constant RBE=1.1 is still used in clinics today. Many models are based on data from monoenergetic (M) *in vitro* cell irradiation, while some also include data obtained with spread-out Bragg peak (SOBP) beams. This study aimed at analyzing all available proton *in vitro* RBE data to identify potential differences between M and SOBP data, and investigate how database restrictions and model assumptions affects the modelling.

**Methods**: A database was collected from the literature. Each data point included linear-quadratic model parameters and beam quality (LET) information. Data was fitted using linear regression so that the resulting fit parameter, k, indicates the slope of the RBE-LET relation. Fits were performed with different database restrictions on the range of included LET and reference (α/β)_x_ values.

**Results**: The database contained 148 data points from M- and 265 from SOBP irradiations. A trend of overall higher k values for SOBP data compared to M data was observed, both with and without database restrictions. Fitting results for databases with restrictions on (α/β)_x_ indicated a non-linear RBE-(α/β)_x_ relationship.

**Conclusion**: Our results indicate that M and SOBP *in vitro* data predicts different RBE values and that RBE models and their estimates are strongly affected by database restrictions and model assumptions. This could partly explain discrepancies observed between different published RBE models.

### P 041 - Design of a preclinical proton minibeam radiotherapy facility: dose monitor and range shifter

#### Jessica Neubauer^1^, Judith Reindl^1^, Tarik Gencaslan^1^, Gerd Datzmann^1^, Michael Mayerhofer^1^, Günther Dollinger^1^

##### ^1^Universtät der Bundeswehr München, applied physics and measurement technology, Neubiberg, Germany

Spatial fractionated radiotherapy using protons, so-called proton minibeam radiotherapy (pMBRT) was developed for better sparing of normal tissue in the entrance channel of radiation. Preclinical in-vivo experiments conducted with pMBRT in mouse ear models or in rat brains support the prospects. However, the research on radiobiological mechanisms and the search for adequate application parameters delivering the most beneficial minibeam therapy is still in its infancy. Progressing towards clinical usage, pMBRT research should overcome technical and biomedical limitations of the current irradiation test stages and animal models. This work discusses the design of a preclinical pMBRT facility located at an existing 68.5 MeV cyclotron. Two major parts, which have to be designed and constructed are the dose monitor and the range shifter. To be able to monitor high local dose rates a parallel plate ionization chamber was constructed. As electrodes 6 μm thick mylar foils coated with aluminum at a distance of 5 mm were used. Here, we present the final prototype construction and first reference measurements using an x-ray beam. The range shifter needs to be designed such that a beam diameter of ∼200μm (s) is not exceeded. We show beam spreading simulations of different materials, which can be used as range shifter, such as PMMA, carbon and aluminum. Also we present the possibility of using a hybrid range shifter splitted in two parts, one in vacuum and one in air. This on the one hand supports keeping beam size small and on the other hand offers maximum flexibility.

### P 042 - Comparison of radiation dose to the immune system between proton and photon radiotherapy for treatment of stage III NSCLC

#### Jimmy Patel^1^, Neal S. McCall^1^, Matthew Thomas^1^, Jun Zhou^1^, Kristin A. Higgins^1^, Jeffrey D. Bradley^1^, Sibo Tian^1^, Mark W. McDonald^1^, Aparna H. Kesarwala^1^, William A. Stokes^1^

##### ^1^Emory Winship Cancer Institute, Department of Radiation Oncology, Atlanta, USA

**Purpose**: Emerging data have illuminated the impact of effective radiation dose to immune cells (EDIC) on outcomes in patients with stage III non-small cell lung cancer (NSCLC) undergoing intensity-modulated radiotherapy (IMRT) and chemotherapy. Hypothesizing that intensity-modulated proton therapy (IMPT) may reduce EDIC versus IMRT, we conducted an institutional dosimetric comparison of EDIC in patients with unresectable Stage III NSCLC.

**Methods**: Data were retrospectively collected for 12 patients treated between 2019 and 2021 who had physician-approved IMRT and IMPT plans generated for conventionally fractionated (chemo)radiotherapy. Data to calculate EDIC from both Jin (PMID: 34944813) and Ladbury (PMID: 31175902) models were abstracted, including mean lung dose, mean heart dose, mean body dose, body volume, and integral total dose. Mean EDIC was compared between the 12 IMPT plans (in GyE) and the 12 IMRT plans (in Gy) with two-sided paired t-tests.

**Results**: Our cohort had a mean age of 69 years, and most had N2 or greater nodal disease (66.7%). All but one received chemotherapy. Mean gross tumor volume was 140cc. One patient was prescribed 70Gy(E), while the rest were prescribed 60 Gy(E). EDIC was lower with IMPT than IMRT for 11 of 12 patients (91.7%). Mean EDIC was significantly lower with IMPT than IMRT by both models (Table).

**Conclusion**: IMPT offers a statistically significant reduction in EDIC compared to IMRT. With EDIC emerging as a modifiable prognostic factor, our dosimetric study highlights a novel potential role for IMPT to improve oncologic outcomes in patients with Stage III NSCLC.

### P 043 - Commissioning of Grid-Lattice proton PBS system using only Gafchromic EBT3 film as detector

#### Robabeh Rahimi Darabad^1^, Jiajin Fan^1^, Peng Wang^1^

##### ^1^Inova Schar Cancer Institute, Radiation Oncology, Fairfax, USA

Commissioning of spatially fractionated proton therapy (Grid/Lattice therapy) is challenging. In order to achieve a clinically acceptable peak-to-valley ratio, the treatment plan includes spatially separated small-dimension fields, which requires detectors with high spatial resolution and wide detection range, for measuring peaks and valleys, respectively. Film has fine resolution (0.002 cm) and can be calibrated for wide range absolute dosimetry. But the non-linear response of film due to its linear energy transfer dependency deteriorates the measurement result accuracy. Ion chambers were used for absolute dose measurements, and film measurement results were scaled up accordingly, in an attempt to account for film under-response due to LET effect. However, clinically available ionization chambers suffer from volume averaging effect for small fields involved in Grid/Lattice therapy. A method is developed in our center to mitigate the film response for combinations of range, modulation and depth of treatment fields. The treatment planning system (TPS) accuracy for Grid/Lattice therapy has been verified utilizing the technique which relies only on Gafchromic EBT3 film for measurements. Figure 1 shows gamma analysis result of a field with only central-axis (CAX) proton spots. Figure 2 shows gamma analysis result of a patient' plan.

### P 044 - Impact of magnetic fields on the ionization chamber response in carbon ion dosimetry

#### Sonja Surla^1,2^, Mathieu Marot^1,3^, Lucas Burigo^1^, Stephan Brons^4,5^, Christian Karger^1,5^

##### *^1^German Cancer Research Center DKFZ, Department Medical Physics in Radiation Oncology, Heidelberg, Germany*

*^2^University of Heidelberg, Faculty of Physics and Astronomy, Heidelberg, Germany*

*^3^University of Heidelberg, Faculty of Medicine, Heidelberg, Germany*

*^4^University Hospital Heidelberg, Heidelberg Ion-Beam Therapy Center HIT, Heidelberg, Germany*

*^5^National Center for Radiation Research in Oncology NCRO, Heidelberg Institute for Radiation Oncology HIRO, Heidelberg, Germany*

Magnetic resonance guided ion beam therapy (MRgIT) may increase treatment effectiveness by combining the high soft tissue-contrast in MR imaging with conformal dose delivery. However, static magnetic field alters the dose distribution as well as detector response. To ensure high precision of MRgIT dose delivery, the dosimetric impact of magnetic fields has to be further investigated before MRgIT can be clinically implemented. This work aims to investigate the chamber response in carbon ion beams as a function of the magnetic field strengths and the chambers' volume. Measurements were performed in a water phantom, placed between the pole shoes of an experimental electromagnet AGEM 5520 Schwarzbeck Mess-Elektronik. Chambers were positioned at the plateau region of a monoenergetic 390.75 MeV/u carbon ion beam. The responses of the Farmer-type ionization chamber PTW 30013 (radius 3 mm) and five custom-built chambers with the same design, but different radii (from 1 to 6 mm), were measured for magnetic field densities from 0 to 1 T. Preliminary results show small but significant alterations of the chamber response with a non-linear dependence on the magnetic field. The largest changes of 0.4 % were observed between 0.2 and 0.3 T for the chamber with 3 mm radius. For the smallest chamber, the alteration was largest (0.6 % at 0.8 T), and for the largest chamber 0.5 % between 0.1 and 0.2 T was observed. Magnetic field and chamber radius impact chamber response by up to 0.6% and these corrections should be considered by magnetic field correction factors.

### P 045 - Use of a new high resolution detector for proton beam spot profile measurement during acceptance testing and commissioning

#### Yajun Jia^1^, Zhangmin Li^1^, Yifeng Yang^1^, Xiaogang Yuan^1^, Bing Liu^1^, Zhiyuan Peng^2^, Jianfeng Li^2^, Yuanshui Zheng^1^, Zuofeng Li^1^

##### *^1^Guangzhou Concord Cancer Center, Radiation Oncology, Guangzhou, China*

*^2^Varian Medical Systems, Proton Therapy, Guanghzou, China*

**Purpose**: The purpose of this abstract is to present our experience on using a new high resolution detector Phoenix (IBA) to measure proton beam spot fluence profile of treatment room 4 at Guangzhou Concord Cancer Center (GCCC) for acceptance testing and clinical commissioning.

**Methods**: The Phoenix has a large active area of 41x41 cm2 and 2048X2048 sensors with a 0.2 mm high resolution. The lateral distribution of beam spot in air was measured using Phoenix in isocenter plane, ±15 cm and ±30 cm from isocenter plane along beam axis at gantry 0°, and repeated at isocenter plane only at gantry 90° and 270°. Beam spot was measured for every 5 MeV between 70 MeV to 244 MeV. Measurement with Phoenix was compared to the vendor provided IAS (Varian), which has 40x30 cm^2^ in sensitive area and 0.392 mm resolution, and EBT3 film (0.005mm resolution).

**Results**: For each energy and position, the spot sigma values in the inline plane (Y) and crossline Plane (X) were within 5%. The difference for sigma values on isocenter plane among Gantry 0°, 90° and 270° is less than 6%, with difference between 90 and 270 the largest. The Phoenix measurement agreed with IAS results well (< 3%). Preliminary measurement with film shows a slightly larger spot sigma than Phoenix measurement and further study is in progress.

**Conclusions**: Phoenix is an efficient and accurate tool for spot fluence profile measurement during acceptance and commissioning.

### P 046 - Use of Monte Carlo as independent verification method for dose distribution calculation

#### Klara Badraoui Cuprova^1^, Vladimir Vondracek^1^, Jan Vilimovsky^1^, Michal Andrlik^1^, Simona Kolaciova^1^, Lubomir Zamecnik^1^, Matej Navratil^1^

##### ^1^Proton Therapy Center Czech, Clinical Physics Department, Prague, Czech Republic

In Proton Therapy Center in Prague we have developed Monte Carlo based method for quality assurance of treatment plans. Dose calculation algorithms of treatment planning systems XiO and RayStation can be verified by means of Monte Carlo independent calculation. It provides complementary information to QA measurement in water as dose distribution is calculated in real inhomogeneous patient environment. Linear energy transfer distribution in patient can be simulated alongside. The more precise the output, the more computationally demanding the simulation is. In PTC, we have built a computer cluster composed of 154 cores and we use open-source software GATE (www.opengatecollaboration.org), which is a Monte-Carlo simulation toolkit for medical physics applications. For each treatment room of PTC facility, specific input files for GATE were created based on the measured data. The files describe radiation source – energy, beam optics and MU to dose calibration. Patient specific input files are created from RT Plan, RT Structure Set and CT scans. These files (in DICOM RT format) are exported from treatment planning system but they are not directly readable by GATE. We have created set of specific scripts in Python to process input and output data. Output dose and LET distributions are converted to DICOM RT format for successive analysis. Gamma analysis is performed in order to compare dose distributions exported from TPS with the ones from GATE simulation. By now, we deal with full automatization of the process.

### P 047 - Pushing the low-energy performance of MLFCs for beam energy validation

#### John Gordon^1^, Paul Boisseau^1^, Andrew Dart^1^, Max Chiou^2^

##### *^1^Pyramid Technical Consultants Inc., Engineering, Waltham, USA*

*^2^Heron Neutron Medical Corp, Medical Device Technology Development, Zhubei, Taiwan*

The Multi-Layer Faraday Collector was developed for proton therapy use at the Harvard cyclotron Laboratory by Gottschalk. We have developed commercial versions that have proved convenient and precise for routine machine QA in proton therapy and other applications. There has been increasing interest in using the device to measure relatively low proton energies, of 30 MeV and below. A particular recent requirement is validation of the proton energy for a cyclotron used for neutron generation for BNCT. For this application a resolution of 0.3 MeV at 30 MeV is needed. Low energy protons have a very sharp stopping peak in the MLFC structure, which results in insufficient data points for robust peak fitting, and therefore degraded energy resolving power below some energy. The work reported describes two parallel developments we have made to overcome this limitation. The first is the use of MLFC layers with lower stopping power than the standard copper. The second, very effective, measure is the addition of a carefully tailored spreading filter at the beam entrance that restores the ability to peak fit and interpolate down to much lower energies, without compromising the basic accuracy of the collector. We present simulations and initial data to show the how these measures extend the usefulness of the MLFC to a wider energy range.

### P 048 - Gamma-ray scintillator-based detection module for the development of a SPECT-BNCT system

#### Anita Caracciolo^1,2^, Davide Di Vita^1,2^, Luca Buonanno^1,2^, Marco Carminati^1,2^, Nicoletta Protti^3,4^, Saverio Altieri^3,4^, Carlo Fiorini^1,2^

##### *^1^Politecnico di Milano, Department of Electronics- Information and Bioengineering, Milan, Italy*

*^2^Istituto di Fisica Nucleare INFN, Sezione di Milano, Milan, Italy*

*^3^Università di Pavia, Department of Physics, Pavia, Italy*

*^4^Istituto di Fisica Nucleare INFN, Sezione di Pavia, Pavia, Italy*

We propose a gamma-ray scintillator-based detection module for the development of a SPECT system for real-time dose monitoring in Boron Neutron Capture Therapy (BNCT). BNCT is a radiotherapy technique where the tumor volume is loaded with boron-10 and irradiated with thermal neutrons. Boron neutron capture reactions occur, and they deposit their energy within the tumor cells, thus sparing normal cells. The ^10^B(n, α)^7^Li reactions create gamma rays at 478 keV, and their detection can be used to quantify and localize the dose delivered to the patient. However, this detection is very challenging because of the mixed radiation field present during BNCT irradiations and the low ^10^B concentration. We report about the performance of the BeNEdiCTE (Boron Neutron CapTurE) module, based on a 2 inches cylindrical LaBr_3_(Ce+Sr) scintillator crystal, optically coupled to a matrix of Silicon Photomultipliers (SiPMs), when irradiating ^10^B-loaded samples with neutrons. Vials filled with different boron concentrations have been irradiated in the TRIGA MARK II nuclear reactor of Pavia University (Italy), and spectra have been acquired with the BeNEdiCTE module, wrapped in cadmium foils to avoid the neutron activation of the detector. The excellent energy resolution of the module (<3% at 662 keV) allows to resolve the photopeak at 478 keV (Picture 1). Great linear correlation between the number of events detected at 478 keV and the boron concentration in the irradiated samples has been achieved (Picture 2).

### P 049 - Enhancing gamma production for real-time dose verification in proton therapy

#### Giorgio Cartechini^1,2^, Elena Fogazzi^1,2^, Luna Pellegri^3,4^, Michela Marafini^5^, Franco Camera^6^, Francesco Tommasino^1,2^, Emanuele Scifoni^2^, Chiara La Tessa^1,2^

##### *^1^University of Trento, Physics, Trento, Italy*

*^2^Trento Institute for Fundamental Physics and Application TIFPA-INFN- Trento- Italy, Physics, Trento, Italy*

*^3^University of the Witwatersrand, Physics, Cape Town, South Africa*

*^4^iThemba LABS, Physics, Cape Town, South Africa*

*^5^Museo Storico della Fisica e Centro Studi e Ricerche E.Fermi, Physics, Roma, Italy*

*^6^La Statale- Università di Milano, Physics, Milano, Italy*

One of the main limits for the full exploitation of proton therapy is the particle range uncertainty due to patient setup, dose calculation and imaging. To account for this, a safety margin is added to the tumor, ensuring that the prescribed dose is uniformly delivered to the target. An accurate monitoring of the beam range during the treatment would allow to reduce the irradiation volume, both ensuring a correct coverage of the tumor and decreasing the irradiation of the normal tissue. We developed a novel strategy for real time range verification in proton therapy. The methodology is based on the detection of prompt gammas (PG), whose production is artificially enhanced with a non-radioactive element transported selectively to the tumor with a drug carrier. Nuclear interactions of this element with protons generate a signature PG spectrum, from which the tumor position can be reconstructed exploiting existing PG Spectroscopy (PGS) methods. In this study, we present the results obtained with three stable elements: ^31^P, ^63^Cu and ^89^Y. We characterized the gammas emitted by solutions of water and the candidate elements (CuSO_4_+H2O, NaH_2_PO_4_+H_2_O and Y(NO_3_)_3_+H_2_O) when exposed to therapeutic proton beam energies. We investigated the minimum element concentration in water required to detect a PG enhancement compared to a pure water solution. Experimental measurements indicated that ^31^P and ^89^Y are the most promising elements, as they produce signature PGs in the 0.8 MeV -1.4 MeV (figure 1) range, with an enhancement of about 4% (^31^P) and 1.4% (^89^Y) at a concentration of 0.5% (figure 2).

### P 050 - Clinical Implementation of a flat-panel based compact daily quality assurance device (Sphinx Compact) for proton pencil beam scanning (PBS) applications

#### Daniel D. Cho^1,2^, Mauricio A. Acosta^1^, Victor Chirinos^1^, Eduardo Pons^1^, Alonso Gutierrez^1,2^, Andrew J. Wroe^1,2^

##### *^1^Miami Cancer Institute, Radiation Oncology, Miami, USA*

*^2^Florida International University, Department of Radiation Oncology- Herbert Wertheim College of Medicine, Miami, USA*

**Purpose:** This work presents the initial benchmark testing of Sphinx Compact (SC) for PBS daily QA.

**Methods:** SC consists of a flat-panel detector, integrated solid water phantom and a PPC05 ion chamber insert. This allows for the completion of daily QA beam checks recommended by TG-224. The SC is indexed to the table using an alignment bar and aligned to the beam central axis using the laser and X-ray alignment system. The provided PBS Layer Definition (PLD) file defines the QA irradiation pattern. It delivers a proton beam pattern to evaluate spots (5 energies), range (3 energies), homogeneity, coincidence and output. MyQA with the sphinx-plugin was used for data acquisition and analysis. Multiple measurements were completed to evaluate stability of the SC. For comparison, data was also acquired using the Lynx with Sphinx attached (SL) as this is our standard daily QA device

**Results:** The table 1 shows the initial results of Daily QA measurements. The maximum variation of spot sigma reached to the tolerances recommended by TG-224 but average fluctuations are within tolerances. Initial comparisons between SL and SC show good agreement with the exception of lower energy spot sigma being larger for SL (Table 2). This could be the result of the difference in resolution or additional effects with flat-panel detector. Additional data is being collected to verify these trends.

**Conclusion:** Following initial evaluation the SC is a stable and accurate tool with which to complete the daily beam QA in PBS as recommended by TG-244.

### P 052 - Implementation single set-up monthly QA at different gantry angles using an array detector and a rotating unit: one year results

#### Esther Decabooter^1^, Gloria Vilches-Freixas^1^, Erik Roijen^1^, Jonathan Martens^1^, Esther Kneepkens^1^, Mirko Unipan^1^, Wiel Habets^1^, Marcel Walpot^1^, Bas Nijsten^1^, Geert Bosmans^1^

##### ^1^Maastro clinic, Physics Innovation, Maastricht, Netherlands

**Purpose**: Evaluation of the clinical implementation of a single set-up monthly QA at different gantry angles over 12 months.

**Materials and methods**: We developed a monthly QA procedure using an array detector (PTW Octavius XDR 1500) embedded in a rotational unit (PTW Octavius 4D) at our pencil beam scanning proton facility. With a single set-up we can monitor output, range, field central axis position, field symmetry and flatness, penumbra widths, spot sizes and positions at different gantry angles (AAMPM TG 224). The set-up is verified by kV-imaging and further irradiated with homogenous 18x18 cm^2^ fields with dynamic aperture and spot patterns with different energies at five gantry angles. A modular 32 cm diameter top is used to check the range consistency. Absolute 3D γ-analysis is performed to compare output and spot shape to TPS dose distributions. All other parameters are directly extracted from the 2D field measurements.

**Results**: Figure 1 shows a trend analysis of the different beam parameters throughout the year for different gantry angles. All parameters are within the ±1mm, ±1% or ±2% tolerance values. Figure 2 summarises the difference in expected spot position relative to the central spot for different spot patterns at five different gantry angles. All deviations are within ±1mm. Range differences are all within ±0.6 MeV relative to baseline. Most (2%,2mm) γ-analysis showed agreement scores higher than 90%. Lowers scores lead to beam adjustments.

**Conclusion**: The time-efficient monthly QA procedure that we developed can accurately be used to measure a large range of beam parameters at different gantry angles.

### P 053 - Substituting experimental patient specific QA (PSQA) by independent dose calculation (IDC) for scanned proton and carbon ion beams

#### Loïc Grevillot^1^, Dreindl Ralf^1^, Carlino Antonio^1^, Stock Markus^1^, Alessio Elia^1^

##### ^1^EBG MedAustron GmbH, Medical Physics, Wiener Neustadt, Austria

Historically, IDC was recommended and implemented as PSQA tool for conformal radiation therapy. Due to the increased complexity of treatments, experimental PSQA has been recommended for IMRT/VMAT treatments, in part due to the limited accuracy of secondary dose computation algorithms and the lack of commercial solutions. In the last decade this status significantly changed and IDC is currently used routinely for IMRT/VMAT. IDC solutions for scanned proton or carbon ion beams are still scarce. Implementing Monte Carlo (MC)-based algorithms in IDC tools can provide calculation accuracy equivalent or superior to currently available TPS. In addition, there are evidences that IDC-based PSQA is significantly more sensitive than experimental-based PSQA in catching errors (AAPM TG219). We implemented clinically the first commercial MC-based IDC system for scanned proton beams in February 2021. Experimental PSQA was substituted step-wise by IDC, considering the treatment delivery complexity and dose per fraction. The machine-specific beam delivery QA program was also revised to increase the overall safety. IDC allows to better review complex treatment plans (e.g., implants, range shifter) and increase the level of QA when implemented in combination with a comprehensive machine QA program. In December 2021, experimental PSQA was reduced by 57% overall (considering proton and carbon ion treatments) and by 88% considering proton treatments only (*Figure 1*). The first MC-based commercial IDC system for scanned carbon ion beams is currently under commissioning and will be deployed similarly in 2022/2023 to further reduce the experimental carbon ion PSQA in our facility.

### P 054 - Non – radioactive elements for prompt gamma enhancement in proton therapy

#### Panagiota Galanakou^1^, Theodora Leventouri^1^, Wazir Muhammad^1^

##### ^1^Florida Atlantic University, Physics, Boca Raton, USA

Regardless of the developed advanced Prompt Gamma (PG) techniques to monitor the proton range *in-vivo*, the poor PG statistics downgrade the potential of their clinical implementation. We propose the injection of the non-radioactive elements ^19^F, ^17^O, ^127^I in the tumor area to enhance the PG production of the 4.44 MeV and 6.13 MeV PG rays that have been correlated with the distal fall-off of the proton Bragg Peak. We have simulated the interaction of a 75 MeV, 100 MeV and 200 MeV incident proton beam in a co-centric cylindrical geometry phantom using the TOol for PArticle Simulation (TOPAS) Monte Carlo (MC) package; the outer cylinder was filled with 100% water and the inner cylinder (hypothetical tumor area) that is leveled with respect to the outer cylinder was filled with water containing 0.1% - 20% weight fractions of each of the tested elements. We demonstrate that the proposed technique improves the PG statistics under certain combinations of the incident proton beam energies and mixture composition. Our MC results indicate that enhancement of the PG rays for monitoring the proton range during proton therapy appears promising. This is the first time that such enhancement technique is proposed; this study is a starting point for further work that is in progress to quantify the necessary, safe amount of the non-radioactive element to be injected.

### P 055 - Development of patient-specific quality assurance system based on Monte Carlo algorithm for carbon-ion radiation therapy

#### Yongdo Yun^1^, Hyo Kyeong Kang^1^, Min Cheol Han^1^, Hikaru Souda^2^, Takayuki Kanai^2^, Sung Hyun Lee^2^, Yuya Miyasaka^2^, Takeo Iwai^2^, Jin Sung Kim^1^

##### *^1^Yonsei University College of Medicine, Department of Radiation Oncology, Seoul, Korea Republic of*

*^2^Yamagata University Graduate School of Medical Science, Department of Heavy Particle Medical Science, Yamagata, Japan*

The objective of this study is to develop a patient-specific quality assurance system (PSQAS) based on machine-measured log files for carbon-ion radiation therapy (CIRT). The system could support a 3D plan re-calculation using TOPAS Monte Carlo (MC) code and provide pre-treatment quality assurance results via MC with log files. The PSQAS is divided into three parts: (1) automatic beam commissioning system based on measurement data, (2) implementation system of DICOM dataset (i.e., CT, RT-Structure, RT-Plan, and RT-Dose) and machine log file, and (3) dose calculation system including a CIRT-dedicated biological dose calculation model. To validate the developed system, automatic beam commissioning was performed by measuring data. Subsequently, implementation tests were performed using a DICOM dataset and a log-file. Finally, a 3D dose distribution in water was calculated by PSQAS and compared with that calculated by treatment planning system (TPS). It was confirmed that the PSQAS was successfully developed. The MC-dedicated beam model was generated within 1% uncertainty for measured beam range. A patient CT and the log file were successfully implemented (Figure 1). The result of dose comparison showed a high accuracy in water (γ-passing rates of >99%). At present, ethical approval is in progress for retrospective study based on patient CTs, and we hope the detailed results will be presented at PTCOG60.

### P 056 - In vitro and in vivo evaluation of Zn nanoparticles for proton range verification in a glioblastoma cell line

#### Marta Ibañez^1^, Irene Fernández-Barahona^2,3^, Rocío Santacruz^1^, Marta Oteo^1^, Victor M. Luján^1^, Natalia Magro^1^, Samuel España^4^, Luis M. Fraile^4^, Fernando Herranz^3^, Miguel Ángel Morcillo^1^

##### *^1^Centro de Investigaciones Energéticas- Medioambientales y Tecnológicas CIEMAT, Medical Applications of Ionizing Radiation, Madrid, Spain*

*^2^Universidad Complutense de Madrid, Facultad de Farmacia, Madrid, Spain*

*^3^Instituto de Química Médica - Consejo Superior de Investigaciones Científicas IQM-CSIC, Nanomedicine and molecular imaging Group, Madrid, Spain*

*^4^Universidad Complutense de Madrid, Nuclear Physics Group, Madrid, Spain*

Proton therapy allows specific dose deposition in tumors, however proton deposition range has uncertainties and a verification method is missed. *In vivo* range verification can be based on proton-activable agents that generate products quantifiable by prompt-gamma detection. Here, we synthesized a citrate-coated iron oxide nanoparticles doped with Zn (IONP@Zn-cit) for this purpose, studying how they affect cells with and without irradiation using *in vitro* studies and evaluating tumor uptake in a preclinical glioblastoma model. First, we assessed their cytotoxicity using MTT assay in U251 cell line (Figure A) and EC50 value of 99.79 μgZn/mL after 24 hours-incubation was obtained. Secondly, clonogenic assays with IONP@Zn-cit were used to evaluate their contribution to cell death within X-ray irradiation. No significant changes were observed in U251 survival fractions due to nanoparticles (Figure B). Moreover, ^67^Ga radiolabeled IONP@Zn-cit (^67^Ga-IONP@Zn-cit) was used to assess accumulation both *in vitro* and *ex vivo*. After 24 hours, a concentration-dependent amount of Zn/cell (Figure C) was accumulated in U251 cells. Regarding their biodistribution, subcutaneous U251 mice model showed accumulation at 1-day post inoculation in liver (32.75%ID/g) and spleen (32.02%ID/g). Tumor uptake value was 0.95%ID/g (Figure D). Finally, IONP@Zn-cit solutions were irradiated with 10 MeV proton beam at the Center for Microanalysis of Materials (Madrid) and prompt signal is observed at the lowest concentration (Figure E). In conclusion, IONP@Zn-cit could be used as a tool for validation of dose deposition with protons using prompt-gamma imaging and our next aim would be improve nanoparticle accumulation in tumor by active targeting.

### P 057 - LETd measurements of double-scattered proton fields with patient-specific range-compensator and aperture using silicon on insulator microdosimeter

#### Linh Tran^1^, Wen Hsi^2^, Romaine Nichols^2^, David Bolst^1^, Marco Petasecca^1^, Susanna Guatelli^1^, Michael Lerch^1^, Erik Traneus^3^, Michael Jackson^4^, Anatoly Rozenfeld^1^

##### *^1^University of Wollongong, Centre for Medical Radiation Physics, Wollongong, Australia*

*^2^University of Florida Health Proton Therapy Institute, Department of Radiation Oncology, Florida, USA*

*^3^RaySearch Laboratories, RaySearch Laboratories, Stockholm, Sweden*

*^4^University of New South Wales, Prince of Wales Clinical School, Sydney, Australia*

Double-scattered proton fields with patient-specific range-compensators and apertures were used to treat various types of cancers at the UF Health Proton Therapy Institute. For pancreatic adenocarcinoma, two posterior-oblique fields delivered 63GyEq in 28 fractions [1]. In this study we focused on the effect of variable Relative Biological Effectiveness (RBE) on dose within organs at risk (OAR). RBE of proton beams, depends on proton and secondary dose mean linear energy transfer (LET_d_), which varies throughout the treatment volume. One of the models incorporated in the RaySearch treatment planning system (TPS) calculates D(GyEq)=RBE×D=(1+κLET_d_) × D, where κ is a cell line related parameter (κ=0.04) [2]. By taking into account variable RBE, the biological dose is actually higher and misplaced relative to the originally planned volume potentially resulting in damage to OARs. MicroPlus microdosimetry system [3] developed by the Centre for Medical Radiation Physics, University of Wollongong was used to measure the microdosimetric stochastic quantity dose mean lineal energy value (close to LET_d_) [4], in a water phantom along the central and 4 cm laterally axes. Figures 1b and 2 show the distribution and its translation to dose (GyEq) as a function of depth in water along the central axis of the beam for the first field (2A2), respectively. Variable LET_d_ leads to dose (GyEq) increasing up to 16% and misplacement about 2mm towards OAR compared to conventional planning with RBE=1.1. Detailed dose and LET_d_ measurements for two plans and their comparison with those calculated by TOPAS Monte Carlo and RaySearch TPS will be presented.

### P 058 - Sensitivity of treatment monitoring using charged nuclear fragments to patient positioning in carbon-ion radiotherapy

#### Laurent Kelleter^1,2,3^, Pamela Ochoa-Parra^1,2,4^, Meera Subramanian^1,5^, Marcus Winter^2,6^, Jürgen Debus^2,3,6,7^, Oliver Jäkel^1,2,3,6^, Maria Martišíková^1,2,3^

##### *^1^German Cancer Research Centre DKFZ, Department of Medical Physics in Radiation Oncology, Heidelberg, Germany*

*^2^National Center for Research in Radiation Oncology, Heidelberg Institute for Radiation Oncology HIRO, Heidelberg, Germany*

*^3^National Center for Tumor Diseases, NCT Heidelberg, Heidelberg, Germany*

*^4^Heidelberg University, Department of Physics and Astronomy, Heidelberg, Germany*

*^5^Otto-von-Guericke-University Magdeburg, Faculty of Electro Engineering and Information Technology, Magdeburg, Germany*

*^6^Heidelberg University Hospital, Heidelberg Ion Beam Therapy Center, Heidelberg, Germany*

*^7^Heidelberg University Hospital, Department of Radiation Oncology, Heidelberg, Germany*

The high dose gradient makes carbon-ion radiotherapy (CIRT) susceptible to anatomical changes or patient positioning errors. Charged nuclear fragments, emitted in nuclear interactions of carbon ions with patient tissue, have been proposed for in-vivo, real-time and non-invasive treatment monitoring. The in-vivo monitoring (InViMo) project aims at bringing carbon-ion beam monitoring to clinical application. A mini-tracker (4 cm^2 sensitive area) based on hybrid silicon pixel detectors TPX3 was used to measure tracks of emerging charged nuclear fragments. Our group previously demonstrated the possibility of detecting and localising 2 mm thick air slabs in a homogeneous plastic head phantom. In this contribution, it was investigated whether fragment tracking is sensitive to patient translations and rotations, which could interfere with the detection of anatomical changes. The experiments were performed at the Heidelberg Ion Beam Therapy Center (HIT) in Germany. A realistic spherical target volume (50 cm^3) located at the base-of-skull of an anthropomorphic Alderson head phantom was irradiated with an RBE-weighted dose of 1.5 Gy(RBE). It was found that translations of the phantom along the beam axis as small as 1 mm could be detected and quantified. Horizontal translations of up to 3 mm as well as rotations of up to 3 degrees did not generate a significant change of the fragment distribution. It is concluded that fragment tracking is insensitive to small patient translations transverse to the beam axis as well as rotations around the vertical room axis, which will therefore not interfere with the detection of anatomical changes.

### P 059 - Helium-beam radiograph of an anthropomorphic head phantom using thin silicon pixel detectors and the assessment of its accuracy

#### Margareta Metzner^1,2^, Mohsin Raza^1,2^, Florian Kehrein^1,2^, Catherine Knobloch^1,2^, Benjamin Ackermann^3^, Stephan Brons^3^, Oliver Jäkel^1,2,3^, Maria Martisikova^1,2^, Tim Gehrke^1,2,4^, Laurent Kelleter^5,6,7^

##### *^1^German Cancer Research Center DKFZ, Medical Physics in Radiation Oncology, Heidelberg, Germany*

*^2^National Center for Radiation Research in Oncology NCRO, Heidelberg Institute for Radiation Oncology HIRO, Heidelberg, Germany*

*^3^Heidelberg Ion-Beam Therapy Center HIT, Radiation Oncology- Heidelberg University Hospital, Heidelberg, Germany*

*^4^Heidelberg University Hospital UKHD, Radiation Oncology, Heidelberg, Germany*

*^5^German Cancer Research Centre DKFZ, Department of Medical Physics in Radiation Oncology, Heidelberg, Germany*

*^6^National Center for Tumor Diseases, NCT Heidelberg, Heidelberg, Germany*

*^7^National Center for Research in Radiation Oncology, Heidelberg Institute for Radiation Oncology HIRO, Heidelberg, Germany*

To use the highly-conformal dose distributions of ion-beam therapy even more effectively, it is of great interest to reduce uncertainties that are often related to anatomical changes and inaccuracies during the determination of the relative stopping power (RSP) within the patient's anatomy. Ion-beam radiography (iRAD) has the potential of directly measuring the integrated RSP along beam direction, called water-equivalent thickness (WET). Therefore, low-dose iRAD could verify the agreement between the actual RSP distribution and the one used for treatment planning. Recently, a method for helium-beam radiography (αRAD) based on a detection system that exclusively consists of thin silicon pixel detectors was developed. At the Heidelberg Ion-Beam Therapy Center, where helium-ion beams are available, the detection system is used to image an anthropomorphic head phantom (figure1(a)).This contribution presents an investigation of a strategy to tackle the biggest challenge using thin detectors—the constrained WET-range, within which WETs can be precisely measured. The strategy, named “energy painting”, describes the modulation of the initial beam energy at multiple small subregions of the image based on estimated WETs. After establishment of calibration curves between measured energy deposition and WET for each beam energy, a WET map of a region in the head was obtained, shown in figure1(b). The spatial resolution compares well to the current gold standard based on dual-energy CT (DECT) (figure1(c)). Most importantly, the WET accuracy in the majority of pixels is already better than 1% compared to DECT (figure1(d)).

### P 060 - A preclinical dosimetry audit using an anthropomorphic murine phantom

#### Emma Biglin^1^, Adam Aitkenhead^2^, Gareth Price^1^, Amy Chadwick^1^, Elham Santina^1^, Kaye Williams^3^, Karen Kirkby^1^

##### *^1^University of Manchester, Division of Cancer Sciences- Faculty of Biology- Medicine and Health, Manchester, United Kingdom*

*^2^The Christie NHS Foundation Trust, Christie Medical Physics and Engineering, Manchester, United Kingdom*

*^3^University of Manchester, Division of Pharmacy and Optometry- Faculty of Biology- Medicine and Health, Manchester, United Kingdom*

One concern with preclinical radiation research is a lack of robust dosimetry protocols that provide traceability to a primary standard. Without this, accuracy and reproducibility between studies is compromised. Our group have utilised 3D printing to create a phantom with the heterogeneous density, size and physiological features of a real mouse. We propose the phantom can be used as part of a proton dose measurement audit, testing the techniques implemented to utilise the beam at the small animal scale and comparing the planned and delivered doses, across multiple international institutions. To demonstrate the suitability of the murine phantom to perform such measurements, a photon dosimetry audit of Small Animal Radiation Research Platforms (SARRPs) installed at 6 UK centres was undertaken. Alanine pellets and Gafchromic EBT3 film situated within the phantom permitted simultaneous measurement of the dose delivered and the 2D dose distribution, using static and arc plans, to assess the positional accuracy, absolute delivered dose and shape of the dose distribution. The absolute differences between the planned and alanine measured doses across all SARRPs was 3.6% ± 4.4% and -2.0 ± 5.9% (mean ± standard deviation) for the static and arc beams, respectively. The main differences in the EBT3 dose distribution measurements were seen around the field edges, slightly blurred in the film compared to a steep dose gradient in the treatment plan. This work demonstrates the use of the bespoke phantom to capture realistic dose measurements, providing the basis for a dosimetry audit with protons and heavier ions.

### P 061 - The impact of a patient-related Craniospinal Range Shifter on dosimetric verification of medulloblastoma treatment plan in proton radiotherapy

#### Marzena Rydygier^1^, Katarzyna Czerska^1^, Hubert Jabłoński^1^, Paweł Rogalski^1^, Marta Bałamut^1^, Magdalena Garbacz^1^, Urszula Sowa^1^, Kamil Kisielewicz^2^, Dawid Krzempek^1^, Renata Kopeć^1^

##### *^1^Institute of Nuclear Physics Polish Academy of Sciences- PL-31342 Krakow- Poland, Cyclotron Centre Bronowice, Krakow, Poland*

*^2^National Oncology Institute- National Research Institute- Krakow- Poland, Krakow Branch, Krakow, Poland*

To ensure sufficient dose coverage for shallowly located tumors in proton radiotherapy, an application of a range shifter is necessary. At the IFJ PAN a specially designed patient-related Craniospinal Range Shifter (*prCRS*) is applied in craniospinal treatments. It is tightly mounted under the table top during irradiation, in order to minimize the air-gap, thus the lateral beam spreading. In this study, two methods of dosimetric verification of the plans including *prCRS* are compared. The *prCRS,* a homogeneous block of PMMA (2.8x24x130cm^3^; WET=3.4cm), was manually added to the clinical medulloblastoma plan in TPS and overridden with 343HU value. The measurements included the dosimetric verification of the plan recalculated on a dedicated water phantom with and without the *prCRS* in the beam path (the beams passing through the *prCRS* were taken into consideration). Corresponding geometries were set up and executed at the gantry room (Fig.1). Three fields (various depths) and junctions (isocenter) were measured using MatriXX® mounted in DigiPhant® water tank. The gamma indexes (3%/2mm), for isocenters and junctions in both methods exceeded the 90% threshold. The results show that the physical inclusion of the *prCRS* has an impact on the dose distribution and show discrepancies when compared to the standard QA protocol (Tab.1), especially concerning distal planes. An additional TPS planes analysis allowed to estimate the distance to agreement (DTA) within 1-2mm. Obtained results recognize the dosimetry verification as geometry independent within the DTA. Further investigation of the *prCRS* impact on the distal planes discrepancies will be put through.

### P 062 - GPU accelerated calculation of PET activation in proton patients

#### Keegan McNamara^1,2^, Angelo Schiavi^3^, Jan Gajewski^4^, Renata Kopeć^4^, Antoni Rucinski^4^, Tomasz Skóra^5^, Shubhangi Makkar^1,2^, Damien C. Weber^1,6,7^, Antony Lomax^1,2^, Carla Winterhalter^1,2^

##### *^1^Paul Scherrer Institut, Center for Proton Therapy, Villigen, Switzerland*

*^2^ETH Zürich, Department of Physics, Zürich, Switzerland*

*^3^Sapienza University of Rome, Department of Basic and Applied Sciences for Engineering, Rome, Italy*

*^4^Polish Academy of Sciences, Institute of Nuclear Physics, Krakow, Poland*

*^5^National Research Institute, National Oncology Institute, Krakow, Poland*

*^6^University Hospital of Zürich, Radiation Oncology Department, Zürich, Switzerland*

*^7^University Hospital of Bern, Radiation Oncology Department, Bern, Switzerland*

Range validation of proton therapy using PET imaging of the patient requires calculation of the expected production of positron emitting isotopes during treatment. Such calculations are both resource and time expensive using full physics Monte Carlo engines, and so a continuous scoring approach has been implemented in the GPU Monte Carlo engine FRED. The calculation of production of relevant isotopes for offline imaging, ^10^C, ^11^C, ^13^N, ^14^O, ^15^O, ^30^P, and ^38^K, has been performed for 95 head and neck patients treated at CCB Krakow on 2.5x2.5x2.5 mm scoring grids. Calculations in FRED (v3.59.9) were performed on a single machine with two NVIDIA TITAN GPUs. These are validated against simulations using GATE (v9.0 using Geant4 v10.7.1) on a computational cluster of 200-700 CPU cores. The mean production predicted by FRED is within one standard deviation of the production predicted by GATE for each isotope in all 472 fields on a voxel-by-voxel basis, showing excellent agreement overall as in Fig. 1. An increase in calculation speed of, on average, 50.2 times is seen in Fig. 2. This is given by an average factor of 5.5 fewer primaries required for convergence thanks to the variation reduction technique implemented, and a factor 10.3 greater primaries per second simulated using the GPUs. The improved calculation speed and massively lower resource requirements will enable easier implementation of PET validation techniques in proton therapy centers.

### P 063 - Small intra-skull tumor treatments with an active pencil beam scanning proton therapy

#### Daniel Maneval^1^, Thibault Bernelin^1^, Gaëlle Angellier^1^, Baptiste Lhomel^1^, Walid Ouakkad^1^, Claudine Colnard^1^, Marie Vidal^1^, Petter Hofverberg^1^, Joël Hérault^1^

##### ^1^Centre Antoine Lacassagne, Physics, Nice, France

This work aims to define the lower limit tumor size which can be treated with an active proton pencil beam scanning technique using energies up to 226MeV and typical spot sizes up to 1cm. A metrological study was performed to characterize detectors in the context of small targeted volumes. Treatment plans were optimized using RayStation 10 with the robustness parameters set to 3%-3mm and for target volumes ranging from 10cmx10cm to 0.5cmx0.5cm. A quality assurance protocol for the treatment of small tumors was then designed. For this purpose, a Lynx PT and a MatrixxOne were used. Six treatment plans of patients were controlled and treated using this protocol. Absolute dose comparisons between measured and calculated doses yielded a maximum discrepancy of 3.1%. Moreover, 2D gamma index showed a 97.6% passing rate with criteria 3%-2mm, 95.5% with 2%-2mm for target size above 0.5cm. The 95% isodoses were up to 200% larger than the target size above 1cmx1cm, while increasing significantly at lower target size. Furthermore, all patient treatments showed agreement better than 95% with criteria 3%-2mm. Dose computations were in good agreement with measurements for target sizes above 1cmx1cm. The irradiated volume increases significantly below 1cm leading to an over-irradiation of the surrounding healthy tissue due to the finite size of the proton spots. Moreover, robustness evaluation is under investigation but preliminary results suggest that the plans remain robust.

### P 064 - Large chirurgical metal implants management in single field uniform dose proton therapy treatments for an active pencil beam scanning facility

#### Joël Hérault^1^, Benjamin Serrano^2^, Régis Aamblard^2^, Nicolas Garnier^2^, Rémy Villeneuve^2^, Gaëlle Angellier^1^, Petter Hofverberg^1^, Marie Vidal^1^, Baptiste Lhomel^1^, Daniel Maneval^1^

##### *^1^Centre Antoine Lacassagne, Department of Physics, Nice, France*

*^2^Princess Grace Hospital, Department of Physics, Monaco, Monaco*

This work aims to define a protocol for proton therapy treatments in presence of large chirurgical metal implants. Large metal implants (rods, screws and plates), with different chemical composition (titanium, Ti6Al4V or Co28Cr6Mo alloys), were characterized with a pristine Bragg peak and a Spread-Out Bragg Peak (SOBP). For this purpose, the implants were positioned using a thermoplastic mask on a phantom composed of solid water. Range measurements (R90) were done using an EBT3 Gafchromic film was inserted in the middle of the slab phantom, longitudinally to the beam direction. Treatment plans were optimized using RayStation 10. The robustness parameters were set to 5%-5mm for SOBP plans. The implants were delineated using their exact dimensions. Their densities and chemical compositions were overridden to the values provided by the vendor. Metallic artifacts were delineated and overridden to the suitable medium. For the irradiations performed with a pristine Bragg peak, the measured 90% falloff positions matched RayStation's R90 within 2.3mm for the titanium rod and 12.9mm for the Co28Cr6Mo screw. For SOBP robust treatment plans, the R90 positions matched within 1.1 mm for the titanium rod and within 3.2mm for both screws composed of Ti6Al4V or Co28Cr6M. A better range agreement was found when a robust plan optimization was done. Four patients with large implants were treated using the protocol. Additional PTV and PRV margins were set to 5mm where the proton beam crossed the implants. Dual energy CT acquisitions with relative stopping power reconstructions are under investigation to refine the proposed method.

### P 065 - Sensitivity of Carbon-Ion Radiotherapy monitoring using charged nuclear fragments to detector positioning

#### Pamela Ochoa-Parra^1,2,3^, Laurent Kelleter^1,3,4^, Luisa Schweins^1,2,3^, Renato Félix-Bautista^1,3,5^, Jürgen Debus^3,4,5,6^, Oliver Jäkel^1,3,4,6^, Gernot Echner^1,3^, Mária Martišíková^1,3,4^

##### *^1^German Cancer Research Center DKFZ, Department of Medical Physics in Radiation Oncology, Heidelberg, Germany*

*^2^University of Heidelberg, Department of Physics and Astronomy, Heidelberg, Germany*

*^3^National Center for Research in Radiation Oncology NCRO, Heidelberg Institute for Radiation Oncology HIRO, Heidelberg, Germany*

*^4^NCT, National Center for Tumor Diseases, Heidelberg, Germany*

*^5^Heidelberg University Hospital, Department of Radiation Oncology, Heidelberg, Germany*

*^6^Heidelberg University Hospital, Heidelberg Ion Beam Therapy Center HIT, Heidelberg, Germany*

Carbon-Ion Radiotherapy (CIRT) is sensitive to treatment uncertainties, which might lead to changes in the dose distribution. Therefore, online monitoring strategies are highly desirable. In-vivo and real-time monitoring methods based on tracking of nuclear charged fragments emerging from the irradiated patients have been developed by our research group. This research is based on a mini-tracker composed of hybrid semiconductor pixel detectors (Timepix3). All monitoring methods are sensitive to detector positioning errors, which could cause false-positive signals. In this work we investigated the impact of 3D detector displacements on the fragment track distribution. At the Heidelberg Ion-Beam Therapy Center (Germany), a spherical target volume at the center of an anthropomorphic head phantom was irradiated with a clinic-like treatment plan of an RBE-weighted dose of 3 Gy (RBE). Measurements were taken with the mini-tracker placed at different angles relative to the beam axis and distances to the room isocenter. In data post-processing, detector displacements of ±5mm in steps of 1mm along the three spatial coordinates were introduced. It was found that the sensitivity of the fragment track distribution to the detector position was different for the three room axes. The largest difference was observed for displacements along the y-axis, (see Figure 1). Changes of the detector distance to the isocenter (*z-*axis) only had a small effect on the signal. No effect was observed for shifts along the *x-*axis. The obtained data could be used for offline detector misalignment corrections. The results help to determine the required accuracy for the detector positioning.

### P 066 - QA of patient-specific 3D-Printed ridge filter for ConformalFLASH radiotherapy

#### Julia Pakela^1^, Sylvain Deffet^2^, Lucian Hotoiu^3^, François Vander Stappen^3^, Rudi Labarbe^3^, Lei Dong^1^, Eric Diffenderfer^1^, Kevin Teo^1^, Wei Zou^1^

##### *^1^University of Pennsylvania, Radiation Oncology, Philadelphia, USA*

*^2^UCLouvain, Molecular Imaging- Radiotherapy and Oncology MIRO, Louvain-la-Neuve, Belgium*

*^3^Ion Beam Applications IBA S.A., R&D – Clinical Solutions Group, Louvain-la-Neuve, Belgium*

**Introduction**: ConformalFLASH radiotherapy with a patient-specific ridge filter can deliver an advantageous dose distribution while achieving ultra-high dose rates in the patient. A ridge filter was designed for proton pencil-beam scanning and fabricated using a 3D printer. We present the QA process of the ridge filter to identify defects and assess its geometrical accuracy and material uniformity.

**Methods**: A patient-specific ridge filter in acrylic material was designed using a modified version of the open-source MIROpt TPS (UCLouvain) with irregular spikes to achieve desired dose and dose-rate distributions in the patient. A CT scanner was used to obtain 3D images of the ridge filter. The CT image was rigidly registered to the designed ridge filter 3D reference image, and an HU-based threshold segmentation combined with post-processing gave a binary mask that was geometrically compared to the reference mask.

**Results**: In a 3D-printed region of 50x50x30 mm^3^, the minimum, maximum and average distance between mask surfaces were obtained. The average and standard deviation distances between the reference and the CT-scan masks are 0.08 mm and 0.15 mm.

**Conclusion**: In this work, we presented the important quality assurance process of a patient-specific ridge filter. A high resolution CT scan of a 3D-printed ridge filter generated sufficient resolution for geometrical assessment demonstrating the feasibility of a routine QA tool in ConformalFLASH radiotherapy.

### P 067 - First measurements using a preclinical BNCT-SPECT system based on CdZnTe detectors

#### Federico Palmacci^1^, Saverio Altieri^1,2^, Giacomo Benassi^3^, Nicola Sarzi Amadè^3^, Nicoletta Protti^1,2^, Silvia Zanettini^3^, Nicola Zambelli^3^, Valeria Pascali^1^

##### *^1^Pavia University, Department of Physics, Pavia, Italy*

*^2^National Institute of Nuclear Physics INFN, Pavia Unit, Pavia, Italy*

*^3^due2lab s.r.l., R&D due2lab s.r.l., Scandiano, Italy*

Real time dose verification is required to fully exploit the potentiality of Boron Neutron Capture Therapy (BNCT) as personalised medicine. Nowadays dose verification is performed indirectly by measuring B10 concentration on one side and the thermal neutron flux on the other. An on-line measurement of B10 dose is possible by the detection of the 478 keV gamma ray emitted after 94% of capture reactions. With a tomographic system optimised to detect such signal in a SPECT-like manner, a 2D map of the B10 dose could be recorded allowing for a real time monitoring of the treatment. Four “DoseCapture” modules have been recently developed by due2lab s.r.l. with the described purposes and to be tested in the highly thermalised and collimated neutron beam of the Prompt Gamma Neutron Activation Analysis (PGNAA) facility of the LENA laboratory of Pavia University, where a research nuclear reactor operates. Each module is based on an array of 4 Frisch Grid CdZnTe (CZT) detectors coupled to a squared hole lead collimator. To reduce neutron activation of the Cd113 naturally occurring in the alloy, Li-6 enriched TLDs surround five out of six sides of the array. A fast digital electronics complete the detection system. The presentation reports about the preliminary characterisation of this BNCT-SPECT. In particular, the response of the detectors to PGNAA (n+g) field is compared to standard calibration sources while preliminary tomographic capabilities are investigated using simple geometrical phantoms.

### P 068 - Independent verification of the geometric calibration accuracy of an inclined in-room imaging system using absolute laser tracker measurements

#### Rosalinda Ricotti^1^, Andrea Pella^1^, Giulia Sellaro^1^, Alessandro Vai^2^, Matteo Rossi^3^, Ester Orlandi^4^, Mario Ciocca^2^, Guido Baroni^1,3^

##### *^1^National Center of Oncological Hadrontherapy CNAO, Bioengineering Unit- Clinical Department, Pavia, Italy*

*^2^National Center of Oncological Hadrontherapy CNAO, Medical Physics Unit- Clinical Department, Pavia, Italy*

*^3^Politecnico di Milano University, Department of Electronics- Information and Bioengineering, Milan, Italy*

*^4^National Center of Oncological Hadrontherapy CNAO, Clinical Department, Pavia, Italy*

The aim of this study is to verify the accuracy of the analytical isocentric geometric calibration procedure using independent laser tracker measurements. Geometric calibration of the in-room imaging system is usually based on double planar projections of a calibration phantom that contains radiopaque markers. The projection traces of these markers from the x-ray tubes onto the flat panels is used to determine the offsets between the position of flat panels, x-ray tubes and treatment isocenter. The accuracy of the geometrical calibration was independently verified by a laser tracker system (Leica AT-960-MR). 3D coordinates of 32 points were measured using a spherical corner cube retroreflector (CCR) of 1.5′'. The investigated volume was 200x200x200 mm^3 ^around treatment isocenter. At each position, the CCR was imaged with double planar projections. The center of the CCR on each x-ray projection was detected using an automatic thresholding procedure and reconstructed in 3D according to the calibrated geometry (Figure1). Geometric accuracy was estimated by the difference of the 3D points measured by the laser tracker and those calculated with the geometrical reconstruction. Submillimeter geometrical accuracy was measured with median (interquartile-range) of 0.23(0.41)mm 0.19(0.42)mm and -0.46(0.23)mm in lateral, longitudinal and vertical direction respectively. Anatomical features detected on x-ray projections are estimated in the 3D space with submillimeter accuracy. Laser tracker measurements can produce a calibration grid not affected by any positioning inaccuracy suitable for accurate verification in the frame of seldom quality assurance; conversely, the phantom with radiopaque markers fits particularly well for system overall daily quality assurance.

### P 069 - Feasibility study of utilizing Sphinx Compact for quality assurance in uniform scanning proton therapy

#### Suresh Rana^1^, Colton Eckert^2^, Biniam Tesfamicael^2^

##### *^1^Lynn Cancer Institute- Boca Raton Regional Hospital- Baptist Health South Florida, Radiation Oncology, Boca Raton, USA*

*^2^The Oklahoma Proton Center, Radiation Oncology, Oklahoma City, USA*

**Purpose**: To investigate the feasibility of utilizing Sphinx Compact detector for quality assurance in uniform scanning proton therapy system.

**Method:** The Sphinx Compact detector was used to measure various dosimetric parameters at the Oklahoma Proton Center: distal range, distal-fall-off, collinearity, field symmetry, flatness, and field size for four different beams. A specially designed brass aperture was used to perform the required measurements. The Sphinx Compact measurement results were validated against the measurement results from the well-established detectors, namely the IBA Zebra, the Sun Nuclear IC profiler, and the Logos XRV-124. The XRV-124 measurement results were previously validated using EDR2 and EBT3 films. The data collected using the Sphinx Compact was analyzed in myQA software.

**Results:** Based on the data analysis performed, the Sphinx Compact measurements were within acceptable accuracy to the results from the detectors mentioned in Method section. Specifically, the lateral penumbra was within ±0.4 mm, collinearity was within ±0.5 mm, flatness was within ±0.6%, symmetry within ±1.6%, distal range was within ±0.5 mm, distal-fall-off was <0.9mm, and field size was within ±1mm. The reproducibility of the Sphinx Compact was tested for range and collinearity, and the results were within ±0.1 mm.

**Conclusion**: Sphinx Compact detector could potentially replace various other detectors utilized for monthly QA in uniform scanning proton therapy. In a multi-room center, performing the QA with one detector as compared to using multiple detectors dramatically reduces total QA time and the complexity of the QA process.

### P 070 - Design of a preclinical proton minibeam radiotherapy facility: Quality Assurance measures

#### Judith Reindl^1^, Aikaterini Rousseti^1^, Jessica Neubauer^1^, Gerd Datzmann^1^, Michael Mayerhofer^1^, Günther Dollinger^1^

##### ^1^Universität der Bundeswehr München, Institute for applied physics and measurement technology, München, Germany

Proton minibeam radiotherapy (pMBRT) is a method of sparing healthy tissue in the entrance channel of radiotherapy by spationally fractionating the incident proton beam. First preclinical studies in the normal tissue reaction as well as tumor control using mouse ear and rat brain models show the beneficial effects of this new therapy method. Nevertheless, the detailed mechanisms and optimal parameter configurations for getting best possible results by keeping the technical effort low still have to be investigated. Progressing towards clinical usage, pMBRT research should overcome technical and biomedical limitations of the current irradiation test stages and animal models. In this work we show the design of a preclinical pMBRT facility located at an existing 68.5 MeV cyclotron, with special interest in the quality assurance. Most importantly we show simulations on the beam spreading in the planned setups and introduce experimental measures, which can qualify and quantify the beam in a daily measurement procedure. We introduce a possible quality assurance procedure, which has to be conducted to be able to apply the pMBRT in small animals with sufficient small beam size, positioning accuracy as well as lateral and axial dose profiles.

### P 071 - Experimental reaction cross sections of the short- and long-lived β+ isotopes of interest in PET range verification in particle therapy

#### Teresa Rodríguez González^1,2^, Carlos Guerrero^1,2^, Claus Maximiliam Bäcker^3,4,5,6^, Julia Bauer^7^, Christian Bäumer^3,4,5,6,8^, Stephan Brons^7^, M. del Carmen Jiménez Ramos^2,9^, Jorge Lerendegui Marco^1,10^, M. de los Ángeles Millán Callado^1,2^, Jose Manuel Quesada^1^

##### *^1^University of Sevilla, Atomic- Molecular and Nuclear Physics, Sevilla, Spain*

*^2^Centro Nacional de Aceleradores, Centro Nacional de Aceleradores, Sevilla, Spain*

*^3^TU Dortmund University, Department of Physics, Dortmund, Germany*

*^4^University Hospital Essen, University Hospital Essen, Essen, Germany*

*^5^West German Cancer Center, West German Cancer Center, Essen, Germany*

*^6^West German Proton Therapy Centre Essen, West German Proton Therapy Centre Essen, Essen, Germany*

*^7^Heidelberg Ion-Beam Therapy Center HIT, Department of Radiation Oncology- Heidelberg University Hospital, Heidelberg, Germany*

*^8^German Cancer Consortium DKTK, German Cancer Consortium DKTK, Heidelberg, Germany*

*^9^University of Sevilla, Department of Applied Physics II- ETSA, Sevilla, Spain*

*^10^Instituto de Física Corpuscular, CSIC-University of Valencia, Valencia, Spain*

In PET-based beam range verification for particle therapy, new measurements and evaluations of the reaction cross-sections producing β+ emitters are required in order to compare the measured and simulated activity distribution in the patient. We have conducted a comprehensive measurement campaign at several irradiation facilities using different ion types in the therapeutic energy regime and different PET imaging technologies to detect short-lived (online PET monitoring) and long-lived (offline PET monitoring) radionuclides. The following experimental data will be presented: a) The proton-induced production of the long-lived ^11^C (t_1/2_=20 min), ^13^N (t_1/2_=9.9 min) and ^15^O (t_1/2_=2 min) isotopes produced in the main elements of the human body (C, N and O) were measured at the Spanish National Center of Accelerators (CNA) up to 20 MeV, and at the West German Proton Therapy Center (WPE) from 30 up to 200 MeV. The employed method combines the activation of an array of thin foils with the induced activity measurement using a clinical PET scanner. The carbon-induced production of ^11^C was obtained at Heidelberg Ion Therapy Center (HIT) up to 400 MeV/u measuring the activation in graphite foils with NaI scintillation detectors. b) The proton-induced cross-sections of the short-lived ^12^N (t_1/2_=11 ms) in C, ^38m^K (t_1/2_=0.92 s) in Ca and ^29^P (t_1/2_=4 s) in P up to 200 MeV and the carbon-induced cross-sections of ^12^N and ^10^C (t_1/2_=19 s) in C up to 400 MeV/u were measured at HIT. The experiments consist in the activation of pure targets and “online” acquisition with LaBr_3 _scintillation detectors operating in coincidence.

### P 072 - Experimental dose quantification in helium ion therapy using positron emission tomography

#### Harley Rutherford^1^, Andrew Chacon^2^, Daniel Franklin^3^, Akram Mohammadi^4^, Hideaki Tashima^4^, Taiga Yamaya^4^, Katia Parodi^5^, Anatoly Rosenfeld^6^, Susanna Guatelli^7^, Mitra Safavi-Naeini^2^

##### *^1^University of Wollongong, Centre for Medical and Radiation Physics CMRP, Wollongong, Australia*

*^2^Australian Nuclear Science and Technology Organisation, Human Health, Lucas Heights, Australia*

*^3^University of Technology Sydney, School of Electrical and Data Engineering, Sydney, Australia*

*^4^National Institutes for Quantum Science and Technology, Imaging Physics Group, Chiba, Japan*

*^5^Ludwig-Maximilians-Universität München, Department of Medical Physics, Munich, Germany*

*^6^University of Wollongong, Centre for Medical and Radiation Physics, Wollongong, Australia*

*^7^University of Wollongong, School of Physics, Wollongong, Australia*

Quantifying the dose delivered in particle therapy from the spatial distribution of positron-emitting fragments is difficult due to the complex and differing underlying physics processes creating a non-unique relationship between dose deposition and fragmentation. Recently, we developed an iterative approach to estimate the dose deposited by a ^12^C ion beam from the distribution of positron-emitting fragments [1]. In this work we demonstrate the extension of this approach to helium ion therapy, then evaluate the method on experimentally-acquired PET data obtained at the HIMAC beamline, at NIRS (Chiba, Japan) [2]. A library of spatio-temporal positron-emitting fragment 1D deposited dose profiles is constructed by simulating a range of monoenergetic helium ion beams. A linear combination of the monoenergetic positron-emitting fragment profiles is then iteratively fitted to an observed positron-emitting fragment profile from a polyenergetic helium ion beam delivery, to estimate the proportional weight of each monoenergetic component in the beam. The monoenergetic weights are then combined with the corresponding monoenergetic dose profiles to estimate the dose distribution. The method was evaluated for a range of simulated and experimentally deliver helium beam deliveries. For the 14 simulated helium beam deliveries, the method was able to estimate the dose profile with a mean relative error of within 0.3%±0.1%, and estimated the distal edge location to within 1.2mm. For the 4 experimental helium beam deliveries, the dose profiles were estimated with an average mean relative error of 3.9%, and estimated the distal edge location to within 1.2mm. [1] H. Rutherford et. al., 10.1088/1361-6560/abaa23. [2] H. Tashima et. al., 10.1109/NSSMIC.2013.6829046.

### P 073 - Dose estimation in carbon- and helium-ion therapy using convolutional neural networks

#### Rohan Saha Turai^1^, Harley Rutherford^2^, Andrew Chacon^2^, Daniel Franklin^1^, Akram Mohammadi^3^, Hideaki Tashima^3^, Taiga Yamaya^3^, Katia Parodi^4^, Anatoly Rosenfeld^5^, Susanna Guatelli^5^, Mitra Safavi-Naeini^2^

##### *^1^University of Technology Sydney, School of Electrical and Data Engineering, Sydney, Australia*

*^2^Australian Nuclear Science and Technology Organisation, Human Health, Sydney, Australia*

*^3^National Institutes for Quantum Science and Technology, Department of Nuclear Medicine Science, Chiba, Japan*

*^4^Ludwig-Maximilians-Universitat Munchen, Department of Medical Physics, Munich, Germany*

*^5^University of Wollongong, Illawarra Health and Medical Research Institute, Wollongong, Australia*

Positron emission tomography (PET) has been extensively studied for dose estimation and range verification in proton therapy, however the developed methods have not yet been extended to heavier ions. The problem is well-suited to a deep learning approach since the relationship between the distribution of generated positron-emitting fragments and dose is non-linear. In this work, a novel method for estimating one-dimensional physical dose deposition profiles in heavy ion therapy via analysis of dynamic PET images using deep residual convolutional neural networks (CNNs) is proposed and evaluated. Dose deposition and positron annihilation basis profile libraries are generated via Monte Carlo simulations for a range of monoenergetic ^12^C and ^4^He ion beams in a PMMA target. Linear combinations of profiles are used to synthesise a large set of random spread-out Bragg peak (SOBP) dose distributions and corresponding positron annihilation profiles and used to train the CNNs. Network performance is evaluated with a second set of simulated ^12^C ion and ^4^He ion SOBP profiles by using the trained neural networks to estimate dose profiles and distal SOBP edge location against ground truth. The CNN estimated ^12^C SOBP dose profiles with a mean relative error of 0.7%±0.5% and distal edge position with an accuracy of 0.1 mm±0.1 mm, while estimating ^4^He SOBP dose profiles with a mean relative error of 0.4%±0.07% and the distal edge position with an accuracy of 0.1 mm±0.1 mm.

### P 074 - Multivariate statistical modelling enhances the predictive power of Prompt Gamma-Ray Timing for proton treatment verification

#### Sonja Schellhammer^1,2^, Julia Wiedkamp^1^, Steffen Löck^1,3,4^, Toni Kögler^1,2^

##### *^1^OncoRay – National Center for Radiation Research in Oncology, Faculty of Medicine and University Hospital Carl Gustav Carus- Technische Universität Dresden- Helmholtz-Zentrum Dresden - Rossendorf, Dresden, Germany*

*^2^Helmholtz-Zentrum Dresden - Rossendorf, Institute of Radiooncology – OncoRay, Dresden, Germany*

*^3^Faculty of Medicine and University Hospital Carl Gustav Carus- Technische Universität Dresden, Department of Radiotherapy and Radiation Oncology, Dresden, Germany*

*^4^German Cancer Consortium DKTK, Partner Site Dresden- and German Cancer Research Center DKFZ, Heidelberg, Germany*

As a light-weight, collimator-free technique that can be easily integrated into existing systems, Prompt Gamma-Ray Timing is a promising candidate to meet the growing demand for on-line proton treatment verification. The development of such a system is challenging, as the proton range delivered in the patient needs to be reconstructed with high accuracy from the temporal distribution of a very limited number of gamma rays. So far, this reconstruction has been based on the arithmetic mean and standard deviation of the distribution, but the accuracy of this method was found to be insufficient. We therefore developed multivariate statistical models based on additional histogram characteristics to improve proton range reconstruction. Prompt Gamma-Ray Timing distributions acquired during pencil beam irradiation of an acrylic glass phantom with air cavities of different thicknesses were analysed. Relevant histogram features were selected from the Image Biomarker Standardisation Initiative recommendations using forward feature selection and the Least Absolute Shrinkage and Selection Operator (LASSO). Candidate models were defined by multivariate linear regression and evaluated based on their coefficient of determination *R*^2^ and root mean square error *RMSE* on an independent dataset. The newly developed models showed a strongly improved predictive power (*R*^2^ > 0.6) compared to the previously used models (*R*^2^ < 0.1) and enabled the identification of introduced air cavities in a scanned treatment field (Figures 1–2). These results demonstrate that elaborate statistical modelling is a valuable tool to enhance the Prompt Gamma-Ray Timing method and increase its potential to be used for proton treatment verification.

### P 075 - Line-of-response (LOR)-based mid-range probing: towards online proton range verification and plan adaptation

#### Lin Ma^1^, Mingli Chen^1^, Dongxu Yang^1^, Yuncheng Zhong^1^, Xun Jia^1^, Xuejun Gu^1^, Yiping Shao^1^, Weiguo Lu^1^

##### ^1^University of Texas Southwestern Medical Center, Radiation Oncology, Dallas, USA

Range verification is critical in proton therapy. Mid-range probing strategy combined with in-beam positron emission tomography (PET) is promising for in-vivo range measurement and on-line plan adaptation. We performed an end-to-end Monte Carlo (MC) simulation from beam delivery to PET acquisition, proposed a novel range measurement strategy and conducted measurement uncertainty analysis. The end-to-end MC simulation was performed in two steps. First, we simulated proton transportation with gPMC_v3.0, an in-house GPU-accelerated proton MC package, which outputs dose and positron emitter distributions. 0.04∼0.08 Gy Dmax corresponded to 10∼20 O^15^ per mm^3^. Second, we simulated PET acquisition (from decay to coincident events) with Gate to obtain LORs detected by PET. The simulation modeled an in-house developed PET scanner (fig1), which had ∼3% sensitivity (#LOR/#decay). We proposed an LOR-based range measurement without PET reconstruction. A volume of interest (VOI) (fig1.c) was selected at distal end to accommodate potential range shift. Only the LORs intersecting VOI (fig1.c) were preserved. We defined the “activity range surrogate” (fig2.a) as the closest point to the preserved LORs. With simulated over-shoot and under-shoot range shifts, we established the correspondence between the surrogate and range, which can be regarded as the calibration curve (fig2.b). For a real measurement, we can acquire LORs, calculate the surrogate, and look up for the beam range using this curve. We further calculated the uncertainty of measured range (fig2.c) using MC simulation to guide spot and acquisition time selection. For a spot with Dmax of 0.4Gy and 60-second acquisition, one standard deviation of range measurement was 0.75mm.

### P 076 - Validation of a novel plan specific interplay QA method using routine plan QA static 2D array measurements

#### Kees Spruijt^1^, Jeremy Godart^1,2^, Yibing Wang^1^, Sven Meijer^1^, Atia Ibrahimi^1^, Renske de Jong^1^, Mischa Hoogeman^1,2^

##### *^1^HollandPTC, Radiation Oncology, Delft, Netherlands*
*^2^Erasmus University Medical Center, Department of Radiotherapy, Rotterdam, Netherlands*

**Introduction**: Moving targets treated with IMPT are subject to the interplay effect. To determine the robustness of fractionated treatments against interplay at the treatment plan level, we developed and validated a QA method that simulates the impact of interplay on fractionated treatments from static 2D array measurements that are routinely used for patient QA.

**Methods**: Three mono-energetic line profiles (70, 140, and 210 MeV) were irradiated on a moving 2D-array for a series of sinusoidal motion patterns with different combinations of period (2, 4, and 6 s) and amplitude (5, 10, 15, and 20 mm). The same motions were also simulated by an in-house developed application on statically measured doses. Dose output of the dynamic measurements and simulations were compared using gamma evaluation (2mm/2%). We applied the same methodology on two lung treatment plans with periods of 4 and 6 seconds and amplitudes of 5, 10, and 20 mm. Dose output was compared to validate that the simulated measurements (foreseen QA method) give similar results on the impact of interplay as the dynamic measurements (reference).

**Results**: All results are presented in figure 1. For the line profile plans of 70, 140, and 210 MeV, the median gamma results were 99.5%, 96.1%, and 93.6% respectively. The median gamma was ≥98.1% for the lung treatment plans.

**Discussion**: The high gamma pass rates confirm that static 2D array measurements acquired routinely for patient QA combined with motion simulations can be used to replace labor intensive dynamic measurements in assessing the impact of interplay.

### P 077 - Neutron dose estimation for a foetus from proton pencil beam scanning radiotherapy of brain and breast targets

#### Liliana Stolarczyk^1^, Anne Vestergaard^1^, Christian Skou Søndergaard^1^, Line Bjerregaard Stick^1^, Stine Elleberg Petersen^1^, Maria Fuglsang Jensen^1^, Kenneth Jensen^1^, Peter Magnus Trock Lægdsmand^1,2^

##### *^1^Aarhus University Hospital, Danish Centre for Particle Therapy, Aarhus N, Denmark*

*^2^Odense University Hospital, Department of Oncology- Laboratory of Radiation Physics, Odense, Denmark*

Complication with cancer is a rare problem occurring in about one in 1,000 pregnancies. Potential adverse effects of foetal radiation exposure may result in radiotherapy being postponed or replaced with other treatment methods. Proton spot scanning therapy may allow optimal cancer treatment with simultaneous foetus protection. At the Danish Centre for Particle Therapy (DCPT) we have estimated the neutron ambient dose equivalent H*(10) for a foetus (week 30) for two realistic clinical scenarios: brain and breast targets (Table1). Plans were calculated with IMPT technique (Eclipse_v16.1).Plan geometry and spot maps were transferred to Monte Carlo calculations (TOPAS v3.5/Geant4 v.10.6) [1] and neutron H*(10) was simulated on the University of Florida (UF) phantom [2] (Fig.1). Measurements were carried out at a position of the belly using Wendi-II (Thermo Scientific™) and solid water phantom representing a 170 cm and 80 kg pregnant woman. Total neutron H*(10) measured for the brain target was equal 0.6 mSv. Simulated mean H*(10) for the uterus, an estimation for foetus neutron dose, was equal 0.3 mSv and 12 mSv for brain and breast targets, respectively. Dose to a foetus for proton PBS radiotherapy is considerably lower than for photon radiotherapy (tens of mSv [3]). Moreover, for PBS there is no need for additional shielding or special consideration during treatment planning. However, foetal doses can still be minimised by avoiding vertex fields and use of range shifters. References: [1] Med Phys 39, 6818 (2012). [2] Phys Med Biol 59, 4325 (2014). [3] Med Phys 34, 1193 (2007)

### P 078 - Secondary neutron and gamma-ray flux measurement for range verification in hadron therapy

#### Jianxin Zhou^1^, Angela Di Fulvio^1^

##### ^1^University of Illinois at Urbana Champaign, Nuclear- Plasma- and Radiological Engineering, Urbana, USA

Improved range verification can reduce the safety margins in treatment planning and increase the treatment conformality in hadron therapy. In this work, we studied the feasibility of a real-time range verification method based on the detection of secondary neutrons and gamma-rays with a recently-developed organic scintillator: deuterated trans-stilbene (stilbene-d_12_). Stilbene-d_12_ can detect both gamma rays and neutrons in the MeV range and efficiently discriminate between them. We studied this method for 150MeV protons irradiating a 30cm x 30cm x 15cm water phantom. We simulated the beam interactions and the secondary particle creations with FLUKA. A detector array with 9-cm thick stilbene-d_12_ cells with a 3-mm pitch between detectors was placed outside the phantom. The detectors' front surfaces were perpendicular to the beam axis. Particle flux, which reached the detector surfaces, was recorded. We accurately simulated the detector responses of these cells using MCNPX-PoliMi, which can render the event-by-event response and incorporate a realistic light output function. We used the spatial distribution of the detector responses to measure the range. [Figure 1-"Detector response to gamma-ray flux outside the phantom” and

Figure 2-"Detector response to neutron flux outside the phantom” show] the simulated results. For both measurements, the 55% fall-off of the detector counts (35.8 plus-minus 0.1 cm) agreed well with the actual range (35.8 cm). Measuring both gamma-ray and neutron signatures can verify the range in real-time with smaller systematic uncertainty compared to using a single signature. The method is being validated for different proton energies as well as for carbon-ion beams.

### P 079 - Determining the number of photon and particle fractions for jointly optimized combined treatments

#### Amit Ben Antony Bennan^1^, Jan Unkelbach^2^, Niklas Wahl^1^

##### *^1^German Cancer Research Center DKFZ, Department of Medical Physics in Radiation Oncology, Heidelberg, Germany*

*^2^University Hospital Zurich, Department of Radiation Oncology, Zurich, Switzerland*

**Motivation**: Treatments combining photon and particle fractions are currently optimized separately. Recent research devised simultaneous optimization of multi-modality treatments within the LQ-model for photon-proton combinations and photon-carbon treatments [1],[2]. We investigate the impact of the number of particle fractions in combined treatments on tradeoffs between planning goals.

**Methods**: We consider 5 fraction SBRT treatments of spinal metastasis with three main competing goals: deliver 5x7Gy photon-equivalent dose to the CTV (α/β=10), protect spinal cord (α/β=2), and minimize integral dose. Photon-proton (RBE=1.1) and photon-carbon (LEM-IV) treatments were jointly optimized for varying numbers of particle fractions using objective functions evaluated for cumulative biological effect (-ln(S)). Optimized combinations were compared to simple combinations where each modality delivers 7Gy(RBE) per fraction.

**Results**: Figure 1(b,d,f) shows a jointly optimized treatment using 4 photon and 1 carbon fraction. Photons deliver dose to tissue surrounding the spinal cord, protecting it through fractionation. Carbon ions deliver an overproportionate dose to the remaining CTV, reducing integral dose in the abdomen, compared to the simple combination (Figure 1(a,c,e)). Figure 2 shows dependence of plan quality indicators on the number of particle fractions. For protons and carbon ions, more particle fractions reduce integral dose (Figure 2b). However, more carbon fractions worsen the tradeoff between target coverage and spinal cord sparing (Figure 2c) due to the reduced fractionation effect of high-LET irradiation. Thus, optimal photon-carbon treatment may use 1 or 2 carbon fractions, depending on tradeoffs. Jointly optimized treatments generally improve on simple combinations. [1] Unkelbach,J.,Fabiano,S.,Bennan,A.B.A.,Mueller,S.,Bangert,M.,2021. IEEE Trans.Radiat.PlasmaMed.Sci. 1–1. https://doi.org/10.1109/TRPMS.2021.3092423. [2] Bennan,A.B.A.,Unkelbach,J.,Wahl,N.,Salome,P.,Bangert,M.,2021. Int.J.Radiat.Oncol. https://doi.org/10.1016/j.ijrobp.2021.05.126

### P 080 - Including variable RBE in IMPT optimization for pediatric brain cancer patients

#### Hadis Moazami Goudarzi^1^, Gino Lim^1^, David Grosshans^2^, Radhe Mohan^3^, Wenhua Cao^3^

##### *^1^University of Houston, Industrial Engineering, Houston, USA*

*^2^MD Anderson Cancer Center, Radiation Oncology, Houston, USA*

*^3^MD Anderson Cancer Center, Radiation Physics, Houston, USA*

**Purpose**: To study the impact of considering variable relative biological effectiveness (RBE) of protons in optimization of intensity-modulated proton therapy (IMPT) treatment plans for pediatric brain patients.

**Methods**: Five pediatric brain cancer patients were included in this study. A phenomenological RBE model was used in variable RBE optimization minimizing the average deviation between calculated and prescribed doses. In addition, two other alternative optimization approaches were implemented for comparison, including linear energy transfer (LET) optimization using a composite objective function for optimizing LET and physical dose simultaneously and standard optimization assuming constant RBE. An analytical algorithm was used to compute dose and LET for all plans.

**Results**: Target coverage and normal tissue sparing in terms of variable RBE weighted dose in all plans generated by variable RBE optimization were comparable to those in terms of constant RBE weighted dose in LET optimized plans or standard plans. By evaluating variable RBE weighted dose, LET optimized plans and standard plans tended to overdose critical structures near target volumes such as brainstem and spinal cord. Meanwhile, LET optimization could consistently achieve increased LET in target volumes and reduced or similar LET in normal tissues compared variable RBE optimization and standard optimization.

**Conclusion**: Variable RBE optimization is important to ensure plan quality when variability in RBE needs to be considered. It is also important to evaluate variable RBE of LET optimized plans or conventionally optimized plans when a constant RBE is assumed.

### P 081 - Optimizing carbon ion radiotherapy: Fixed beam or rotating gantry? That is the question

#### Siven Chinniah^1^, Amanda Deisher^2^, Michael Herman^2^, Jedediah Johnson^2^, Anita Mahajan^2^, Robert Foote^2^

##### *^1^Mayo Clinic, School of Medicine, Jacksonville- FL, USA*

*^2^Mayo Clinic, Radiation Oncology, Rochester- MN, USA*

**Purpose:** This study evaluates whether fixed beam or rotating gantry systems would provide the most optimal treatment plan for carbon ion radiotherapy (CIRT).

**Methods and materials:** We retrospectively evaluated patients with primary tumors treated with pencil beam scanning intensity modulated proton radiotherapy at our institution from 2015 to 2020 who had indications for CIRT. All patients were treated with a 190° rotating gantry with a robotic patient positioning system. Treatment plans were individualized to provide optimal dose delivery to the tumor target while sparing healthy organs at risk. Beam angles utilized were grouped, and anatomic sites with at least 10 different beam angles were sorted into histograms.

**Results:** A total of 467 patients with 484 individualized plans and 1196 unique treatment beam angles were evaluated and characterized by anatomic treatment site, mean treatment beam angle, and range of beam angles. The most common beam angles used were 0° (direct anterior) and 180° (direct posterior). A wide range of beam angles were used in treating almost all anatomic sites, with only esophageal cancers having a strong unimodal grouping of beam angles (112 beam angles; 84% fit in the 160° ± 10° range). Pancreas cancers showed moderate grouping of beam angles (42 beam angles; 62% fit in the 155° ± 10° range).

**Conclusions:** The wide distribution of beam angles used to treat CIRT-eligible patients at our institution suggests that a rotating gantry is more capable of providing optimal beam arrangements than fixed beam systems. Future technologies for treatment positioning and imaging are discussed.

### P 082 - Systematic errors at intermediate source-to-axis distance (SAD)

#### Benjamin Clasie^1^, Hanne Kooy^1^, Brian Winey^1^, Nicolas Depauw^1^

##### ^1^Massachusetts General Hospital- Harvard Medical School, Radiation Oncology, Boston, USA

We expose potential systematic errors in proton pencil beam scanning (PBS) dose calculations, simulations, and treatments. The corrections described are present in all finite-SAD systems but become more important at small SAD, a direction that allows for smaller gantries and smaller vault size. Systematic errors occur when the angle of the beam with respect to the nozzle axis increases. This angle reduces the number of protons delivered per MU due to the longer proton pathlength in the monitor chamber. As a result, a measurement with uniform gigaprotons per spot and uniform spot spacing does not result in uniform dose in a measurement plane. Treatment machines should correct for the increase in pathlength of protons in the monitor chamber or have consistent meterset between the machine, dose calculations and Monte Carlo simulations. The pencil beam angle also changes the shape of the beam projected onto voxels which can be challenging to model analytically, but the onset of this effect (SAD=59-98 cm, depending on the field size) occurs after the onset of the pathlength correction (SAD=120-200 cm, depending on the field size). Intermediate SAD gantries have SAD between these two onsets. Intermediate SAD gantries deserve more discussion especially in the context of PBS (which reduces the entrance dose vs. double scattering), proximal targets, multiple beam directions, and/or arc therapy. In conclusion, systematic errors due to the scanned beam angle are described as well as the onset of these corrections. This work helps to make proton therapy more accurate and accessible.

### P 083 - Assessment of IMPT and VMAT for bone marrow sparing in prostate bed and lymph node treatment

#### Mariluz De Ornelas^1^, Elizabeth Bossart^1^, Giuseppe Carlo Iorio^2^, Crystal Seldon^1^, Alan Dal Pra^1^, Michael Butkus^1^

##### *^1^University of Miami, Radiation Oncology, Miami, USA*

*^2^University of Turin, Radiation Oncology, Turin, Italy*

**Purpose**: To compare photon and proton treatment plans for bone marrow sparing (BMS) to potentially reduce hematologic toxicity.

**Materials and methods**: Twenty patients previously treated to the prostate bed and pelvic lymph nodes with volumetric arc photon therapy (VMAT) were retrospectively contoured to include the whole pelvic bone marrow (WPBM) and sub-volumes: lumbar-sacral (LSBM), iliac (ILBM), and lower pelvis (LPBM). Three additional plans were created: VMAT with BMS sparing, intensity modulated proton therapy (IMPT) and IMPT with BMS sparing. Standard planning methods were used for both VMAT and robustly optimized (±5mm/±3.5%) IMPT plans. Optimization of BMS plans was done as to not compromise target volume coverage or non-BMS OAR dose constraints. All plans were normalized to have prescription dose covering 95% of the treatment volume. Maximum, mean, and volumes receiving different dose levels were analyzed and compared. Statistically significant differences were assessed using Wilcoxon signed rank test (p≤0.05).

**Results**: Mean dose to all bone marrow (BM) structures were significantly lower for VMAT_BMS, IMPT, and IMPT_BMS compared to VMAT. IMPT_BMS had significantly lower mean doses for all BM structures. Mean and maximum doses to the femoral heads were significantly lower for VMAT_BMS compared to IMPT_BMS. Volumes receiving dose above 50 Gy within ILBM and LSBM were significantly higher for proton plans compared to photon plans.

**Conclusions**: BMS is achievable with VMAT and IMPT without compromising target coverage or OAR constraints. IMPT was better at reducing the mean and low doses to the BM structures but had larger high dose volumes in ILBM and LSBM.

### P 084 - Dosimetric advantages of small-SAD PBS delivery

#### Nicolas Depauw^1^, Hanne Kooy^1^, Brian Winey^1^, Benjamin Clasie^1^

##### ^1^Massachusetts General Hospital, Radiation Oncology, Boston, USA

Proton pencil beam scanning (PBS) delivery systems with reduced source-to-axis distance (SAD) necessitate smaller radii for beamline magnets, and therefore reduce the size of the outer radius for the gantry. This, in part, can help make proton therapy more accessible. Additionally, a small SAD, as defined as <59 or <98 cm, depending on the field size, may present unique dosimetric advantages. As depicted in Figure 1, the entry angle in the patient, resulting from the smaller SAD, potentially allows greater sparing of organs-at-risks (OAR). It may also permit the use of more beams than on a large-SAD machine for those cases where the entrance dose is a concern. In this work, we evaluate the dosimetric effects of a smaller SAD in three different sites of interests: a spine sarcoma (located between the kidneys), a breast patient (skin dose sparing), and a CNS (optics OAR proximity and skin dose sparing). Plans were generated with two machines sharing the same beam model at the exception of their SAD. The SAD was set to a value of 200 cm for the first machine (large-SAD), and 60 cm for the second one (small-SAD). Dose distributions and DVH metrics were evaluated in order to assess the potential benefits of using a PBS treatment delivery system with a reduced SAD.

### P 085 - A novel simultaneous plan quality and beam delivery time SPArc optimization platform using Alternating Direction Method of Multipliers (ADMM)

#### Lewei Zhao^1^, Juntao You^2^, Gang Liu^3^, Xiliang Lu^4^, Xuanfeng Ding^1^

##### *^1^Beaumont Health System, Department of Radiation Oncology, Royal Oak, USA*

*^2^Hong Kong University of Science and Technology, Department of Mathematics, Hong Kong, China*

*^3^Huazhong University of Science and Technology, Cancer center- Union Hospital- Tongji Medical College, Wuhan, China*

*^4^Wuhan University, School of Mathematics and Statistics, Wuhan, China*

**Purpose/Objectives:** Proton arc is a new treatment modality, which delivers proton beams while the gantry is continuously rotating. This study proposed a regularized l_0_-minimization alternating direction method of multipliers (ADMM) algorithm for proton arc spot sparsity optimization to simultaneously optimize the plan quality and the beam delivery time (BDT).

**Methods:** Based on the previously published beam delivery sequence model of IBA ProteusONE^®^ from our institution is dominated by spot switching time (SSWT). SSWT is approximately linearly depended on spot numbers. So we used a non-convex l_0-_norm to control the sparsity level of the regularized solution. The clinical objective is formulated as a l_2_-norm. The proton arc optimization problem is then rewritten in ADMM form as equality-constrained optimization problem. Brain and lung clinical cases are used to validate the optimized result. l_2_-norm value is calculated for evaluating clinical objective as well as plotting DVH. Both the objective value and optimization time are compared with Spot-Scanning Proton Arc (SPArc-original) algorithm.

**Results:** The results showed that ADMM can ensure both spot and plan quality (Figure1). This new planning framework could effectively improve the optimization speed by a factor of about four (2.6 times to 6.6 times from 20%-60% sparsity) compared to the SPArc-original (Figure 2).

**Conclusion:** This study introduced the first simultaneously optimize the plan quality and BDT SPArc optimization platform utilizing the ADMM. Additionally, the successful implementation of the ADMM algorithm into SPArc can significantly improve the optimization time, which is a critical step forward in the era of proton arc therapy.

### P 086 - Optimized implementation of a 4D-pencil beam algorithm for RBE-weighted doses in TRiP98

#### Christian Graeff^1^, Moritz Wolf^1^

##### ^1^GSI, Biophysics, Darmstadt, Germany

Carbon ion therapy requires non-linear RBE-weighted dose calculation, which imposes a significant computational cost. We re-implemented the LEM-based dose engine in TRiP98 to improve computation times especially for 4D and robust optimization. Following the low-dose approximation of the RBE [Krämer 2006], we restructured the dose matrix computation so that variables constant for a given energy and water-equivalent thickness are precomputed and stored only once. This includes alpha, beta, depth dose contribution and LET. Only the lateral dose contribution from a double-Gaussian model is stored for each beam and dose voxel combination. For iterative inverse optimization, this matrix is held in memory, which often constitutes a bottleneck for 4D-robust optimization. The changes reduced code complexity and the number of function calls. For demonstration, the old and new implementation was compared using the same executable on the same machine (Intel Xeon 8 cores, 16 GB RAM), for a 2-field IMPT plan on a lung and an H&N case. In both cases, plans were optimized with conventional margins and a 9-scenario worst-case robust optimization. For the lung, a 4D-optimization on 4 motion phases was conducted and a 4D-dose distribution was calculated on 10 motion phases. The computed dose cubes were identical for the same input. The optimized fields differed slightly due to the streamlined iterative process, resulting in differences of target coverage D95 < 0.5%. The table states computation times and storage sizes. In conclusion, the new implementation could significantly speed up the dose computation, especially for robust optimization and 4D-dose calculation.

### P 087 - Statistical Analysis of an imbalanced case-control cohort of paediatric posterior fossa tumour patients with symptomatic brainstem necrosis

#### Andreas Havsgard Handeland^1,2^, Daniel J Indelicato^3^, Lars Fredrik Fjæra^2,4^, Kristian S Ytre-Hauge^2^, Helge Egil S Pettersen^1^, Yasmin Lassen-Ramshad^5^, Ludvig P Muren^6^, Camilla H Stokkevåg^1,2^

##### *^1^Haukeland University Hospital, Department of Oncology and Medical Physics, Bergen, Norway*

*^2^University of Bergen, Institute of Physics and Technology, Bergen, Norway*

*^3^University of Florida, Department of Radiation Oncology, Jacksonville, USA*

*^4^Oslo University Hospital, Department of Medical Physics, Bergen, Norway*

*^5^Aarhus University Hospital, Danish Centre for Particle Therapy, Aarhus, Denmark*

*^6^Aarhus University Hospital, Department of Medical Physics, Aarhus, Denmark*

**Introduction**: In the treatment of paediatric posterior fossa tumours, the brainstem is a sensitive organ close to the tumour site. Due to the uncertainty of the proton relative biological effectiveness (RBE) the brainstem may be subject to higher dose than the clinically applied RBE-factor of 1.1 would yield. This study investigated a case-control group of paediatric patients with brainstem necrosis possibly attributed to proton variable RBE, with a subgoal of minimising statistical bias and uncertainties introduced by various hypothesis tests.

**Method**: Dose volume histograms (DVHs) and FLUKA-recalculated dose and dose-averaged linear energy transfer (LETd) distributions were extracted for 36 patients (1:3 case-control ratio) matched on both dosimetric and clinical factors. Conditional logistic regression (LR) was used to establish statistical significance, which minimises bias from matching criteria. However, normal distribution is assumed leading to bias for small sample sizes. Hence, the conditional LR results were compared with unconditional LR accounting for model fit to the data points, Wilcoxon signed rank tests independent of the normal distribution and Chi-squared tests for the conditional LR.

**Results**: Variable RBE led to increased RBE-wighted dose (Figure 1). However, *p* values describing the difference between cases and controls were mostly insignificant and varied between the test (Figure 2). The conditional LR model was considered the most relevant test due to accounting for matching criteria.

**Conclusion**: Slightly increased LETd was observed to cases compared to controls, although this was difficult to establish with statistical uncertainty and the tests disagreed due to inherent assumptions and statistical biases.

### P 088 - Towards a generalized (N)TCP optimization framework across modalities using classical and machine learning models

#### Jennifer Hardt^1,2,3^, Tim Ortkamp^1,4,5^, Niklas Wahl^1,3^

##### *^1^German Cancer Research Center, Medical Physics in Radiation Oncology, Heidelberg, Germany*

*^2^Ruprecht Karl University of Heidelberg, Faculty of Physics and Astronomy, Heidelberg, Germany*

*^3^Heidelberg Institute for Radiation Oncology-HIRO, Medical Physics in Radiation Oncology, Heidelberg, Germany*

*^4^HIDSS4Health, Helmholz Information and Data Science School for Health, Karlsruhe/ Heidelberg, Germany*

*^5^Karlsruhe Institute of Technology, Steinbruch Centre for Computing, Karlsruhe, Germany*

Finding the best possible trade-off between tumor-control-probability (TCP) and normal-tissue-complication-probability (NTCP) defines the basis rationale of radiotherapy treatment plan optimization. However, planning using (N)TCP based objective functions has mostly been used for photons and with classical, logistic (N)TCP models with few variates, which may not accurately capture the complex relationship between applied dose and therapeutic outcome. This work explores the technical feasibility of a generalized (N)TCP optimization framework incorporating particle therapy and multivariate machine learning (ML) (N)TCP models. Dose calculation and optimization are conducted in the open-source toolkit matRad, extended to compute the biologically effective dose (BED) for photons, protons (RBE=1.1) and carbon ions (LEM-IV). An (N)TCP optimization module featuring classical models, i.e., the Lyman-Kutcher-Burman (LKB) model and the Niemierko model, was implemented. In addition, an exemplary, differentiable ML classification model, i.e., a neural network, was trained on morphological dose features from 77 head-and-neck patients and for the endpoint of grade 2+ long-term xerostomia (Gabryś et al 2018). Auto-differentiation computates model and feature gradients w.r.t. dose during inverse planning with interior-point quasi-Newton optimization. (N)TCP based optimization for photon, protons and carbon ions was executed on a prostate and head-and-neck patient (CORT-Dataset), demonstrating technical feasibility of an effect-based BED for (N)TCP optimization with photons, protons, and carbon ions (Figure 1). Further, auto-differentation facilitated the use of the neural network NTCP model in optimization, effectively reducing the predicted NTCP for photons and protons (Figure 2). More sophistically obtained models may then enable personalized, AI-based plan optimization beyond technical feasibility within the presented framework.

### P 089 - Sensitivity of RBE and biological dose to variations in model parameters across common models in carbon radiotherapy

#### Shannon Hartzell^1^, Paige Taylor^1^, Rebecca Howell^1^, Christine Peterson^2^, Stephen Kry^1^

##### *^1^The University of Texas MD Anderson Cancer Center, Radiation Physics, Houston, USA*

*^2^The University of Texas MD Anderson Cancer Center, Biostatistics, Houston, USA*

Variable RBE in carbon radiotherapy may be calculated using several models, including the microdosimetric kinetic model (MKM), adapted stochastic MKM (SMKM), and repair misrepair fixation (RMF) model. This work compares the sensitivity of each of these models to variations in input biological and reference parameters. Geant4 Monte Carlo was used to simulate clinically realistic monoenergetic carbon beams (ranges 5/15/25 cm) incident on a water phantom. Input parameters for RBE models (microdosimetric spectra, double strand break yield, physical dose) were scored. Data were used along with cellular and model specific parameters to calculate linear (α) and quadratic (β) components, which were combined with reference α and β values and physical dose to calculate RBE. Model sensitivity to various parameters were quantified by statistically introducing uncertainty into independent parameters over 1000 iterations and sampling resultant RBE. Reference cell lines were also varied to assess RBE differences due to histology. Results of the sensitivity study to variation in input parameters are shown below, demonstrating that microdosimetric models had greater sensitivity to changes in reference α, β, and physical dose than RMF. Within microdosimetric models, changes in domain radius had the largest effect on RBE. Both RBE and model sensitivity differed notably across cell lines, and even with different reported values within a single cell line. While focus is largely placed on comparing models, the variation within each based on reference parameters alone is drastic. This study gives insight robustness of models and emphasizes need for continued computational and in vitro RBE research.

### P 090 - On-axis Monte Carlo pencil beam scanning model validation for SFPTI using TOPAS

#### Shreen Ibrahim^1^, Charles Shang^2^, Wazir Muhammad^3^

##### *^1^Florida Atlantic university, physics, boca raton, USA*

*^2^South Florida Proton Therapy Institute, South Florida Proton Therapy Institute, Delray beach, USA*

*^3^Florida Atlantic University, department of physics, Boca Raton, USA*

The objective of this work is to model the proton beam line of Varian ProBeam proton therapy system located in South Florida Proton Therapy Institute (SFPTI) using Geant4-based TOPAS Monte Carlo toolkit (version 3.7). The phase space characteristics of the proton beam modelling have been validated against the measurements including the energy spectrum, lateral spot profiles.. Emittance was the beam source to extract the spot profiles according to Courant-Snyder's theory for four energies selected (70, 110, 160 and 220) MeV. In-air spot profiles applied to get the beam size. Spot profiles for (±10, ±20) cm upstream and downstream distance from the isocenter implemented. Gaussian beam has been used to simulate the dose distribution for the energy spectrum (70 to 220 MeV) in an interval 5MeV for 10^5 ^incident proton particles. Proton interaction physics list pertained to Geant4 used in this work includes “g4em-standard_opt3” “g4h-phy_QGSP_BIC_HP” “g4decay” “g4h-elastic_HP” “g4ion-binarycascade” “g4stopping”. The normalized Bragg peak curves extracted and the distal depth at 80% of the maximum dose deposition was defined as proton range compared to the measured integrated depth dose. The results show 95-100 % of the dose points tested within (2%/2 mm) gamma index. The simulated in-air spot profiles showed a good agreement with the measured data for 70 MeV and 220 MeV. According to the proton Bragg peak simulation and spot profiles results the TOPAS based Monte Carlo Varian ProBeam beam model is ready to use in further proton therapy investigations.

### P 091 - Application of knowledge-based auto treatment planning for proton therapy of high-risk prostate cancer using simultaneous integrated boost

#### Pingfang Tsai^1^, Casey Johnson^1^, Shaakir Hasan^1^, Sheng Huang^1^, Andy Shim^2^, Christine Oh^2^, Jack Tung^1^, Francis Yu^1^, Haibo Lin^1^

##### *^1^New York Proton Center, Physics, New York, USA*

*^2^New York Proton Center, Dosimetry, New York, USA*

**Purpose**: To implement and validate the knowledge-based proton auto-planning model for high-risk prostate cancer patients.

**Methods**: The RapidPlan module in the Eclipse treatment planning system (v16.1) was implemented to train the auto planning model with datasets of thirty patients who received simultaneous integrated boost (SIB) with 70.2Gy to prostate and seminal vesicle and 46.8Gy to the pelvic lymph nodes in 26 fractions. The model was verified by analyzing the goodness-of-fit summary statistics and identifying possible outliers. The established model was validated on five new cases. The RapidPlan plans (RPP) quality were compared to expert plans (EP) in terms of target coverage and sparing of seven organs-at-risks (OARs) using clinical constraints.

**Results**: The coefficient of determination and chi-squared of training results were 0.74±0.15 and 1.16±0.08 for OARs. The average optimization time is less than 15 minutes for one run. For RPP, D95% of the high and low dose targets were 70.5±0.2Gy and 46.8±0.1Gy, respectively. The Dmax dose evaluators of the RPP show 1.3 Gy deviation from EP. The volumetric dose evaluators between RPP and EP show minimal difference (0.06±0.7Gy). The *t*-test indicates no significant difference between EP and RPP, except the Dmax dose for the rectum (p=0.04).

**Conclusion**: The RapidPlan auto planning approach can generate competent plans with adequate target coverage and comparable OAR sparing compared to the EP for high-risk prostate patients with reduced planning time and efforts.

### P 092 - Study of variable RBE (Radiobiological Effectiveness) based planning techniques for proton therapy

#### Hisham Kamal^1^, Peng Wang^1^

##### ^1^Inova Schar Cancer Institute, Radiation Oncology, Fairfax, USA

**Purpose**: Comparative Study of various forward and inverse planning techniques that incorporate dose averaged LET

**Materials and methods**: A new Monte Carlo based dose computation with the ability to report the dose average LET ( RayStationIonPG Research9) with variable RBE modeling was used in this study. The tool has the ability to report RBE dose and optimize RBE related quantities. Semi Forward planning (SFP) and inverse planning (IP) techniques were evaluated retrospectively on 10 patients (3 brain and 3 breast). The SFP was performed by optimizing the target coverage and OAR sparing using the physical dose in addition to auxiliary objectives based on the biological hot spots. In IP, both physical and biological objectives were included in the optimization objectives. Several RBE models were used in this study including Carabe and McNemara's models.

**Results**: For the brain patients, plans with 4 beams showed lower biological dose to OAR compared to 3 beam plans. An observed reduction in the RBE dose to OAR were also observed as the beam angle separation increased between the beams. For the breast patients, three beam plans with larger angular separations resulted in lower biological dose to OAR. The reduction in the RBE dose due to larger angular separation was less significant compared to the brain patients. Both SFP and IP showed similar results in target coverage, while the max OAR RBE dose difference (Dmax and Dmean) was within 6% on average between the two methods.

### P 093 - Protons or photons? A moving target's dilemma: Can volumetric repainting mitigate interplay effect in a compact superconducting synchrocyclotron proton system?

#### Asadulla Khan^1^, Francesca Fiorini^1^, Jamil Lambert^2^

##### *^1^Rutherford Cancer Centre Thames Valley, Medical Physics, Reading, United Kingdom*

*^2^Rutherford Cancer Centre South Wales, Medical Physics, Newport, United Kingdom*

**Introduction:** Previous studies have investigated the effect of volumetric repainting, using proton machines including IBA ProteusPLUS; however, similar studies using the IBA ProteusONE are not available. IBA ProteusONE is a compact superconducting synchrocyclotron proton system, delivering dose to each spot with a pulsed proton beam using a *“blind golfer” algorithm* which repaints each energy layer in three bursts to improve dose accuracy. It does not support automatic gating yet, and the system is prone to random pauses during beam delivery. Interplay effect in such a situation creates concerns regarding real dose delivered to moving targets.

**Methods:** We used the CIRS Dynamic Thorax Phantom moving sup-inf from 0 to 15mm in steps of 2.5mm with a period of 4 seconds. The dose calculation was carried out on a helical CT with 1mm slice thickness of a 3cm diameter moving target in lung equivalent material using the clinically commissioned Mote Carlo dose algorithm in RayStation 10B. A Semiflex ionisation chamber was used to measure the delivered dose: 10 repetitions were carried out for each magnitude of motion to account for a different interplay between target position and beam start.

**Results:** We calculated the dose degradation by comparing the dose delivered to static versus moving target and showed correlation between the amount of volumetric repainting and interplay effects, whilst using the IBA ProteusONE system.

### P 094 - A feasibility study of beam angle optimization in carbon ion radiotherapy for liver cancer

#### Changhwan Kim^1^, Chae-Seon Hong^1^, Seyjoon Park^2^, Min Cheol Han^1^, Jihun Kim^1^, Hojin Kim^1^, Dong Wook Kim^1^, Jin Sil Sung^1^, Jin Sung Kim^1^

##### *^1^Yonsei Cancer Center- Yonsei University College of Medicine, Radiation Oncology, Seodaemun-gu- Seoul, Korea Republic of*

*^2^Yonsei Cancer Center, Radiation Oncology, Seodaemun-gu- Seoul, Korea Republic of*

To investigate beam angle arrangement that can maintain plan robustness in carbon ion radiotherapy for liver cancer, we proposed the beam angle optimization method which considers various factors related to range uncertainties at the same time. In this study, three different factors which can arise range uncertainties were considered; the position uncertainty from set-up, the motional uncertainty from respiration, and density uncertainty from OAR-weighted radiological pathlength. Then, the difference with the nominal beam angle arrangement was compared, in terms of plan robustness. Plan robustness was evaluated by calculating the perturbed dose through shifting plan isocenter with 3 mm set-up uncertainties in six cardinal directions, and scaling CT density with ±3.5% uncertainties. Moreover, the maximum inhale and exhale phases of 4DCT were employed to investigate motional uncertainty. To validate the proposed method, a comparative study was performed with four patients' data. Small dose variations in both target and OARs were obtained in the plans with optimal beam angle, compared to the plans with nominal beam angle. These results showed that the proposed method can provide optimal beam angle arrangement that can preserve plan robustness in carbon ion radiotherapy for liver cancer. Future work will be towards further validation with more patients' data.

### P 095 - Robust planning after optimal beam angle selection for carbon ion radiotherapy planning for locally advanced pancreatic cancer

#### Su Chul Han^1^, Hojin Kim^1^, Seyjoon Park^2^, Chae-Seon Hong^1^, Min Cheol Han^1^, Jihun Kim^1^, Dong Wook Kim^1^, Ik Jae Lee^1^, Jin Sung Kim^1^

##### *^1^Yonsei University College of Medicine, Dept. of Radiation Oncology, Seoul, Korea Republic of*

*^2^Yonsei Cancer Center, Dept. of Radiation Oncology, Seoul, Korea Republic of*

**Objective**: Robust planning is an ultimate objective to attain in carbon ion radiotherapy (CIRT). This work proposes a new CIRT robust optimizing scheme for locally advanced pancreatic cancer (LAPC) by regulating elements associated with uncertainties. It first chose optimal angles considering uncertainties, followed by robust plan optimization and evaluation for validation.

**Methods**: This study defined three uncertainties that can affect the quality of plan robustness: range, set-up (positional inaccuracy), and respiratory motion. While varying the beam angles in posterior direction, absolute values of water-equivalent thickness (WET) was calculated for quantifying range uncertainty. Also, the WET variations were investigated with 4D CT, and translating the average intensity projection (AIP) of 4D CT images that simulate the uncertainties from set-up and respiratory motions. After beam angle selection, robust optimization considering three categories was conducted in RayStation (RaySearch Laboratories^TM^). The proposed plan with 2 selected fields was compared against the conventional 2-port (135° and 225°) and 4-box plans by quantifying passing rate on 12 scenarios under robust evaluation.

**Results**: The two angles, 165° and 255°, were chosen for the optimal 2-port plan. Compared against the conventional 2-port plan, the proposed had greater passing rate in target volume, stomach, duodenum and bowel in robust evaluation. It also resulted in greater passing rate for bowel than 4-box plot, while yielding slightly worse passing rate for stomach without compromising the target volume.

**Conclusion :** Beam angle optimization considering the uncertainties help enhance plan robustness in CIRT for LAPC.

### P 096 - Upright position for abdominal proton therapy: evaluation study with a digital anthropomorphic phantom

#### Wioletta Kozlowska^1^, William Larsen^1,2^, Jonathan Farr^1^

##### *^1^AVO-ADAM, Clinical Office, Meyrin, Switzerland*

*^2^École Polytechnique Fédérale de Lausanne, Micoengineering, Lausanne, Switzerland*

**Introduction:** Upright imaging and treatment provide opportunities to reduce system cost and improve treatment quality for certain indications. This project aimed to evaluate the feasibility of upright abdominal proton therapy (PT).

**Methods:** Upgrading PT techniques requires extensive modelling studies. In this project, patient models, based on XCAT2 [1] digital anthropomorphic phantoms (with various weights, heights, sex, and age), were used for upright Treatment Plan (TP) development. Using Finite Element Method (FEM) simulations, a gravity force was applied to four phantoms, creating deformations and translations of abdominal organs, due to postural changes. Subsequently, modified phantoms were used to prepare proton TPs for liver and kidney tumours.

**Results:** Initial FEM simulations showed an average displacement of selected abdominal organs in phantoms comparable with the available literature [2]. For example, the maximum deformation visible for a liver was 29.1 mm, and the average kidney displacement was 10.5 mm (Table 1). Using modified seated phantoms, developed TPs for abdominal organ tumours (Figure 1) resulted in dose-volume histogram metrics for the upright position comparable to those obtained for the supine position.

**Conclusions:** Anthropomorphic phantoms can be valuable in TP exercises to evaluate innovative approaches like an upright position for PT. Our results showed no decrease in TP quality for the upright position. A future study will evaluate the potential benefit from motion reduction. To confirm phantom modelling, we anticipate benchmarking predicted displacements and deformations with a currently ongoing study using Ultrasonography and Computer Tomography scans with volunteers. [1] https://otc.duke.edu/technologies/4d-extended-cardiac-torso-xcat-phantom-version-2-0/. [2] Reif et al. Int.J.Rad.Onc.Biol.Phys., 45/2/1999.

### P 097 - GSM2 (generalized stochastic microdosimetric model): a new model for RBE prediction

#### Francesco Cordoni^1,2^, Marta Missiaggia^2,3^, Enrico Pierobon^2,3^, Giorgio Cartechini^2,3^, Francesco Tommasino^2,3^, Emanuele Scifoni^2^, Chiara La Tessa^2,3^

##### *^1^University of Trento, Civil Environment and Mechanical Engineering, Trento, Italy*

*^2^INFN-TIFPA, Physics, Trento, Italy*

*^3^University of Trento, Physics, Trento, Italy*

The assumption of a generic and spatially constant Relative Biological Effectiveness (RBE) of 1.1 in proton therapy disregards experimental evidence that RBE varies with beam physical characteristics, physiological and biological factors, and clinical endpoints. An inaccurate RBE can translate into unexpected dosage of both the tumor and normal tissue, decreasing tumor control and increasing treatment-related toxicities. To provide a more accurate prediction of RBE, we have developed the generalized stochastic microdosimetry model (GSM^2^) (Figure 1). RBE correlates with radiation quality, which is determined by particle species and energy and is modified by electromagnetic and nuclear interactions. Current RBE models describe radiation quality either with the dose-average linear energy transfer (LET_d_), or with the equivalent microdosimetric quantity (y_d_)_. _Microdosimetry measures the energy deposited by particles in a micrometer size volume, and thus provides a higher resolution description of the radiation field quality seen by a cell nucleus compared to standard LET_d_. However, both the LET_d_ and y_d_ are average values, and for this reason cannot take into account stochastic variations of energy deposition that occurs at the cell-size level (Figure 2). GSM^2 ^describes the radiation quality using the entire microdosimetric spectrum. In the present study, we characterized the radiation quality generated in- and out-of-field by therapeutic protons in water using microdosimetry, and used GSM^2^ to calculate the RBE. The findings demonstrate that radiation quality changes reflect on the RBE both in- and out-of-field, causing deviations from the reference value of 1.1, and challenging the validity of this approximation.

### P 098 - NTCP model for acute radiation dermatitis in patients with head and neck cancer treated with CIRT

#### Yang Li^1^, Makoto Sakai^1^, Anna Tsunoda^2^, Nobuteru Kubo^1^, Yoko Kitada^1^, Yoshiki Kubota^1^, Akihiko Matsumura^1^, Yuan Zhou^2^, Tatsuya Ohno^1^

##### *^1^Gunma University Heavy Ion Medical Center, Radiation Oncology, Maebashi, Japan*

*^2^Gunma University Graduate School of Medicine, Radiation Oncology, Maebashi, Japan*

**Purpose:** Acute radiation dermatitis (ARD) is common in head and neck cancer (HNC) receiving radiotherapy. This study aimed to construct a normal tissue complication probability (NTCP) model for ARD in HNC patients treated with carbon ion radiotherapy (CIRT).

**Materials and methods:** A cohort of 187 patients were retrospectively analyzed, and the endpoint was ≥grade 2 ARD. The possible prognostic factors associated with ARD were investigated and used as variables for construction of multivariable logistic models. The mean area under the receiver operating characteristic curve (AUC) in the internal cross-validation and Nagelkerke's *R*^2^ were calculated to evaluate the model performance. Calibration curves were created for probability calibration.

**Results:** 80 (42.8%) patients experienced grade 2-3 ARD. Tumor volume, planning target volume to the skin, radiation technique, and all dose–surface parameters were significantly correlated with ARD (*P* < 0.05). The most significant prognostic predictors were S_35 Gy (relative biological effectiveness, RBE)_ and S_15 Gy_ [absolute surface area receiving RBE-weighted dose of 35 Gy (RBE) or physical dose of 15 Gy]. The internal cross-validation-based AUC was 0.78 for both models (Fig.1). The model performances were similar between those with biological and physical dose–surface parameters. However, compared with univariate models, the performances of multivariate models were not improved.

**Conclusions:** NTCP models for ARD were successfully developed in patients with HNC receiving CIRT. The model may help provide the individualized CIRT treatment. Moreover, using biological dose for model construction seems reliable. But further validation study is needed.

### P 099 - Optimal use of limited proton resources for liver cancer patients

#### Louise Marc^1^, Jan Unkelbach^1^

##### ^1^University Hospital Zurich, Department of Radiation Oncology, Zurich, Switzerland

**Objective**: Liver cancer patients may benefit from proton therapy through reduction of mean dose to normal liver (NL=liver-GTV). However, proton therapy is a limited resource and may not be available for all patients. We investigate how to optimally use a limited number of proton fractions in combined proton-photon liver SBRT treatments (CPPT).

**Methods**: Proton and photon plans were created for five liver cancer patients, delivering 10 Gy per fraction to the PTV (Figure 1). In CPPT, limited proton fractions may be optimally exploited by increasing the proton dose (i.e. upscaling the proton and downscaling the photon plan). To determine the proton and photon fraction dose, we minimize the cumulative mean BED3 to the NL while delivering a cumulative BED10 of 100 Gy to the PTV (corresponding to 5x10 Gy). The resulting CPPT balances the benefits of fractionation in the NL versus exploiting the superior proton dose distributions.

**Results***:* For a given number of proton fractions, larger reductions in mean NL dose can be achieved by delivering variable doses in proton and photon fractions, compared to a constant fraction dose of 10 Gy (Figure 2). Fulfilling a mean NL dose constraint of 16 Gy in 5 fractions for all 5 patients requires 4/0/1/4/0 proton fractions for patients 1/2/3/4/5. Instead, for a constant fraction dose of 10 Gy, 5/0/3/5/0 proton fractions are needed.

**Conclusion**: Limited proton resources can be optimally exploited via CPPT by increasing the PTV dose in proton fractions and allocating them to patients with the highest mean NL dose.

### P 100 - Characterisation of the effect of Co-Cr, Ti and PT metal implants in protontherapy

#### Juan Antonio Vera Sánchez^1^, Juan Maria Perez Moreno^1^, Juan Castro^1^, Fernando Cerron Campoo^1^, Marta Montero Feijoo^1^, Morena Sallabanda Hajro^1^, Carme Ares^1^, Ignacio Azinovic^1^, Raymond Miralbell^1^, Alejandro Mazal^1^

##### ^1^Centro de Protonterapia Quironsalud, Medical Physics, Pozuelo- Madrid, Spain

Proton beams are sensitive to the presence in their path of metallic devices implanted in tissues. While traditionnaly fiducials (e.g.Ta) and screws (e.g.Ti) were found in calculations since long time ago, some other metals can appear such as Au, Pt and Co-Cr. In this work we present data on (a) measurements of the effect of those devices on the range of a clinical proton beam of 230 MeV, with planar detectors (chromic films, scintillators) and ion chambers; (b) images acquired with a double-energy CT with extended Hounsfield numbers and artifact reduction algorithms; (c) treatment planning with MonteCarlo approaches; (d) measurement of quality assurance and (e) clinical cases treated with this approach (Fig.1).

### P 101 - Proton minibeam radiation therapy for treating brain metastases: a treatment plan evaluation

#### Ramon Ortiz^1^, Ludovic De Marzi^2^, Yolanda Prezado^1^

##### *^1^Institut Curie Recherche, U1021-UMR3347, Orsay, France*

*^2^Institut Curie, Centre de Protonthérapie d'Orsay, Orsay, France*

Proton minibeam radiation therapy (pMBRT) is a new radiotherapy approach (Prezado, 2013) that has shown a significant increase of the therapeutic window in glioma-bearing rats, as compared to conventional proton therapy (Prezado, 2018). The treatment of brain metastasis situated close to critical structures seems to be a suitable candidate to benefit from this technique. In this study, the potential of pMBRT was evaluated by comparison with stereotactic radiosurgery (SRS) treatments, a technique currently used in clinics for this type of indication (Spetch, 2016). Two clinical cases, initially treated with SRS, were selected for this study. pMBRT treatments consisted of one fraction and 1-2 fields, as proposed for phase I/II clinical trials. Three criteria were used to compare SRS and pMBRT treatments: (i) the tumour coverage, (ii) the dose to organs-at-risk, and (iii) the possible adverse effects in the normal brain by computing the normalized total dose at 2 Gy per fraction. Dose calculations were computed by means of Monte Carlo simulations. pMBRT treatments can provide a similar or superior tumour coverage as compared to SRS. This approach also reduces the biologically effective dose (BED) to critical structures by 44-100% and meets the dose constraints regarding the maximum dose tolerated by healthy brain. These results were obtained using one fraction and 1-2 fields only, which may reduce the possible uncertainties between sessions and the total treatment duration. Therefore, pMBRT seems to be a promising alternative for treating brain metastases. These results can guide the design of the first pMBRT clinical trials.

### P 102 - Clinical proton planning processes currently in use for the pragmatic RADCOMP consortium trial for patients with non-metastatic breast cancer

#### Mark Pankuch^1^, Basit Athar^2^, Chin-Cheng Chen^3^, Yong Chen^4^, Kuan Ling Chen^5^, Nicolas Depauw^6^, Jennifer Dorand^7^, Lei Dong^8^, Samantha Hedrick^9^, Katja Langen^10^, Peng Wang^11^, Tony Wong^12^, LingShu Yin^13^, Omar Zeidan^14^, Hien Lu^8^

##### *^1^Northwestern Medicine Chicago Proton Center, Medical Physics, Warrenville, USA*

*^2^McLaren Proton Therapy Center, Medical Physics, Flint, USA*

*^3^New York Proton Center, Medical Physics, New York, USA*

*^4^University of Oklahoma Health Science Center, Radiation Oncology, Oklahoma, USA*

*^5^Willis-Knighton Cancer Center, Radiation Oncology, Shreveport, USA*

*^6^Massachusetts General Hospital, Radiation Oncology, Boston, USA*

*^7^ProCure Proton Therapy Center, Medical Physics, Somerset, USA*

*^8^University of Pennsylvania, Radiation Oncology, Philadelphia, USA*

*^9^Provision Center for Proton Therapy, Medical Physics, Knoxville, USA*

*^10^Emory Proton Therapy Center, Radiation Oncology, Atlanta, USA*

*^11^Inova Health System, Medical Physics, Fairfax, USA*

*^12^SCCA Proton Therapy Center, Medical Physics, Seattle, USA*

*^13^Johns Hopkins University, Radiation Oncology, Washington DC, USA*

*^14^Orlando Health Cancer Institute, Radiation Oncology, Orlando, USA*

**Purpose**: The RADCOMP consortium trial is a randomized photon vs. proton study for patients with non-metastatic breast cancer. The trial is constructed to be pragmatic in nature and therefore does not provide detailed guidelines on the proton planning process. In this work, the proton planning processes for 14 centers that enroll patients in this study are collected to review the range of optimization methods and robustness criteria that this community is using clinically.

**Methodology**: The physics committee of the RADCOMP study created and distributed a survey to acquire information specifically on the enrolling center's proton planning processes. This information is collected with the intention to share best practices among the contributing centers.

**Results**: For standard breast planning, 57% use SFO optimization, 14% use MFO and 29% use both. For patients with breast expanders in place, 86% use MFO methods. 69% of center reported using robust optimization when planning with SFO, and 79% when planning with MFO methods. 100% of centers report using robust evaluations to evaluate plan robustness. For robust evaluation, 86% of centers use a range error of 3.5%, and 21%, 7% and 71% use a positional offset errors of 3mm, 4mm, and 5mm respectively. 79% of centers evaluate each of the RADCOMP CTV's independently. 50% perform robust evaluation on OARs.

**Conclusions**: Although the RADCOMP trial is designed to collect patient outcomes in a pragmatic nature, the results of this survey demonstrate that the 14 responding centers use similar planning methods and quality indices when developing proton plans.

### P 103 - Feasibility study of eye tracking procedures for ocular proton therapy: A comparison of different open-source pupil detection methods

#### Andrea Pella^1^, Matteo Rossi^2^, Rosalinda Ricotti^1^, Maria Rosaria Fiore^3^, Gabriele Belotti^2^, Luca Antonioli^1^, Giuseppe Magro^4^, Ester Orlandi^5^, Mario Ciocca^4^, Guido Baroni^1,2^

##### *^1^National Center of Oncological Hadrontherapy CNAO, Bioengineering Unit- Clinical Department, Pavia, Italy*

*^2^Politecnico di Milano University, Department of Electronics- Information and Bioengineering, Milan, Italy*

*^3^National Center of Oncological Hadrontherapy CNAO, Radiotherapy Unit- Clinical Department, Pavia, Italy*

*^4^National Center of Oncological Hadrontherapy CNAO, Medical Physics Unit- Clinical Department, Pavia, Italy*

*^5^National Center of Oncological Hadrontherapy CNAO, Clinical Department, Pavia, Italy*

During ocular proton therapy (OPT) at CNAO pupil motion monitoring is performed in real-time with two stereo-cameras mounted on a custom eye tracking system (ETS) [1]. Throughout treatment, gaze stability is qualitatively evaluated by comparing pupil position against a reference contour segmented manually by operators. Aim of this study is to preliminarily evaluate the performance of different algorithms for automatic pupil extraction within the framework of our clinical procedures. We selected three open-source algorithms: DeepVog [2], YOLO [3] and PuReST [4]. DeepVog is a fully convolutional neural network based on a U-NET architecture; YOLO method employs a hybrid model based on Inception V4, PuReST is an edge-based pupil detection method. Four volunteers were asked to sit on the treatment chair, and the ETS was aligned in different clinical-like orientations. The only requirement was to maintain the position as steadily as possible and to look at the fixation LED. We recorded short videos of 10 seconds circa at 25 Hz. In a total of 560 video frames randomly selected, pupils were manually contoured. Differences between manual (reference) and automatic pupil contour identification has been quantified. All the methods under investigation demonstrated noticeable performances. DeepVog, YOLO and PuReST algorithms returned median and interquartile ranges (IQR) of Dice coefficients of: 0.87(0.11), 0.95(0.04) and 0.92(0.06), respectively. Automatic procedures for eye tracking during OPT could provide a significant aid during clinical operations. A larger and heterogenous dataset for training and testing methods based on machine learning is mandatory to fully exploit their performances. [1]doi:10.1118/1.4915921. [2]doi:10.1016/j.jneumeth.2019.05.016. [3]doi:10.1145/3314111.3319914. [4]doi:10.1145/3204493.3204578

### P 104 - Improved predictions of the relative biological effectiveness of ions based on the local effect model

#### Tabea Pfuhl^1^, Thomas Friedrich^1^, Michael Scholz^1^

##### ^1^GSI Helmholtzzentrum für Schwerionenforschung, Biophysics, Darmstadt, Germany

The local effect model (LEM) is a radiobiological model for the prediction of the increased biological effect after exposure to ion radiation, which is quantified by the relative biological effectiveness (RBE). Comparisons of model predictions to measurement data revealed an underestimation of RBE for high-energetic carbon ions for the current model version (LEM IV). In the LEM IV, the effect after ion radiation is determined from measurements with photon radiation, assuming that a local dose unit of either radiation leads to the same effect, i.e. the same number of double-strand-breaks in the DNA. Thus, the underlying composition of secondary electrons, which are responsible for the major part of the biological damage, is neglected. However, it varies with the primary radiation species and also within ion tracks with the radial distance to the track center. Now, a new model version (LEM V) explicitly includes the different biological effects resulting from different secondary electron spectra in the effect prediction. By combining an amorphous track structure approach with details of the secondary electron spectrum, the improvements lead to an increased model accuracy, especially in the high-energy regime. Furthermore, the prediction of the minimum RBE at large ion doses shows a clearly improved agreement with measurement data compared to previous LEM versions. Additionally, the model consistency was increased through a more detailed description of the double-strand-break induction and distribution in an ion track.

### P 105 - ConformalFlash Proton therapy planning with RayStation

#### Arnaud Pin^1^, Erik Traneus^2^, Rasmus Nilsson^2^, Claes Fälth^2^, Rudi Labarbe^1^, Lucian Hotoiu^1^, François Vander Stappen^1^

##### *^1^Ion Beam Applications IBA SA, Research, Louvain-La-Neuve, Belgium*

*^2^RaySearch Laboratories AB, Research & Development, Stockholm, Sweden*

FLASH proton therapy (FLASH-PT) aims to use ultra-high dose rates (>40 Gy/s) to induce a normal tissue sparing effect whilst maintaining the anti-tumour effectiveness of conventional dose rates. Consequently, dose rates, beam delivery, and fractionation schemes for FLASH-PT differ from standard radiotherapy. To fulfil the FLASH time constraints and achieve high dose-rates in proton PBS irradiation, the ConfromalFLASH^(R)^ technique consists in scanning the pencil-beam in a single energy layer delivery, whilst ensuring the target coverage within the generated Spread-Out-Bragg-Peak (SOBP). This technique requires passing the proton pencil-beam at the highest cyclotron energy through a range shifter adjusting the beam maximum range and through a conformal energy modulator (CEM) that ensure the control of the distal and proximal conformality and modulation. The RayStation TPS allows the creation of ConformalFlash proton plans. The plan creation process consists in the optimization of an initial IMPT plan. Then the SOBP covering the target volume is obtained in RayStation by computing the CEM shape that reproduces the energy fluence map from the initial IMPT plan. The range-shifter thickness is maximized and the aperture shape is selected. The Monte Carlo dose computation in RayStation includes the CEM, the Range-shifter and the aperture in the transport. The CEM patient specific device is exported to be 3D-printed.

Integrating MIROpt TPS (http://openmiropt.org) algorithms in RayStation script, the spot scanning trajectory is computed, and the spot delivery timing is simulated to compute dose rate metrics for each voxel.

### P 106 - Accurate uncertainty analysis demonstrates consistency of the Dutch robustness evaluation protocol for head and neck cancer IMPT

#### Jesus Rojo-Santiago^1,2^, Steven J.M. Habraken^1,2^, Erik Korevaar^3^, Danny Lathouwers^4^, Zoltán Perkó^4^, Stefan Both^3^, Mischa S. Hoogeman^1,2^

##### *^1^Erasmus MC Cancer Institute, Radiotherapy, Rotterdam, Netherlands*

*^2^HollandPTC, Medical Physics & Informatics, Delft, Netherlands*

*^3^University Medical Center Groningen, Radiation Oncology, Groningen, Netherlands*

*^4^Delft University of Technology, Radiation Science and Technology, Delft, Netherlands*

**Purpose/Objective:** To assess inter-patient variation in CTV dose with the Dutch robustness evaluation protocol for head-and-neck (H&N) patients, treated in two centers with proton beam hardware from different vendors.

**Material and methods:** Clinical treatment plans for 80 patients were included, divided in subcohorts of 20 plans with 3mm/3% and of 20 plans with 5mm/3% geometrical (GRS) and range robustness settings per center. In both centers, dose was prescribed to the voxel-wise minimum dose of 28 evaluation scenarios: VWmin-D_98%,CTV_≥94%D_pres,Center1 _and VWmin-D_98%,CTV_≥95%D_pres,Center2_. Polynomial Chaos Expansion was applied to generate a plan-specific analytical model of voxel doses, enabling the evaluation of 100,000 realistic fractionated treatment scenarios for each plan. Geometrical systematic (Σ) and random (σ) errors were sampled from Gaussian distributions with errors (1SD) of (i) Σ=0.94mm and σ=1.14mm and (ii) Σ=1.65mm and σ=1.25mm, consistent with a 3mm and 5mm margin based on van Herk's recipe: M=2.5Σ+0.7σ, respectively. For each center and each GRS, cumulative population D_98%,CTVs _distributions for the elective and primary CTVs were derived.

**Results:** At 90% population coverage, treatment plans with GRS=3mm realized a population median D_98%,CTVs_ of 68.7 (5th/95th percentiles: 68.5/68.9) GyRBE and 53.4 (53.3/53.5) GyRBE for center 1 and 69.0 (68.8/69.2) GyRBE and 53.8 (53.6/54.0) GyRBE for center 2. For a GRS=5mm, a population median D_98%,CTVs_ of 68.9 (68.7/69.2) GyRBE and 53.5 (53.3/53.7) GyRBE for center 1 and 69.0 (68.6/69.3) GyRBE and 53.9 (53.6/54.1) GyRBE for center 2 were found respectively.

**Conclusion:** The robustness evaluation protocol leads to consistent results between centers. Furthermore, with GRS=3mm, it ensures adequate robustness of planned CTV dose for H&N patients.

### P 107 - Dosimetric evaluation of proton therapy between multi-field optimization (MFO) and single-field optimization (SFO) technique for esophagus abutting lung cancer

#### Heesoon Sheen^1^, Woojin Lee^2^, Hongryull Pyo^2^, Kwanghyun Jo^2^

##### *^1^Samsung Advanced Institute for Health Sciences & Technology/ Sungkyunkwan University, Department of Health Sciences and Technology, Seoul, Korea Republic of*

*^2^Samsung Medical Center, Department of Radiation Oncology, Seoul, Korea Republic of*

**Purpose**: The purpose of this study is to show the superiority of multi-field optimization (MFO) than single-field optimization (SFO) by comparing dose distribution of scanning proton therapy for esophagus abutting lung cancer patients.

**Materials and methods**: Thirty-three patients with lung cancer located adjacent to the esophagus were studied. The proton therapy (PT) plans of MFO and SFO were calculated by Monte Carlo algorithm along with robust optimization tool in RayStation v6 (Raysearch Lab, Stockholm, Sweden). The Conformity index (CI), homogeneity index (HI) and other indices from dose histogram volume (DVH) of PTV and three OARs (lung, heart, spinal cord, and esophagus) were calculated, and their results were compared using Wilcoxon signed-rank test in MedCal 20.015 (MedCalc Software Ltd, Ostend, Belgium).

**Results**: The volume of PTV (planning target volume) was distributed from 79.93 cm^3^ to 1332.33 cm^3^ and its median was 432.55 cm^3^ and mean was 601.70 cm^3^. Two indices, CI_RTOG, _and HI were not significantly different (relative % difference > 3% and p-value <0.05) between MFO and SFO (table 1). In all OARs, MFO plan was significantly lower than SFO plan (from -7.37% to -14.12%) except for the D_max _of the spinal cord, which was 5.57% higher than that of SFO. All results were presented in Table 2.

**Conclusions**: Compared to SFO, MFO offered the similar robustness in the targets, and improved dose sparing in all critical organs except the spinal cord.

### P 108 - Dosimetric comparison of robust angles for prostate cancer in carbon-ion radiation therapy

#### Han-Back Shin^1^, Han Min Cheol^1^, Hong Chae-Seon^1^, Park Seyjoon^2^, Koom Woong Sub^1^, Kim Jin-Sung^1^

##### *^1^Yonsei Cancer Center- Yonsei University College of Medicine, Radiation Oncology, Seoul, Korea Republic of*

*^2^Yonsei Cancer Center, Radiation Oncology, Seoul, Korea Republic of*

In this study, planning study of carbon-ion radiation therapy (CIRT) for prostate cancer was performed with different beam angle configurations, and various dosimetric parameters obtained from its relative biological effectiveness (RBE) dose and linear energy transfer (LET) distributions were compared. Ten patients treated for prostate cancer at our institution were retrospectively included in this study. The prescribed dose and dose constraints for CIRT were adopted from National Institute of Radiological Sciences protocol 9904-4 and 1002. To evaluate the dosimetric effect of beam angle selections, total five plans were considered for each patient with different angle pairs as shown in Fig. 1. Robust optimization method was adopted considering setup and range uncertainties. For the comparison of dosimetric parameters, RBE dose and LET distribution were calculated and compared each other. In our results, there was no significant difference of RBE doses for target and OARs according to beam angle selection, but regarding LET, it was confirmed that oblique beam fields had accrued dosimetric benefit compared to conventional fields (Fig. 2); according to the beam angle selection, the LET to D95 of rectum was reduced to approximately 40% of that of the conventional beam. The detailed comparison results regarding RBE and LET-related parameters considering perturbed scenarios will be presented at PTCOG60 in June 2022.

### P 109 - Evaluation of an automated planning IMPT workflow to create IMPT comparative plans for insurance approval

#### Alexander Stanforth^1^, William LePain^1^, Mosa Pasha^1^, Jonathan Wolf^1^, Mark McDonald^1^, Jun Zhou^1^, Roelf Slopsema^1^

##### ^1^Emory University, Radiation Oncology, Atlanta, USA

**Introduction:** In the US, insurance authorization for proton therapy often requires comparative treatment planning. To expedite comparative planning in H&N cancer, we developed an automated planning (AP) process for intensity-modulated proton therapy (IMPT) comparative plans. We evaluated the quality of AP IMPT plans compared to manually generated plans (MP).

**Materials and methods**: We evaluated 29 H&N patients receiving bilateral neck radiation with prior manually-generated IMPT plans. Within the treatment planning system, the scripting module was used to generate AP plans using our standard 5-field technique, which were compared to the MPs based on dose statistics to CTVs and OARs, and plan robustness. MPs and APs were made using both analytical pencil beam algorithm and Monte Carlo dose calculation algorithm to evaluate the differences between the algorithms.

**Results:** AP and MP dose statistics were comparable for CTV coverage (APs average difference 0.5% ± 1.46% higher coverage). APs showed on average lower OAR dose by 2.85 ± 5.812 Gy. APs on average showed 98% coverage on more robust scenarios (47.7% vs 29.1%, p < 0.001), and achieved a higher worst-case V95% for each CTV (99.2% vs 96.2%, p < 0.001). APs using a pencil beam algorithm on average had a lower OAR dose compared to APs using a Monte Carlo algorithm by 0.69 ± 2.91 Gy.

**Discussion**: The automated planning procedure produced plans which were of the same quality of MPs.

### P 110 - Investigation of an efficient plan conversion method between two types of proton systems

#### Zhong Su^1^, Wen Hsi^2^

##### *^1^University of Arkansas for Medical Sciences, Radiation Oncology, Little Rock, USA*

*^2^University if Florida Proton Therapy Institute, Radiation Oncology, Jacksonville, USA*

**Purpose**: To increase machine switching efficiency by modeling a Proteus-1 proton machine with built-in range shifter (P1RS) for direct recalculation of treatment plans between P1 and universal nozzle of Proteus-plus (P+US) systems

**Materials and method**: Re-planning is usually required if a patient needs to be moved from P+ to P1 machines due to their different spot sizes. This usually requires re-planning of the patient and creates significant time delay when one machine is down. We are proposing commissioning a P1 machine model with built-in range shifter (P1RS) with fixed snout position at 45cm up stream. The spot size and PDD of the P1RS are compared to those of the P+US. The P+ patient plans are recalculated using P1RS machine directly with both pencil beam and Monte Carlo dose engines.

**Results**: The P1RS machine model has similar spot size as those of P+US (Figure 1). There are noticeable differences between the PDDs of the two models. For patient plan with field size less than 20cm by 20cm, preliminary investigation of direct conversion of treatment plans between the two machine models indicating it is feasible.

**Summary**: P1 model with a built-in range shifter can simulate a P+US system. Direct recalculation of treatment plan between the two systems is feasible and could increase machine switching capabilities and efficiency.

### P 111 - Deep learning Proton dose verification using a deep learning approach for palm-sized dual-head PET solution

#### Atiq Ur Rahman^1^, Mythra Varun Nemallapudi^2^, Shih-Chang Lee^2^

##### *^1^National Central University/ Academia Sinica, Physics, Taipie, Taiwan*

*^2^Institute of Physics-Academia Sinica, Physics, Taipie, Taiwan*

In-vivo dose monitoring in proton therapy is currently being investigated because it is crucial for the optimization of treatment planning. Emission of the positron emitters has a good correlation with dose profiles, but such correlations are nonlinear and hard to estimate under real situations. We have studied deep learning based direct dose mapping approach which takes the raw coincidence data coming from positron-emitting isotopes produced during the proton beam irradiation detected by a small palm-sized detector in the form of 2D coincidence maps. We have simulated the proton doses for varying proton energies, beam sizes, and irradiation positions using a patient CT and obtained a 2D raw data to train our deep learning model. We have used a conditional generative adversarial network (cGAN) to leverage the capacity of the small detector to reconstruct absolute dose profile. The performance of the model has been evaluated using mean relative error (MRE), absolute dose difference, and shift in the Bragg peak (BP) positions. The model can predict the dose with MRE value 1.67 %, absolute dose with uncertainty of 2.5 %, and shift in BP value less than 1%. It turns out that the accuracy is the function of photo-peak coincidences. The model requires approximately 11000 coincidences on average to achieve less than 1% uncertainty in the prediction of BP position. The study is useful to predict 2D dose map in a very short time directly by utilizing raw detector data without undergoing conventional image reconstruction and kernel-based approaches.

### P 112 - Dosimetric comparison of TRiP4D and Syngo treatment planning systems for proton and carbon ion radiotherapy

#### Yinxiangzi Sheng^1^, Chrisitian Graeff^1^, Lennart Volz^1^, Weiwei Wang^2^

##### *^1^GSI Helmholtzzentrum für Schwerionenforschung GmbH, Biophysics, Darmstadt, Germany*

*^2^Shanghai Proton and Heavy Ion Center, Department of Medical Physics, Shanghai, China*

**Purpose:** To benchmark the accuracy of the treatment planning system (TPS) TRiP4D in reproducing doses delivered at Shanghai Proton and Heavy Ion Center (SPHIC), by comparing to the experimentally verified doses computed by the clinically used TPS Syngo.

**Methods:** Proton and carbon base data for TRiP4D were imported from Syngo. Plans were generated in Syngo for a water phantom and were imported into TRiP4D for 3D dose reconstruction. A total of 17 plans were compared, including 7 RBE-weighted dose optimization carbon plans with target dose of 3 to 4.5Gy (RBE). The 3D global gamma-index (2%-2 mm with 10% threshold), 1D dose profiles, and target mean dose were evaluated.

**Results:** The difference in physical dose distribution between TRiP4D and Syngo is negligible. Gamma pass rates (γ-PRs) were > 99.9% for all the plans. The mean target RBE-weighted dose was -1.17%±0.19% (mean±SD) lower for TRiP4D than Syngo. In TRiP4D, the faster analytical ‘low dose approximation' [Krämer 2006] was used, while Syngo used a stochastic implementation [Krämer 2000]. The mean target dose deviation could be reduced to -0.59%±0.07%, and γ-PRs were >97% for all plans with the same biological effect algorithm—however, this increased dose recalculation time by a factor of 150 to 300.

**Conclusion:** The residual difference between TRiP4D and Syngo is small. We will use TRiP4D for the evaluation of doses delivered to moving targets at SPHIC.

### P 113 - Development of an Auto Planning Tool for particle therapy in Raystation using Organ at Risk tiers

#### Tyler Williamson^1^, Thomas Whitaker^1^, Rachel Hunter^1^, Xiaodong Zhang^1^

##### ^1^The University of Texas MD Anderson Cancer Center, Radiation Physics, Houston, USA

We developed an auto planning tool (APT) that does not rely on previous plans nor doses, and instead uses an organ at risk (OAR) tier. A dose constraint and tier is assigned to each OAR, to indicate the permissible target coverage loss relative to that OAR/constraint. The APT will use the OAR tier and scripting in Raystation to create objectives, optimize the plan, and iteratively adjust objectives. Ten recent bilateral base of tongue patients were selected. A three field setup (gantry-couch = 300-20/ 60-340/ 180-0) and robust optimization (2mm shift +/- 2.5% range uncertainty) were used for planning. Two clinical beamlines were used to represent a dosimetric challenge (large spots ∼7-14mm vs small spots ∼4-10mm). Plans were optimized independently and resulted in different ending objectives. No manual optimization adjustments were made. Results: (Contralateral_Parotid_MeanDose = 17.5Gy vs 10.1Gy, Ipsilateral_Parotid_MeanDose = 31.8Gy vs 18.9Gy, Larynx_MeanDose = 40.2Gy vs 28.7Gy, Oral_Cavity_MeanDose = 20.6Gy vs 18Gy) (CTV_High_D95 = 70.2Gy vs 70.4Gy, CTV_Mid1_D95 = 67.5Gy vs 67.5Gy, CTV_Mid2_D95 = 63.4Gy vs 63.1Gy,

CTV_Low_D95 = 57Gy vs 57.3Gy). OAR tier APT via Raystation scripting was able to create reasonable plans for both beamlines without the need for a plan database nor dose prediction tool. Additionally, when utilizing a smaller spot beamline, the APT tool further reduced OAR doses while achieving similar coverage. Next step is comparing the OAR tier APT against multi-institutional clinical patients. Future direction and benefits can include the Physician adjusting OAR tiers for a patient based on clinical indications and/or adding new OARs.

### P 114 - A mathematical method to adjust MLC leaf end position for accurate dose calculation in carbon ion therapy treatment planning system

#### Yan Zhang^1^, Ye Yan-Cheng^1^, Jia-Ming Wu^1,2^

##### *^1^Gansu Wuwei Academy of Medical Sciences- Gansu Wuwei Tumor Hospital-, Heavy Ion Center of Wuwei Cancer Hospital-, Wuwei City- Gansu province- China., China*

*^2^Department of Medical Physics- Chengde Medical University- Chengde City- Hebei Province- China., Dept. of Medical physics, Hebei, China*

**Introduction:** We present a mathematical method to adjust the leaf end position for dose calculation correction in the carbon ion radiation therapy treatment planning system.

**Methods and materials:** A straggling range algorism of 400 MeV/n carbon ion beam in nine different multi-leaf collimator (MLC) materials was conducted to calculate the dose 50% point to derive the offset corrections in the carbon ion treatment planning system (ciPlan). The visualized light field edge position in the treatment planning system is denoted as X_tang.p_ and MLC position (X_mlc.p_) is defined as the source to leaf end mid-point projection on-axis for monitor unit calculation. The virtual source position of energy at 400 MeV/n and straggling range in MLC at different field sizes were used to calculate the dose 50% position on-axis. On-axis MLC offset (correction) could then be obtained from the position corresponding to 50% of the central axis dose minus the X_mlc.p_ MLC position.

**Results**: The exact MLC position in the carbon ion treatment planning system can be used as an offset to do the correction. The offset correction of pure tungsten is the smallest among the others due to its shortest straggling range of carbon ion beam in MLC. The positions of 50% dose of all MLC materials are always located in between X_tang.p_ and X_mlc.p_ under the largest field of 12 cm by 12 cm.

**Conclusions:** MLC offset should be adjusted carefully at different field sizes in the treatment planning systems especially of its small penumbra characteristic in the carbon ion beam.

### P 115 - A linear formulation of the Proton Arc Therapy Optimization problem solved with Mixed-Integer Programming

#### Sophie Wuyckens^1^, Michael Saint-Guillain^2^, Guillaume Janssens^3^, Edmond Sterpin^1,4^, John Lee^1^, Kevin Souris^1^

##### *^1^UCLouvain, Molecular Imaging- Radiotherapy and Oncology MIRO, Woluwe-Saint-Lambert, Belgium*

*^2^UCLouvain, Institute of Information and Communication Technologies- Electronics and Applied Mathematics ICTM, Louvain-La-Neuve, Belgium*

*^3^Ion Beam Applications SA, Research, Louvain-La-Neuve, Belgium*

*^4^KULeuven, Department of Oncology, Leuven, Belgium*

**Goal**: Similarly to VMAT, Proton Arc Therapy (PAT) is the evolution of proton therapy (IMPT) with a gantry that rotates continuously around the patient while delivering the treatment, forming an arc. Several planning studies have already shown numerous advantages compared to IMPT (better conformality, LET, robustness, …). However, the increased number of beams makes PAT treatment optimization more complex. The delivery time can also drastically rise if not optimized during planning. New algorithms are therefore needed to address the challenges of PAT optimization.

**Methods and materials**: We propose an exact method using a Mixed-Integer Programming (MIP) formulation and an appropriate solver that conducts a complete search of the solution space in order to obtain optimality proofs but also treatment guarantees. The PAT algorithm is tested on a brain case and compared to VMAT, IMPT and SPArcling, an in-house version of SPArc algorithm proposed by Beaumont.

**Results**: For a similar target coverage and the best conformity index obtained, the MIP method was able to spare the brain stem better than the other modalities by achieving 66 % of the dose limit set to 55 Gy versus 77% (SPArcling), 91.5 % (VMAT) and 80% (IMPT). In terms of delivery efficiency, the MIP was constrained to give at most 6 energy upward switching to obtain a fair comparison with the SPArcling algorithm.

**Conclusion**: With a new modality comes new challenges. The PAT treatment planning optimization problem is one of them and the MIP method might become a concrete solution with extensive search.

### P 116 - Towards the complete automated knowledge-based planning pipeline for photon-proton plan comparison in head and neck cancer adaptive radiotherapy

#### Adam Yeo ^1^

##### ^1^Peter MacCallum Cancer Centre, Physical Sciences, Melbourne, Australia

In Australia, a proton-photon comparative planning is required for medication treatment overseas program (MTOP) application and its federal funding needs to be justified by clinical recommendations based on predicted dosimetric assessment. This work aims to demonstrate the complete automated photon-proton comparative planning pipeline using RapidPlan knowledge-based-planning (KBP) (Eclipse v16.1, Varian Medical Systems, Palo Alto, USA). This clinical application of machine-learning in comparative planning is also implemented to perform sensitivity assessment against given anatomy changes in H&N adaptive radiotherapy (ART) scenarios, e.g. weight loss and/or tumour response. Two separate groups of 20 H&N cancer patients (total 40 patients) were included to build both photon and proton RapidPlan models and validate the models, respectively. The validated photon-proton plans were recalculated on its associated re-scan CT, which was due to anatomy change (mainly reduction in neck separation), in order to perform sensitivity analysis via robust evaluation of the two plans against the given anatomy changes. The vast majority of cases (CI95%) shows favourable dosimetry in protons plans over photon plans in terms of adjacent OAR doses and clinical goals as per our departmental protocol. Whereas, dosimetric variation against the given anatomy changes showed proton plans are more sensitive and less robust against the given anatomy changes relative to photon plans. This automated KBP pipeline allows effective and efficient photon-proton comparative planning, which consequently enabled adaptive dosimetric assessment in H&N ART scenarios. This work has potential to play an important role as independent credentialing tools in context of MTOP as well as clinical trials.

### P 117 - Design and evaluation of a new couch insert for proton therapy

#### Yuanshui Zheng^1^, Yajun Jia^1^, Zuofeng Li^1^, Zhiwen Zeng^2^, Wei Wang^3^, Guoping Dong^1^

##### *^1^Guangzhou Concord Cancer Center, Radiation Oncology, Guangzhou, China*

*^2^Clarity, Clarity, Guangzhou, China*

*^3^Clarity, Research, Guangzhou, China*

**Purpose**: The purpose of the study is to design and evaluate a new couch insert for head and neck immobilization during proton beam treatment.

**Methods**: The physicist team and therapist team at our proton center worked together with vendor engineers to design a couch insert (head frame) for proton treatment, with goal for ease of mounting and better tolerance for positional error. A prototype was first manufactured and evaluated for dosimetry with CT scanned images and ease of use for mask mounting with a therapist lying down. Evaluation feedback was incorporated in the second version, which was manufactured and tested in more details including couch load test and robustness analysis by changing head phantom position (shift and rotation) relative to the couch and evaluating dose changes for both target and normal tissues. Comparison was also carried out with another commercially available head frame.

**Results**: A new couch insert was designed and evaluated for clinical use (see figure). Couch load test indicates it is comparable or slightly more rigid than another commercial product, with about 1.7mm couch top sag for a 10 kg weight on the couch head and almost no further sag with time for up to 30 mintutes. Preliminary dosimetric study indicates the couch is relatively tolerant on positioning errors, with more detailed study in progress. The couch insert has been commercialized and used clinically.

**Conclusions**: The new couch insert meets our expectation both mechanically and dosimetrically and are therefore suitable for proton therapy.

### P 118 - Deep learning-based accurate material density map generation using MRI and DECT

#### Chih-Wei Chang^1^, Raanan Marants^2^, Yuan Gao^1^, Matthew Goette^1^, Jeffrey Bradley^1^, Tian Liu^1^, Jun Zhou^1^, Atchar Sudhyadhom^2^, Xiaofeng Yang^1^

##### *^1^Emory University, Radiation Oncology, Atlanta, USA*

*^2^Harvard University, Radiation Oncology, Boston, USA*

**Purpose:** This work explores the feasibility of using physics-informed deep learning (PIDL) to integrate MRI and dual-energy CT (DECT) information to enhance material mass density inference for proton Monte Carlo dose calculation.

**Methods:** We propose a PIDL framework to correlate MRI and DECT to material mass density constrained by a physics model (Figure 1). To demonstrate the benefit of MRI, we compared three models including the empirical model and ResNet-DECT using DECT data and ResNet-MR-DECT trained by DECT and MRI data. Four tissue substitute MRI phantoms were used for deep-learning-based material calibration including adipose, skin, muscle, and cortical bone. The training inputs include T1-weighted Dixon water- and fat-only images, T2-weighted STIR, twin-beam 120 kVp high/low images, 80 keV images, and relative electron density map. The reference mass densities of each phantom were based on relative stopping powers measured by a 200 MeV proton spot.

**Results:** The empirical model, ResNet-DECT, and ResNet-MR-DECT shows mass density errors of: 1.4%±4.0%, 2.9%±2.0%, and 0.4%±3.9% for adipose; 1.0%±0.8%, 1.6%±0.8%, and 0.5%±0.9% for skin; 0.1%±0.9%, -0.6%±1.0%, and 0.0%±0.9% for muscle; -0.7%±3.8%, -0.8%±1.4%, and -0.4%±1.2% for cortical bone. ResNet-MR-DECT shows consistent trend compared to Hounsfield unit (HU) line profile variations of an 80 keV images, while ResNet-DECT results in large deviation from the profiles (Figure 2).

**Conclusion:** The physics-informed ResNet-MR-DECT demonstrated more accurate material mass density conversion using MRI phantoms than ResNet-DECT trained using only DECT data. The patient study shows that ResNet-MR-DECT has the potential to enhance proton treatment uncertainty due to material conversion.

### P 119 - Physics-Informed deep learning DECT Parametric mapping for proton Monte Carlo dose calculation

#### Chih-Wei Chang^1^, Yuan Gao^1^, Tonghe Wang^1^, Yang Lei^1^, Qian Wang^1^, Jeffrey Bradley^1^, Tian Liu^1^, Liyong Lin^1^, Jun Zhou^1^, Xiaofeng Yang^1^

##### ^1^Emory University, Radiation Oncology, Atlanta, USA

**Purpose:** A framework of physics-informed deep learning (PIDL) based dual-energy CT (DECT) parametric mapping was developed to infer material characteristics for proton Monte Carlo dose calculation. This work investigates material conversion and dosimetric accuracy using PIDL models.

**Methods:** A PIDL framework was used to generate material mass density and relative stopping power (RSP) maps from DECT with artificial neural networks (ANN), physics-informed ANN (PANN), and physics-informed ResNet (PRN). The material characteristic maps acquired from empirical models (DECT) and a stoichiometric method using single energy CT (SECT) were compared to the PIDL method. Dose plans were calculated using Monte Carlo. A CIRS anthropomorphic was irradiated at an HN site using anterior multiple-single-energy proton beams ranging from 175 to 189 MeV with 1-MeV incremental energy. A MariXX PT was placed under a proton couch to measure the exit dose from the phantom. Gamma criteria of 3%/3mm were used.

**Results:** Mass density errors of bone by empirical model, ANN, PANN, and PRN are 3.0%, 3.8%, 2.5%, and 0.5%, and RSP errors are 3.2%, 3.5%, 2.5%, and 0.9%. The soft tissue mass density errors of soft tissues of the four models are 2.7%, 2.5%, 1.3%, and 1.1%, and RSP errors of lung are 7.5%, 11.0%%, 10.9%, and 0.7%, respectively. The mean gamma passing rates of clinical plan using SECT, empirical model, and PRN are 97.0%, 94.8%, and 98.5%.

**Conclusion:** The PRN model demonstrated accurate material mass density and RSP conversion, and dosimetry delivery using DECT with an anthropomorphic phantom.

### P 120 - Online adaptation reduces the need for offline replanning due to anatomy changes in head and neck intensity-modulated proton therapy

#### Mislav Bobić^1^, Arthur Lalonde^1^, Konrad Nesteruk^1^, Hoyeon Lee^1^, Bram Gorissen^2^, Alejandro Bertolet^1^, Brian Winey^1^, Antony Lomax^3^, Harald Paganetti^1^

##### *^1^Massachusetts General Hospital and Harvard Medical School, Department of Radiation Oncology, Boston, USA*

*^2^Broad Institute of Harvard and MIT, Stanley Center for Psychiatric Research, Boston, USA*

*^3^Paul Scherrer Institute, Center for Proton Therapy, Villigen, Switzerland*

The goal of this study was to compare daily online adaptation (OA) with offline replanning (RP) for head and neck (H&N) intensity-modulated proton therapy (IMPT). Treatment plans were created for a cohort of eight H&N cancer patients that had required offline replanning due to severe anatomy changes. The anatomy changes were mainly caused by weight loss (median = 5 kg, max =15 kg) and clinical target volume (CTV) shrinkage (median = 15%, max = 30%). To simulate the RP workflow, nominal treatment plans were created on the planning CT (pCT), while replanning was based on a repeat CT (rCT) to meet the same clinical goals as the initial plan. To simulate the OA workflow, plans were created on the pCT and adapted at each fraction using a fast online adaptation framework, employing Monte Carlo dose calculations on cone-beam CTs and deformable image registration for contour propagation. Compared to RP, daily OA maintained or improved the target coverage while reducing the OAR dose. Across all OARs, the median dose was reduced between 2.5% and 34.2%. In terms of target dose, OA reached the clinical goals in 8/8 patients, compared to 5/8 for RP. The median integral dose to healthy tissue was 12% lower for OA than for RP. Both adaptation scenarios yield improved target coverage compared to no adaptation. In conclusion, daily OA can restore the planned dose distributions in H&N patients experiencing severe anatomy changes, reducing the need for offline and manual replanning.

### P 121 - Margin reduction in particle therapy: potential benefits of using radioactive ion beams

#### Olga Sokol^1^, Daria Boscolo^1^, Christian Graeff^1,2^, Micol De Simoni^3,4^, Laura Cella^5^, Giuseppe Palma^6^, Roberto Pacelli^7^, Caterina Oliviero^7^, Katia Parodi^8^, Marco Durante^1,9^

##### *^1^GSI Helmholtz Centre for Heavy Ion Research, Biophysics, Darmstadt, Germany*

*^2^Technische Universität Darmstadt, Fachbereich Elektrotechnik und Informationstechnik, Darmstadt, Germany*

*^3^INFN, Section of Rome 1, Rome, Italy*

*^4^Sapienza- University of Rome, Physics, Rome, Italy*

*^5^National Research Council CNR, Institute of Biostructures and Bioimaging, Napoli, Italy*

*^6^National Research Council CNR, Institute of Nanotechnology, Lecce, Italy*

*^7^Azienda Ospedaliera Universitaria, Federico II, Napoli, Italy*

*^8^Ludwig-Maximilians-Universität München, Fakultät für Physik, Munich, Germany*

*^9^Technische Universität Darmstadt, Fachbereich Physik, Darmstadt, Germany*

Range uncertainty remains one of the main limitations of carbon ion therapy and entails wider margins to the CTV. PET imaging is the beam verification method with most clinical applications, but for ^12^C ion beams its applicability is limited by the low signal/noise ratio and the shift between the dose and activity peaks. Direct use of β^+^-emitting radioactive beams (RIBs) for treatment could increase the signal and its correlation with the dose distribution, allowing margins reduction. The experimental campaign to characterize RIBs and enable their therapeutic application is ongoing at GSI (BARB project; abstract by D. Boscolo). This work aims to estimate the potential clinical benefits of tumor margin reduction granted by treatments with RIBs. We developed a FLUKA beam model for ^11^C for use within the TRiP98 treatment planning system and established the workflow to predict the resulting activity maps. We demonstrate that in ^11^C plans, the range margins can be practically eliminated since the activity fall-off strictly follows the dose fall-off. Following that, we performed robust treatment plan optimization with ^11^C and ^12^C beams for head-and-neck and liver tumors. Reduced margins in ^11^C plans lead to a substantial increase in the number of patients and treatment scenarios where critical structures receive the dose below the allowed maximum (see the figure). The results of the NTCP evaluation, additionally performed to estimate the toxicity for serial and parallel critical organs in the target proximity, will be presented.

### P 122 - Biomedical applications of radioactive ion beams: first results of the BARB project at GSI

#### Daria Boscolo ^1^

##### ^1^GSI Helmholtzzentrum für Schwerionenforschung, Biophysics, Darmstadt, Germany

Heavy ion particle therapy is potentially the most effective and precise radiotherapy technique. However, range uncertainties remain one of its limitations: they jeopardize the benefits of the sharp Bragg peak and force to use wide margins extending in the normal tissue. The use of radioactive ion beams (RIBs) for simultaneous treatment and online range verification using PET could help overcoming this limitation, due to increased signal/noise ratio and alignment of the activity peak with the Brag peak compared to PET imaging obtained when using primary stable ions. In this context, the BARB (Biomedical Applications of Radioactive ion Beams) project was initiated at GSI aiming to assess the technical feasibility and investigate possible advantages of RIBs in preclinical studies. During the first year of experiments, radioactive beams (^10,11^C and ^15^O) were produced by isotopic separation with the fragment separator and transported to the medical vault of GSI. Thanks to the upgrade of the SIS-18 in the FAIR in Darmstadt, it was possible to achieve RIB intensities sufficient to treat a small animal tumor. Beam implantation in plastic phantoms was visualized by two independent imaging setups: a dual-panel PET scanner from the UMCG (Groeningen) and a subset of a high resolution small animal γ-PET detector in development at the LMU (Munich) (see abstracts Kostyleva et al., Lovatti et al.). Range and depth dose distributions measurements have been performed with a water column setup. These first experimental results will be presented.

### P 123 - In-beam PET imaging and range verification using positron-emitters of carbon produced by the FRS fragment separator of GSI

#### Daria Kostyleva^1^, Peter Dendooven^2^, Sivaji Purushothaman^3^, Emma Haettner^1^, Christoph Scheidenberger^1,4^, Katia Parodi^5^, Marco Durante^6,7^

##### *^1^GSI Helmholtzzentrum fuer Schwerionenforschung, Department of FRS/SFRS Experimente, Darmstadt, Germany*

*^2^University Medical Center Groningen, Department of Radiation Oncology, Groningen, Netherlands*

*^3^GSI Helmholtzzentrum fuer Schwerionenforschung, Department of System Planning SFRS, Darmstadt, Germany*

*^4^Justus-Liebig University Giessen, Department of II. Physikalisches Institut, Giessen, Germany*

*^5^Ludwig-Maximilians Universitaet Muenchen, Department of Medical Physics, Munich, Germany*

*^6^GSI Helmholtzzentrum fuer Schwerionenforschung, Department of Biophysics, Darmstadt, Germany*

*^7^TU Darmstadt, Department of Condensed Matter Physics, Darmstadt, Germany*

For decades, beams of stable ions have been a well-established tool for radiotherapy. In the case of ion-beam therapy with stable ^12^C ions, the positron emitters ^10,11^C are produced via projectile and target fragmentation. Their decay enables visualization of the beam via positron emission tomography (PET). However, the PET activity peak does not very closely match the Bragg peak, and PET counting statistics are low. These issues can be mitigated by using a short-lived positron emitter as a therapeutic beam. An experiment studying range verification of beams of positron-emitting carbon isotopes has been performed at the FRS fragment-separator facility at GSI, Germany, within the BARB project (Biomedical Applications of Radioactive ion Beams, see abstracts Boscolo et al., Lovatti et al.). High purity beams of ^10^C and ^11^C ions with therapy-relevant energies of about 150 and 270 MeV/u and intensities up to 10^9^ ions/sec were produced at the FRS facility. The high purity is illustrated by the particle identification plot of the ^10^C beam in Fig.1(a). The positron-emitters were implanted into tissue-equivalent materials like PMMA, and their high-quality PET images were obtained, see Fig.1(b). The preliminary results of the image analysis will be presented, and the relationship between the quality of the PET image and the dose deposited during ion beam irradiation will be discussed. The investigations conducted, and their results will be used for testing tumor control with radioactive ion beams in pre-clinical settings.

### P 124 - Reproducibility and stability of upright patient positioning for proton radiotherapy

#### Jon Feldman^1^, Sion Koren^2^

##### *^1^Hadassah Medical Center, Radiation Oncology, Jerusalem, Israel*

*^2^Ziv Medical Center, Radiation Oncology, Sefat, Israel*

**Purpose/Objectives:** Historically proton radiotherapy for treatment of brain, head/neck and ocular disease often involved upright or “seated” patient positioning utilizing a fixed beam delivery system. Incorporation of advanced image guidance techniques (In-room CT, CBCT) and robotic patient positioning makes it possible to extend this approach to the treatment of extra-cranial locations including thoracic, abdominal and pelvic sites. In this work, we investigate stability and reproducibility of upright patient positioning, examining intra- and inter-fraction seated patient motion effects.

**Materials and methods:** Patient positioning was examined using the P-Cure Proton Therapy System, 360 gantry-less turnkey solution for treatment of seated patients. Three female, three male subjects, 15-69 yo, 141-188 cm, 40-101 kg were positioned for four treatment sites: head/neck, thoracic, abdomen and pelvis using standard immobilization masks (Orfit Ltd). A 3D optical scanner (ATOS Compact, Gem Inc, 0.017mm accuracy) was used to measure fiducial marks on the subject's skin (small cutouts made in the thermoplastic masks) and on the mask (around the cutouts). Patients were fixated, and position measurements taken during a simulated treatment including the setup position, movement to 4 beam positions and a return to setup position. For each patient, positioning was repeated 3 times, releasing the patient from the system for 5-minute intervals between attempts. This entire study was repeated for each patient after a minimum interval of 5 days.

**Results and conclusions:** Results are reported in attached table. Variation between individual beam positions, all beam positions within a fraction, and all positions between fractions is presented as Inter-beam, Intra-fraction and Inter-fraction repeatability.

### P 125 - Influence of manual contouring in DIR on accumulated VMAT vs IMPT dose evaluation for cervical cancer

#### Elske M. Gort^1^, Jannet C. Beukema^1^, Marjan J. Spijkerman-Bergsma^1^, Marianne L. de Vries-de Groot^1^, Stefan Both^1^, Johannes A. Langendijk^1^, Witold P. Matysiak^1^, Charlotte L. Brouwer^1^

##### ^1^University Medical Center Groningen, Department of Radiation Oncology, Groningen, Netherlands

**Purpose**: Adaptive radiotherapy strategies for cervical cancer patients could be required to maintain target coverage. Manual contouring on repeat-CT scans (reCTs) is time-consuming. We investigated clinical re-planning decision differences based on manual vs automated dose accumulation for VMAT and IMPT.

**Materials and methods**: CTVs and OARs were manually contoured on five weekly reCTs for 12 cervical cancer patients treated with VMAT. Clinical re-planning was performed for 3 patients. Two-arcs VMAT and four-field IMPT plans were made on the planCT and (robustly) evaluated on each reCT^1^. Deformable hybrid intensity and structure-based image registrations (DIR) between planCT and reCTs were performed using the targets as controlling ROIs (DIR_control-ROI) and without controlling ROIs (DIR_automated). The planCT contours were warped using DIR_automated and resulting automated contours and their doses were compared with their manual counterparts. The voxelwise minimum reCT doses were warped back to the planCT using DIR_control-ROI and DIR_automated, and the accumulated doses were compared for conclusions on (not) passing criterion ITV D98>95%.

**Result**: Pass/no pass conclusions on accumulated target coverage improved moving from DIR_automated to DIR_control-ROI for 3 vs 7 out of 12 patients using VMAT and IMPT, respectively (Figure 1A, green-shaded). Using automated vs manual contouring bowel bag doses were underestimated, while pelvic target coverage was overestimated (Figure 1B, Table 1). Manual vs automated contours only agreed for lymph nodes and bony structures (PPV≥0.9).

**Conclusion**: Replacing manual with automated contouring in cervical cancer could lead to inadequate estimation of individual target and OAR doses, and larger DIR-based accumulated dose differences for IMPT compared to VMAT.

### P 126 - Impact of challenging patients on proton therapy recalculation and replanning

#### Filippo Grillo Ruggieri^1^, Alessandra Bolsi^1^, Dominic Leiser^1^, Barbara Bachtiary^1^, Alessia Pica^1^, Nicola Bizzocchi^1^, Michael Zorneth^1^, Arturs Meijers^1^, Damien Charles Weber^2,3^

##### *^1^Paul Scherrer Institute, Center for Proton Therapy, Villigen, Switzerland*

*^2^Paul Scherrer Institute-, Center for Proton Therapy, Villigen, Switzerland*

*^3^University of Bern and University of Zürich, Medical Schools, Bern and Zürich, Switzerland*

**Aim**: To evaluate the need of plan recalculation and re-planning for challenging patients, i.e those with medical and positioning issues, and anatomical changes.

**Material and methods**: From 01.09.2021 to 31.10.2021, 45 patients were in treatment on our two gantries (33% of the yearly gantry workload). All were evaluated in terms of the three different treatment issues affecting the dose distributions: 1) Medical (i.e. pain, dysphagia, nausea), 2) Anatomical changes (i.e. shape, tumor response, cavity filling) or 3) Positioning (i.e. residual rotations, kiphoscoliosis, obesity). Incidences of recalculation and/or re-planning arising from these, some of which were combined, have been evaluated for both Gantry 2 (in-house developed gantry) and Gantry 3 (Varian ProBeam) patients.

**Results**: On Gantry 2, 8/18 (44%) pts were defined as challenging patients due to medical (4), anatomical (4) and/or positioning (5) issues, whilst on Gantry 3, 14/27 (52%) pts were challenging (8 medical, 7 anatomical and/or 5 due to positioning issues). Recalculations were performed on 21 different plans for 14/18 (78%) pts in Gantry 2, resulting in 6/18 (33%) having a total of 6 re-planning. For Gantry 3, 18 re-calculations were performed for 10/27 (37%) pts, of which 8 re-plans were performed for 6/27 (22%) pts.

**Conclusion**: The incidence of plan recalculation and re-planning in our Center is currently substantial. As such, and at least to partially predict such events, a score including medical, anatomical and positioning aspects is currently under development.

### P 127 - Assessment of dosimetry accuracy of deep learning-based synthetic CT for photon and proton robust optimization planning for lung cancer

#### Mi Huang^1^, Zhuobiao Qiao^2^, Zeyu Zhang^3^, Aman Anand^4^, Chen Hao^5^, Zhuoran Jiang^3^, Keith Furutani^1^, Dapeng Wu^2^, Lei Ren^6^, Chris Beltran^1^

##### *^1^Mayo Clinic Florida, Radiation Oncology, Jacksonville, USA*

*^2^University of Florida, Electrical and Computer Engineering, Gainesville, USA*

*^3^Duke University, Medical Physics Graduate Program, Durham, USA*

*^4^Mayo Clinic Arizona, Radiation Oncology, Phoenix, USA*

*^5^Johns Hopkins Sibley Proton Center, Radiation Oncology, Washington. DC, USA*

*^6^University of Maryland School of Medicine, Radiation Oncology, Baltimore, USA*

This study aims to evaluate the dosimetry accuracy of synthetic CT (SynCT) generated from CBCTs using a deep learning Pix2Pix Generative-Adversarial-Network (GAN) for both photon and proton robust planning. To eliminate the registration error raised from CT to CBCT, ∼900 CBCT projections containing primary, scatter, and noise signals were simulated from each patient's CT images using Monte Carlo and then used to reconstruct CBCT with Varian iterative reconstruction package (MC-CBCT). A Pix2Pix GAN was developed to generate synthetic CTs from MC-CBCT with the original CT images as ground truth. The model was trained using 29 lung patients' data and tested using 3 patients' data. Treatment plans were then made for both photon and proton therapy in Eclipse using ground truth CT and SynCT, respectively. 3D robust optimization was done on PTV with 3% range uncertainty and 3mm setup uncertainly around the target for proton plans. For image quality comparison, SSIM (structural-similarity-index), PSNR (peak-signal-to-noise-ratio) and MAE (mean-absolute-error) were accessed for MC-CBCT and synthetic CTs. Dosimetric metrics, including DVH for PTVs, OARs, and 3D gamma index, were calculated among different plans using SynCT to CTs. The comparison results are presented in Figure1 and Table1. Image quality comparison is presented in Table2. For image quality (SSIM, PSNR, MAE) SynCT achieves significant improvement over MC-CBCT and similar quality to CT. For plan quality, Gamma-index was above 94% and 97% for proton and photon plans, respectively. For proton optimized plans, SynCT demonstrated the close dosimetric performance quality compared with CT.

### P 128 - Commissioning of an in-room CT on rails (CTOR) system for adaptive proton radiotherapy

#### Hansjoerg Wertz^1^, Patricia Cambraia Lopes^1^, Paul van Beers^1^, Jesse Clarijs-de Jong^1^, Tina Lengkeek^1^, Carina Brama^1^, Edwin Timmermans^1^, Eva van Weerd^1^, Anne Lisa Wolf^1^, Mischa Hoogeman^1^, Jasper Kouwenberg^1^

##### ^1^Holland PTC, Holland PTC, Delft, Netherlands

**Purpose**: Anatomical changes during the radiotherapy treatment course can cause negative dosimetric consequences particularly for proton therapy due to its sensitivity to range changes. To determine the effects, a CT on rails (CTOR) inside the treatment room can be used. The aim was to setup and verify a workflow for detection of anatomical changes, using a CTOR system for dose evaluation and plan adaptation.

**Material and methods**: A CTOR (SOMATOM Confidence RT, Siemens) implemented within a proton treatment facility (ProBeam, Varian) with pencil beam scanning gantry was commissioned. Hounsfield Unit (HU) to mass density calibration curves were measured and evaluated. Dose distributions based on CTOR scans were calculated on inhomogeneous phantoms including target and organ mimicking structures and compared with calculations based on standard planning CT (PCT) scans (dose difference maps). A full adaptive workflow was tested in addition.

**Results**: The CTOR behaved similar to a standard diagnostic CT scanner in terms of image quality, imaging dose and geometric accuracy. The scans were suitable for dosimetric calculations and treatment planning. Dosimetric comparisons showed very good agreement. Dose differences were in general within 2% (in small regions up to around 5-6%), dose profiles and dose volume histograms agreed well and resulting average doses for artificially created structures were within around 1Gy (70Gy prescribed dose). A full adaptive workflow was tested successfully.

**Conclusion**: The in-room CTOR system can be used for dose evaluation and plan adaptation. Current work also focuses on using the CTOR system for online adaptations of the treatment plan.

### P 129 - Dosimetric investigation of upright versus supine proton treatments

#### Sion Koren^1^, Jon Feldman^2^, Michael Marash^3^

##### *^1^Ziv Medical Center, Radiation Oncology, Safed, Israel*

*^2^Hadassah Medical Center, Radiation Oncology, Jerusalem, Israel*

*^3^P-Cure Ltd, Administration, Lod, Israel*

**Purpose/Objectives:** Proton radiotherapy systems utilizing upright, 6 DOF robotic patient positioning, introduce the ability to treat a range of extra-cranial target sites without a gantry, reducing cost and complexity, thereby improving access to a larger community of patients. To utilize upright positioning in routine treatment, careful consideration must be made regarding organ displacement and normal tissue toxicity. This study utilizes a computational phantom, XCAT, to simulate a large population of upright and supine patients, where a treatment planning study may be executed to examine potential changes in organ toxicity and anatomic access, comparing supine and upright patient orientations.

**Methods and materials:** Five female and five male computational phantoms were generated using the XCAT software, representing 90% coverage of international height and weight by population according to gender. Each phantom was generated in both an upright (seated) and supine orientation. Tissues for upright patients were displaced according to actual human seated CT data, taken from the P-Cure P-Artis robotic 6DOF patient positioning system. Treatment plans, 180 in total, were generated for brain, nasopharynx, bilateral head/neck, parotid, mediastinum, lower lung, pancreas, liver and prostate for identical target volumes on all patients and orientations using identical target coverage goals using the Pinnacle 16.2 TPS.

**Results/Conclusions:** Results for some statistically significant variances are presented in accompanying table. Plans for brain, parotid, lower lung and liver demonstrated no statistically significant variation as a result of patient orientation. In general, no significant increase in toxicity was found for upright positioning when compared with supine oriented patients.

### P 130 - Hypoxia based IMPT dose escalation using HX4 PET images in HNSCC patients

#### Shubhangi Makkar^1,2^, Anne-Sophie Bogaert^1^, Barbara Bachtiary^1^, Catharina M.L. Zegers^3^, Erik Roelofs^3^, Keegan McNamara^1,2^, Jan Hrbacek^1^, Damien C. Weber^1,4,5^, Antony Lomax^1,2^, Carla Winterhalter^1,2^

##### *^1^Paul Scherrer Institute, Center for Proton Therapy, Villigen PSI, Switzerland*

*^2^ETH Zürich, Department of Physics, Zürich, Switzerland*

*^3^Maastricht University Medical Centre+, Department of Radiation Oncology Maastro- GROW School for Oncology, Maastricht, Netherlands*

*^4^University Hospital of Bern, Department of Radiation Oncology, Bern, Switzerland*

*^5^University Hospital of Zürich, Department of Radiation Oncology, Zürich, Switzerland*

This in-silico planning study investigates the potential of hypoxia-based dose painting for head and neck squamous cell carcinoma (HNSCC). For a patient with T2N2b oropharyngeal SCC, HX4-PET images are used to delineate a hypoxic volume with tumour-to-muscle ratio > 1.4. A non-linear conversion model is used to obtain partial oxygen pressure (pO_2_) which along with LET_d_ is used for the proton based OER calculation. The patient specific dose boosting factor, derived using the OER, is 12.5 % (mean) to the hypoxic PTV. Three radiotherapy plans are compared: conventional IMRT and IMPT plans with a prescription dose of 70 Gy to the high dose PTV, and a hypoxia dose escalated plan with 78.8 Gy prescribed to the hypoxic sub-volume within the high dose PTV (Figure 1). Tumour control probabilities (TCP) are evaluated using a Poisson model based on disjoint sub-volumes taking into account the spatially varying local failures. Targeting hypoxia improves the TCP by approximately 9% compared to the conventional plans. The dose escalated proton plan simultaneously reduces the mean doses to oral cavity by 8.75 Gy_RBE_, contralateral submandibular by 21.85 Gy_RBE_, contralateral parotid by 2.7 Gy_RBE_, and spinal cord by 13.75 Gy_RBE_ compared to the non-dose escalated photon plan (Table 1). This decrease in mean doses to critical organs with dose escalated IMPT translates to significant NTCP reduction of dysphagia by 26% and of oral mucositis by 10%.

### P 131 - Suitability of low-dose CT scanning protocols for online adaptive proton therapy

#### Konrad Nesteruk^1^, Mislav Bobić^1^, Gregory Sharp^1^, Arthur Lalonde^1^, Brian Winey^1^, Antony Lomax^2^, Harald Paganetti^1^

##### *^1^Massachusetts General Hospital and Harvard Medical School, Department of Radiation Oncology, Boston, USA*

*^2^Paul Scherrer Institute, Center for Proton Therapy, Villigen, Switzerland*

Imaging doses can be significant when CBCT or CT-on-rails scans are repeated daily. This may compromise one of proton therapy aims to minimize the dose to healthy tissue. Low-dose scanning protocols address this problem. We evaluate the influence of using low-dose CT protocols on online plan adaptation based on a fast GPU Monte-Carlo algorithm. We acquired scans of a head phantom with protocols corresponding to CTDI_vol_ in the range 4.2-165.9 mGy. The highest value corresponds to the standard protocol used for CT simulations of 10 head-and-neck patients. For each patient and each low-dose protocol, the noise relative to the standard protocol was applied to the planning CT and to a virtual CT (vCT). The vCT was obtained from a daily CBCT scan corresponding to the fraction with the largest anatomical changes. We ran an established adaptive workflow twice for each low-dose protocol - using a high-quality daily vCT and the corresponding low-dose synthetic vCT. For a relative comparison of the adaptation efficacy, two adapted plans were evaluated on the high-quality vCT with the contours obtained through deformable registration of the planning CT. Results show that the Dice similarity coefficient between propagated contours obtained with low-dose and standard protocols is in the range 0.980-0.998, while discrepancy in D_98_ for CTV and mean dose to OARs are within 0.6 % and 3.0 %, respectively. We conclude that low-dose CT protocols are suitable for online adaptive proton therapy of head-and-neck cancers.

### P 132 - Inhomogeneity detection in carbon-ion beam therapy based on nuclear fragmentation tracking

#### Renato Felix-Bautista^1,2,3^, Laura Ghesquière-Diérickx^1,3,4^, Pamela Ochoa-Parra^1,3,5^, Laurent Kelleter^1,3,6^, Marcus Winter^7^, Gernot Echner^1,3^, Oliver Jäkel^1,3,6,7^, Tim Gehrke^1,2,3^, Mária Martišíková^1,3,6^

##### *^1^German Cancer Research Center DKFZ, Medical Physics in Radiation Oncology, Heidelberg, Germany*

*^2^Heidelberg University Hospital, Radiation Oncology, Heidelberg, Germany*

*^3^Heidelberg Institute for Radiation Oncology HIRO, National Center for Research in Radiation Oncology NCRO, Heidelberg, Germany*

*^4^Heidelberg University, Medical Faculty, Heidelberg, Germany*

*^5^Heidelberg University, Faculty of Physics and Astronomy, Heidelberg, Germany*

*^6^National Center for Tumor Diseases NCT, National Center for Tumor Diseases NCT, Heidelberg, Germany*

*^7^Heidelberg Ion Beam Therapy Center HIT, Radiation Oncology, Heidelberg, Germany*

Non-invasive monitoring methodologies based on the tracking of nuclear fragments produced during the carbon-ion treatment delivery have been under development for ion range verification and monitoring of lateral beam positions. This contribution aims at the evaluation of the performance of our method for detecting inter-fractional changes in the irradiated object for clinical treatment plans. The experiments were conducted at the HIT facility in Heidelberg, Germany. A carbon-ion treatment plan with a homogeneous RBE-weighted dose of 3 Gy (RBE) was designed and irradiated to a head-sized PMMA phantom. The target volume was 50 cm^3^. Nuclear fragments were tracked by means of a mini-tracker of 2-cm^2^ sensitive area based on silicon pixel Timepix3 detectors. This mini-tracker was placed at 30° relative to the beam axis. In order to mimic an inter-fractional change, a disk-shaped air cavity of 2 cm diameter and 0.2 cm thick was used in the irradiated phantom for a second measurement. Perturbations in the measured nuclear fragment emission profiles were assessed to detect the presence of the small air cavity. By considering a realistic tracking system with 14-fold detection area, such perturbations were detected with more than 2.7 of significance for cavity positions upstream of the target. We successfully localized the cavity in a 4-cm^2^ area within the target volume from the beam's eye view. This work quantitatively demonstrates the ability of our methodology for detecting small structure changes in realistic treatment deliveries. This non-invasive treatment monitoring methodology is promising for further investigations in clinical trials.

### P 133 - Design of a multi-organ custom phantom for particle therapy applications

#### Andrea Pella^1^, Rosalinda Ricotti^1^, Gabriele Belotti^2^, Alessandro Vai^3^, Stefania Russo^3^, Barbara Vischioni^4^, Chiara Paganelli^2^, Mario Ciocca^3^, Ester Orlandi^5^, Guido Baroni^1,2^

##### *^1^National Center of Oncological Hadrontherapy CNAO, Bioengineering Unit- Clinical Department, Pavia, Italy*

*^2^Politecnico di Milano University, Department of Electronics- Information and Bioengineering, Milan, Italy*

*^3^National Center of Oncological Hadrontherapy CNAO, Medical Physics Unit- Clinical Department, Pavia, Italy*

*^4^National Center of Oncological Hadrontherapy CNAO, Radiotherapy Unit- Clinical Department, Pavia, Italy*

*^5^National Center of Oncological Hadrontherapy CNAO, Clinical Department, Pavia, Italy*

The aim of this study was to design and evaluate an anthropomorphic phantom representative of the pelvic district. It was customized for quantitative studies on setup verification procedures exploiting the capabilities of in room soft tissue imaging as cone beam CT (CBCT). We considered prostate gland (PG), urinary bladder (UB), rectum (R) and femurs as organs of interest. We focused on the layout more than the radio-equivalency of materials: PG has been represented with wood, UB with a liquid inflated balloon and R with spongy material. Properly pre-treated swine bones were preferred for femurs. All the components were fixed without any metal or magnetic parts in a water box, to simplify indexing and positioning on diagnostic or treatment supports. Different configurations of rectum, prostate gland and urinary bladder are allowed. Bones were fixed on the support to facilitate comparisons between rigid and non-rigid registration performances. The characterization of the phantom foreseen CT scans (Siemens Somatom) and in room CBCT acquisitions (Figure1). In this preliminary assessment, a treatment plan was roughly optimized (Raystation), to simulate a prostate treatment. The phantom and an example of in room alignment relying on CBCT are depicted in figure 1. CT scan reported a mean Hounsfield unit distribution of PG, UB and R of -313HU, 60HU, -562HU respectively. This phantom could be useful in a wide range of applications. Efforts will be devoted to materials selection and to studies aimed at dose recalculation based on CBCT information, simulating intra-fraction physiological variability of patients.

### P 134 - Patient positioning workflow optimization at a light ion gantry based on optical surface guidance

#### Abdallah Qubala^1,2,3^, Andrea Schwahofer^3,4^, Stefan Jersemann^1,3^, Saleh Eskandarian^1,3^, Semi Harrabi^1,3,5,6^, Patrick Naumann^1,3,5,6^, Malte Ellerbrock^1,3^, Marcus Winter^1,3^, Klaus Herfarth^1,3,5,6^, Oliver Jäkel^1,3,4,6^

##### *^1^Heidelberg Ion-Beam Therapy Center HIT, Department of Radiation Oncology- Heidelberg University Hospital, Heidelberg, Germany*

*^2^Heidelberg University, Faculty of medicine Heidelberg, Heidelberg, Germany*

*^3^National Center for Radiation Research in Oncology NCRO, Heidelberg Institute of Radiation Oncology HIRO, Heidelberg, Germany*

*^4^German Cancer Research Center DKFZ, Medical Physics in Radiation Oncology, Heidelberg, Germany*

*^5^Heidelberg University Hospital, Department of Radiation Oncology, Heidelberg, Germany*

*^6^National Center for Tumor Diseases NCT, Department of Radiation Oncology, Heidelberg, Germany*

**Introduction:** A Surface Guided Radiation Therapy (SGRT) system for tracking of patient positioning before and during treatment has been installed in our light ion beam gantry. Here we present first clinical results for accuracy, reliability and efficiency of SGRT used in particle beam treatments.

**Materials and methods:** The study included 32 patients, divided into three cohorts: pelvis, limb, and chest/spine. The statistical analysis is based on 300 fractions. Two patient positioning workflows (WF) were compared followed by a planar kV imaging for verification: (1) positioning by in-room-laser using skin marks, (2) positioning by SGRT.

**Results:** The use of SGRT significantly decreases the group translational couch magnitude shifts by 0.56±1.41 mm for pelvis patients and 1.89±0.46 mm for limb patients, whereas for chest/spine patients SGRT increases the magnitude shifts by 0.71±0.32 mm (p < 0.05). The necessary rotational corrections were generally lower with SGRT for all cohorts with significant differences in pitch for pelvis and chest/spine cohorts (p < 0.05). Using SGRT for patient positioning for entire cohort has shown a significant reduction of re-positioning and re-imaging by 11 to 3 times for skin marks and SGRT, respectively. In comparison to conventional skin marks positioning, the SGRT-WF required 18%, 9%, and 15% less time for the entire setup procedure for pelvis, limb, and chest/spine patients.

**Conclusions:** In this study, we found that the patient positioning WF prior to treatment can be optimized using SGRT without additional imaging dose. Furthermore, a marked reduction of positioning time and re-imaging frequency was also shown.

### P 135 - The clinical accuracy and registration efficiency of proton radiography for intracranial target alignment using different image acquisition configurations

#### Hazel Ramirez^1^, Mark Pankuch^1^, DeJongh Ethan^2^, Fritz DeJongh^2^

##### *^1^Northwestern Medicine Chicago Proton Center, Medical Physics, Warrenville, USA*

*^2^ProtonVDA, Physics, Naperville, USA*

**Purpose**: Proton radiography (pRad) provides images that can be used to align the patient. For pRad to be considered acceptable for patient alignment, pRads should produce similar accuracy and time efficiency as standard alignment methods. In pRad, clinical image quality is dependent on the detector configuration and the total number of protons used for reconstruction. In this study, we quantify the difference in alignment accuracy and registration durations for two pRad detector configurations using two different total proton fluences.

**Methodology**: A head CT was randomly offset in 15 combination of positional and 15 positional and rotational offsets. X-Ray and pRad DRR's were simulated and reconstructed for each of the offset conditions then used to obtain offset vectors by four expert RTT's using clinical IGRT software. pRad were reconstructed in three configurations: single downstream detector with 4.5M protons(single/4.5M), single downstream detector with 18M protons(Single/18M) and double downstream detectors with 18M protons(Double/18M). The absolute accuracy of the alignment and the time required to complete the registration were collected.

**Results**: The average positional offset vector was 0.45mm for X-rays and <0.70mm for all pRad configurations. The average rotational offset was <0.04 degrees for X-rays, <0.4 degrees for Single/4.5M and <0.03degrees for Single/18M and Double/18M. The standard deviation for position and angle were larger for pRad. The average duration to obtain the registration vector was 49.5s, 51.1s, 45.9s and 46.8s for X-ray, Single/4.5M, Single/18M and Double/18M respectively.

**Conclusions**: pRad can be used for cranial alignment with similar accuracy and registration duration of X-ray methods.

### P 136 - An autocorrelation function analysis of noise properties of proton CT images

#### Ethan DeJongh^1^, Alexander Pryanichnikov^2,3^, Fritz DeJongh^1^, Reinhard Schulte^4^

##### *^1^ProtonVDA LLC, Physics, Naperville, USA*

*^2^Moscow State University, Physics, Moscow, Russian Federation*

*^3^Physical-Technical Center of Lebedev Physical Institute, Physics, Protvino, Russian Federation*

*^4^Loma Linda University, School of Medicine, Loma LInda, USA*

**Purpose**: A least-squares iterative algorithm for proton CT (pCT) image reconstruction converges toward a unique solution that optimally fits the protons. We present a stopping criterion that stops iterations when correlations of relative stopping power (RSP) between voxels are relatively low, providing a method to produce pCT images with standard noise properties useful for treatment planning, which relies on summing RSP along columns of voxels.

**Methodology**: With simulated and real images with varying heterogeneity from the ProtonVDA system, we calculate autocorrelation from average RSP correlations between voxel pairs in a uniform region-of-interest versus distance between voxels. We define a stopping criterion based on *r*, the remaining distance to the unique solution relative to estimated image noise.

**Results**: As shown in the figures for a pCT image of a pork shoulder and ribs, there are large correlations between voxels for larger values of *r,* and anticorrelations for smaller values. For *r* in the range of 0.5 to 1, voxels are relatively uncorrelated, and compared to smaller values of *r* have lower noise with only slight loss of spatial resolution.

**Conclusions**: A least-squares iterative algorithm with an *r-*based stopping criterion defines a pCT image reconstruction method with consistent statistical properties such as noise power spectra which can be used to compare image noise and dose of pCT with other modalities such as dual-energy CT.

### P 137 - Investigation of on-line PET image-based proton intra-beam range verification and range-guided adaptive beam delivery

#### Dongxu Yang^1^, Xiaorong Zhu^2^, Lin Ma^1^, Mingli Chen^1^, Weiguo Lu^1^, Yang Kyun Park^1^, David Grosshans^3^, Yiping Shao^1^

##### *^1^University of Texas Southwestern Medical Center, Radiation Oncology, Dallas, USA*

*^2^University of Texas MD Anderson Cancer Center, Radiation Physics, Houston, USA*

*^3^University of Texas MD Anderson Cancer Center, Radiation Oncology, Houston, USA*

This study investigated the capability of using a newly developed on-line PET to measure the proton-induced activity-range (AR) within a fractionated therapy. This approach could permit using a fraction of therapeutic beam to verify the beam-range (BR) and when necessary to adaptively modify the remaining beam delivery to compensate the range-shift. PET consists of 20 detector panels in a polygon configuration with ∼3mm resolution and ∼32cm diameter and 6.4cm axial field-of-view, with two removable detector panels for beam passing. Protons with 105MeV energy irradiated a Lucite phantom consisting of a replaceable Lucite rod inserted inside a ∼16x16x16 cm water tank for including attenuation and scatter events in PET data. Additional Lucite sheets with different thicknesses were placed between the rod and beam nozzle for measuring the distal activity position (50% peak) vs the sheet thickness. Same irradiation-imaging studies were also conducted with a Stereotactic End-to-End Verification (STEEV) phantom. With 0.3Gy (at the Bragg peak) beam and 60-second PET acquisition, data with Lucite and STEEV phantoms showed ∼0.9mm and ∼2.1mm AR standard deviations, respectively, and excellent linearity of distal activity position vs inserted sheet thickness. Both phantom studies also showed <1.5mm difference in ARs measured with 0.3 to 6 Gy beams. We are investigating the “range-shift compensated” adaptive beam delivery with the STEEV phantom that will be irradiated by planned and mimicked “overshot” or “undershot” 0.3Gy beams through different inserted Lucite sheets. We will assess the accuracy by measuring and comparing their dose distributions with inserted Gafchromatic films.

### P 138 - Commissioning of a Surface Guidance system for patient positioning, monitoring and beam gating during proton treatments

#### Gloria Vilches-Freixas^1^, Ana Vaniqui^1^, Vicki Trier Taasti^1^, Esther Kneepkens^1^

##### ^1^Department of Radiation Oncology MAASTRO- GROW School for Oncology- Maastricht University Medical Centre+- Maastricht- The Netherlands, Maastro, Maastricht, Netherlands

**Introduction**: We report the results of the commissioning of the first C-RAD Catalyst X4 surface scanning system installed on a Mevion S250i Hyperscan system for positioning, monitoring and gating during proton therapy treatment.

**Materials and methods**: The surface guidance (SG) system is installed in a unique two-isocenter configuration consisting of 4 cameras to enable SG during both treatment and CBCT imaging using a medPhoton Imaging Ring (Fig.1). The SG isocenter was checked against the couch, laser, CBCT and kV-kV isocenter. Static and dynamic localization accuracy were determined comparing to kV and CBCT. Temporal camera drift and field-of-views at both treatment and imaging isocenter were assessed. For gating, the accuracy of the breath-hold and respiratory trace detection was tested and dose consistency between gated and non-gated treatment was checked. End-to-end tests were performed to optimize and verify the patient workflow. Phantoms used are shown in Fig.1.

**Results**: A selection of results is reported in Table 1. Static and dynamic localization accuracy were 0.8±0.4 mm and 0.6±0.3 mm respectively.

**Discussion and Conclusion**: Based on the commissioning results, C-RAD was released for clinical use in December 2021 and is currently used for positioning of breast cancer patients and compared to CBCT. First clinical results are being collected. While the commissioning ensured SG accuracy, clinical practice still provided challenges e.g. the surface being obscured through a combination of nozzle position and patient anatomy. Therefore, testing on volunteers with different anatomies is encouraged. The next application is gating in breath-hold for breast-cancer and lymphoma patients.

### P 139 - Workflow treatment times for a couch mounted CBCT image guided proton therapy system

#### Brian Winey^1^, Gregory Sharp^1^, Benjamin Clasie^1^

##### ^1^Massachusetts General Hospital/Harvard Medical School, Radiation Oncology, Boston, USA

A novel compact proton therapy system has been deployed clinically. The treatment system utilizes a half-gantry proton scanning nozzle and a CBCT imaging ring connected to the patient positioning system (PPS). The integrated CBCT imaging and PPS should allow for rapid image guided proton therapy. Patients treated in the systems were selected for timing analysis from a variety of treatment sites. The system creates a record of the treatment session that includes the time stamps at the creation of the CBCT image and the time of the session record. The difference in time between the two time stamps will exclude the initial patient positioning on the couch and acquisition time of the CBCT, but will include image registration. Brain patients were treated 3 field plans with average treatment time of 22.9±2.7 minutes. Other sites included H&N (2 fields, 24.4±1.7 minutes; 3 fields, 27.2±6.6 minutes), prostate (2 fields, 24.4±3.2), mediastinal (1 field, 23.6±6.7; 2 fields, 31.8±4.8 minutes), and sarcoma spine (2 fields, 23.5±2.5 minutes). The current workflow requires greater time compared to prior proton therapy experiences with 2D imaging. Time savings for the current workflow are limited by the beam delivery time and speed of motion of the gantry and PPS. Future system developments are anticipated to decrease the workflow time.

### P 140 - Evaluation of two alternating plans versus a single plan for proton therapy in the setting of metal implants

#### Chih-Wei Chang^1^, Mark McDonald^1^, Zachary Diamond^1^, Jun Zhou^1^

##### ^1^Emory University, Radiation Oncology, Atlanta, USA

**Purpose:** We evaluated the dosimetric impact of a novel alternating plan delivery (APD) technique intended to mitigate uncertainties with proton therapy in patients with metal implants.

**Methods:** We evaluated fifteen patients (12 metal implants, 3 tumors abutting critical OAR) with tumors of the spine or sacrum previously treated with APD. In APD, two comparable plans (A & B) were generated using different (typically 2-5° shift) beam angles and spot positions, delivered on alternating days. To evaluate the impact of APD on patient variation over the treatment course, the deformed cumulative dose (DCD) was summed using the first CBCT of each week (see figure). V100, D99, D95, and D0.03cc for the highest dose level CTV and D0.03cc for the cord/cauda equina were compared between the DCD of Plan A, Plan B, or APD using related-sample Wilcoxon Signed-Rank test.

**Results:** DCD_APD CTV V100, D99, and D95 were significantly higher than DCD_PlanB, (91.9 ±5.9% vs 91.2 ±6.2% (p=0.013), 92.0 ±7.8% vs 91.7 ±7.8% (p=0.015), 98.0 ±3.8% vs 97.8 ±3.8%, (p=0.012), but not those in DCD_PlanA. The cumulative cord/cauda D0.03cc dose of DCD_APD was significantly lower (-1.3±1.7% of nominal, p<0.01) than the worst value from DCD_PlanA or DCD_PlanB.

**Conclusion:** When evaluating variation over the treatment course, APD had better target coverage compared to one of the two plans and reduced maximum dose to the cord/cauda compared to a single plan. Further steps include evaluation of risk to serial organs with the end of range RBE uncertainty.

### P 141 - Motion model driven log-file based 4D proton dose reconstruction: proof of concept

#### Alisha Duetschler^1,2^, Lili Huang^1,3,4^, Giovanni Fattori^1^, Gabriel Meier^1^, Christian Bula^1^, Sairos Safai^1^, Damien C Weber^1,5,6^, Antony J Lomax^1,2^, Ye Zhang^1^

##### *^1^Paul Scherrer Institute, Center for Proton Therapy, Villigen PSI, Switzerland*

*^2^ETH Zurich, Department of Physics, Zurich, Switzerland*

*^3^ETH Zurich, Department of Mechanical and Process Engineering, Zurich, Switzerland*

*^4^EPFL, School of Basic Sciences, Lausanne, Switzerland*

*^5^University Hospital of Zurich, Department of Radiation Oncology, Zurich, Switzerland*

*^6^University of Bern, Department of Radiation Oncology, Bern, Switzerland*

**Purpose**: The conventional approach to retrospective 4D dose reconstruction in proton therapy with pencil beam scanning relies on a single pre-treatment 4DCT. However, breathing motion can vary considerably in both frequency and amplitude intra- and inter-fractionally. We present a 4D dose calculation (4DDC) method considering respiratory irregularity that combines machine log-files with a patient-specific motion model used for the estimation of time-resolved CT images from a respiratory surrogate.

**Methods and materials**: Principal component analysis is applied to relate an external motion surrogate with anatomical deformations in the planning 4DCT. For 4DDC, the motion model is used to generate synthetic time-resolved images by warping the planning or daily CT according to displacements of a bodymarker monitored in real-time during the treatment with an optical-tracking-system (OTS) (Figure 1). The model was validated on a clinical dataset and four fractional doses were reconstructed for one abdomen patient treated with respiratory gating and rescanning.

**Results**: Considerable variations in motion frequency and amplitude were observed (Figure 2(a)), especially for later fractions. The reconstructed 4D dose based on daily CT and actual motion during treatment showed inter-fraction differences up to 1.6% in CTV V_95%_ and 17.2% in D_max_ spinal cord (Figure 2(b)). 4D fraction doses with daily motion on planning CT compared to same motion on daily CT resulted in differences up to 2.3% (20.3%) in V_95%_ (D_max_).

**Conclusion**: A framework for retrospective 4D dose reconstruction was implemented and validated by considering both intra- and inter-fractional motion and anatomy changes. The results show the importance of considering both variabilities for retrospective 4D dose reconstruction.

### P 142 - A tool for the evaluation of 4D proton therapy treatments utilising novel motion models

#### Sam Manger^1^, Adam Aitkenhead^2^, Matthew Lowe^2^, Gareth Price^1^, Jamie McClelland^3^, Michael Taylor^1^

##### *^1^University of Manchester, Division of Cancer Sciences- School of Medical Sciences- Faculty of Medicine Biology and Health, Manchester, United Kingdom*

*^2^The Christie NHS Foundation Trust, Christie Medical Physics and Engineering, Manchester, United Kingdom*

*^3^University College London, Department of Medical Physics and Biomedical Engineering, London, United Kingdom*

Proton beam therapy has the potential to significantly improve the outcomes of thoracic tumour patients due to its excellent dose localisation properties compared to conventional X-ray radiotherapy. This high dose conformality, however, presents a challenge for moving tumours which is reflected in the slow adoption of proton therapy for, for example, lung cancer. The availability of 4DCT imaging offers the opportunity to plan proton therapy treatments incorporating organ motion however conventional 4DCT reconstruction relies on the assumption that organ motion is repeatable, with no breath-to-breath variability. A new approach to 4DCT reconstruction uses motion modelling to map internal motion as a function of a surrogate input signal. The motion model is fitted while simultaneously registering individual 4DCT slices and allows internal motion to be parameterised. In turn, this allows us to study the effects of variation in the breathing cycle on radiotherapy treatments to understand the efficacy of proton therapy treatment techniques for centrally located tumours. In this project we are developing an in-silico tool, incorporating state-of-the-art Monte-Carlo radiation transport simulations and tumour motion models to aid oncologists in assessing the efficacy of proton therapy for lung tumours on a patient-by-patient basis. The tool will shed light on key parameters affecting treatment such as breathing irregularity, the interplay effect, dose to organs at risk and the effectiveness of current motion mitigation strategies. We will present an overview of the methodology used and the results to-date.

### P 143 - 4D dose evaluation of airgap reduction in proton therapy for mediastinal lymphoma patients

#### Pietro Pisciotta^1^, Sabine Visser^1^, Adriaan C. Hengeveld^1^, Anne Niezink^1^, Anne Crijns^1^, Gabriel Guterres Marmitt^1^, Cássia Oraboni Ribeiro^1^, Johannes A. Langendijk^1^, Stefan Both^1^

##### ^1^University Medical Centre Groningen, University of Groningen, Department of Radiation Oncology, Groningen, Netherlands

**Introduction**: Large airgaps increase plan robustness due to larger spot size but induce higher dose to organs-at-risk (OARs). The main aim of this study was to evaluate the impact of smaller airgaps on plan robustness and OARs dose in mediastinal lymphoma patients treated with intensity-modulated proton therapy (IMPT).

**Materials and methods**: Thirteen patients with mediastinal lymphoma were evaluated retrospectively. A 4DCT was acquired for treatment planning and weekly repeated 4DCTs were used to assess any deviations in target coverage along the treatment course. Layer repainting (5x), large airgaps (∼20cm) and 3D-robust optimization were used to generate clinical plans. An additional plan was created with the smallest possible airgap (5-7.5cm) for each patient. For the two air gap configurations, patient-specific dose accumulation of the entire treatment course was performed using an in-house developed 4D robustness evaluation method (4DREM), which simulates range and setup errors in combination with machine errors (log files), anatomy changes and breathing motion (repeated 4DCTs) and interplay effects (log file based splitting the plan on 4DCT phases).

**Results**: The target was adequately covered for both airgap configurations in all patients according to the nominal and 4DREM scenarios (V95>99% and D98>97%). Dose statistics reduction in OARs were observed both in nominal and 4DREM doses when the airgap was decreased (Fig.1 a)-b)).

**Conclusion**: This study indicates that airgap reduction is feasible for 3D robust optimized plans, maintaining clinically acceptable target coverage and decreasing the OARs dose. The latter is especially relevant for reducing normal tissue complication probability (NTCP) and the risk of secondary cancers in young patient population.

### P 144 - Clinical applicability of deep-learning based respiratory signal prediction models for radiation therapy

#### Jeong Sangwoon^1^, Wonjoong Cheon^2^, Sungkoo Jo^3^, Youngyih Han^3,4^

##### *^1^Sungkyunkwan University, Department of Health Sciences and Technology, seoul, Korea Republic of*

*^2^Proton therapy center- National cancer center, Department of Radiation Oncology, Goyang, Korea Republic of*

*^3^Samsung Medical Center, Department of Radiation Oncology, Seoul, Korea Republic of*

*^4^Sungkyunkwan University, School of Medicine, Seoul, Korea Republic of*

**Purpose**: In order to reduce the dose uncertainty in four-dimensional radiation therapy (4DRT), an accurate compensation for the beam on-off latency is important.. Thus, we investigated the accuracy of deep-learning models (Long Short-Term Memory: LSTM, Bi-directional LSTM: Bi-LSTM and Transformer) in predicting the breathing motion and evaluated the clinical applicability to 4DRT.

**Methods**: Of the 527 data collected by the respiratory tracking system (AZ-733VI, Anzai Medical Co, Ltd.) during 4DCT of proton therapy, 27 were assigned to a test set, and the remaining 500 data were grouped into a training (70%) and a validation set (30%). Prediction accuracies were measured using the Root Mean Square Error (RMSE) and Correlation Coefficient (CC) for different input training sequences (T_s_: 1000, 1200, 1400msec) and different output prediction points (P_t_: 300, 500, 700, 900msec). By equating the respiration signals with target position (max motion 5mm), the prediction errors were measured in mm at 30% and 70% of respiration phases.

**Results**: Under the condition of T_s_: 1000msec and P_t_: 500msec, RMSE was 0.2501, 0.2554, 0.2367 and CC was 0.9667, 0.9640, 0.9717 for LSTM, Bi-LSTM, Transformer, respectively. Errors predicting the target positions were: the maximum of the average errors was 0.39mm, 0.68mm, 1.55mm and the maximum error was 2.20mm, 3.90mm, 4.58 mm at the beam on-off latency of 300, 500, 700msec, respectively.

**Conclusions**: The preliminary results demonstrate that Transformer outperformed LSTM and Bi-LSTM. And the three models provide averaged error of less than 1.5mm for 5mm of tumor motion for gated 4DRT.

### P 145 - Deep learning based 4D-synthetic CTs from CBCTs for proton dose calculations in adaptive proton therapy workflows

#### Adrian Thummerer^1^, Carmen Seller Oria^1^, Sabine Visser^1^, Paolo Zaffino^2^, Gabriel Guterres Marmitt^1^, Joao Seco^3,4^, Johannes A. Langendijk^1^, Antje Knopf^1,5^, Maria F. Spadea^2^, Stefan Both^1^

##### *^1^University Medical Center Groningen UMCG, Department of Radiation Oncology, Groningen, Netherlands*

*^2^Magna Graecia University, Department of Experimental and Clinical Medicine, Catanzaro, Italy*

*^3^Deutsches Krebsforschungszentrum, Department of Biomedical Physics in Radiation Oncology, Heidelberg, Germany*

*^4^Heidelberg University, Department of Physics and Astronomy, Heidelberg, Germany*

*^5^University Hospital of Cologne, Department I of Internal Medicine- Center for Integrated Oncology Cologne, Cologne, Germany*

**Purpose/Objective**: Deep convolutional neural networks (DCNN) have shown promising results for creating CBCT-based synthetic CTs (sCTs) for accurate proton dose calculations in adaptive proton therapy (APT) workflows. In the thorax, so far only DCNN-based 3D-sCTs were investigated, without accounting for breathing motion and its impact on dose distributions. In this study we extended our 3D-sCT generation method to 4D, and investigated image quality and dosimetric accuracy of 4D-sCTs.

**Methods**: A dataset containing 4D-CTs and same-day 4D-CBCTs of 50 lung cancer patients was used to train/evaluate a U-net like DCNN (training: 28 patients, validation: 4, testing: 18) to generate 4D-sCTs. To generate reference images, individual phases of the 4D-CT were registered to the 4D-CBCT phases. Image quality of the resulting 4D-sCTs was evaluated by calculating mean absolute error (MAE) for the extreme phases (0% and 50%), an average sCT (4D-sCT-ave) and a 3D-sCT. Dosimetric accuracy was quantified globally (3%/3mm gamma analysis) and locally (CTV and Lung) by recalculating clinical treatment plans.

**Results**: Figure 1 shows an image comparison of 4D-CT, 4D-sCT, 4D-sCT-ave, and 3D-sCT, together with MAE-values. Highest MAE was observed for the individual 4D-sCT phases (48.1±6.5 HU). 4D-sCT-ave (37.2±7.7 HU) and 3D-sCT (37.7±6.2 HU) resulted in similar MAE. Figure 2 presents results from the dosimetric evaluation (4D-sCT-50%: 93.7±4.4%, 4D-sCT-ave: 94.3±4.7%, 3D-sCT: 92.6±5.2%). Local dose differences for CTV and lung were all within ±2.2%.

**Conclusion**: Despite lower image quality in individual 4D-sCT phases, the dosimetric accuracy of 4D-sCTs and 4D-sCT-ave is comparable to 3D-sCTs, suggesting clinical suitability for proton dose calculations in APT.

### P 146 - Patterns Of Practice for Adaptive and Real-Time Particle Therapy (POP-ART PT), Part I: intrafractional respiratory motion

#### Ye Zhang^1^, Petra Trnkova^2^, Toshiyuki Toshito^3^, Ben Heijmen^4^, Christian Richter^5^, Marianne Aznar^6^, Albertini Francesca and Alessandra Bolsi^1^, Juliane Daartz^7^, Jenny Bertholet^8^, Antje Knopf^1^

##### *^1^Paul Scherrer Institut, Center for Proton Therapy, Villigen-PSI, Switzerland*

*^2^Medical University of Vienna, Department of Radiation Oncology, Vienna, Austria*

*^3^Nagoya City University West Medical Center, Nagoya Proton Therapy Center, Nagoya, Japan*

*^4^Erasmus Medical Center, Department of Radiotherapy, Rotterdam, Netherlands*

*^5^Technische Universität Dresden- Helmholtz-Zentrum Dresden - Rossendorf, OncoRay – National Center for Radiation Research in Oncology- Faculty of Medicine and University Hospital Carl Gustav Carus, Dresden, Germany*

*^6^University of Manchester, Division of Cancer Sciences- Faculty of Biology- Medicine and Health, Manchester, United Kingdom*

*^7^Massachusetts General Hospital, Department of Radiation Oncology, Boston, USA*

*^8^Inselspital- Bern University Hospital, Division of Medical Radiation Physics and Department of Radiation Oncology, Bern, Switzerland*

Real-time respiratory motion management (RRMM, intra-fraction geometrical intervention) and Adaptive Particle Therapy (APT, inter-fraction adaptation of treatment plans) enable to account for anatomical variations and changes to optimize target coverage and organs-at-risk sparing. However, their current clinical implementation is unclear and expected to be highly heterogeneous. An institutional questionnaire, *Patterns Of Practice for Adaptive and Real-Time Particle Therapy (POP-ART-PT)*, was distributed between 2021/01-06 to evaluate current clinical practice and wishes and barriers of the implementation. Here, we summarise the international results on RRMM from 70 particle therapy centers in 17 countries. Response rate was 100% for Europe, 96% for Japan and 53% for USA. RRMM is used in 83% centres with 40% using both rescanning and active methods (figure1a). Of the 97% clinically operational centres, 62% apply rescanning and 16% indicated implementation plan in future (figure1b). 64% respondents use active RRMM for at least one tumour site (figure1c), with voluntary deep inspiration breath-hold and free-breathing expiration gating as the most common techniques. Most often used RRMM signals are external marker. 48% active RRMM users wish to increase or change their technique for tumour sites already treated with RRMM. 48% respondents wish to implement active RRMM for new tumour sites, of which 24% plan to enable it in 2-year. The main barriers are technical limitations and limited resources (figure2). RRMM with rescanning, active methods or both, has been implemented by majority PT, but substantial interest is shown to implement more active RRMM, requiring increased support to address technical limitations and enhanced resources.

### P 147 - Commissioning of a Monte Carlo algorithm for the proton gantry installed at MedAustron ion therapy center

#### Patrick Roisl^1^, Mansure Schafasand^1^, Veronika Kraus^2^, Michaela Daniel^1^, Alessio Elia^1^, Jhonnatan Osorio^1^, Ralf Dreindl^1^, Loïc Grevillot^1^, Markus Stock^1^, Antonio Carlino^1^

##### *^1^MedAustron Ion Therapy Center, Medical Physics, Wiener Neustadt, Austria*

*^2^Technical University of Vienna, Biomedical Engineering, Vienna, Austria*

In this work we report the results of dosimetric commissioning of the Monte Carlo algorithm MC5.3 available in the Treatment Planning System (TPS) RayStation RSv11B (RaySearch Laboratories, Sweden). The beam model reflects the scanned proton beam delivery at the gantry of MedAustron (MA). Measurements were performed with detectors and water phantoms positioned at different air gaps between the extractable snout and the isocenter including plans with range shifter (RaShi). For 1D/2D, integrated radial dose profiles as function of depth in water, lateral spot profiles in air and absorbed dose to water in reference conditions were measured. For 3D tests the complexity was increased from box-shaped fields in homogeneous phantoms to more complex clinical cases measured with 24 PinPoint ionization chambers (PTW, Freiburg) (figure 1). The TPS-predicted physical range (R80%) in water agreed within ±0.1mm with the measured R80% at isocenter. For the spot profiles, the deviation between measured and calculated full width at half maximum (FWHM) was below 4%. The validation for box-shaped plans in water showed an average global dose deviation within ±0.6% for isocentric setups with and without RaShi. Regarding the clinical cases, the average global dose deviations of were 0±0.6% and 1±0.6% for beams without and with RaShi respectively. In addition 2D dose distribution were acquired at different depths in RW3 with Octavius (PTW, Freiburg) (figure1) and compared to the TPS planned dose. Evaluation based on 3D global gamma index analysis (DD=2%, DTA= 2mm) showed an average pass rate of 94.7% (figure 2).

### P 148 - Development of modular shielding approach for proton therapy centers

#### Cynthia Keppel^1^, Pawel Ambrozewicz^2^

##### *^1^Thomas Jefferson National Accelerator Facility, Physics Division, Newport News, USA*

*^2^Thomas Jefferson National Accelerator Facility, Phyisc Division, Newport News, USA*

The design and coordination of concrete shielding is one of the most critical and complicated processes in proton therapy construction. It has been estimated that almost half of the construction cost of a proton therapy project is associated with the concrete construction requirements. It may be possible to largely bypass these obstacles with the use of transportable, modular, shielding that would also allow for efficient reconfiguration or decommissioning of proton therapy facilities. To this end, alternate shielding materials and approaches have been investigated and modeled using GEANT4, FLUKA, and benchmark measurements. Suitable alternative shielding materials to facilitate modular fills have been identified and novel shielding studies, leading up to a full center design, will be presented.

### P 149 - Dynamic evolution model of radionuclides induced in air at the Argentine Center for Proton Therapy

#### María Belén Alvarez^1^, Gustavo Santa Cruz^2^

##### *^1^Faculty of Engineering- Exact and Natural Sciences- Favaloro University.”, Medical physics engineering, Cuidad Autónoma de Buenos Aires, Argentina*

*^2^National Atomic Energy Commission, Nuclear medicine and radiotherapy., Cuidad Autónoma de Buenos Aires, Argentina*

In this work we will present a variety of topics that are related to radiation protection in the context of proton therapy, in particular the activation of air and production of radionuclides at the Argentine Center for Proton Therapy (CeArP). A simplified model of the controlled, restricted, and supervised areas of this center was developed with the purpose of modeling and analyzing air activation. This model was made using PHITS, a Monte Carlo radiation transport code capable of simulating primary and secondary particles, providing a dynamic projection of the radionuclides which are induced in the air in the areas of the cyclotron, treatment rooms and research laboratory. The induced activity was evaluated in a typical working day, considering the production of specific radionuclides induced by protons and secondary neutrons. Also, levels of concentration of these nuclides, and the interplay with the air injection and extraction system was considered to create a dynamic evolution model of the air activation. This concept shows a clear advantage compared with the approach that only considers the saturation activity, since it allows considering situations like HVAC ventilation system failures or over or underestimation of air renovations. The information obtained with this dynamic model was employed to calculate DACs (Derived Air Concentration) and compare these values with limits allowed in each relevant area. Finally, a two-dimensional simplified model of the air flux in CeArP was also developed to anticipate the possibility of studying the dynamic spatial distribution of radionuclides, e.g., studying possible stagnation regions.

### P 150 - Re-evaluation of beam energy and field size limits for clinical proton beam therapy and related machine requirements

#### Richard Amos^1^, Erik Traneus^2^, Stefan Schmidt^3^

##### *^1^Unversity College London, Medical Physics & Biomedical Engineering, London, United Kingdom*

*^2^RaySearch Laboratories AB, Physics, Stockholm, Sweden*

*^3^Particle Therapy Consultant, Physics, Overath, Germany*

Proton beam therapy (PBT) is an increasingly well-established radiotherapy modality with more than 100 facilities currently in operation worldwide and many more under development. However, the large financial investment required to build a new PBT facility, even for compact single-room systems, hampers more widespread adoption of this technology thus limiting access to cancer patients who may benefit. Since the 1990s the design of most clinical PBT systems has been based on a similar set of technical requirements that are generally recognized by the PBT community. This study challenges those requirements and attempts to re-establish a new baseline by examining: (a) the most relevant tumor/target locations for proton irradiation; (b) treatment planning techniques used for these indications; (c) related proton beam field parameters. Initial investigation suggests that a maximum proton beam energy between 150 and 180 MeV is sufficient to treat a large fraction of indications eligible for PBT. Furthermore, consideration is given to novel approaches to treatment planning and delivery with the aim of increasing the number of indications that may be treated with reduced system requirements. System design concepts are provided to transform a reduction in technical requirements for clinical application into equipment cost savings with the aim of further democratizing PBT for eligible cancer patients.

### P 151 - Proton beams for experimental radiobiological investigations at TOP-IMPLART facility

#### Monia Vadrucci^1^, Daniela Giovannini^2^, Maria Denise Astorino^1^, Giulia Bazzano^1^, Evaristo Cisbani^3^, Cinzia De Angelis^3^, Carmela Marino^2^, Luigi Picardi^1^, Simona Pazzaglia^2^, Concetta Ronsivalle^1^

##### *^1^ENEA, Development of Particle Accelerators and Medical Applications, Frascati RM- Italy, Italy*

*^2^ENEA, Division of Health Protection Technology, Rome, Italy*

*^3^ISS, Istituto Superiore di Sanità, Rome, Italy*

TOP-IMPLART (Intensity Modulated Proton Linear Accelerator for RadioTherapy) is a pulsed linear proton accelerator built in the ENEA-Frascati Research Center for proton therapy applications. The Project is led by ENEA with ISS and Regina Elena Hospital (IFO). In this framework, preliminary *in-vivo* experimental radiobiological investigations in mice were carried out. The modular structure of the LINAC accelerator allows to vary the delivered proton beam energy: 35 MeV and 55.5 MeV were set for these experiments. In the two-meters long beamline a scattering foil, a beam current diagnostic, a set of collimators, a dosimetry system based on EBT3 Gafchromic films, a diamond detector and on-line monitor ionization chambers and finally a removable range modulator wheel were arranged. Mice irradiations were performed in air. Heterozygous *Ptch1*^+/–^ mice are a well-established mouse model of medulloblastoma (MB), a pediatric cerebellar tumor. High incidence of MB is induced following irradiation of neonatal *Ptch1*^+/–^ mice. Focusing on radiation-quality effects, we want to compare the incidence of MB induced by irradiation of the cerebellum of 2 days old mice with protons with those elicited by cranial irradiation with the same dose of photons. In addition, a mouse model of MB allograft was also employed to compare cell killing induced by irradiation with the same dose of protons and photons. These results are relevant in view of the potential role of proton therapy in the treatment of pediatric MB. Experimental results, with a focus on aspects concerning the proton beam for mice exposure, will be presented and discussed.

### P 152 - A real-time transparent dosimeter and beam monitor for FLASH-RT and other advanced radiotherapy modalities

#### P.S. Friedman^1^, Vladimir Bashkirov^2^, Claudio Ferretti^3^, Alexander Kaipainen^3^, Daniel Levin^3^, Dale Litzenberg^4^, Nicholas Ristow^3^, Reinhard Schulte^2^

##### *^1^Integrated Sensors- LLC, Research & Development, Palm Beach Gardens- FL, USA*

*^2^Loma Linda University, Biomedical Engineering Sciences, Loma Linda- CA, USA*

*^3^University of Michigan, Physics, Ann Arbor- MI, USA*

*^4^University of Michigan, Radiation Oncology, Ann Arbor- MI, USA*

**Purpose**: To present a novel technology for precise, real-time beam monitoring based on a recently patented, transparent, large-area beam imaging detector. The technology provides a beam termination signal and ensures patient safety. In this presentation, we discuss applying this approach to particle FLASH-RT and other advanced RT modalities.

**Methods**: The FLASH-RT monitor (FRTM) utilizes a highly efficient, semi/micro-crystalline scintillator film. The scintillator signal is registered by a multi-camera machine vision system operating at 10,000 frames/second. The system allows continuous streaming and automated online analysis during treatment. The FRTM comprises an inline large-area scintillator detector (26x30 cm^2^). The scintillator films are practically transparent to the beam, and temporal sensitivity changes are corrected by a rapid internal calibration system. Images are analyzed every 100 μs for beam position, profile, and dosimetry.

**Results**: A proof-of-concept was demonstrated with 2D-position and beam profile spatial resolutions of <10 μm. The new scintillator material is very radiation hard as measured under accelerated aging conditions with an intense 10 nA, 5.4 MeV proton beam at the Michigan Ion Beam Laboratory. Accelerated test results will also be described from an intense 8 MeV electron beam linac source. Applications other than proton, electron and photon FLASH-RT include monitoring hypofractionated proton and carbon-ion SBRT, spatially-fractionated RT (microbeams and GRID), and BNCT.

**Conclusions**: The described FRTM provides a large-area, precise, beam profile analysis and dosimetry system with real-time continuous verification updated at 10^4^ Hz for all FLASH and other advanced external-beam RT modalities with a current focus as an enabling technology for electron and proton FLASH-RT.

### P 153 - Investigation of the dose-rate influence of a 1 MeV electron beam on hydroxyle radical yields

#### Antoine Danvin^1^, Abbas Nasreddine^2^, Catherine Galindo^1^, Philippe Peaupardin^1^, Lévana Gesson^1^, Marie Vanstalle^1^, Florent Kuntz^2^, Rémi Barillon^1^, Mirella Del Nero^1^, Quentin Raffy^1^

##### *^1^University of Strasbourg, Iphc - CNRS, Strasbourg, France*

*^2^Aerial-CRT, Aerial-CRT, Illkirch-Graffenstaden, France*

FLASH radiotherapy is a modality that gives high hopes, as it has a lower impact on healthy tissues compared to conventional radiotherapy [1]. Its principle is based on irradiation with very high dose rates (> 40 Gy/s) in a very short time. At very high dose rates, the reactive species formed in the track of an ionizing radiation can react with those formed in another track, which will have an impact on their yields. Presently, FLASH effect has been mostly studied with electrons. In our lab, we are developing a systematic study of the chemical effects of water radiolysis species on protein biomolecules. For this purpose, we are studying water radiolysis by measuring radiolytic yields G of its main species (HO^•^, e^-^_aq_, H_2_O_2_), that is the amount of species per unit of energy deposited. This presentation will focus on the measurements of the radiolytic yields of hydroxyl radical (HO^•^) with dose rates from 0.1 to 2000 Gy/s, under irradiation by 1 MeV electrons (Van De Graaff accelerator, Aerial-CRT). These measurements were performed with two different molecular scavenging probes (Coumarin-3-carboxylic acid and KBr/formate), and the kinetics of the radical were reconstructed from 370 ps to 1480 ns. A significant impact of the dose rate has been evidenced (*Figure 1*), with different thresholds depending on the scavenging time.

### P 154 - Oxygen depletion in ultra-high dose rates for protons and electrons: Measurements of G-value's dependency on dose rate

#### Jeannette Jansen^1^, Elke Beyreuther^2,3^, Florian-Emanuel Brack^3,4^, Leonhard Karsch^2,5^, Florian Kroll^3^, Josephine Metzges-Ng^3^, Jörg Pawelke^2,5^, Marvin Reimhold^3,4^, Ulrich Schramm^3,4^, Joao Seco^1,6^

##### *^1^German Cancer Research Center DKFZ, Biomedical Physics in Radiation Oncology E041, Heidelberg, Germany*

*^2^TU Dresden and Helmholtz-Zentrum Dresden–Rossendorf, OncoRay – National Center for Radiation Research in Oncology- Faculty of Medicine and University Hospital Carl Gustav Carus, Dresden, Germany*

*^3^Helmholtz-Zentrum Dresden – Rossendorf HZDR, Institute of Radiation Physics, Dresden, Germany*

*^4^Technische Universität, Dresden, Dresden, Germany*

*^5^Helmholtz-Zentrum Dresden – Rossendorf HZDR, Institute of Radiooncology - OncoRay, Dresden, Germany*

*^6^Ruprecht-Karls-University Heidelberg, Faculty of Physics and Astronomy, Heidelberg, Germany*

In FLASH radiotherapy (RT), a protective effect of healthy tissue was observed, while tumor control remains comparable to conventional RT. A possible explanation is the oxygen depletion hypothesis, where radiolysis of water/cytoplasm gives rise to radicals that react with dissolved O2. This would cause a reduction in O2, which results in a hypoxic target and thus a radio-protective effect. In a previous study (Jansen et al. 2021), we measured O2 depletion using an optical sensor in sealed water phantoms during irradiation at high dose rates (<300 Gy/s) for protons, carbon ions and photons and found the O2 depletion to be highly dependent on dose rate. In the study presented here, this experiment was conducted further to ultra-high dose rates (10^9 Gy/s) with protons at DRACO and electrons at ELBE, where also the impact of different pulse structures on O2 depletion in water was tested.

We were able to confirm the results of our previous study even at ultra-high dose rates and with electrons. Not enough O2 was depleted at clinical doses to explain a FLASH effect based on radiation-induced hypoxia. The amount of O2 depleted per dose depends on dose rate, and higher dose rates deplete slightly less O2, hinting towards radical-radical recombinations as a possible mechanism in FLASH. Furthermore, regarding pulse structures, our results showed that the effect of the average dose rate seemed to be dominant over the pulse dose rate with respect to radical production and O2 depletion.

### P 155 - A novel IMPT optimization method enforcing a minimum spot MU per energy layer for FLASH-RT

#### Pierre Lansonneur^1^, Tatu Leinonen^1^, Matti Ropo^1^, Lesley Rosa^2^, Anthony Magliari^2^, Michael Folkerts^2^

##### *^1^Varian Medical Systems, FLASH program, Helsinki, Finland*

*^2^Varian Medical Systems, FLASH program, Palo Alto, USA*

**Introduction:** In Intensity-Modulated Proton Therapy (IMPT), the monitor units (MU) of each spot are optimized to fulfill dosimetric constraints. In ProBeam system, the cyclotron beam current is moreover determined by the minimum MU of a spot in the energy layer [1]. Therefore, a high minimum MU/spot supports ultra-high dose-rate delivery (FLASH-RT) but may reduce significantly the number of spots, thus degrading the plan quality. We propose a method to optimize spot MUs above a user-defined value while preserving dosimetric constraints.

**Methods:** An optimization algorithm was implemented to enforce a minimum spot MU per energy layer. Single-energy-layer transmission proton IMPT plans were optimized for four lungs cases with varying PTV sizes. Following SBRT protocol [2], each plan was optimized to deliver 34 Gy in one fraction to the PTV while maintaining a minimum spot MU of 400 or 600. The PTV dose homogeneity and several dose metrics were characterized and compared to the one reached for conventional IMPT plans . The PBS dose rate [3] distribution was then calculated and evaluated using the 40 Gy/s volume coverage.

**Results:** All plans achieved clinically acceptable target uniformities (homogeneity indices from 1.04 to 1.13). The fraction of irradiated volume (Dose > 2 Gy) receiving at least 40 Gy/s was 89.5 % for lungs and 98 % for esophagus. The execution time of the algorithm was only two to three times longer than for conventional optimization.

**Conclusion**: This new optimization method will support the development of treatment planning for FLASH-RT clinical trials. [1] https://doi.org/10.3390/cancers13143549. [2] https://www.nrgoncology.org/Clinical-Trials/Protocol/rtog-0915?filter=rtog-0915. [3] https://doi.org/10.1002/mp.14456

### P 156 - Challenges and workarounds to deliver FLASH beams with a saturated monitor chamber

#### Haibo Lin^1,2^, Yunjie Yang^1^, Minglei Kang^1^, Alex Wei^1^, Sheng Huang^1^, Qing Chen^1^, Anna Zhai^1^, Francis Yu^1^, Michael Folkerts^3^, Chengyu Shi^1^

##### *^1^New York Proton Center, Medical Physics, New York, USA*

*^2^Memorial Sloan Kettering Cancer Center, Medical Physics, New York, USA*

*^3^Varian Medical Systems Inc., FLASH Research, Palo Alto, USA*

**Purpose**: This study presents workarounds to overcome challenges when operating a ProBeam system in FLASH research-only mode for pre-clinical experiments. The challenges arise due to ultra-high beam currents (>20nA) saturating the machine's monitoring chamber (MC).

**Methods**: Our FLASH experience is based on performance in one horizontal-beamline research room. We discuss the top three issues impacting performance and safety caused by the saturated MC: 1. under-reporting of delivered monitor units (MUs); 2. Machine output variations due to beam current fluctuation; 3. Radiation shielding and activation of devices in the radiation field. We present our experience and recommendations for each major concern as a reference for other ProBeam centers conducting FLASH pre-clinical research and facing the same challenges.

**Results**: The system delivered more doses than requested due to MC saturation. This dose deviation depends on beam current and MU/spot. The relationship between planned and delivered doses is established to determine an MU scaling factor. The delivery is repeated 2-3 times, and the measured doses are averaged to fine-tune MU scaling factor, minimizing the impact of output variations to achieve an accurate dose delivery. Hourly and daily limits in nA*second are recommended based on conservative evaluation of the radiation survey and radiation level from activation.

**Conclusions**: It is critical to understand the ProBeam system behavior and safety concerns when operating in FLASH research-only mode with a saturated MC. Institutional protocols and workflows should be developed to address each issue before using the system for research and pre-clinical experiments.

### P 157 - A novel optimization framework for FLASH proton therapy based on a cyclotron accelerator proton therapy system

#### Gang Liu^1,2^, Lewei zhao^2^, Xiaoqiang Li^2^, Shuyang Dai^3^, Xiliang Lu^3^, Xuanfeng Ding^2^

##### *^1^Huazhong University of Science and Technology, Cancer Center Union Hospital Tongji Medical College, Wuhan, China*

*^2^Beaumont Health System, Radiation Oncology, Royal oak, USA*

*^3^Wuhan University, School of Mathematics and Statistics, Wuhan, China*

**Purpose:**Proton Pencil Beam Scanning(PBS) is an attractive solution to realize the advantageous normal tissue sparing elucidated from FLASH high dose rates. However, the treatment planning system(TPS) only optimizes the dose distribution at the present stage without considering the dose rate. This study aims to develop a novel platform optimizing the dose distribution and dose rate sequentially based on the IBA Proteus machine.

**Methods:**IBA Proteus machine's spot spill efficiency depends on the energy layer and cyclotron beam current. In order to achieve FLASH dose rate(40Gy/s), the energy layer and cyclotron beam current can be adjusted. A two-fields intensity-modulated proton therapy(IMPT) plan with multiple energies was optimized to acquire optimal dose distribution. Then the strategy of dose rate optimization was realized by constraint dose volume on dose-average dose rate considering spot spill time only to generate optimal cyclotron beam current per spot based on the IMPT plan. This new optimization concept was implemented on an open-source proton planning platform(matRad). A prostate case was utilized for testing purposes.

**Result:**The dose-averaged dose rate(40Gy/s) cannot be achieved to FLASH dose rate in IMPT using the current clinical beam current. While with the same dose distribution, the new optimization framework improves dose-averaged dose rate of V*r*>40Gy/s to 95%,98%,75%,55% in PTV,prostate,rectum and bladder,respectively. The optimal cyclotron beam current per spot is simultaneously generated(Fig1).

**Conclusion:**The study developed a new framework to achieve an ultra-high FLASH dose rate based on the IBA Proteus machine through dose distribution and dose-average dose rate optimization, laying the foundation for flash proton therapy TPS platform.

### P 158 - Creating IMPT equivalent FLASH proton therapy treatment plans

#### Nathalie Lovgren^1^, Kristoffer Petersson^1^, Rudi Labarbe^2^, Lucian Hotoiu^2^, Ingrid Fagerström Kristensen^3^

##### *^1^University of Oxford, Oncology, Oxford, United Kingdom*

*^2^Ion Beam Applications SA IBA, IBA Research, Louvain-la-Neuve, Belgium*

*^3^Skåne University Hospital, Oncology, Lund, Sweden*

FLASH proton therapy (FLASH-PT) aims to use beams of ultra-high dose rates ( ≥ 40 Gy/s) to induce a normal tissue (FLASH) sparing effect whilst maintaining the anti-tumour effectiveness of conventional PT. Compared to conventional PT, FLASH-PT differ in the beam delivery, dose rate, and fractionation schemes used. Hence, no guidelines exist for the clinical implementation or treatment planning of this technique. This study aims to compare the *in silico* results produced by a novel FLASH-PT treatment planning system (TPS) against plans for IMPT. FLASH-PT and IMPT treatment plans were created using a research version of the MIROpt TPS, developed by Ion Beam Applications SA from the open source version of UCLouvain. The FLASH-PT beam delivery involved monoenergetic spot-scanned protons traversing through a conformal energy filter, a range shifter, and an aperture. Plans were created for nine patient cases of bone (3), brain (3), and lung (3) metastases, all previously clinically treated with 3DCRT or VMAT. A dose rate constraint of ≥ 40 Gy/s was included in the plan optimisation. Dose volume histograms, boxplots, and the Wilcoxon Rank Sum Test (p < 0.05) were used to compare the FLASH-PT plans to the optimised IMPT plans. High-speed conformal FLASH-PT plans were produced which satisfied the FLASH dose rate constraint whilst also achieving equivalent dose distributions as IMPT plans. Future work involves the verification of the calculated dose against the physical dose delivered to confirm the safety, accuracy, and feasibility of conformal FLASH-PT.

### P 159 - A novel energy selection system to achieve FLASH dose rates even for low-energy beams in cyclotron-based proton therapy facilities

#### Vivek Maradia^1,2^, David Meer^1^, Damien Charles Weber^1,3,4^, Antony John Lomax^1,2^, Serena Psoroulas^1^

##### *^1^Paul Scherrer Institut, Center for Proton Therapy, Villigen, Switzerland*

*^2^ETH Zurich, Department of Physics, Zurich, Switzerland*

*^3^University Hospital Zurich, Department of radiation oncology, Zurich, Switzerland*

*^4^University Hospital, Department of radiation oncology, Bern, Switzerland*

In proton therapy, the potential of ultra-high (FLASH) dose-rate for cancer treatment is being explored due to its potential for enhancing biological effect, faster hypo-fractionated treatments, and efficient motion mitigation. However, energy degradation for FLASH irradiations using cyclotrons is far from optimal; if performed upstream (as common clinically, using a degrader plus an energy/momentum selection system, or ESS), very large losses result, while if performed downstream, just before the patient, the beam width is substantially compromised. In both cases, the net effect is a loss of dose rate, either by transmission loss or by beam scattering, making the delivery of FLASH dose rates challenging for lower energies. To avoid downstream energy degradation and its effects on the beam shape, we propose using an ESS without momentum selection slits, which compensates the momentum spread in the beam without introducing significant beam loss. In Monte Carlo (BDSIM) simulations, we found that this new ESS concept, along with matched beam optics, results in a significantly increased overall transmission (Fig.1) compared to current installations, even achieving 10% beam transmission, from the cyclotron to isocenter, for a 70 MeV beam. This corresponds to a current of 80 nA at the isocenter assuming 800nA extracted from the cyclotron. At Bragg peak (in water), we achieved dose-rate of 952 Gy/s and 2105 Gy/s for 70 MeV and 230 MeV beams respectively (Fig.2). Such a system could easily be implemented in future cyclotron-based particle facilities to increase dose rates for efficient motion mitigation, hypofractionation and FLASH experiments alike.

### P 160 - Fit for FLASH: Are the radiation protection provisions of existing centres suitable for ultra-high dose rate FLASH proton beams?

#### Michael Taylor^1^, Mark Hardy^2^, Ashworth David^3^, Michael Merchant^1^, Ranald MacKay^3^, Karen Kirkby^1^

##### *^1^The University of Manchester, Division of Cancer Sciences, Manchester, United Kingdom*

*^2^The Shrewsbury and Telford NHS Foundation Trust, Radiotherapy, Shrewsbury, United Kingdom*

*^3^The Christie NHS Foundation Trust, Medical Physics and Engineering, Manchester, United Kingdom*

The use of ultra-high dose rate beams to investigate, in vitro, the normal tissue sparing effects of FLASH has been adopted by many proton beam therapy research facilities worldwide. This research has been accelerated due to the ability of current clinical proton therapy equipment to deliver the required FLASH dose rates. The radiation protection provision for these existing facilities, however, was designed for beams delivering dose rates two orders of magnitude lower than those required for FLASH. This work aims to investigate the radiation protection measures in place for the Christie proton beam research facility to ascertain whether or not they were “Fit for FLASH”. Three aspects of radiation protection are being considered: shielding provision, activation and accident scenarios. Dose rate surveys along with state-of-the-art Monte-Carlo radiation transport simulations are being used to assess the current provisions and inform any modifications required. It is hoped that the methodology developed could be translated to other centres to assess their fitness for FLASH. An overview of the measurements undertaken as well as the results and radiation protection modifications required for FLASH beams will be presented.

### P 161 - Evaluation of proton pencil beam scanning (PBS) plan quality and delivery time as a function of spot and layer spacing

#### Jailan Alshaikhi^1,2,3^, Gary Royle^2^, Derek D'Souza^3^, Richard Amos^2^

##### *^1^Saudi Proton Therapy Center, Medical Physics Department- King Fahad Medical City, Riyadh, Saudi Arabia*

*^2^University College London, Department of Medical Physics and Biomedical Engineering, London, United Kingdom*

*^3^University College London Hospital, Department of Radiotherapy Physics, London, United Kingdom*

**Purpose/Objective:** To evaluate variable spot- and layer-spacing on proton PBS plan quality and delivery time.

**Materials and methods:** Plans to 10x10x10cm homogeneous target calculated using Eclipse v13.5, commissioned with IBA beam data. All plans used 2.5mm calculation grid and optimised for a *Rx* of 100% ≤ *Rx* ≤ 102% to entire volume. Layer-spacing (Bragg peak 90-90% of most proximal target region) varied 3.7mm-10mm with 5mm spot-spacing. Spot-spacing varied 3mm-10mm with constant layer-spacing of 7.6mm (4 x σ of next highest energy). Numbers of spots and layers were extracted from DICOM files using a MATLAB script. For all plans, times taken for calculation and optimisation were recorded, and dose homogeneity was evaluated (D_1_-D_99_, D_2_-D_98_, and D_5_-D_95_). Treatment delivery times for each plan were estimated using Eq.1:



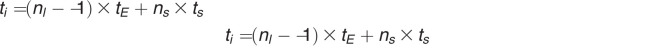




where: *t_i_* is estimated delivery time;* n_l _*is the number of layers; *t_E_* is layer switching time, (approx 1.4 s (averaged across all energies));* n_s_* is number of spots; *t_s_* time to deliver a single spot.

**Results:** See Table 1 and Figure 1.

**Conclusions:** Reducing spot- and layer-spacing in proton PBS plans increases number of spots to be delivered, resulting in improved homogeneity but increased delivery time. Layer-spacing ≥ 5mm resulted in dose inhomogeneity. Ideally, spot-spacing would vary as a function of depth to account for multiple Coulomb scattering, however, where a constant spot-spacing is required, 5mm was found to maintain homogeneity. As compromise between plan quality and delivery time, both spot- and layer-spacing of 5mm is recommended for proton PBS plans.

### P 162 - Timing and energy discrimination of prompt gamma signatures for neutron capture dose quantification in NCEPT

#### Andrew Chacon^1^, Kielly Marissa^2^, Caracciolo Anita^3^, Luca Buonanno^3^, Ilenia D'Adda^3^, Anatoly Rosenfeld^2^, Susanna Guatelli^2^, Marco Carminati^3^, Carlo Fiorini^3^, Mitra Safavi-Naeini^1^

##### *^1^Australian Nuclear Science and Technology Organisation, Human Health, Lucas Heights, Australia*

*^2^University of Wollongong, Centre for Medical Radiation Physics CMRP, Wollongong, Australia*

*^3^Politecnico di Milano- Milano, Electronics and Information Technologies, Milano, Italy*

Neutron Capture Enhanced Particle Therapy (NCEPT) boosts the effectiveness of particle therapy by capturing thermal neutrons produced by beam-target nuclear interactions in and around the treatment site, using tumour-specific 10B or 157Gd-based neutron capture agents. Neutron captures release high-LET secondary particles together with gamma photons with energies of 478keV or one of several energies up to 7.94MeV, for 10B and 157Gd, respectively. A key requirement for NCEPT's translation is the development of in-vivo dosimetry techniques which can measure both the direct ion dose and neutron capture dose. In this work, we report signatures which can be used to discriminate between photons resulting from neutron capture and those originating from other processes. Monte Carlo simulations into timing and energy thresholds for discrimination of prompt gamma photons resulting from thermal neutron capture during NCEPT were conducted. Three simulated 300mm cubic PMMA targets were irradiated by 4He or 12C beams with a 60mm spread-out Bragg peak (SOBP); one target is homogeneous while the others include 10mm cubic neutron capture inserts of pure 10B or 157Gd. The arrival times of photons and neutrons entering a simulated 50mm cubic ideal detector were recorded. A temporal mask of 50-60ns was found to be optimal for maximising the discrimination of the photons resulting from the neutron capture by boron and gadolinium. Thermal neutron shielding materials were investigated, and detections meeting the acceptance criteria were classified as true/false positives, depending on their origin with highest ratio of true/false were cadmium-shielded CdTe and boron-shielded LSO, respectively.

### P 163 - Comparison of strategies of dose reconstruction from prompt-gamma radiation in proton therapy

#### Beatrice Foglia^1^, Chiara Gianoli^1^, Thomas Bortfeld^2^, Katia Parodi^1^, Marco Pinto^1^

##### *^1^Ludwig-Maximilians-Universität LMU Munich, Experimental Physics - Medical Physics, Garching b. München, Germany*

*^2^Massachusetts General Hospital MGH Boston, Radiation Oncology, Boston MA, USA*

In proton therapy, uncertainties can have a considerable impact, so a reconstruction of the delivered dose is desirable as verification method. The distribution of prompt gamma (PG), emitted after nuclear interactions of proton beams with the tissue, could be exploited for *in vivo* verification. To tackle the reconstruction of the actual delivered dose from a distribution of detected secondary particles, different strategies have been proposed. Very promising are the initially investigated analytical deconvolution method [1], the evolutionary algorithm [2][3] and the maximum likelihood expectation maximization (ML-EM) algorithm [4][5]. These techniques are based on the forward filtering approach, firstly developed by Parodi and Bortfeld [6] for positron emission tomography monitoring and recently extended to PG monitoring by Pinto et al. [7]. These methods have mostly been applied in order to reconstruct the dose from positron emitters distributions. Only the evolutionary algorithm has been applied on PG distributions so far, and only on homogeneous targets [2]. This work addresses the comparison of these dose reconstruction approaches as part of their applicability to real-time adaptive particle therapy. Dose reconstruction accuracy, computation time and integration into clinical workflow will be considered based on *in silico* studies using both homogeneous and heterogeneous phantoms, and patient data. This work is performed as part of the RAPTOR project, funded by the EU's Horizon 2020 MSCA, Grant Agreement No. 955956. 1.Remmele et al. 2011, Phys.Med.Biol.56. 2.Schumann et al. 2016, Phys.Med.Biol.61. 3.Hofmann et al. 2019, Phys.Med.Biol.644.Masuda et al. 2019, . Phys.Med.Biol.64. 5.Masuda et al. 2020, Phys.Med.Biol.65. 6.Parodi and Bortfeld 2006, Phys.Med.Biol.51. 7.Pinto et al. 2020, Phys.Med.Biol.65

### P 164 - Measurement of secondary particles and radiochemical damage associated in hadrontherapy

#### Lévana Gesson^1^, Antoine Danvin^1^, Nicolas Arbor^1^, Clément Corneille^1^, Christian Finck^1^, Stephane Higueret^1^, Daniel Husson^1^, The-Duc Le^1^, Quentin Raffy^1^, Marie Vanstalle^1^

##### ^1^University of Strasbourg, Iphc - CNRS, Strasbourg, France

Hadrontherapy exhibits a better dose conformation compared to conventional therapy. However, nuclear fragmentation between the primary beam and the target volume leads to the production of lighter particles, which can contribute to unwanted dose inside the healthy tissues. Therefore, measurements of these secondary particles and their effects on human tissues are necessary. One crucial step in the understanding of biological response to ions treatment is the study of detailed processes occurring in the biomolecules composing living cells. We propose here an innovative interdisciplinary approach, combining physical chemistry and nuclear physics study of primary ions and their secondary particles. Both physical and chemical processes will be measured in order to better characterize the peripheral dose in particle therapy, and to assess Monte Carlo codes, such as Geant4 and Geant4-DNA. Neutrons will be measured with a recoil proton telescope, and the detection of charged particles will be performed with a ΔE-E telescope, made of a thin-plastic scintillator and a cerium bromide crystal (Fig 1.a). The irradiation of biomolecules is performed thanks to flow-through cells, to circulate the solutions in the beam. In this work, we will present an overview of this project, as well as preliminary results obtained at CAL (Nice, France) and Cyrcé (Strasbourg, France) with proton beams of 62 and 25 MeV. The setup of the CAL experiment is presented on Figure 1.

### P 165 - Robustness in proton arc treatments for head and neck cancer patients: impact of gantry angle spacing and number of revolutions

#### Otte Marthin^1^, Viktor Wase^1^, Lars Glimelius^1^, Rasmus Bokrantz^1^, Björn Andersson^1^, Albin Fredriksson^1^, Bas de Jong^2^, Erik W Korevaar^2^, Stefan Both^2^, Erik Engwall^1^

##### *^1^RaySearch Laboratories, Research & Development, Stockholm, Sweden*

*^2^University Medical Center Groningen, Department of Radiation Oncology, Groningen, Netherlands*

Plan robustness might be a challenge in proton arc therapy since the spots are distributed over multiple directions. We employ an Early Layer and Spot Assignment (ELSA) algorithm prior to robust spot weight optimization to design multi-revolutional arc plans for six H&N patients to assess the impact of gantry angle spacing and number of revolutions on robustness. The arcs are modelled by discrete gantry angles, each angle being assigned no more than one energy layer. Seven arc plans are created per patient with the number of revolutions between 1 and 3 and varying gantry angle spacing. The plans are optimized with the same objectives as the clinical IMPT plans. Figure 1 shows robustness evaluation on the planning CT (3% density, 3 mm setup uncertainty) and recomputations on repeated CTs. For a constant number of energy layers distributed over the revolutions, 360 energy layers give better robustness than 180 layers. Spreading a constant number of layers over several revolutions, i.e., increasing the gantry angle spacing, negatively impacts the robustness. If the gantry angle spacing is instead kept constant, robustness increases with the number of revolutions. All arc plans give increased sparing of OARs compared to IMPT, with drastic improvements in the brainstem and spinal cord (see Table 1). There is no clear trend between OAR sparing and multi-revolutions. In conclusion, robustness in proton arc therapy is a complex topic affected by several factors such as gantry angle spacing and energy layers in non-obvious manners.

### P 166 - A novel proton RBE model based on alternative metrics to LET

#### Fredrik Kalholm^1,2^, Leszek Grzanka^3^, Iuliana Toma-Dasu^1,2^, Niels Bassler^4,5,6^

##### *^1^Stockholm University, Medical Radiation Physics, Stockholm, Sweden*

*^2^Karolinska Institute, Department of Oncology and Pathology- Medical Radiation Physics, Stockholm, Sweden*

*^3^Polish Academy of Sciences, Institute of Nuclear Physics, Krakow, Poland*

*^4^Aarhus University Hospital, Danish Centre for Particle Therapy, Aarhus, Denmark*

*^5^Aarhus University Hospital, Department of Experimental Clinical Oncology, Aarhus, Denmark*

*^6^Aarhus University, Department of Clinical Medicine, Aarhus, Denmark*

Several models for proton RBE have been proposed for evaluating and/or optimizing the treatment plans in proton radiotherapy, motivated by observed unexpected toxicities near proton track ends. Typically, dose averaged linear energy transfer (LETd) is used as radiation quality descriptor in these models, but the method for calculating the LETd is not always consistent, potentially impacting the corresponding RBE value. This study compares consistently calculated LETd with other quantities as input variables for a novel phenomenological RBE model and evaluates their performances. Experimental set-ups of recent high-throughput *in vitro* cell survival studies for proton RBE determination were simulated using the SHIELD-HIT12A Monte Carlo particle transport code. Together with LET, z*^2/β^2, here called *effective* Q (Qeff) and Q are scored. Each quantity is calculated using the dose and track averaging methods, while the scoring includes all secondaries, all protons or only primaries. A phenomenological linear-quadratic based RBE model is applied to the *in vitro* data with the various beam quality quantities used as input variables and the goodness of fit was determined and compared. Qeff- and Q- outperform LET-based models with a statistically significant margin, with the best linear models having a relative root-mean-square-error (RMSE) for RBE 2Gy ± one standard error of 2.83 ± 0.07 (Qeff, d, primary), compared to 3.39 ± 0.08 for LETd, protons, indicating that a more accurate phenomenological RBE model for protons might be achieved by using of Q or Qeff as input parameter.

### P 167 - Neutron shielding strategies for dose quantification in Neutron Capture Enhanced Particle Therapy

#### Marissa Kielly^1^, Andrew Chacon^2^, Daniel Franklin^3^, Anita Caracciolo^4^, Ilenia D'Adda^4^, Anatoly Rosenfeld^1^, Susanna Guatelli^1^, Marco Carminati^4^, Carlo Fiorini^4^, Mitra Safavi-Naeini^2^

##### *^1^University of Wollongong, Centre for Medical Radiation Physics, Wollongong, Australia*

*^2^Australian Nuclear Science and Technology Organisation, NST Human Health, Lucas Heights, Australia*

*^3^University of Technology Sydney, Faculty of Engineering & IT, Sydney, Australia*

*^4^Politecnico di Milano, Department of Electronics and Information Technologies, Milano, Italy*

Neutron Capture Enhanced Particle Therapy (NCEPT) boosts the effectiveness of particle therapy by capturing thermal neutrons produced by beam-target interactions in and around the treatment site, using tumour-specific 10B or 157Gd-based neutron capture agents. Neutron capture by these isotopes results in the production of high-LET secondary particles and prompt gamma photons of 478 keV and 7.94 MeV for 10B and 157Gd, respectively and if quantified, a measure of the neutron capture dose. However, neutrons present in the mixed radiation field may contribute to false positive counts, which arrive within the same timing and energy windows as these photons, but are not the result of neutron capture. As such, neutron shielding or discrimination is necessary. A Geant4 simulation consisting of a shielding layer of variable thickness and a detection layer of air has been constructed. Following the irradiation of this layer by a neutron beam with a spectrum as seen in NCEPT, the neutron fluence is recorded and changes with thickness and material are investigated through a MATLAB optimisation process. Front and side shielding strategies are proposed for the two neutron capture agents, which require different materials due to the limitations of secondary photon emission creating false positives. These strategies were then tested via an additional simulation of a full detection system, with the calculation of the true:false positive ratio (RTF) at the detector.

### P 168 - A novel approach for adapting material decomposition acquisitions to DECT calibrations in proton therapy planning

#### David Viar Hernandez^1^, Juan Antonio Vera Sanchez^2^, Borja Rodrigez-Vila^1^, Fernando Cerron^2^, Juan Castro Novais^2^, Juan Maria Perez Moreno^2^, Norberto Malpica^1^, Angel Torrado-Carvajal^1^, Alejandro Mazal^2^

##### *^1^Universidad Rey Juan Carlos, Medical Image Analysis and Biometry Laboratory, Madrid, Spain*

*^2^Centro de Protonterapia Quironsalud, Servicio de fisica medica, Madrid, Spain*

Most dual energy (DECT) calibration methods for proton therapy rely on Hounsfield Units Low (HU_L_) and High (HU_H_) images to derive Z-effective and electron densitymaps. In some cases, these low and high images are not accessible when using single source DECT (ssDECT), since only decomposition maps (DM) are provided by the scanner. Although some ssDECT approaches can generate virtual monochromatic images mimicking both single energy (SECT) acquisitions for visualization, their use for calibration is still under research. Therefore, we study the reliability of a novel method for direct computation of HU images based on the DM pair WATER-IODINE. The computational method presented is based on the theoretical equation cited in report 291 of the American Association of Physicists in Medicine (AAPM). This equation is solved by acquiring two sets of SECT data (E_L_ = 80kvp and E_H_ = 140kvp) and one DECT. The acquisitions were performed using a GE REVOLUTION CT scanner, with SECT and ssDECT protocols, and a GAMMEX multi-energy CT phantom conformed by tissue surrogates.



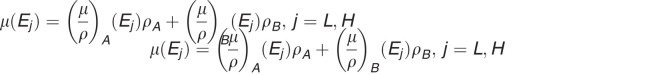




After calibration, HU_H_ and HU_L_ images (Fig.1b) are derived from new DM pairs (Fig.1a) and used to derive their corresponding Z-effective and electron density-maps (Fig.1c). This methodology was evaluated using the GAMMEX phantom. Mean±standard-deviation was computed for each tissue surrogate and compared to the ground truth based on the SECT images. Our results show no significant differences (p<0.001) for water and iodine surrogates between the reference and images derived from the DM. However, differences for the other tissues exist, requiring further improvements.

### P 169 - Study of thread divergence in GPU-based Monte Carlo particle transport simulations

#### Costanza Panaino^1^, Hok Seum Wan Chan Tseung^1^

##### ^1^Mayo Clinic, Radiation Oncology, Rochester, USA

The efficiency of GPU-based Monte Carlo particle transport (GPU-MCPT) simulations for dose calculations is limited by thread divergence (TD). We discuss the main TD sources in GPU-MCPT and propose a mitigation strategy. At the core of all MCPT simulations there is a version of the iterative loop shown in Fig.1a. The four dominant TD sources in this loop were identified and listed in Fig.1b (A to D). A bare-bones proton GPU-MCPT was built and modified to implement the TD sources, individually and combined. The computational impact of TD sources were studied as a function of branching fraction (BF), arithmetic complexity and density, particle energy and GPU memory type. To mitigate the effects of TD, the GPU-MCPT kernel is split into smaller, dedicated kernels to process GPU threads with identical branching path. As an example, Fig.2 shows the combined effects of sources A and C on GPU-MCPT computation time, as a function of the BF for both cases (see caption for details of their implementations). Typically, for any single source the calculation time saturates quickly with increasing BF. The processing of a 100 MeV secondary by the same GPU thread can greatly slow down the simulation. The dashed curves demonstrate the speed-ups achieved using our control strategy. We have identified TD sources generally present in GPU-MCPT simulations, and devised methods to systematically study and compensate for them. This work paves the way to more optimal GPU-MCPT deployments.

### P 170 - Dependence of microdosimetric quantities on the cell nucleus size and placement for radiopharmaceutical alpha emitters.

#### Daniel Suarez-Garcia^1^, Miguel Antonio Cortes-Giraldo^1^, Alejandro Bertolet^2^, Alejandro Carabe-Fernandez^3^

##### *^1^Universidad de Sevilla, Department of Atomic- Molecular and Nuclear Physics, Sevilla, Spain*

*^2^Massachusetts General Hospital and Harvard Medical School, Department of Radiation Oncology, Boston, USA*

*^3^Hampton University, Hampton University Proton Therapy Institute, Hampton, USA*

Radiopharmaceutical therapy (RPT) is based on the use of radiolabeled agents affine to antigens overexpressed in tumor cell environments. This type of treatment has the potential to improve outcomes for oncologic patients due to its ability to concentrate radiation in a small environment around the tumor. This phenomenon is based on the much higher linear energy transfer (LET) of the alphas emitted together with their considerably shorter range as compared to beta emissions. In this work, we studied how the microdosimetric quantities are affected by the displacement of the cell nucleus and variations in its radius. Interestingly, microdosimetry can help in the assessment of the biological effect of these particles to fully take advantage of the features mentioned above. Previously, an analytic algorithm was developed upon which microdosimetric quantities (yF, yD, zF, zD) were calculated for spherical cells whose membrane acted as uniform source [Bertoletet al. Radiat. Res. (2020)]. Besides exploring the influence of the eccentricity and radius of the nucleus, the analytic model was tested under these conditions. To calculate the distribution of these microdosimetric quantities, Monte Carlo simulations were run with Geant4-DNA. Our preliminary results (Figures 1–2) show that the microdosimetry quantities are not affected significantly by the cell nucleus position, while the dependence on the nucleus radius is clearly more significant. The analytical method agrees reasonably well with the Geant4 simulations.

### P 171 - Proton arc therapy with static-gantry spread-energy-layer optimization; An alternative to interstitial HDR brachytherapy for gynecologic cancer with parametrial/pelvic sidewall extension

#### Byongyong Yi^1^, Sina Mossahebi^1^, Arezoo Modiri^1^, Elizabeth Nichols^1^, Mariana Guerrero^1^, Narrottam Lamichhane^1^, Pranshu Mohindra^1^

##### ^1^University of Maryland School of Medicine, Radiation Oncology, Baltimore, USA

**Purpose:** To investigate whether proton arc therapy plans with static-gantry and spread-energy-layer optimization (SSO) are comparable to those of interstitial high-dose-rate (iHDR) brachytherapy for patients with gynecologic cancer with disease extension to parametrial/pelvic sidewall.

**Methods**: Plans of 10 patients previously treated with iHDR brachytherapy for high-risk clinical target volumes (HRCTV) (lateral dimensions, 4.2–5.6 cm) were selected and compared to SSO plans. Nine to 11 static proton beams, evenly spaced at ∼30° intervals, were used to optimize a plan with static-gantry beams. No beams were assigned to anterior and posterior directions to avoid beams directed to the bladder or rectum. After optimization, each energy layer was split into 1° intervals to create a proton arc plan.

**Results:** HRCTV coverage of the SSO plans was comparable to that of the iHDR plans, with V150% showing no statistical differences. The V100%, V90% and D100 of SSO plans were higher than those of the iHDR plans and the differences are statistically significant *(P* < 0.05) for all parameters. In a SSO 3-mm/3.5% robustness analysis, the worst scenario V100% decreased by 5%. The D_2cc_ of bladder, rectum, and small bowels in the SSO plans were considerably lower than those in iHDR plans (by 17.4%, 35.2%, and 65.6%, respectively [*P <* 0.05 for all OARs]).

**Conclusion:** SSO plans were dosimetrically superior to those with HDR brachytherapy with interstitial needles for locally advanced gynecologic cancer with parametrial/pelvic sidewall disease extension. Dosimetrically, SSO provides a noninvasive alternative to iHDR brachytherapy with a superior dosimetric profile.

### P 172 - Characterization of the mechanical and radiation isocenter of a proton gantry

#### Alessio Elia^1^, Alexander Ableitinger^1^, Tibor Madar^1^, Lisa Hartl^2^, Markus Stock^1^, Loïc Grevillot^1^

##### *^1^EBG MedAustron GmbH, Medical Physics, Wiener Neustadt, Austria*

*^2^University of Technology, Institute of applied physics, Vienna, Austria*

**Introduction:** Proton rotating gantries with active scanning (PBS) always challenge the beam position delivery accuracy. The variability in space of the mechanical as well as of the radiation isocenter is difficult to control as they both vary with the gantry angle. Typical dosimetric equipment provides useful insights in this regard. However, the detector setup itself might still hide important gantry angle dependencies, especially when directly attached at the rotating gantry nozzle. In this study, we propose an exhaustive characterization of the pencil beam position at isocenter considering both mechanical and dosimetric input.

**Material and methods:** First, by means of EBT3 gafchromic films, we assessed the signal response stability as a function of gantry angle for the Lynx (IBA-dosimetry) detector. Second, we deeply characterized the mechanical isocenter movements as a function of gantry angle by means of laser tracker AT402 (Leica) measurements. The Lynx alignment with respect to the room isocenter was then adjusted accordingly. Eventually, we characterized the radiation isocenter at different gantry angle via Lynx measurements. An independent verification was performed with an XRV-124 (Logos system). The Winston-Lutz test was also taken into consideration.

**Results:** No gantry angle dependency is found in the Lynx response. The laser tracker provides mechanical correction tables up to 1.8mm (see Figure 1). The accuracy of the beam delivery in position is found always within 1mm (see Figure 2). The Winston-Lutz test provide results within 1 mm.

**Conclusion:** A proton position accuracy delivery is ensured within 1 mm for the rotating gantry room.

### P 173 - Development of in-house QA trending software and long-term performance stability of TG-224 compliant daily QA program in modern proton therapy

#### Mauricio Acosta^1^, Andrew Wroe^1^, Jaafar Bennouna^1^, Daniel Cho^1^, Len Coutinho^1^, Edgar Gelover-Reyes^1^, Jerry George^1^, Jen Yu^1^, Alonso N. Gutiérrez^1^

##### ^1^Miami Cancer Institute, Radiation Oncology, Miami, USA

**Purpose**: This is a systematic review of the daily QA data collected at our institution over a 4-year period. The goal is to provide benchmark performance data of a modern pencil beam scanning system and insight into potential QA program improvements.

**Methods**: The daily QA program is based on the recommendations of AAPM TG-224 using a setup previously published. The dosimetry portion utilizes an IBA Lynx detector coupled with a Sphinx QA phantom and parallel plate (PPC05) ion chamber. The imaging portion uses the MIMI/HexaCheck phantoms for kV/kV and CBCT as well as 6DoF couch validation. Data is analyzed and recorded using myQA (Figure 1). Additionally, our center has developed standalone Daily QA Dashboard for instant visualization of results and data trends. The Daily QA Dashboard is a Flask web application written using D3 JavaScript library and Python.

**Results**: Parameters evaluated as part of the daily QA program are described in Figure 1. More than 2000 measurements collected over 4 years demonstrates the stability of our proton therapy system is well-within tolerances set forth by AAPM TG-224 for all daily dosimetric, imaging, and mechanical metrics recommended. To assess temporal performance, the Daily QA Dashboard (Figure 2) shows the color-encoded results of every measurement for a specific gantry donut chart. An interactive trending scatterplot updates given user selection.

**Conclusions**: The daily QA program at our center shows stable performance of our system based on TG-224 tolerances. An in-house data visualization and trending tool is invaluable to identify deviations of measured parameters.

### P 174 - A highly-efficient planning and delivery method for proton PBS spatially fractionated radiation therapy GRID

#### Jen Yu^1^, Andrew Wroe^1^, Rupesh Kotecha^1^, Alonso N. Gutiérrez^1^

##### ^1^Miami Cancer Institute, Proton Therapy- Radiation Oncology, Miami, USA

**Purpose**: To develop a highly-efficient planning and delivery methodology for spatially-fractionated radiation therapy (GRID) with proton pencil beam scanning (PBS).

**Methods**: For a large, symptomatic, primary lung malignancy (839cc), two proton GRID plans, A and B, were generated (Figure 1). Plan A had 31 energy layers with a single spot at each GRID location and energy layer for a total of 313 spots with a maximum MU/spot of 48. Plan B had 101 energy layers, and at each energy layer/GRID location, multiple spots were placed with a spacing of 0.1cm. This limited the maximum MU/spot to 4.4 and resulted in 14,965 total spots. Delivery and plan quality were reviewed in RayStation 9A and verified by 2D absolute dose measurements at 4 depths using an IBA Matrixx PT (upsampled measurement resolution of 3.8mm).

**Results**: Both plans delivered similar GRID patterns (Figure 1). Average dose to GTV and body were 3.7 and 0.4Gy, and 3.8 and 0.41Gy in plan A and B, respectively. Plan A had a larger peak-to-valley ratio than plan B (5.6 vs 4.4) (Figure 1e) and delivered 3 times faster. Average gamma index results are listed in Table 1 using various criteria.

**Conclusion**: Proton GRID therapy utilizing a single spot per GRID location per energy layer at an energy spacing proportional to the spot size provides superior peak-to-valley ratio and efficient treatment delivery without compromising the quality of the spatial fractionation. Standardization of treatment planning techniques for SFRT is needed to allow for comparative clinical trials to be performed.

### P 175 - Challenges in long-term sustainability of particle therapy accelerator technology in view of RF and instrumentation electronics

#### Claus Schmitzer^1^, Matjaz Repovz^1^, Markus Wolf^1^, Miha Cerv^1^, Matthias Kronberger^2^, Christoph Kurfürst^1^, Borut Baricevic^3^, Ales Bardorfer^3^, Peter Paglovec^3^, Manuel Cargnelutti^3^

##### *^1^EBG Medaustron, Manufacturer Therapy Accelerator, Wr.Neustadt, Austria*

*^2^EBG Medaustron, Operations & Service, Wr.Neustadt, Austria*

*^3^Instrumentation Technologies, Development, Solkan, Slovenia*

The MedAustron Ion Therapy Centre is a synchrotron-based particle therapy facility located in lower Austria which delivers proton and carbon beams for clinical treatments of several hundred patients per year. The long-term sustainability of such a complex and unique machine is a demanding task which requires a high effort from the engineering and development department to maintain operations and hardware support. The MedAustron treatment centre was intended for an operational duration of 33 years which exceeds the life cycle of many technologies, especially electronics, and sometimes even the lifetime of suppliers. Already now, six years since the start of clinical operation, MedAustron is experiencing end-of-life issues concerning crucial control electronics. A potential solution to this problem is to rely on basic and robust, yet versatile systems which can be set up in many different applications, therefore facilitating hardware support both for the local engineering teams as well as for external suppliers. An example of this sustainability concept, the commonly developed low-level radiofrequency (LLRF) system based on a commercial MicroTCA platform is presented. Its versatile design qualifies this setup to relieve several systems in the existing accelerator setup resulting in an easier support structure. It will supply not only MedAustron's four resonant Linac structures but shall also replace the existing synchrotron LLRF system and feature active RF knock out exciter control. In addition, it will provide synchronized readout electronics for phase probe measurements in the Linac as well as aeveral pick up systems in the synchrotron, thus unifying eight systems.

**Table:** 

Table of Contents	
Program Description	pg. 306
2022 Committees	pg. 307
Oral Presentations	pg. 307
Poster Presentations	pg. 387

